# Advanced leukocyte classification using attention mechanisms and dual channel U-Net architecture

**DOI:** 10.1038/s41598-025-96918-3

**Published:** 2025-04-22

**Authors:** Gauri Kalnoor, Vijayalaxmi Kadrolli

**Affiliations:** 1 Department of CSE, Manipal Institute of Technology Bengaluru, Manipal Academy of Higher Education, Manipal, 560064 India; 2 Department of IT, Terna Engineering College, Navi Mumbai, India

**Keywords:** Attention, Blood smear, Datasets, Dual channel U Net, Dung beetle, Leukocyte, Levy flight, Segmentation, Machine learning, Computational biology and bioinformatics, Diseases, Medical research, Engineering

## Abstract

Leukocytes or white blood cells plays an important role in protecting the body from various contagious diseases and infectious agents. Different conventional leukocyte analysis approaches often face several problems like inaccuracies, demanding the need for sophisticated approaches to improve diagnostic precision. Therefore, a holistic structure namely a novel Attention-based Dual Channel U-shaped Network (ADCU-Net) utilizing three datasets is introduced in this paper for effective leukocyte classification. The image quality is boosted in the preprocessing phase through noise reduction, contrast enhancement, and background removal, significantly improving clarity. Then, the Dung Beetle Optimization (DBO) algorithm enhanced with Levy flight optimization is implemented for effective image segmentation processes. A dung beetle with a levy flight strategy assists in streamlined exploration of the search space thereby the detection and delineation of specific regions within images are improved, which results in higher boundary detection accuracy. The evaluation of major quantitative measures such as standard deviation, mean and entropy is comprised in the feature extraction process which offers crucial insights into the structural characteristics of leukocytes. Finally, a novel ADCU-Net model is utilized for the effective classification process. This ADCU-Net model is particularly selected to effectively capture various features and preserve spatial data, achieving98.4% accuracy. Overall, this paper highlights the performance of integrated sophisticated deep-learning structures for accurate leukocyte classification and segmentation, enabling the path for improved diagnostic tools in clinical settings.

## Introduction

Blood is an essential and dispersed fluid that carries and transfers nutrients, oxygen, and energy to all body cells by eliminating waste and carbon dioxide^1^. The main function of Red Blood Cells (RBCs) is to carry oxygen from the lungs to the rest of the body, while WBCs protect the body by fighting against many bacterial and viral diseases^2^. Leukocyte segmentation and classification represent an urgent component in clinical imaging investigations, particularly within hematology settings. The leukocyte segmentation comprises embedding and imaging individual WBCs within complex microscopic films. This task is extremely challenging due to the variations in cell shapes, sizes, and potential coverage of cells in dense areas of images^3^. Also, changes in imaging conditions and illumination results in additional difficulty. Accurate segmentation is significant for research applications, where the errors result in ineffective analysis. Thus, the segmentation accuracy is enhanced by employing sophisticated image processing techniques^4^.

Various conventional machine learning (ML) approaches have been developed to classify WBCs in microscopic blood smear images^5^. Although the results based on conventional approaches show many merits, there are several challenges such as the classification performance of conventional ML approaches depends on feature extraction and feature selection. This difficulty is tackled by integrating many Deep Learning (DL) approaches based on Convolutional Neural Networks (CNNs)^6^. Different DL approaches such as Convolutional Neural Networks (CNNs) are involved to improve the detection and segmentation accuracy of leukocyte in clinical images^7^. The significant features are extracted by the CNN from the cell images in which automatic detection and classification of leukocyte is achieved based on cell morphology. Accurate cell boundary detection is effectively achieved by several segmentation approaches like U-Net^8^. These DL models are trained on larger annotated datasets to attain higher accuracy in classifying the leukocyte subtypes such as neutrophils and lymphocytes. Thus, the diagnostic procedures are enhanced by these approaches in terms of pathology and hematology^9^.

Overall, the accuracy and efficiency of medical diagnostics are improved by the effective classification and segmentation of leukocytes by employing deep learning approaches. Thus, advanced structures such as CNNs and U-Net are leveraged for classification and segmentation respectively. These approaches automate the detection and classification of leukocyte subtypes with high accuracy^10^.

Importantly, a growing number of studies have identified adverse health effects related to environmental Cu exposure and its association with the occurrence of many diseases, including female infertility^11^. *The absolute network-based penalty* enables the model to integrate the feature network knowledge and helps select higher reproducibility genes^12^. Research has shown that lncRNA can be expressed through differences involved in the pathogenesis of diseases such as inflammation, metabolic diseases, and cancer^13^. Deep learning methods based on generative adversarial networks are used in various research fields due to their powerful generative capabilities, especially image super-resolution^14^. CAR domain design of adoptive cell therapy, which leads to differences in antitumor activity and triggered antitumor potential, remains poorly understood for macrophages^15^. Efficient sample preparation like sorting (i.e., separating target cells from the mixed population) and desalting (i.e., moving the cells off non-volatile salt solution) is urgently required in single-cell MS^16^. The modification is a prevalent form of post-transcriptional modification (PTM) found in various types of RNA^17^. Conventional methods of diagnosing dental diseases, like visual examination and radiographic testing, depend on qualified medical professionals and can be incredibly labor-intensive and imprecise in their diagnosis^18^. The medical data is initially massively pre-processed, where the data is purified with various mechanisms, including missing values resolution, data transformation, and the employment of normalization procedures^19^. Mammography involves imaging the same breast from two angles: the Medio lateral oblique (MLO) and the craniocaudally (CC)^20^.

### Motivation

The driving force behind this investigation stems from the aim to improve the accuracy of leukocyte classification and segmentation using sophisticated image analysis approaches. The major aim of this research is to set new standards by introducing a comprehensive framework namely Attention-Based Dual Channel U-Shaped Network (ADCU-Net) and segmentation through the Dung Beetle Optimization (DBO) algorithm integrated with the Levy flight for enhanced segmentation performance. A dung beetle with a levy flight strategy assists in streamlined exploration of the search space thereby the detection and delineation of specific regions within images are improved, which results in higher boundary detection accuracy. The potential implications of this work go beyond academic concerns; it aims to provide healthcare professionals with the best diagnostic tools, enabling them to make quick and accurate decisions in patient care. This research endeavours to make a significant contribution to the fields of medical imaging and hematology by overcoming the problems of existing approaches and ultimately improving clinical outcomes and improving healthcare practices.

### Novelty

*Advanced classification structure*: The introduction of the Attention-based Dual Channel U-shaped Network (ADCU-Net) represents a notable improvement in leukocyte classification. This model effectively captures diverse features through its dual-channel structure, enabling it to simultaneously process diverse aspects of the input data.

*Innovative segmentation method*: The Dung Beetle Optimization (DBO) algorithm enhanced with Levy flight strategy is implemented for effective image segmentation processes. A dung beetle with a levy flight strategy assists in streamlined exploration of the search space thereby the detection and delineation of specific regions within images are improved, which results in higher boundary detection accuracy.

*Effective feature extraction*: In this research, feature extraction is comprehensive by applying major quantitative measures such as standard deviation, mean, entropy and so on which offer crucial insights into the structural characteristics of leukocytes, and assist in enhanced classification.

### Contribution

*Classification accuracy*: A novel ADCU-Net model is developed for the effective classification of leukocytes. This ADCU-Net model is particularly selected to effectively acquire various features and preserve spatial data, achieving an exceptional accuracy over all other traditional approaches, exhibiting the model’s capability to accurately detect various types of leukocytes.

*Segmentation precision*: The segmentation of leukocytes is performed by applying the Dung Beetle Optimization (DBO) algorithm enhanced with Levy flight strategy, which attained a Dice coefficient of 0.94. The performance of a model in the accurate detection and delineation of cell boundaries is defined by this dice score, representing an effective method to segmentation that can handle the complexities of medical images.

*Feature extraction insights*: In this study, a diverse set of statistical measures such as mean, standard deviation, correlation, entropy and so on are applied for effective feature extraction, which enhanced the input data. The integration of these statistical measures provided crucial insights into leukocyte morphology, further enhancing the classification accuracy.

*Validation of methodologies*: The experimental findings validate the performance of both ADCU-Net and the DBO algorithm, enhancing their prospects for real-world applications. Substantial evaluation metrics reveal these approaches as important tools for clinical diagnosis, enabling a path to improved patient care.

The rest of the paper is structured as follows. In Section "Literature survey", the prior literature works regarding leukocyte classification are reviewed. The proposed methodology explaining different pivotal phases is presented in Section "Proposed methodology". In Section "Results and discussions", the results and discussions are analysed, Section "Challenges in deploying the ADCU-Net model in clinical settings and potential solutions" explains the Challenges In Deploying The Adcu-Net Model In Clinical Settings And Potential Solutions and finally, Section "Challenges in deploying the ADCU-Net model in clinical settings and potential solutions" concludes the article.

## Literature survey

Asghar et al.^21^ presented the automatic classification of ten blood cell subtypes by utilizing transfer learning through pre-trained convolutional neural networks. In this paper, transfer learning with a series of pre-trained Convolutional Neural Network (CNN) models including ResNet-101, InceptionV3, and DenseNet-201 were implemented to the Peripheral Blood Cells dataset (PBC). The blood cell images from the LISC, Kaggle and PBC datasets were used for effective testing of the proposed transfer learning CNN model. Thus, the experimental analysis exhibited that the accuracy of the proposed transfer learning CNN model in classifying the leukocytes was 98.79, 99.68 and 99.91% based on the LISC, Kaggle and PBC datasets respectively which was better than that of all other existing approaches. Meanwhile, higher execution time was the major drawback.

Khan et al.^2^ introduced the convolutional neural network coupled with a dual attention network for effective leukocytes detection. The major objective of this paper was to improve the medical hematology systems and accelerate the diagnosis process. A dual attention mechanism was integrated with the proposed model to enhance the generalization and overall efficiency of a proposed model. In this experiment, the PBC, LISC and Raabin-WBC benchmark datasets were utilized for the effective validation process. The broad analysis revealed that the accuracy of the proposed convolutional neural network coupled with a dual attention network was 99.83, 99.35, and 99.60% respectively. Thus, the results proved that the proposed model outperformed all other existing models in the detection and classification of leukocytes. On the other hand, computational complexity was the major drawback.

Anand et al.^3^ presented a deep Convolutional Neural Network (CNN) model is utilized on the collected dataset to perform feature extraction and classification. In this experiment, a publicly accessible dataset from Kaggle of 12,444 images of four types of leukocytes were utilized for the effective experimentation. After an extensive analysis, the results revealed that the accuracy and precision of the proposed model were 97.98% and 97.97% respectively which was superior to all other existing models in terms of classifying the leukocytes. Meanwhile, lack of attention mechanisms was the major drawback of this experiment.

Zonyfar et al.^22^ developed the Convolutional Attention BLSTM network (CAB-Net) for enhanced leukocyte classification. In this paper, end-to-end deep learning-based model named CAB-Net is proposed by integrating the convolutional neural network with an attention mechanism and BLSTM to classify the leukocyte effectively. A stack of convolutional and pooling layers were utilized by the proposed CAB-Net model for effective feature extraction. Thus, the experimental analysis exhibited that the proposed CAB-Net outperformed all other existing models in terms of classifying the leukocytes. Meanwhile, insufficient dataset was the major drawback.

Fathy et al.^23^ introduced the Novel Meta-Heuristic Optimization Algorithm in White Blood Cells (WBCs) classification. Different models such as Visual Geometry Group (VGG) and AlexNet are used for the effective feature extraction. In addition, the significant features are effectively extracted using the Binary Border Collie Optimization (BBCO). Here, a hybrid ResNet101-BBCO-SVM was developed by integrating the ResNet101, BBCO feature reduction, and Support Vector Machine (SVM) classifier. Thus, the simulation analysis exhibited that the accuracy of the proposed ResNet101-BBCO-SVMmodel was 99.21%. Thus, the proposed ResNet101-BBCO-SVM outperformed all other existing approaches in terms of classifying the WBCs. Meanwhile, limited data samples were the major drawback.

Saikia et al.^24^ developed the Optimized Support Vector Machine by utilizing Whale Optimization algorithm for acute lymphoblastic Leukemia detection from microscopic blood smear images. In this paper, the segmentation of White Blood Cells is carried out using the CIEL*a*b colour-based K-means clustering with a marker-controlled watershed. The important features are verified by conducting the ANOVA test. The Zero Phase Component Analysis whitening was introduced to eliminate the feature’s covariance structure. Thus, the experimental analysis revealed that the accuracy of the proposed WOA-SVM was 98.42% on ALL-IDB1 dataset, better than that of all other existing models. Meanwhile, higher computational time was the major drawback.

Rivas et al.^25^ presented the automatic base-model selection for white blood cell image classification by utilizing meta-learning. In this paper, a novel approach to automatically select samples to tackle WBC classification tasks was proposed. The meta-level and base-level learning were comprised in the proposed model. The selected models ere adapted by applying the learning rate finder approach. In this experiment, the Raabin dataset, BCCD dataset, and UACH dataset were utilized for the effective validation purposes. The performance of a proposed model was better than that of all other existing models in terms of classifying the white blood cells. On the other hand, increased computational overhead was the major drawback.

Veeraiah et al.^26^ developed a concept to classify the Leukemia from blood smear images using Mayfly Optimization with Generative Adversarial Network-Based Deep Learning Method (MayGAN). In this paper, the MayGAN was introduced to improve the feature extraction and classification of Leukemia. In the feature extracted model, the Generative Adversarial System (GAS) and Principal Component Analysis (PCA) are combined for the effective classification of blood cancer in the given input data. Thus, the experimental analysis proved that the proposed MayGAN model outperformed all other existing models in terms of classifying the leukemia from blood smear images. Meanwhile, insufficient data samples were the major drawback.

Prasad et al.^27^ introduced the Deep U_ClusterNet for precise diagnosis and future treatment plan. In this paper, an automated Deep U-Net with clustering-based segmentation (Deep U_ClusterNet) was developed for effective segmentation. From an extensive analysis, the accuracy, precision, recall, specificity and Dice similarity coefficient (DSC) of the proposed Deep U_ClusterNet model were 98.2, 95.7, 99.8, 98.3 and 95.1% respectively which was better than that of all other existing models in terms of segmenting the white blood cells. Meanwhile, large number of computational resources were required.

Leng et al.^28^ developed the deep learning detection network for peripheral blood leukocytes based on improved detection transformer Initially, the vision transformer and deformable attention module into the DETR model was introduced for enhancing the convergence speed and overall performance of a proposed model. Next, an enhanced model was trained using the obtained dataset to achieve the pretrained weights. Then, the transfer learning on the modified Raabin leukocyte dataset was implemented to determine an effective and optimal model. Thus, the results proved that the performance of the proposed model was better than that of all other existing approaches. Meanwhile, computational complexity was the major drawback.

### Limitations and research gaps

Existing leukocyte classification approaches face several general challenges. There are many techniques that rely heavily on specific datasets, thereby limiting their performance when implemented in a real-world scenario, hence failing to classify the different types of leucocytes. When compared to the state-of-the art deep learning models the conventional machine learning techniques show poor performance with low accuracy in classification. In addition to this, several systems fail in the precise differentiation of the similar leukocyte subtypes making the diagnostic procedure more complicated. The problem of misclassification, particularly between rare and common leukocytes, results in false positives or negatives, further reducing reliability. Several feature selection approaches that are most widely used lack sufficient precision or adaptability to accommodate the complex and evolving nature of cell morphology. Finally, the need for real-time analysis and adaptability regarding newly discovered leukocyte characteristics remains a crucial challenge, emphasizes the need for more advanced and flexible classification solutions.

## Proposed methodology

**Fig. 1:**
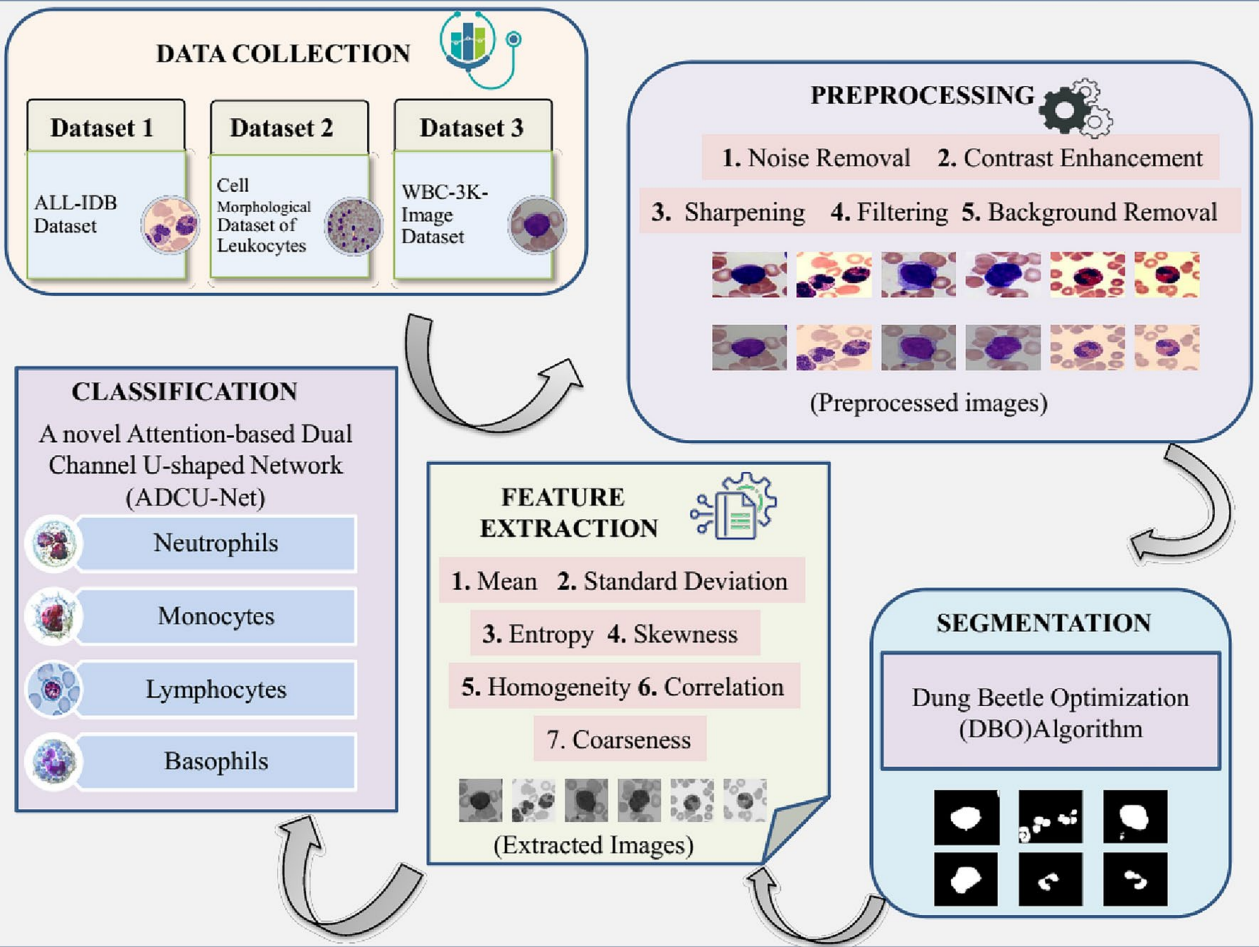
Conceptual framework of the proposed model.

Figure 1 depicts the image analysis process for cell classification and segmentation. Initially, data collection is carried out using three datasets including ALL-IDB, Cell Morphological Dataset of Leukocytes, and WBC-3 K-Image Dataset. Then obtained data are preprocessed to improve the image quality for further analysis. The preprocessing is performed using several approaches such as noise removal, background removal contrast enhancement, filtering, and sharpening. The Dung Beetle Optimization (DBO) algorithm is implemented for the segmentation process in which the specific regions in images are detected effectively. Then, feature extraction is carried out by evaluating the statistical measures like mean, standard deviation, entropy, skewness, homogeneity, correlation, and coarseness. Lastly, a novel Attention-based Dual Channel U-shaped Network (ADCU-Net) is used for the effective classification of leukocytes by utilizing the extracted features.

The Attention-based Dual Channel U-shaped Network (ADCU-Net) plays a central role in classifying leukocytes. To improve understanding of its decision-making process, several strategies can be employed to analyze its internal operations and enhance interpretability.

1. *Attention map analysis*:
  The objective is to highlight areas of the input images that the model focuses on during classification.

The attention modules integrated into ADCU-Net direct the network’s focus to the most relevant image regions. Visualizing these attention maps can reveal which areas—such as the cell nucleus or boundaries—significantly influence the classification outcome.

2. *Understanding dual channel contributions*
  *Channels’ roles*:
    The first channel processes spatial information, helping identify structures like cell shapes and edges.
    The second channel captures contextual information, including texture and intensity variations.
  *Analysis*: By examining intermediate outputs from each channel, we can gain insight into how the spatial and contextual data are combined to refine the model’s understanding of leukocytes.
3. *Progressive feature refinement*
  *Layer-wise Interpretation*: Each layer within ADCU-Net refines the input data differently.
    Initial layers focus on fundamental features like edges and simple shapes.
    Deeper layers process higher-level details, such as intricate textures or cell patterns.
  *Benefit*: Visualizing outputs at different layers demonstrates how the model transforms raw data into task-specific features for classification.
4. *Saliency maps and Grad-CAM*: The objective is to visualize how sensitive the model’s output is to various parts of the input image. Tools such as Gradient-weighted Class Activation Mapping (Grad-CAM) create heatmaps that show which regions of the image had the most influence on the model’s final decision.

*Application*: For example, when classifying a neutrophil, the heatmap might emphasize the nucleus, providing insights into the features the model prioritizes.

5. *Feature contribution analysis*: The objective is to determine the importance of statistical features (e.g., mean, entropy, homogeneity) in influencing model predictions. Techniques like Shapley Additive Explanations (SHAP) or permutation importance can be used to rank these features by their impact on classification accuracy.

*Advantage*: This evaluation ensures transparency in how specific image properties contribute to the network’s decisions.

6. *Error pattern analysis* is to understand patterns in misclassifications and areas where the model may need improvement. By comparing attention maps and feature activations for correctly and incorrectly classified images, researchers can identify potential biases or limitations in the model.

### Data collection

In this section, a detailed description is provided on the basis of each dataset, which typically includes images of various leukocyte types, interpreted with relevant morphological features, to assist thorough analysis and classification.

*ALL-IDB Dataset (Dataset 1)*: The ALL-IDB (Acute Lymphoblastic Leukemia Image Database) is developed to classify the leukemic cells, particularly denoting acute lymphoblastic leukemia. Numerous images of various types of leukemic cells, such as lymphoblasts and normal cells, with images typically sized at 512 × 512 pixels are comprised in this dataset. Generally, the images are in the form of JPEG or PNG. In this dataset, 1500 images and 500 images are used for both training and testing purposes respectively that offers a strong foundation for to develop and validate different classification algorithms. Thus, this dataset is accessible by using (https://www.kaggle.com/datasets/sizlingdhairya1/all-idb-images).

*Cell morphological dataset of leukocytes (Dataset 2)*: In this dataset, single-cell images of leukocytes are provided, focusing on morphological analysis for research purposes. Different types of white blood cells, such as neutrophils and lymphocytes, with image sizes generally varying around 512 × 512 pixels are comprised in this dataset. Generally, the images are in the form of JPEG or PNG, interpreted with relevant morphological features, to assist thorough analysis and classification. In this dataset, 1000 images and 500 images are used for both training and testing purposes respectively, enabling thorough analysis and model training. Thus, this dataset is accessible by using (https://www.kaggle.com/datasets/lsaa2014/single-cell-morphological-dataset-of-leukocytes/data).

*WBC-3 K-Image dataset (Dataset 3)*: The WBC-3 K dataset comprises different white blood cell images, focusing on the classification process within clinical imaging. In this dataset, different classes of white blood cells such as monocytes, eosinophils, and basophils are included. Generally, the images are in the form of JPEG sized at 256 × 256 pixels or 512 × 512 pixels. Based on the type of white blood cells, each image is labeled. In this dataset, 3000 images including 2,400 images and 600 images are utilized for training and testing purposes. Thus, effective machine learning models are developed to classify the white blood cells by this comprehensive collection. Thus, this dataset is accessible by using (https://www.kaggle.com/datasets/quangnguynvnnn/wbc-3k-image).

The selection of the ALL-IDB dataset, the leukocyte cell morphology dataset, and the WBC-3 K-image dataset was carefully made to ensure a diverse and comprehensive representation of leukocyte images. These datasets were chosen based on the following considerations:

1. *Diversity of leukocyte types*: The datasets collectively include images of various types of white blood cells (WBCs), such as neutrophils, lymphocytes, monocytes, and basophils, ensuring broad coverage of cell types for classification tasks.
2. *Variety in imaging conditions*: These datasets feature images captured under different staining techniques, imaging resolutions, and laboratory environments. This diversity makes the model robust and capable of generalizing to real-world scenarios.
3. *Data quality and annotations*: Each dataset provides high-quality images along with detailed annotations, which are essential for tasks such as segmentation and classification. This ensures accurate extraction and reliable model performance.
4. *Benchmarking and comparability*: ALL-IDB, leukocyte morphology datasets, and WBC-3 K are widely recognized in the research community. Using these datasets allows for benchmarking the model’s performance against other state-of-the-art methods.

The combination of these three datasets provides a comprehensive foundation for training and validating the ADCU-Net model. However, to further evaluate the sufficiency of coverage:

- *Population diversity*: The datasets should ideally represent a wide range of patient demographics, including age, gender, and medical conditions, to ensure the model’s applicability across different clinical scenarios.
- *Rare leukocyte types*: If rare WBC types or abnormal cell morphologies are underrepresented, additional datasets or synthetic data augmentation may be necessary to enhance coverage.
- *Imaging variability*: Ensuring that the datasets capture diverse imaging conditions (e.g., different microscopes and staining protocols) is crucial for robust real-world performance.

### Data preprocessing

Image preprocessing comprises several steps taken to improve the image quality before further analysis. The major objective of this phase is to boost the image’s data content by improving the crucial features like contrast and minimizing the noise. Thus, the obtained image becomes more suitable for further processing tasks by enhancing these features.

*Noise reduction*: Noise reduction involves eliminating unwanted variations in pixel values to obtain an enhanced image^29^. A common method of noise reduction is to use a filter such as a median filter. Let $J\left( {j,\,k} \right)$ is the pixel value at a position $\left( {j,\,k} \right)$, then the median filter is derived in the below equation.

1 $$J^{\prime} \left( {j,\,k} \right) = \frac{1}{M}\sum\limits_{(n,\,m) \in \gamma } {J\left( {n,\,m} \right)}$$

From the above equation, the number of pixels is represented by $M$ and $\gamma$ signifies the pixel’s neighborhood $\left( {j,\,k} \right)$.

This filtering process smoothens the image, reduces pixel-level variations, and retains essential boundaries for accurate segmentation.

*Contrast enhancement*: Contrast enhancement involves adjusting brightness and contrast to make features more distinct. Here, the histogram equalization is applied to perform contrast enhancement. For histogram equalization, the Cumulative Distribution Function (CDF) is utilized to map the pixel values.

2 $$J^{\prime} \left( {j,\,k} \right) = CDF\left( {J\left( {j,\,k} \right)} \right)$$

Contrast Enhancement emphasizes leukocyte structures, such as the nucleus and cytoplasm, making them easier to segment and analyze.

*Background removal*: The object of interest is isolated by removing distracting background elements. Background removal can be done by thresholding.

3 $$J^{\prime} \left( {j,k} \right) = \left\{ {\begin{array}{*{20}l} {J\left( {j,k} \right)} \hfill & {{\text{If}}\;J\left( {j,k} \right) > U} \hfill \\ 0 \hfill & {{\text{Otherwise}}} \hfill \\ \end{array} } \right.$$

From the above equation, a predefined threshold value is represented by $U.$

By removing irrelevant elements, this step ensures that the focus remains on leukocytes, reducing distractions and computational complexity for subsequent stages.

*Filtering*: Using techniques like Gaussian filters to smooth the image while preserving crucial details. The equation below derives the pixel value for Gaussian filter.

4 $$J^{\prime} \left( {j,\,k} \right) = \sum\limits_{(n,\,m) \in \gamma } {J\left( {n,\,m} \right) \cdot H\left( {j - n,\,k - m} \right)}$$

From the above equation, $H\left( {j - n,\,k - m} \right)$ represents the Gaussian function.

*Sharpening*: During the sharpening process, edges and fine details are improved to further accentuate features. Here, Laplacian operators are applied for effective image sharpening.

5 $$J^{\prime} \left( {j,\,k} \right) = J\left( {j,\,k} \right) + \nu \,\nabla^{2} J\left( {j,\,k} \right)$$

From the above equation, the scaling factor and laplacian of the image is represented by $\nu$ and $\nabla^{2} J\left( {j,\,k} \right)$ respectively.

#### Contribution to improved classification

*Enhanced clarity*: Preprocessing ensures that images are free from noise, with well-defined features that improve both human and algorithmic interpretation.

*Accurate segmentation*: Clearer boundaries enable precise detection and delineation of leukocyte regions during segmentation.

*Better Input quality*: The preprocessing steps provide clean, high-quality input data for deep learning models, such as ADCU-Net, improving feature extraction and classification accuracy.

*Reduction in errors*: Removing noise and irrelevant data helps minimize misclassifications, allowing the model to focus on the relevant features of leukocytes.

### Segmentation using novel dung beetle optimization with levy flight strategy

In this subsection, we used local region-based architecture to guide active contours. The general concept of curve fitting based on local area is to delineate a locally defined set of energy at each point of the specified curve.

*Nuclei extraction:* At each point of a local region, energy intensity based local energy optimization was carried out. Neighboring regions of the curve were divided into two regions including local outer and inner curves and a complete regional energy analysis is carried out.

$J_{D}$ represents the image comprising closed contours. Generally, it defines the set of signed distance function at zero level $\eta.$

6 $$J_{D} = \left\{ {y|\eta \left( {y = 0} \right)} \right\}$$

*Region Refinement:* The interior of $J_{D}$ is specified by the below expression of smoothed Heaviside function.

7 $$I\mu \left( y \right) = \left\{ {\begin{array}{*{20}l} {1,} \hfill & {\eta \left( y \right) < - \varepsilon } \hfill \\ {0,} \hfill & {\eta \left( y \right) > \varepsilon } \hfill \\ {1/2\left\{ {1 + \frac{\eta }{\varepsilon } + \frac{1}{\varphi }\sin \frac{\varphi \eta \left( y \right)}{\varepsilon }} \right\},} \hfill & {otherwise} \hfill \\ \end{array} } \right.$$

From the above equation, the nearer curve regions are specified by using the $\eta \left( y \right)$. Similarly, $\left( {1 - I\left( {\eta \left( y \right)} \right)} \right)$ represents the exterior of $J_{D}$. Here, the second spatial variable with $y$ is considered as $y^{\prime}$. Both the $y$ and $y^{\prime}$ is utilized as independent special variables to define all the points in $\varpi$. Thus, the mask function of a local region $C\left( {y,\,y^{\prime} } \right)$ is initiated by utilizing the specified radius $s$, $y$ and $y^{\prime}$ and it is expressed as follows.

8 $$C\left( {y,\,y^{\prime} } \right) = \left\{ {\begin{array}{*{20}l} {1,} \hfill & {||y - y^{\prime} || < s} \hfill \\ {0,} \hfill & {otherwise} \hfill \\ \end{array} } \right.$$

Here, the mean intensities of both interior and exterior region-based localized energy function, $G$ are represented by $v_{y} \,and\,\,u_{y}$ and it is introduced by using the below equation.

9 $$G_{1} = \eta \left( {y^{\prime } } \right) \cdot \left( {J_{{y^{\prime } }} - v_{y} } \right)^{2} + \left( {1 - I\eta \left( y \right)} \right) \cdot \left( {J_{{y^{\prime } }} - u_{y} } \right)^{2}$$

The derivative of $G$ was considered based on the $\eta \left( y \right)$ to determine the estimated curve for $\eta$. The central points of the nuclei are obtained from the $J_{DG}$. In case of complicated nuclei detection, the boundary extraction was carried out. The mean intensities of both the interior and exterior regions are represented by a localized energy function, introduced through a specific equation. The derivative of this function is utilized to estimate the curve for the nuclei. The central points of the nuclei are determined from the contours. In cases of complex nuclei detection, boundary extraction is performed using techniques like the Canny edge detector and discrete morphological edge thinning for effective nuclei region boundary extraction.

To enhance the contour estimation process, we incorporate a Levy flight-based dung beetle optimization algorithm. This optimization technique improves the sampling of partial view kernels and facilitates more effective segmentation by exploring the search space efficiently. The Levy flight mechanism helps in achieving a balance between exploration and exploitation, leading to more accurate boundary evaluation and nuclei detection. Leukocyte segmentation is a crucial task in medical imaging, especially in analyzing blood samples for diagnosing various conditions. The Dung Beetle optimization algorithm, inspired by the foraging behavior of dung beetles, along with the Levy flight strategy, can enhance segmentation techniques by effectively exploring the search space^30^.

The DBO algorithm emulates the behavior of dung beetles, particularly their systematic process of:

- Searching for resources (mimicking global and local optimization).
- Rolling dung balls (simulating an optimization trajectory).
- In image segmentation, this process translates into finding the optimal parameters or thresholds to segment the regions of interest (e.g., leukocytes) from the background.

*Dung beetle optimization algorithm with levy flight strategy*: The Dung Beetle Optimization algorithm (DBO algorithm) is a swarm intelligence optimization algorithm based on the habits of dung beetles. The mathematical expressions for the four major categories such as ball rolling, reproduction, small dung beetle and thief are discussed and derived in the following subsection.

As dung beetles roll the dung ball, they must ensure that their path is straight line based on the position of celestial bodies. The individual’s position is altered by this process, and it is expressed in the below equation.

10 $$Y_{m}^{j + 1} = Y_{m}^{j} + b \cdot l \cdot Y_{m}^{j - 1} + a \cdot \Delta y$$

11 $$\Delta y = \left\{ {Y_{m}^{j} - Y^{u} } \right\}$$

From the above equations, the $m{\text{th}}$ dung beetle position’s is represented by $Y_{m}^{j + 1}$ at $j\,th$ iteration, the natural coefficient and deflection coefficient are signified by $a \in \left( {0,\,1} \right)$ and $l \in \left( {0,\,0.2} \right)$. The ineffective position within the current position is represented by $Y^{u}$ and degree of variability in the light intensity is denoted by $y$.

The following expressions define the behavior of dung beetles.

12 $$\begin{gathered} Y_{m}^{j + 1} = Y_{m}^{j} + \tan \beta \left| {Y_{m}^{j} - Y_{m}^{j - 1} } \right| \hfill \\ 0 \le \beta \le \varphi \hfill \\ \end{gathered}$$

From the above equation, the reflection angle between the new and original direction of dung beetles is represented by $\beta$.

13 $$Y_{m}^{j + 1} = Y^{u} + A_{1} \cdot \left( {Y_{m}^{j} - LA_{1} } \right) + A_{2} \cdot \left( {Y_{m}^{j} - UA_{1} } \right)$$

From the above equation, the $m\,th$ hatching dung ball position’s is represented by $Y_{m}^{j}$ at $j\,th$ iteration. The two independent random matrices are represented by $A_{1}$ and $A_{2}$.

The position of the small dung beetle is updated after the specific area is determined and it is expressed by using the equation below.

14 $$Y_{j}^{m + 1} = Y_{j}^{m} + D_{1} \cdot \left( {Y_{j}^{m} - LA_{2} } \right) + D_{2} \cdot \left( {Y_{j}^{m} - UA_{2} } \right)$$

From the above equation, the position of the small dung beetle is represented by $Y_{j}^{m + 1}$, $D_{1}$ represents the random number that follows Gaussian distribution and $D_{2} \in \left( {0,\,1} \right)$.

The position of thief dung beetles is given in the below equation.

15 $$Y_{m}^{j + 1} = Y^{t} + Q \cdot e \cdot \left( {\left| {Y_{m}^{j} - Y^{u} } \right| + \left| {Y_{m}^{j} - Y^{ - } } \right|} \right)$$

From the above equation, $Q$ represents a constant value and random vector of size 1 is denoted by $e$.

*Levi Flight Strategy*: Levy flight (LF) belongs to a class of non-Gaussian stochastic processes where the distribution of stationary increments is developed by utilizing a Levy stationary distribution. The term “Levy” denotes the French mathematician Paul Pierre Lévy, who first studied Levy motion. The term “flight” refers to the distance of straight-line at its peak level between two points where an object covers in its motion without any pause or change of direction. Levy walk or LF is used interchangeably in literature. Birds are often observed to have limited or lack of knowledge of where resources are available. Contrasts arise irregularly due to the uneven distribution of prey in the levee plane^31^.

The Levy Distribution (LD) determines the step size of LF. LD represents the distribution of the sum of $A$ identically and independently distributed variables, with its Fourier transform attained from the following equation.

16 $$F_{A} \left( l \right) = \exp \,\left[ { - A\,\left| {\,l\,} \right|^{\delta } } \right]$$

The LD is expressed by the integral expressed below.

17 $$L\left( t \right) = \frac{1}{\varphi }\int\limits_{0}^{\infty } {\cos \left( {\kappa t} \right)f^{{ - \chi \eta^{\delta } }} e\kappa ,\quad \left( {0 < \delta \le 2} \right)}$$

From the above equation, the short time is represented by $\kappa$ at each step where $\chi$ represents constant, where the distribution scaling is controlled by taking any positive values. The $\chi$ value is similar to unity in maximum applications. Thus, the approximation of the above equation is derived below.

18 $$L\left( t \right)\sim t^{ - 1 - \delta }$$

From the above equation, $\delta$ represents the constant and it is known as the shape parameter, determining the weight distribution. In the $e$ dimensional space, the Brownian variance is attained by using the below equation.

19 $$\sigma^{2} \left( u \right) = \left| {w_{0} } \right|^{2} u^{2} + \left( {2cC} \right)u$$

From the above equation, the diffusion coefficient is represented by $C$ with the step size $t$ over the shortest time interval $\kappa$ at each step is expressed by using the below equation.

20 $$E = \frac{{t^{2} }}{2\kappa }$$

Levy flight optimization introduces random movements characterized by a heavy-tailed probability distribution. This enhances the DBO algorithm in the following ways:

#### Efficient search space exploration

Levy flight allows the algorithm to take a mix of short and long steps, effectively balancing local exploration and global search. This helps avoid getting stuck in suboptimal solutions and ensures a thorough exploration of the search space.

#### Faster convergence

The probabilistic nature of Levy flight accelerates the convergence process by focusing on regions with higher potential, reducing computation time while maintaining precision.

- *Improved boundary detection accuracy*

The combination of DBO and Levy flight enhances segmentation by:

- *Accurate region separation*
  It precisely identifies the optimal segmentation thresholds, enabling accurate separation of objects (e.g., leukocytes) from the background.
- *Noise resilience*
  The method is robust to image noise, ensuring that detected boundaries are sharp and well-defined even under challenging conditions.
- *Adaptability to Complex structures*
  The hybrid approach handles intricate and irregular boundaries effectively, ensuring high-quality segmentation across varying datasets.

- *Relevance in medical image segmentation*

This enhanced DBO algorithm is particularly beneficial for medical imaging tasks, such as segmenting leukocytes, due to:

- *High precision*
  It ensures that regions of interest are accurately detected, improving the reliability of downstream tasks like feature extraction and classification.
- *Computational efficiency*
  The optimized search mechanism reduces processing time, making it feasible for large datasets.
- *Versatility*
  The algorithm adapts to different image types and resolutions, ensuring consistent segmentation performance.

**Pseudocode:**
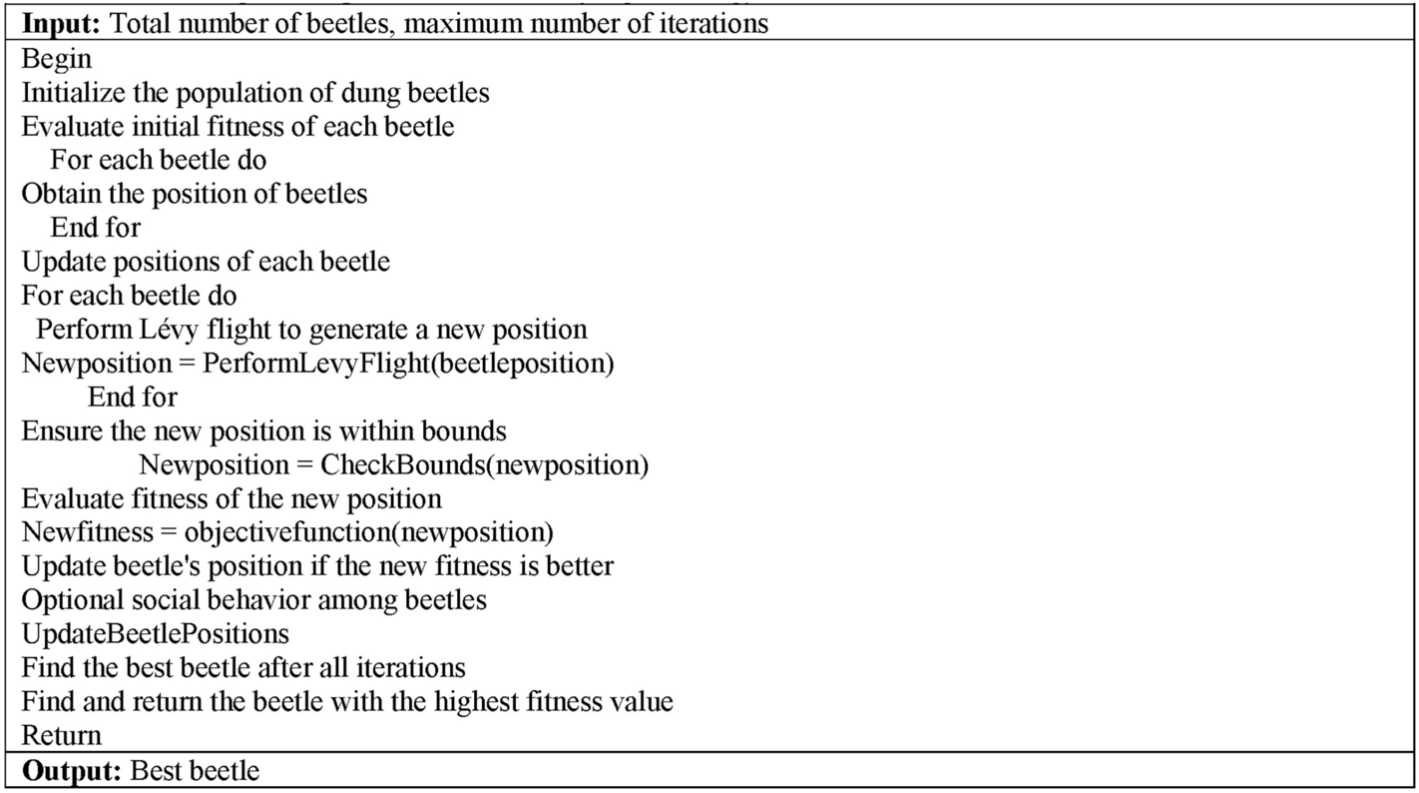
Dungbeetle optimization with levyflight strategy.

### Feature extraction

Feature extraction involves identifying and quantifying relevant characteristics that can be used for further processing and classification. This section outlines various statistical features derived from an image, each of which provides insights into its texture and intensity.

Feature extraction is a critical step in image analysis, aiming to quantify meaningful information from an image to facilitate classification and analysis tasks. In the context of leukocyte analysis, metrics such as standard deviation, mean, and entropy play a pivotal role in capturing the structural and statistical characteristics of cells.

*Mean*: The Mean of an image is obtained by adding an image total pixel value divided by of a specific image and then divided by the total pixel value of an image and it is derived using the below equation.

21 $$M = \left( {\frac{1}{m \times n}} \right)\sum\limits_{x = 0}^{m - 1} {\sum\limits_{y = 0}^{n - 1} {g\left( {x,{\mkern 1mu} y} \right)} }$$

*Standard Deviation*: The standard deviation is one of the major central moments where a given population is represented as the probability distribution and to evaluate the in homogeneity^32^. The better intensity level and contrast between the image edges are signified by best values.

22 $$SD\,\left( \rho \right) = \sqrt {\left( {\frac{1}{m \times n}} \right)\sum\nolimits_{x = 0}^{m - 1} {\sum\nolimits_{y = 0}^{n - 1} {\left( {g\left( {x,\,y} \right) - M} \right)^{2} } } }$$

*Entropy*: The randomness of a textured image is characterized by the entropy, and it is expressed by the below equation,

23 $$E = \sum\limits_{x = 0}^{m - 1} {\sum\limits_{y = 0}^{n - 1} {g\left( {x,{\mkern 1mu} y} \right)} } \log_{2} g\left( {x,{\mkern 1mu} y} \right)$$

*Skewness*: Skewness refers to a symmetry absence or symmetry attribute. $V_{k} \left( X \right)$ represent the skewness and the ransom variable is represented by $X$.

24 $$V_{k} \left( X \right) = \left( {\frac{1}{m \times n}} \right)\frac{{\sum {\left( {g\left( {x,\,y} \right) - M} \right)^{3} } }}{{SD^{3} }}$$

*Homogeneity or Inverted moment of Difference (IDM)*: The local consistency of an image is evaluated by the IDM. The mathematical expression for IDM is given below

25 $$IDM = \sum\limits_{x = 0}^{m - 1} {\sum\limits_{y = 0}^{n - 1} {\frac{1}{{1 + \left( {x - y} \right)^{2} }}g\left( {x,{\mkern 1mu} y} \right)} }$$

*Direction Moment*: Direction moment is the textured feature of the images, evaluated by considering the image’s alignment angle and it is expressed by using the below equation.

26 $$DM = \sum\limits_{x = 0}^{m - 1} {\sum\limits_{y = 0}^{n - 1} {g\left( {x,{\mkern 1mu} y} \right)} } \left| {x - y} \right|$$

*Correlation*: The spatial dependencies between the pixels are explained and determined by the correlation features and it is represented by using the below equation.

27 $$C = \frac{{\sum\nolimits_{x = 0}^{m - 1} {\sum\nolimits_{y = 0}^{n - 1} {\left( {x,\,y} \right)g\left( {x,\,y} \right) - M_{x} M_{y} } } }}{{\rho_{x} \rho_{y} }}$$

From the above equation, the standard deviation and mean in the vertical spatial domain is represented by $\rho_{y}$ and $M_{y}$ respectively. In the horizontal spatial domain, the standard deviation and mean are denoted by $\rho_{x}$ and $M_{x}$ respectively.

*Coarseness:* The texture is coarser when the window size is fixed. Thus, the coarser is the roughest layer and the fine textures are represented by smaller texture values. The Coarseness is derived by the below equation.

28 $$I = \frac{1}{{2^{m + n} }}\sum\limits_{x = 0}^{m - 1} {\sum\limits_{y = 0}^{n - 1} {g\left( {x,{\mkern 1mu} y} \right)} }$$

1. *Standard deviation: quantifying intensity variability*

The standard deviation measures the dispersion of pixel intensities around the mean. For leukocyte images, it provides valuable insights into the texture and contrast of the cells. A higher standard deviation indicates greater variation in intensity, which can signify distinct cellular boundaries, granules, or irregular textures within leukocytes. This metric helps differentiate between cell types based on the degree of variability in their internal structure or surrounding regions.

2. *Mean: determining average intensity*

Mean represents the average pixel intensity in the region of interest. This metric reflects the overall brightness of a leukocyte or its surrounding area. Leukocytes often have specific intensity patterns compared to the background or other objects. The mean value provides a baseline for distinguishing between different cells or structures. It is particularly useful for normalizing the image data and identifying cells with unique brightness levels relative to others.

3. *Entropy: Measuring texture complexity*

Entropy quantifies the randomness or complexity of pixel intensities within a region. It is especially useful for identifying structural details and variations in leukocytes. High entropy indicates complex texture or diverse intensity patterns, which are often characteristic of specific leukocyte types or abnormalities. Entropy aids in capturing fine details like granules, edges, or nuclear structures, making it essential for differentiating leukocytes with similar appearances.

These metrics collectively capture crucial features of leukocytes:

- *Standard deviation* highlights variations and structural irregularities.
- *Mean* provides a measure of general intensity levels.
- *Entropy* identifies intricate textures and complexity within cells.

Additionally, the below mentioned criteria of quality assessment are also necessary to enhance the overall results.

### A novel attention based dual channel U-shaped network (ADCU-NET) for leukocyte classification

The Attention-based Dual Channel U-shaped Network (ADCU-Net) is a novel architecture designed for the effective classification of leukocytes in medical images. Leveraging the strengths of attention mechanisms and U-Net architecture, ADCU-Net enhances the accuracy and efficiency of leukocyte detection and classification, which are crucial for diagnosing various blood disorders^32,33^.

#### Architecture of the attention-based dual channel U-shaped network (ADCU-Net)

The Attention-based Dual Channel U-shaped Network (ADCU-Net) is a specialized deep learning model designed for precise leukocyte classification. Its architecture is engineered to capture a wide range of features while preserving critical spatial information, ensuring high accuracy in medical image analysis. Below is an overview of its design and functionality:

*Dual Channel Design*: The dual channel structure is integral to processing both low-level and high-level features simultaneously. This allows the model to analyze detailed spatial information while also capturing abstract representations of the input data. One channel focuses on fine-grained spatial details such as edges and textures, while the other extracts broader semantic features, such as the overall morphology of leukocytes.

*U-shaped Architecture*: U-shaped architecture facilitates a symmetric encoder-decoder structure. This design is particularly effective in image segmentation and classification tasks because it allows the model to capture features at multiple resolutions.

- *Encoder*: The encoder compresses the input image by applying convolutional layers and pooling operations, extracting high-level abstract features.
- *Decoder*: The decoder progressively restores the spatial resolution using upsampling and concatenation layers, ensuring that fine-grained spatial information is retained.

*Attention mechanisms*: Attention modules are incorporated to enhance the model’s ability to focus on relevant regions in the image. This is crucial for distinguishing leukocytes from background noise or other irrelevant components. Attention mechanisms dynamically weigh the importance of specific features, highlighting regions that are most significant for classification.

*Skip connections*: Skip connections are utilized to bridge the encoder and decoder layers at corresponding levels. These connections mitigate the loss of spatial information during feature extraction. By transferring low-level features from the encoder directly to the decoder, the model effectively combines spatial and semantic information.

#### Feature capturing and preservation

- *Multi-scale feature extraction*: The ADCU-Net captures features at various scales, ensuring that both local and global patterns are analyzed. This is particularly important for distinguishing subtle differences between leukocyte types.
- *Spatial preservation*: Through dual channel design, skip connections, and attention modules, the model maintains spatial integrity, which is critical for accurately classifying cells based on their morphology.

#### Effectiveness in leukocyte classification

The ADCU-Net’s architecture, combining dual channels, attention modules, and a U-shaped design, allows it to process medical images with exceptional precision. It not only captures a wide range of features but also retains spatial data essential for distinguishing between different leukocyte types. This comprehensive approach makes ADCU-Net highly effective for applications in medical diagnostics, where accuracy and reliability are paramount.

**Fig. 2:**
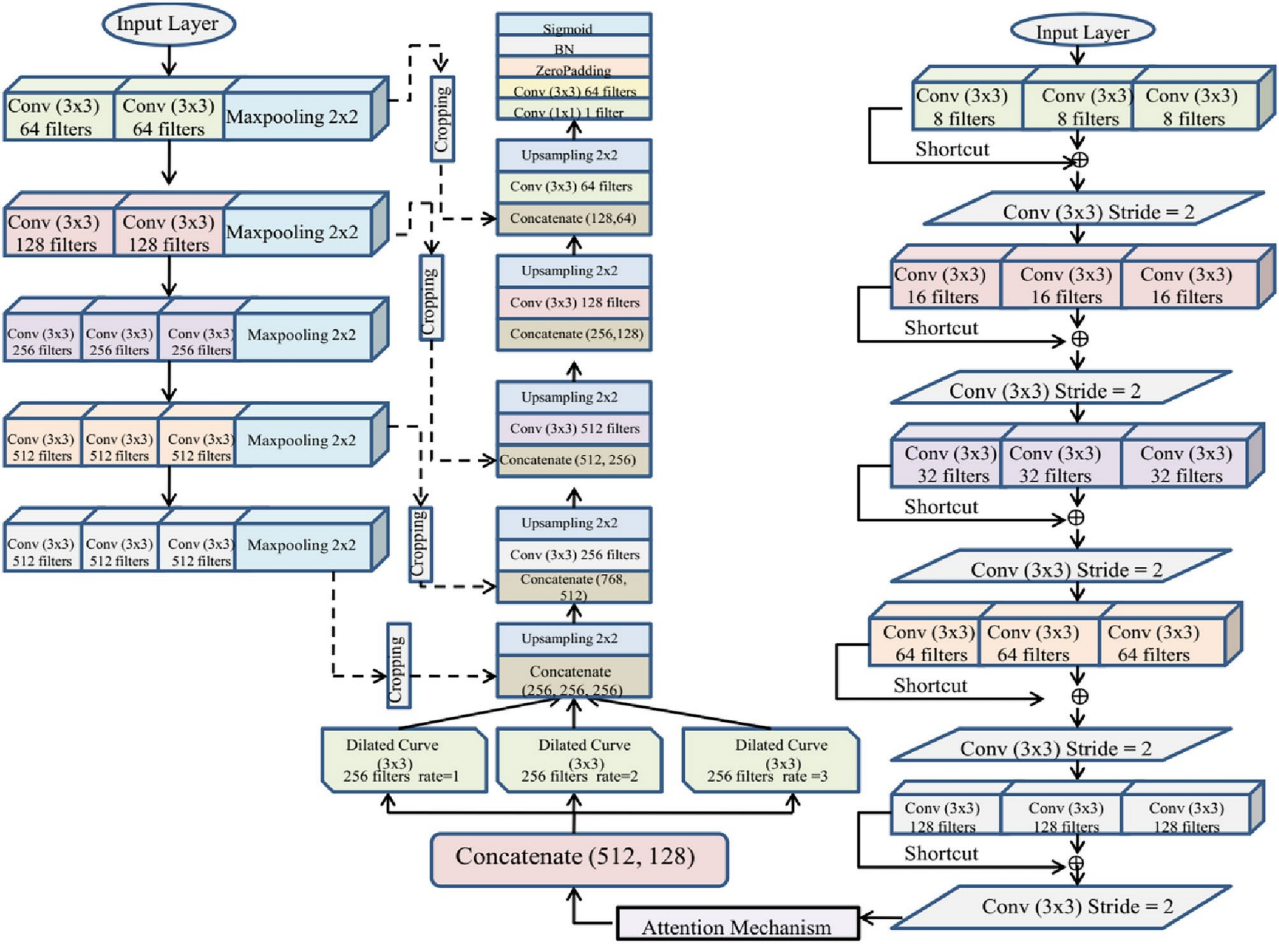
Proposed ADCU-Net model.

*Attention-based dual channel u-shaped network (ADCU-Net) Model*: Continuous convolution and deconvolution operations are used by maximal deep learning models for image tampering localization, which leads to continuous data loss in the feature extraction process. Compared to the conventional model, a crucial enhancement of the U-Net model is the integration of an intermediate skip connection framework,

Hence, a model same as the U-Net framework is utilized for detection. There are two encoders in the proposed method as mentioned in Fig. 2. A max-pooling layer and two convolution layers are comprised in the first and second convolution layers. The third, fourth and fifth convolution layer also consists of three convolution layers and a max-pooling layer. Thus, the findings evaluated from the stacked convolution layers are nearer to $I\left( y \right)$. Hence, the below equation derives the formulation of ordinary convolution block.

29 $$x = G\left( {y,\,v} \right)$$

From the above equation, the mapping function is represented by $G\,\left( . \right)$ , the input and output of the convolution block is represented by $y$ and $x$ respectively and the learning weight is represented by $v$. Let, $y$ represents the input of the residual module where $I\,\left( y \right)$ represents the expected output. When the input is fed into output directly, then $I\,\left( y \right) = G\,\left( y \right) + y$. Hence, only the residual function, $G\,\left( y \right) = I\,\left( y \right) - y$ is learned by the module without dealing the $I\,\left( y \right)$ straightforward. Thus, the below equation derives the definition of residual function.

30 $$x = G\left( {y,\,v} \right) + y$$

From the above equation, the residual mapping learned from the residual block is represented by $G\,\left( . \right)$.

The depth feature data is extracted by layer-wise convolution of two channels as illustrated in Fig. 2. The proposed approach completely relies on diverse dilated rates in expanding the filter view, thereby different sizes are detected effectively.

31 $$x\left[ j \right] = \sum\limits_{l} y \left[ {j + sl} \right]\,v\,\left[ l \right]$$

From the above equation, the filters and input feature maps are represented by $y$ and $v$ respectively. The sampling step of the input signal is evaluated by the dilated rate,$s$. It is similar to the convolution of input $y$ with respect to the up-sampling filter. The $s - 1$ zeros between two consecutive filter values are inserted to generate the up-sampling filter in spatial dimension. The receptive field is enlarged by the dilated convolution without introducing additional parameters. Initially, the rate of dilation is considered as 1, 2 and 3 in the dilated convolution for expanding the receptive field and so the feature data of diverse ranges are effectively extracted in the encoder module. Then the fusion of image semantic features extracted from various dilated rates is carried out. The proposed structure is a form of end-to-end deep learning system. Cross entropy loss function is the most common loss functions in deep learning structure. Here, the binary cross-entropy loss function is introduced to evaluate the loss rate in training. The below equation derives the binary cross entropy loss function.

32 $$BCE_{L} = \frac{1}{M}\sum\limits_{j}^{M} {h\left( y \right)} \log q\left( y \right) + \left( {1 - f\left( y \right)} \right)\log \left( {1 - q\left( y \right)} \right)$$

From the above equation,$BCE_{L}$ represents the binary cross-entropy loss function, the number of pixels is represented by $M$, the expected output is represented by $h\left( y \right)$, the actual data label denotes $h\left( . \right) \in \left[ {0,\,1} \right]$; where $q$ is the real output that denotes $q\left( . \right) \in \left[ {0,\,1} \right]$. The below equation derives the formula of dice loss function.

33 $$D_{L} = 1 - \frac{{2\sum\nolimits_{j}^{M} {q\left( y \right)h\left( y \right)} }}{{\sum\nolimits_{j}^{M} {q^{2} \left( y \right) + \sum\nolimits_{j}^{M} {h^{2} \left( y \right)} } }}$$

Thus, the final loss function is expressed in the below equation.

34 $$Loss_{L} = v * BCE_{L} + D_{L}$$

From the above equation, the weight of $BCE_{L}$ is represented by $v$. Thus the Adaptive Moment Estimation (Adam) with Nesterov momentum as the optimization algorithm is applied to enhance the prediction ability and speed up the training process. Adam has various merits such as adaptive adjustment of learning rate, high computational efficiency, and lower memory consumption than all other conventional approaches.

*Attention mechanism*: The Dual Channel U-shaped Network integrated with an attention mechanism is an innovative architecture designed to enhance leukocyte classification in medical images. By employing a dual-channel approach, the network processes different aspects of the data simultaneously, capturing diverse features related to color and texture. Its U-Net structure facilitates effective feature extraction through an encoder-decoder design, preserving crucial spatial information. The integrated attention mechanism improves the distinction between various leukocyte types while reducing the impact of noise. This combined approach results in superior classification accuracy and reliability, making it a valuable tool for clinical diagnostics in identifying conditions such as infections and blood disorders.

The attention mechanism helps distinguish the purpose of all features in seizure prediction, such as time-sensitive GRU and extraction of temporal features from CNN. In machine translation, persistent dependencies are improved by implementing the attention mechanism. Multiplicative attention, also referred to as Luang attention, is used in this chapter for useful evaluation of alignment function $c_{w}$ and it is expressed in the below equation.

35 $$c_{w} = \frac{{\exp \left( {j_{w}^{W} j_{t} } \right)}}{{\sum\nolimits_{w} {\exp \le \left( {j_{w}^{W} j_{t} } \right)} }}$$

From the above equation, key and query are represented by $j_{t}$ and $j_{w}$ respectively. The key and query are configured as the output state of the final GRU layer. In addition, the perspective of feature sequence $j_{w}$ is considered in the new feature set $Z_{c}$ and it is represented by using the attentive alignment score $c_{w}$ as shown in the below equation.

36 $$Z_{c} = \sum\limits_{w} {c_{w} j_{w} }$$

Then, the Fully Connected (FC) layer with the softmax function is employed to achieve the effective classification. The below expression is used to derive the probability of the classifier $C_{\alpha }$.

37 $$C_{\alpha } = soft\max \left( {XZ_{c} + d} \right)$$

From the above equation, the bias and weight are denoted by $d$ and $X$ respectively. The cross-entropy loss between the ground-truth label and the predicted probability is minimized for model training. The below equation derives the binary cross-entropy loss function.

38 $$L = - \sum\limits_{w} {\left( {X_{w} \log \left( q \right) + \left( {1 - Z_{w} } \right)\log \left( {1 - Z_{w} } \right)} \right)}$$

From the above equation, $L$ is represents the loss. The predicted probability and the target label are represented by $q_{w}$ and $Z_{w}$ at time $\left( w \right)$ respectively.

#### Integration of ADCU-Net into clinical workflows

The ADCU-Net model has great potential to enhance clinical diagnostics by automating leukocyte classification and segmentation. Its implementation within clinical workflows can be outlined as follows:

1. *Preprocessing stage*: Clinical samples often vary in quality due to differences in preparation methods, lighting, and imaging equipment. ADCU-Net includes preprocessing techniques such as noise reduction, contrast enhancement, and sharpening to standardize image quality before analysis. This ensures that input images are consistent and suitable for automated processing, improving reliability in diagnostic results.
2. *Segmentation and feature extraction*: By utilizing the Dung Beetle Optimization (DBO) algorithm, ADCU-Net segments relevant areas of the cell images. Extracted features, including statistical measures like entropy and homogeneity, offer deeper insights into cell characteristics. These extracted features enable the detection of abnormalities crucial for identifying conditions such as infections or blood disorders.
3. *Automated classification*: ADCU-Net categorizes leukocytes into groups like neutrophils, lymphocytes, monocytes, and basophils with high precision. This classification process is fully automated. It reduces manual workload, minimizes human error, and accelerates diagnostic workflows, allowing medical professionals to focus on complex cases.

#### Challenges in clinical implementation

While ADCU-Net has significant potential, its adoption into clinical practice comes with several challenges:

1. *Data variability*: Clinical datasets can vary widely due to differences in staining techniques, imaging quality, and patient demographics. This variability could affect the model’s performance. The Solution is to Enhance model training by including diverse datasets or employing domain adaptation techniques to generalize across different data types.
2. *Speed of analysis*: Real-time performance is crucial in clinical environments. However, the model’s computational demands may limit its speed, particularly when processing large datasets. The Solution is to Optimize the network by applying model compression methods like pruning or quantization to improve efficiency.
3. *Regulatory compliance and validation*
  The model must be rigorously validated and meet regulatory standards before it can be deployed in clinical settings.
  *Solution*: Collaborate with clinical institutions to conduct comprehensive trials and obtain certifications to ensure reliability.
4. *Transparency and Interpretability*
  For clinicians to adopt and trust the model, its decisions must be interpretable.
  *Solution*: Use interpretability tools such as attention maps and feature importance analysis to provide insights into the model’s predictions.
5. *Integration with Clinical Systems*
  Seamless integration with existing healthcare systems, such as laboratory information systems (LIS) and electronic medical records (EMR), is essential for widespread adoption.
  *Solution*: Develop user-friendly interfaces and APIs for smooth integration into clinical workflows.

By addressing these challenges, ADCU-Net can evolve into a practical diagnostic tool for clinical use. Future research should focus on these aspects to ensure that the model is both effective and applicable in real-world medical settings, ultimately enhancing diagnostic accuracy and efficiency.

## Results and discussions

The experimental evaluation presented in this section emphasizes the capability of the proposed approach to analyze its performance by using standard performance metrics on three different datasets.

### Experimental setup

The experiments were carried out on a Dell Optiplex 5050 computer, which has 16 GB of RAM as well as a 500 GB hard drive, and Intel Core i5 7th generation processor. By utilizing Python, all the models were created.

### Parameter settings

Table 1 lists the parameters and their corresponding values used in this experiment.

**Table 1:** ADCU-Net hyperparameter configuration.

Parameters	Values
Learning rate	0.001
Dropout rate	0.4
Epochs	100
Batch size	32
Pooling layer	Max pooling of size 2 × 2
Hidden layer	ReLu

### Evaluation indicators

The overall performance of the proposed approach is evaluated using diverse performance metrics such as accuracy, precision, recall, f1-score, Hausdorff distance, Jaccard Index score (JI score) and Dice Coefficient Score (DSC). The definition and mathematical formulae for each respective metric are expressed in the following sub-section.

*Accuracy*: Accuracy is one of the major performance metrics in which the overall capability of a model is effectively determined in terms of Leukocyte classification. Thus, the mathematical expression for accuracy is expressed in the below equation.

39 $$Accuracy = \frac{t\alpha }{{t\alpha + \,t\beta + \,\,f\alpha + \,f\beta }}$$

*Precision*: Precision is an important measure in which the performance of the proposed approach is obtained by evaluating the performance of positive predictions. Thus, the precision in classifying the Leukocyte is derived by using the below equation.

40 $$Precision = \frac{{t\alpha }}{{t\alpha + {\mkern 1mu} f\alpha }}$$

*Recall*: The ability of a model to find all matching events within a dataset is measured by recall. It is defined as the ratio of true positives to the sum of total number of true positives and false negatives. The recall is expressed by using the below equation.

41 $$Recall = \frac{{t\alpha }}{{t\alpha + {\mkern 1mu} f\beta }}$$

*F-score*: F1-score is a crucial metric that integrates the precision and recall into a single measure. The mathematical expression to assess the F1-score value is derived below. Thus, the trade-off between precision and recall is balanced.

42 $$F1-score = \frac{2 \times t\alpha }{{\left( {\left( {2 \times t\alpha } \right) + \,f\alpha + \,f\beta } \right)}}$$

From the above equations, the True Positives, True Negatives, False Positives, and False Negatives are represented by $t\alpha$, $t\beta$, $f\alpha$ and $f\beta$ respectively.

*Hausdorff distance*: It estimates the highest distance among the boundary points of two groups. It is the measurement of similarity degree among actual boundaries and predicted boundaries. The Hausdorff distance is derived by utilizing the below equation.

43 $$\theta_{Hausforff} = \max \left( {\mathop {\max }\limits_{{x \in \theta_{Xi} }} \mathop {\min }\limits_{{H \in \theta_{hi} }} \,D\left( {\theta_{Xt} ,\theta_{ht} } \right),\mathop {\max }\limits_{{H \in \theta_{hi} }} \mathop {\min }\limits_{{x \in \theta_{Wi} }} D\left( {\theta_{hi} ,\theta_{Xi} } \right)} \right)$$

*Jaccard Index score (JI Score)*: In the classification of leukocytes, the similarities between two sets comprising predicted and actual diagnoses are measured by the JI score. It is evaluated as the cross-ratio of sets for their union, with higher scores indicating effective agreement.

44 $$\theta_{JI\,score} = \frac{{\left| {\theta_{Xi} \cap \theta_{hi} } \right|}}{{\left| {\theta_{Xi} \cup \theta_{hi} } \right|}}$$

*Dice coefficient score (DSC)*: In the classification of leukocytes, the Dice coefficient is a quantitative indicator applied to estimate the similarity between two sets, including clinical diagnoses and diagnostic test outcomes. It measures the overlap between predicted and actual events, with higher values indicating better agreement.

45 $$\theta_{dcs} = \frac{{2\left| {\theta_{Xi} \cap \theta_{hi} } \right|}}{{\left| {\theta_{Xi} } \right| + \left| {\theta_{hi} } \right|}}$$

From the above equations, the ground truth and segmented images are represented by $\theta_{hi}$ and $\theta_{Xi}$ respectively.

### Performance analysis

**Fig. 3:**
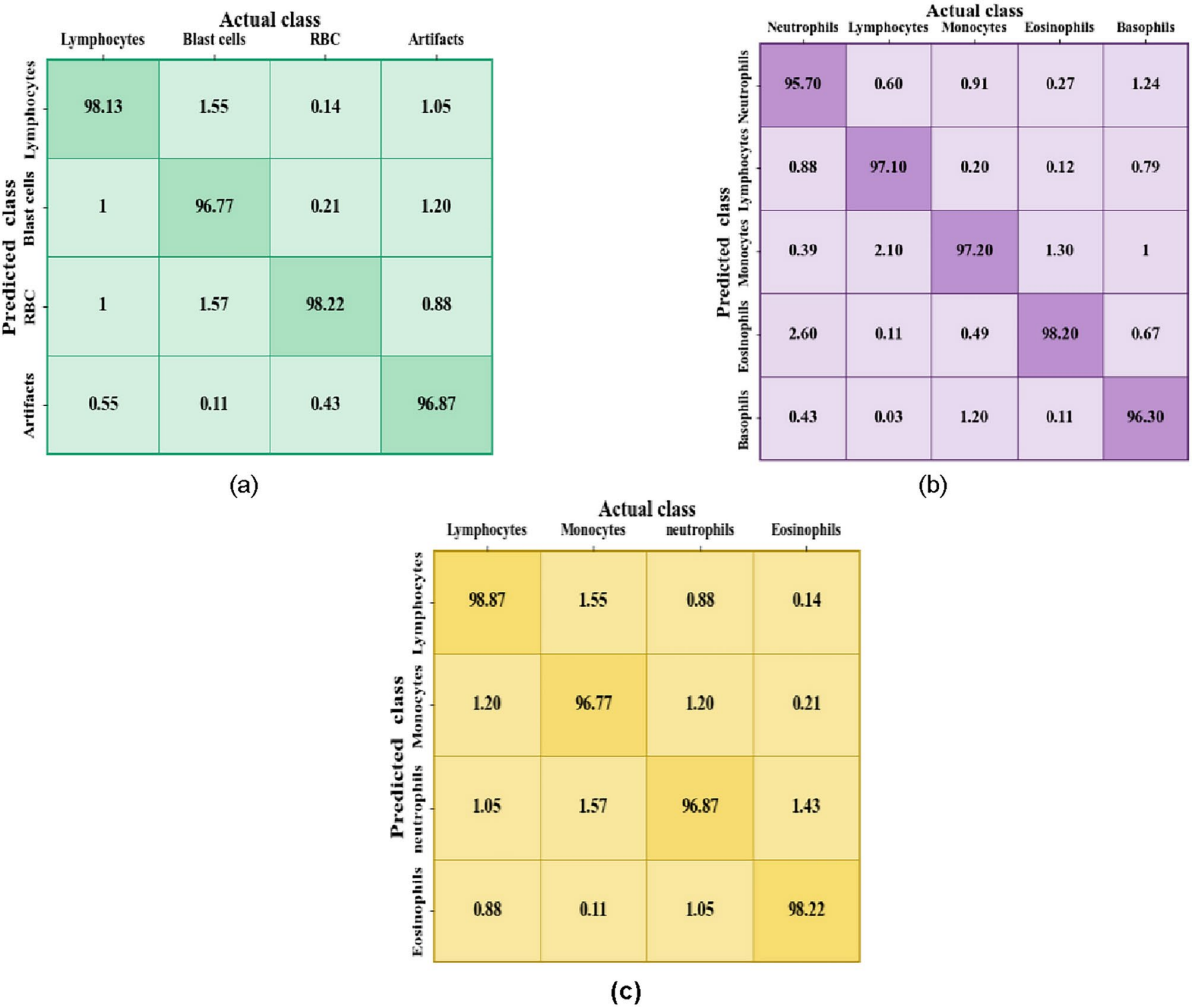
Confusion matrices for (**a**) ALL-IDB dataset (**b**) Cell morphological dataset of leukocytes (**c**) WBC-3 K-image dataset.

The effectiveness of a classification model is assessed and evaluated by the confusion matrix and it is achieved by the comparison of both the predicted and actual classes. It exhibits the total number of true positives, false positives, true negatives, and false negatives, highlighting the accuracy, precision, recall, and other metrics of a model. Thus, below Fig. 3 illustrates the confusion matrices for (a) the ALL-IDB Dataset, (b) Cell Morphological Dataset of Leukocytes and (c) WBC-3 K-Image Dataset.

**Fig. 4:**
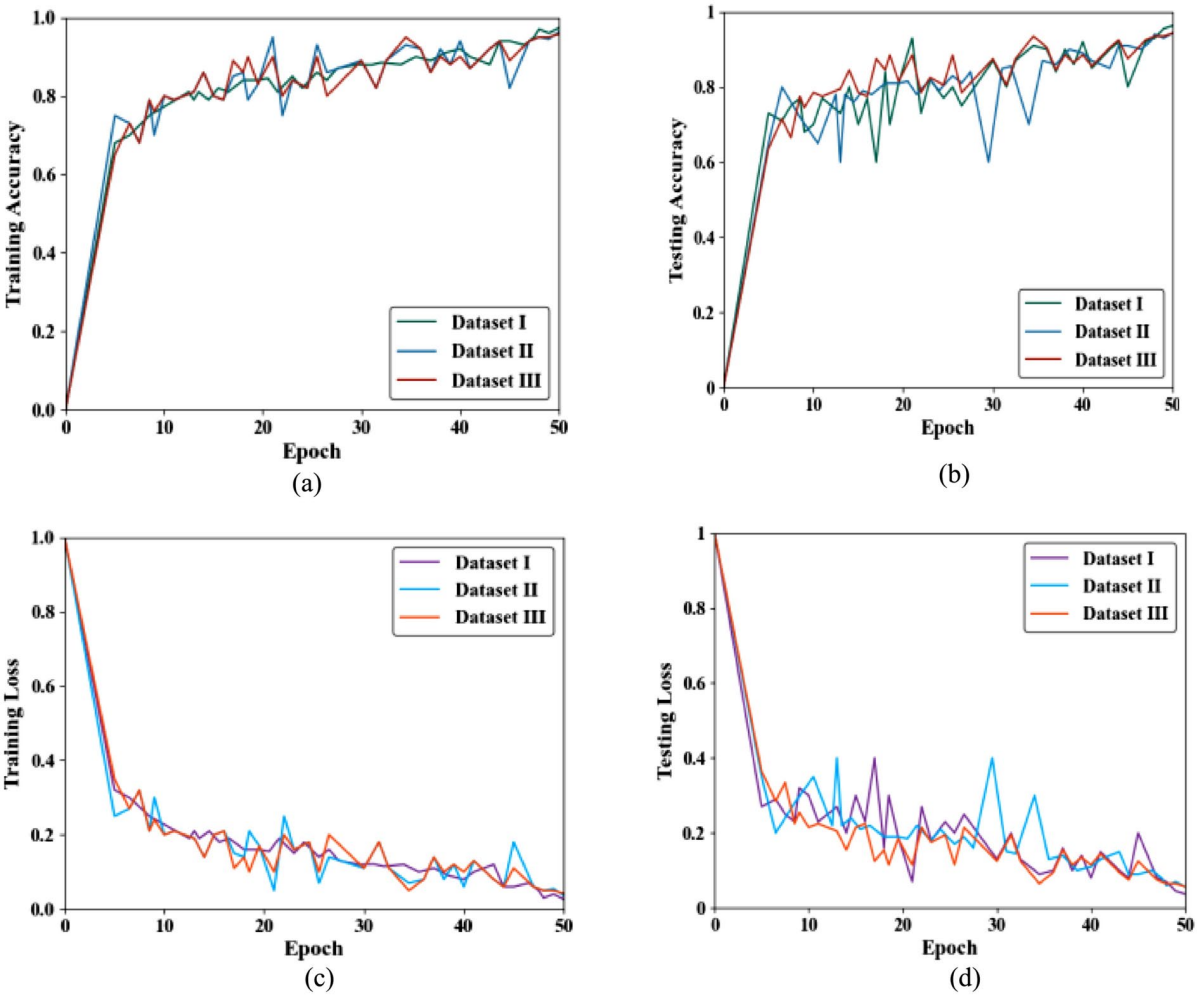
Performance analysis for (**a**) training accuracy and (**b**) testing accuracy (**c**) training loss and (**d**) testing loss for diverse datasets.

Figure 4 illustrates the rate of training accuracy, testing accuracy, training loss and testing loss of all three datasets including the ALL-IDB Dataset, Cell Morphological Dataset of Leukocytes, and WBC-3 K-Image Dataset. Figure 4a is plotted between the number of epochs and the training accuracy rate. From the graph, it is noted that the ALL-IDB Dataset has the highest training accuracy at 97.4%, followed by Cell Morphological Dataset of Leukocytes at 96.3%, and WBC-3 K-Image Dataset at 87.9%. Figure 4b is plotted between the number of epochs and the testing accuracy rate. The generalization gap is expressed in lower test accuracies, where the performance of the model is not at the level of the unseen data. ALL-IDB Dataset still exhibits a high test accuracy of 94.3%, while WBC-3 K-Image Dataset shows the biggest drop, with a test accuracy of 85.9%. Figure 4c is plotted between the number of epochs and training loss. From this graph, it is revealed that the ALL-IDB Dataset achieves the lowest loss at 0.0270, signifying an effective model fit during training. On the other hand, the WBC-3 K-Image Dataset shows the highest training loss at 0.121, exhibiting the model faces more difficulties with this dataset in the training. Figure 4d is plotted between the number of epochs and testing loss. The test loss values are greater than the training loss values on all datasets, exhibiting a gradual drop in model performance on unseen data. Thus, this graph shows that the ALL-IDB Dataset again has the lowest testing loss at 0.057, denoting robust generalization, while WBC-3 K-Image Dataset reveals the highest testing loss at 0.141, representing different complexities in generalizing to new data.

**Fig. 5:**
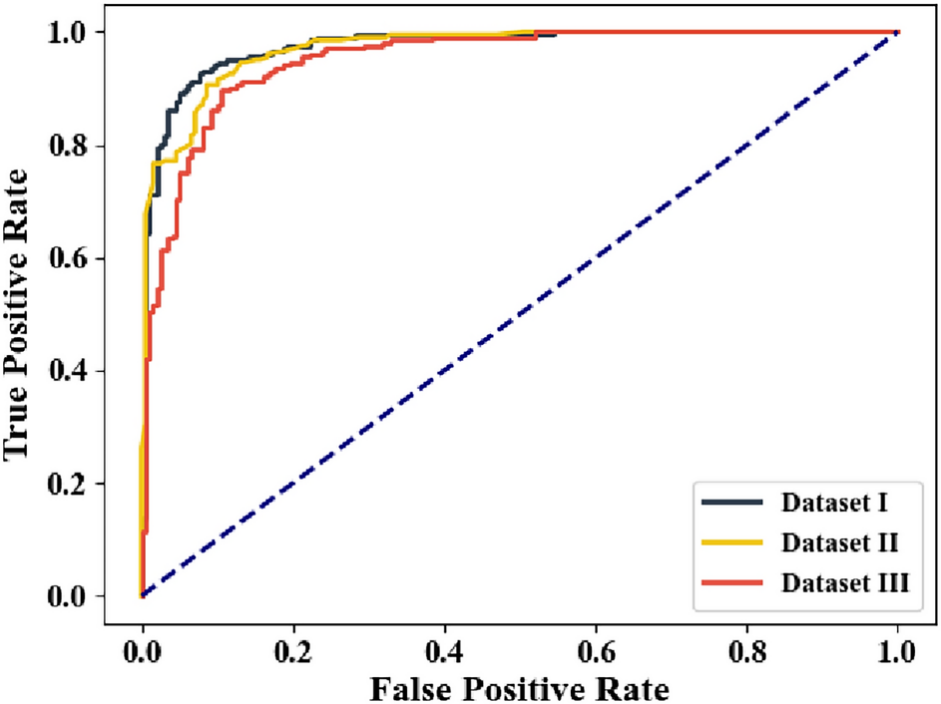
AUCROC analysis of different datasets.

Figure 5 illustrates the AUCROC analysis of all three datasets including the ALL-IDB Dataset, Cell Morphological Dataset of Leukocytes, and WBC-3 K-Image Dataset. Here, the graph is plotted between the false positive rate and the true positive rate. From the graph, it is exhibited that the ALL-IDB Dataset attains a higher AUCROC value of 0.96. Thus, the ALL-IDB Dataset performs better than all other datasets.

Table 2 describes an extensive summary of the proposed model’s efficiency over diverse datasets including the ALL-IDB Dataset, Cell Morphological Dataset of Leukocytes, and WBC-3 K-Image Dataset. Thus, the ADCU-Net model shows excellent performance concerning accuracy, precision, recall, f1-score, Hausdorff distance, JI score and DSC for all the three datasets implemented in this work. In addition, the proposed model attains significantly low Hausdorff distances of 11.2 mm, 9.87 mm, and 13.43 mm on ALL-IDB Dataset, Cell Morphological Dataset of Leukocytes, and WBC-3 K-Image Dataset respectively. These results highlight the effectiveness of the proposed model in accurately classifying the leukocyte in all three datasets. The mean values of each measure are evaluated to determine an overall assessment of the model’s performance.

**Table 2:** Performance evaluation of the proposed model on different datasets.

Metrics	Performance rate (Mean ± SD)		
	ALL-IDB dataset	Cell morphological dataset of leukocytes	WBC-3 K-image dataset
Accuracy	0.9812 ± 0.0032	0.9846 ± 0.0537	0.9790 ± 0.0638
Precision	0.9792 ± 0.0032	0.9812 ± 0.0009	0.9638 ± 0.0378
Recall	0.9697 ± 0.0432	0.9923 ± 0.0389	0.9832 ± 0.0087
F1-score	0.9791 ± 0.0043	0.9823 ± 0.0003	0.9643 ± 0.0078
DSC	0.8875 ± 0.0892	0.9570 ± 0.0003	0.8901 ± 0.0983
JI Score	0.9125 ± 0.0098	0.9479 ± 0.0387	0.8959 ± 0.0375
Hausdorff (mm)	11.2	9.87	13.43

### Comparative analysis

**Fig. 6:**
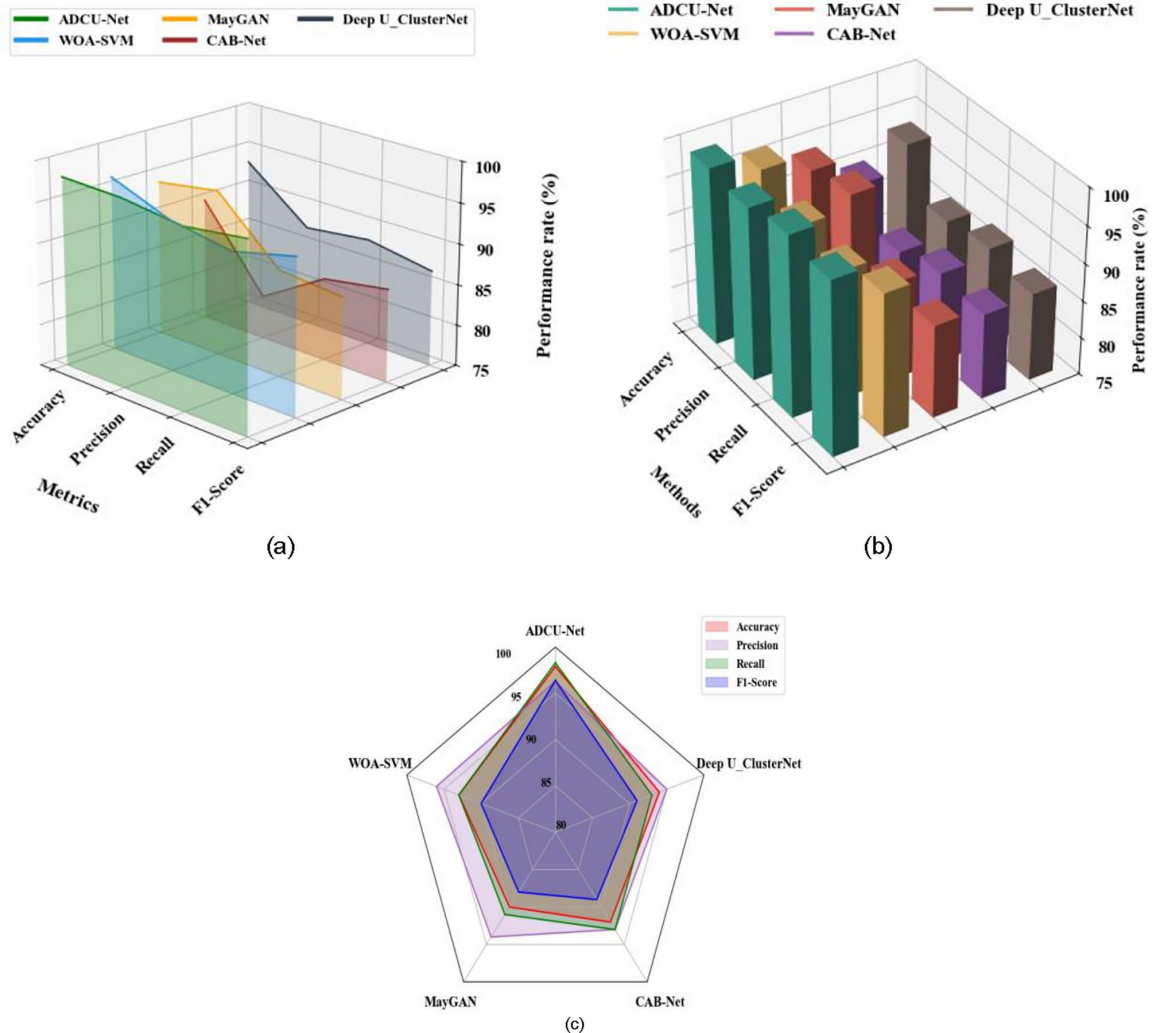
Comparative analysis of different approaches based on (**a**) ALL-IDB dataset (**b**) Cell morphological dataset of leukocytes and (**c**) WBC-3 K-image dataset.

Figure 6a–c shows the comparative graphical representation of the proposed ADCU-Net algorithm and various other approaches such as WOA-SVM , MayGAN, CAB-Net , Deep U_ClusterNet for different evaluation indicators based on different datasets including the ALL-IDB Dataset, Cell Morphological Dataset of Leukocytes, and WBC-3 K-Image Dataset. Figure 6a depicts the graphical representation to determine the performance rate for diverse approaches based on ALL-IDB dataset. From the graph, it is noted that the accuracy, precision, recall, and F1-score of the proposed ADCU-Net approach are 98.1, 97.9, 96.9, and 97.9% respectively. Therefore, the proposed ADCU-Net approach is better than that of all other existing approaches based on ALL-IDB Dataset. Figure 6b depicts the graphical representation to determine the performance rate for various approaches based on Cell Morphological Dataset of Leukocytes. From the graph, it is noted that the accuracy, precision, recall, and F1-score of the proposed ADCU-Net approach are 98.4, 98.1, 99.2, and 98.2% respectively. Therefore, the proposed ADCU-Net approach outperformed all other existing approaches based on Cell Morphological Dataset of Leukocytes. Figure 6c illustrates the graphical representation to determine the performance rate for diverse approaches based on WBC-3 K-Image Dataset. From the graph, it is noted that the accuracy, precision, recall, and F1-score of the proposed ADCU-Net approach are 97.9, 96.3, 98.3, and 96.4% respectively. Therefore, the proposed WEGASCN approach is superior all other existing approaches based on NewHandPD dataset.

**Fig. 7:**
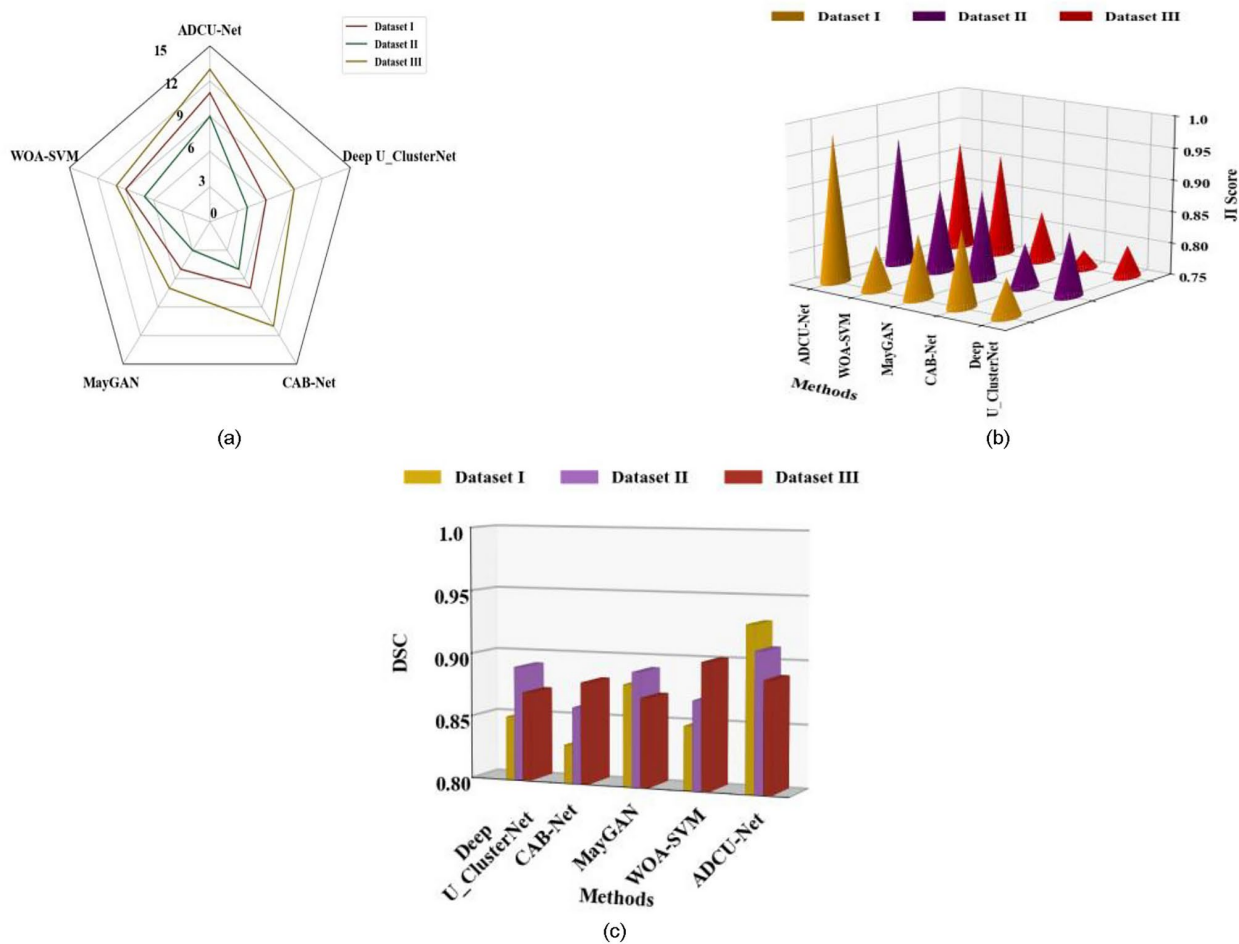
Comparative analysis of different approaches based on (**a**) Hausdorff distance (**b**) JI score and (c) DSC.

This section depicts the comparative graphical representation of the proposed ADCU-Net approach with different approaches such as WOA-SVM, MayGAN, CAB-Net, Deep U_ClusterNetin terms of Hausdorff distance, JI score and DCS. In Fig. 7a–c, the comparison of Hausdorff distance, JI score and DCS of different approaches including the proposed ADCU-Net approach is performed. Figure 7a depicts the graphical representation to determine the Hausdorff distance for various approaches based on all the three datasets namely ALL-IDB Dataset, Cell Morphological Dataset of Leukocytes, and WBC-3 K-Image Dataset. From the graph it is noted that the proposed ADCU-Net approach outperformed all other approaches such as WOA-SVM, MayGAN, CAB-Net, Deep U_ClusterNet in terms of Hausdorff distance. Figure 7b illustrates the graphical representation to determine the JI score for various techniques based on all the three datasets. Thus, the comparative analysis exhibited that the proposed ADCU-Net approach attains higher JI score compared to all other approaches. Figure 7c depicts the graphical representation to determine the DSC for various techniques based on all the three datasets. The comparative results revealed that the proposed ADCU-Net approach attains higher value compared to all other existing approaches.

The proposed Attention-based Dual Channel U-shaped Network (ADCU-Net) achieved an accuracy of 98.4% in leukocyte classification, demonstrating its high effectiveness.

1. *Evaluation of achieved accuracy*
  *Significance of 98.4% accuracy*:
    The achieved accuracy of 98.4% indicates the model’s ability to correctly classify leukocytes with minimal errors. This high level of accuracy reflects the model’s robust architecture, including attention mechanisms and dual-channel pathways that effectively capture spatial and contextual features.
  *Factors contributing to high accuracy*:
    Preprocessing techniques (noise reduction, contrast enhancement, and background removal) improve image clarity.
    Advanced segmentation using the Dung Beetle Optimization (DBO) algorithm with Levy flight optimization enhances boundary detection, ensuring better feature extraction.
    The novel ADCU-Net architecture enables the model to focus on relevant features while preserving spatial details.
2. *Comparison with state-of-the-art methods*
  The proposed model’s performance can be benchmarked against existing methods for leukocyte classification to highlight its advantages.
  *Conventional machine learning approaches*:
    Traditional models using handcrafted features and classifiers like Support Vector Machines (SVM) or k-Nearest Neighbors (k-NN) typically achieve accuracies in the range of 80–90%. These methods are limited by their reliance on manually extracted features and sensitivity to noise.
  *Deep learning models*:
    CNN-based models often achieve accuracies between 90 and 96% but may struggle with overfitting or losing spatial details during feature extraction.
    U-Net-based architectures are widely used for segmentation tasks, with some achieving accuracies close to 97%, but they may lack the precision in capturing finer details without attention mechanisms.
  *Performance of ADCU-Net*:
    The ADCU-Net surpasses most existing methods by leveraging dual-channel inputs and attention modules. These innovations enhance feature representation and focus on critical regions, achieving a higher classification accuracy of 98.4%.
3. Comparative Insights
  *Advantages over traditional methods*:
    The ADCU-Net’s use of advanced preprocessing and segmentation significantly improves image quality and boundary detection, areas where traditional methods often falter.
  *Edge over deep learning models*:
    The inclusion of attention mechanisms and dual channels allows the ADCU-Net to outperform standard CNN and U-Net architectures by preserving spatial and contextual details, which are critical for accurate leukocyte classification.

The ADCU-Net model demonstrates significant potential for scalability to larger and more diverse datasets, owing to its robust architecture and advanced feature extraction capabilities. Below is an analysis of its adaptability and performance in handling varying image qualities and different leukocyte types.

1. *Scalability to larger datasets*
  *Efficient preprocessing*:
    The model incorporates preprocessing techniques such as noise reduction, contrast enhancement, and background removal, which enhance the quality of input images. These steps are computationally efficient, allowing the model to scale effectively when handling larger datasets.
  *Robust segmentation*:
    The integration of the Dung Beetle Optimization (DBO) algorithm with Levy flight optimization ensures precise segmentation across a wide range of images. This segmentation robustness makes the model scalable to datasets with varied imaging conditions and complexities.
  *Training efficiency*:
    The attention mechanisms embedded in the ADCU-Net architecture optimize resource utilization during training, reducing computational overhead while maintaining high performance. This efficiency facilitates the processing of extensive datasets without significant degradation in speed or accuracy.
2. *Adaptability to varying image qualities*
  *Noise tolerance*:
    The preprocessing pipeline effectively addresses variations in image quality, such as noise or poor contrast, ensuring consistent feature extraction. This adaptability is particularly beneficial for datasets with non-uniform imaging standards.
  *Generalization across modalities*:
    The dual-channel architecture of ADCU-Net is designed to preserve both spatial and contextual information, enabling it to perform well across diverse image modalities and resolutions.
3. *Classification of diverse leukocyte types*
  *Feature sensitivity*:
    The attention mechanisms enhance the model’s ability to focus on relevant features, making it capable of distinguishing between different leukocyte types with subtle structural differences.
  *Robust feature extraction*:
    Metrics like standard deviation, mean, and entropy, used during feature extraction, capture both global and local characteristics of leukocytes. This ensures reliable classification across varying cell types and conditions.
4. *Challenges and mitigation*
  While the ADCU-Net model shows strong scalability and adaptability, potential challenges include:
  *High computational requirements*:
    Larger datasets may increase the demand for computational resources. This can be mitigated by leveraging distributed training and hardware acceleration (e.g., GPUs/TPUs).
  *Dataset diversity*:
    Variations in imaging techniques and leukocyte types across datasets may introduce inconsistencies. Incorporating domain adaptation techniques or transfer learning can address these challenges.

### Ablation study

**Fig. 8:**
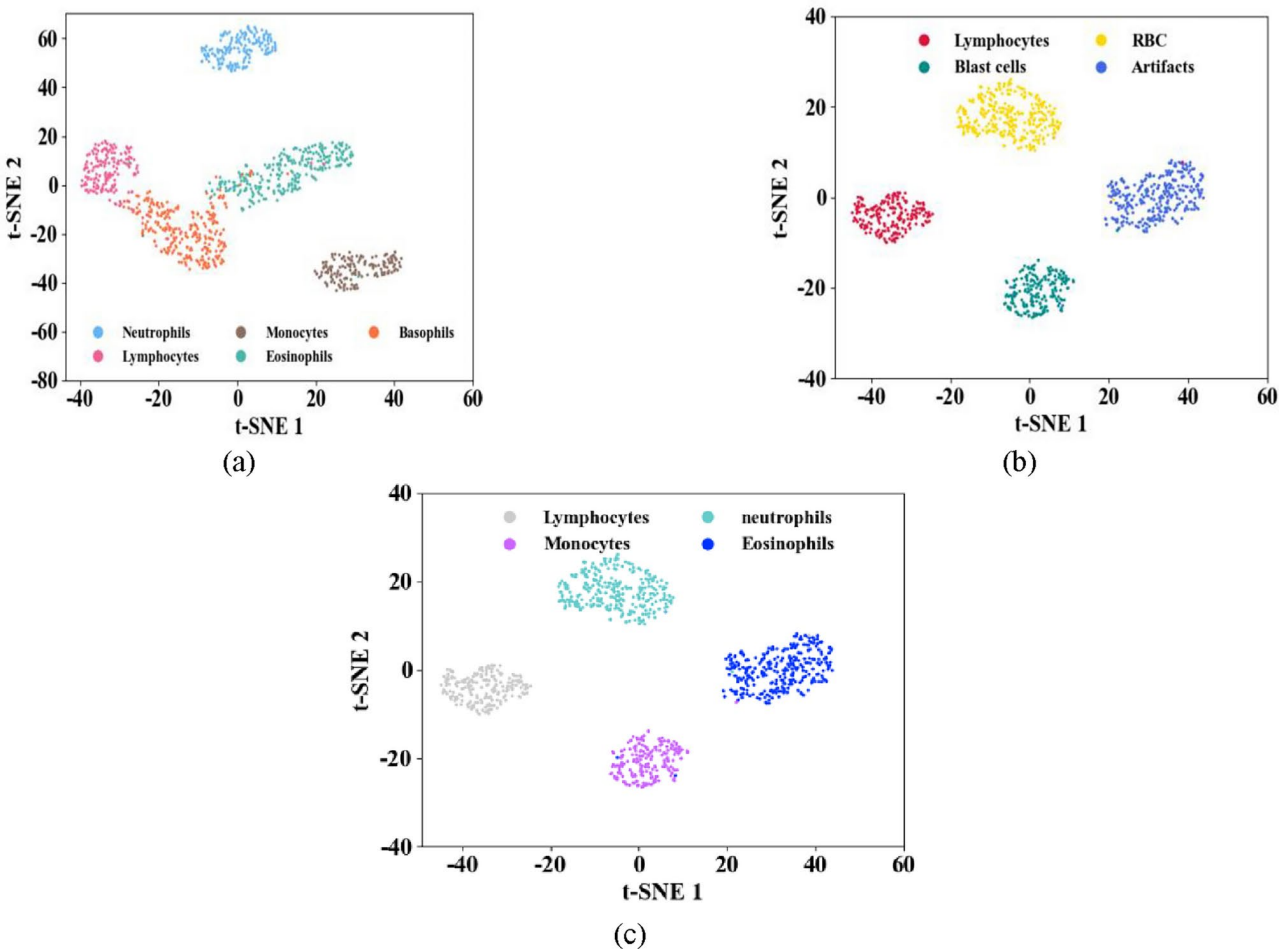
Scatter plots for (**a**) ALL-IDB dataset (**b**) Cell morphological dataset of leukocytes and (**c**) WBC-3 K-image dataset.

Figure 8a–c shows four various scatter plots, each related to different datasets, including the ALL-IDB Dataset, Cell Morphological Dataset of Leukocytes, and WBC-3 K-Image Dataset. These plots use t-distributed stochastic neighbor embedding (t-SNE) to map high-dimensional information into a two-dimensional space. The X-axis of the graph contains t-SNE 1 and the Y-axis contains t-SNE 2. The colour of the data point indicates the different classifications or classes within each dataset. These visual representations suggest different levels of clustering and class differentiation between datasets, as well as prompt investigation of factors contributing to variations in underlying data dimensionality, cluster characteristics, and class isolation. A deeper insight into the original information and characteristics used for t-SNE may generate further insights into these observed patterns and their significance.

### Statistical analysis

Table 3 provides a detailed evaluation using the Friedman rank test to evaluate the performance of the proposed model in comparison to existing approaches. This test is utilized to determine whether there are statistically important variations between the set average of different metrics, like accuracy, precision, F1-score, Dice Coefficient Score (DSC), Jaccard Index score (JI score). In the context of this evaluation, each model is assigned a rank according to its performance on these metrics. The proposed approach achieves a rank of 1, which indicates its robustness across existing approaches and establishes it as a leading approach in terms of performance according to the results of the experiment. This ranking highlights the high performance of the model relative to the other approaches evaluated.

**Table 3:** Friedman ranking analysis.

Methods	Accuracy	Precision	Recall	F1-score	JI score	DSC	Average rank
ADCU-Net	1	1	1	1	1	1	1
WOA-SVM	4	3	5	3	4	2	3.6
MayGAN	5	2	4	5	2	7	4.1
CAB-Net	2	2	3	7	5	4	3.7
Deep U_ClusterNet	3	6	7	5	3	2	4.3

## Challenges in deploying the ADCU-Net model in clinical settings and potential solutions

Deploying the ADCU-Net model in clinical environments presents certain challenges related to computational demands, real-time processing, and integration with existing systems. Below, we highlight these challenges and propose solutions to address them.

1. *High computational demands*
  *Challenge*:
    The ADCU-Net model, with its advanced architecture and preprocessing steps, requires significant computational power for training and inference. This demand can be a barrier in clinical settings where resources may be limited.
  *Solutions*:
    *Hardware acceleration*: Utilizing GPUs, TPUs, or specialized hardware like edge AI devices can significantly reduce processing time.
  *Model optimization*: Techniques like model pruning, quantization, or knowledge distillation can reduce the size and complexity of the model while maintaining accuracy.
  *Cloud integration*: Deploying the model on cloud platforms enables access to scalable computational resources, reducing on-premise hardware dependency.
2. *Real-time processing*
  *Challenge*: In clinical workflows, real-time or near-real-time analysis is often required to make quick decisions. The preprocessing and segmentation steps of ADCU-Net may introduce delays, making real-time processing challenging.
  *Solution*: Pipeline Optimization: Streamlining the preprocessing and segmentation pipelines can help minimize delays.
    *Batch processing*: Instead of processing one image at a time, batch processing can optimize throughput for real-time requirements.
    *Edge computing*: Deploying the model on edge devices close to the data source reduces latency and ensures faster processing.
3. *Integration with Clinical Systems*
  *Challenge*:
    Integrating the model into existing clinical systems, such as electronic health records (EHRs) or diagnostic platforms, requires compatibility with diverse data formats and workflows.
  *Solutions*:
    Interoperability standards: Ensuring the model adheres to standards like HL7 or DICOM facilitates seamless integration.
    APIs and middleware: Developing APIs or middleware layers can bridge the gap between the model and clinical systems, enabling smooth communication and data exchange.
    User training: Training healthcare professionals on the use of the model ensures its effective deployment and acceptance in clinical workflows.
4. *Data privacy and security*
  *Challenge*: Handling sensitive patient data raises concerns about privacy and compliance with regulations such as HIPAA or GDPR.
  *Solution*:
    *Data encryption*: Encrypting data during transmission and storage ensures security.
    *Federated learning*: This approach allows training models on decentralized data without transferring it, preserving privacy.
    *Regular audits*: Conducting regular security audits ensures compliance with regulatory requirements.

## Conclusion

This research introduces the Attention-based Dual Channel U-shaped Network (ADCU-Net) for effective leukocyte classification, addressing challenges in conventional leukocyte analysis. The experimental results underscore the effectiveness of the proposed Attention-based Dual Channel U-shaped Network (ADCU-Net) for leukocyte classification across three diverse datasets. The model demonstrated exceptional performance, achieving accuracy rates of 98.4% on the Cell Morphological Dataset, 98.1% on the ALL-IDB Dataset, and 97.9% on the WBC-3 K-Image Dataset. The integration of advanced image preprocessing and optimization techniques significantly contributed to improved diagnostic precision, as evidenced by favorable metrics such as precision, recall, F1-score, and low Hausdorff distances. Additionally, comparative analyses confirmed ADCU-Net’s superiority over existing models, achieving the highest rankings in performance metrics. The results indicate that this model can serve as a powerful tool for enhancing diagnostic capabilities in clinical settings, paving the way for further research and application in automated leukocyte analysis. In the future, implementing unsupervised learning methods to automatically identify and extract relevant features could further streamline the classification process and reduce reliance on predefined parameters.

### Future improvements for enhanced segmentation and classification

Future improvements to the ADCU-Net model can focus on several key areas to enhance its performance in leukocyte segmentation and classification. Incorporating additional biomarkers, such as chemical compositions, fluorescence signals, or genetic markers, could provide deeper insights into leukocyte characteristics. By utilizing multimodal data that combines imaging and non-imaging biomarkers and integrating these into the feature extraction process, the model can capture a broader range of features, improving its ability to differentiate between leukocyte subtypes and detect subtle abnormalities. Exploring advanced optimization algorithms is another promising direction. While the Dung Beetle Optimization (DBO) algorithm with Levy flight optimization has been effective, alternatives like Particle Swarm Optimization (PSO), Genetic Algorithms (GA), or Reinforcement Learning (RL) could be tested. These methods may streamline segmentation processes, enhance search efficiency, and improve boundary detection accuracy. Refining the model architecture also offers significant potential for improvement. Adding multi-scale attention modules can focus on features at different resolutions, while residual connections can prevent gradient vanishing and retain critical information during feature extraction. Additionally, self-supervised learning can leverage unlabeled data to reduce dependence on large, labeled datasets, making the model more robust and adaptable. Finally, expanding dataset diversity by including images from various clinical setups, imaging modalities, and rare leukocyte types will enhance the model’s generalizability, reduce biases, and ensure it performs well across diverse real-world scenarios. These advancements will collectively improve the accuracy, scalability, and clinical applicability of the ADCU-Net model.

## Supplementary Information

Supplementary Information 1.

Supplementary Information 2.

Supplementary Information 3.

## Data Availability

Data is provided within the manuscript or supplementary information files. The link from which dataset can be accessed is provided in Section “Proposed methodology”, Subsection “Data collection”. However, the datasets generated and/or analysed during the current study are available in the [Kaggle] repository, [https://www.kaggle.com/datasets/sizlingdhairya1/all-idb-images], [https://www.kaggle.com/datasets/lsaa2014/single-cell-morphological-dataset-of-leukocytes/data] and [https://www.kaggle.com/datasets/quangnguynvnnn/wbc-3 k-image]. Also, all data generated or analysed during this study are included in this published article [and its supplementary information files].
