# Supplementary figures and images for: Advanced leukocyte classification using attention mechanisms and dual channel U-Net architecture (part 2 of 4)
Source: Sci Rep. 2025 Apr 22;15:13825. doi: 10.1038/s41598-025-96918-3 (PMC12015285; doi:10.1038/s41598-025-96918-3)

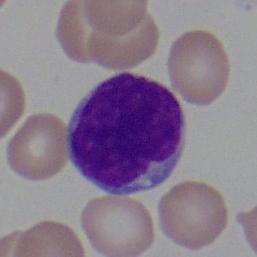

Supplement: Supplementary file 1 — Supplementary Information 1. [file 41598_2025_96918_MOESM1_ESM.zip › ALL_IDB Dataset/L3/Im003_30.jpg]

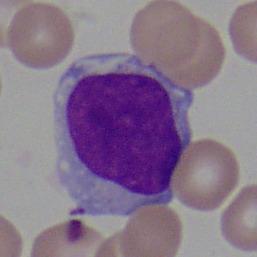

Supplement: Supplementary file 1 — Supplementary Information 1. [file 41598_2025_96918_MOESM1_ESM.zip › ALL_IDB Dataset/L3/Im003_31.jpg]

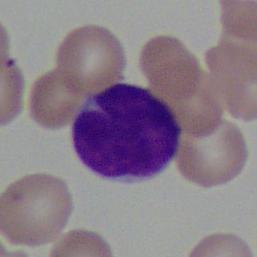

Supplement: Supplementary file 1 — Supplementary Information 1. [file 41598_2025_96918_MOESM1_ESM.zip › ALL_IDB Dataset/L3/Im003_32.jpg]

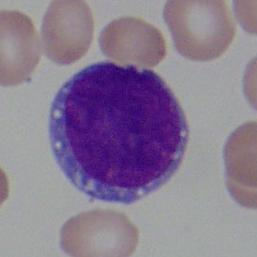

Supplement: Supplementary file 1 — Supplementary Information 1. [file 41598_2025_96918_MOESM1_ESM.zip › ALL_IDB Dataset/L3/Im003_33.jpg]

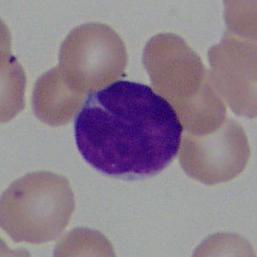

Supplement: Supplementary file 1 — Supplementary Information 1. [file 41598_2025_96918_MOESM1_ESM.zip › ALL_IDB Dataset/L3/Im003_34.jpg]

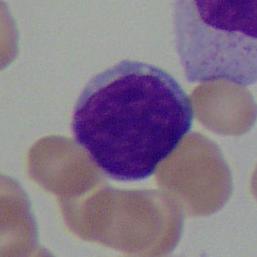

Supplement: Supplementary file 1 — Supplementary Information 1. [file 41598_2025_96918_MOESM1_ESM.zip › ALL_IDB Dataset/L3/Im003_35.jpg]

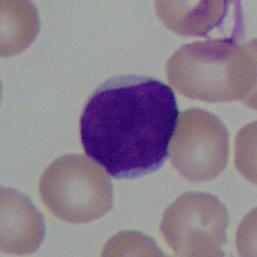

Supplement: Supplementary file 1 — Supplementary Information 1. [file 41598_2025_96918_MOESM1_ESM.zip › ALL_IDB Dataset/L3/Im003_36.jpg]

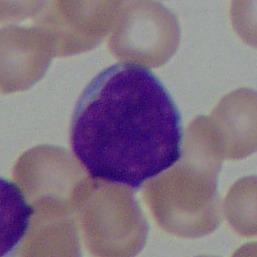

Supplement: Supplementary file 1 — Supplementary Information 1. [file 41598_2025_96918_MOESM1_ESM.zip › ALL_IDB Dataset/L3/Im003_37.jpg]

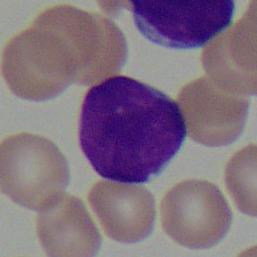

Supplement: Supplementary file 1 — Supplementary Information 1. [file 41598_2025_96918_MOESM1_ESM.zip › ALL_IDB Dataset/L3/Im003_38.jpg]

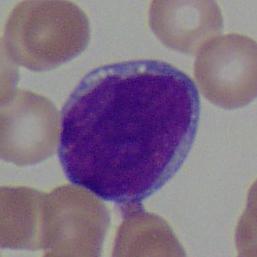

Supplement: Supplementary file 1 — Supplementary Information 1. [file 41598_2025_96918_MOESM1_ESM.zip › ALL_IDB Dataset/L3/Im003_39.jpg]

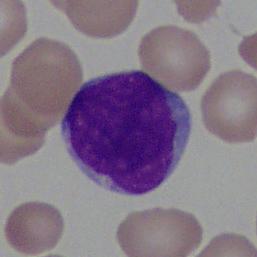

Supplement: Supplementary file 1 — Supplementary Information 1. [file 41598_2025_96918_MOESM1_ESM.zip › ALL_IDB Dataset/L3/Im003_40.jpg]

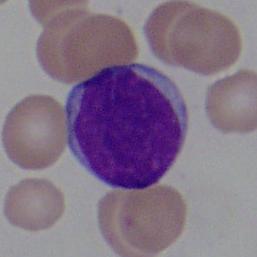

Supplement: Supplementary file 1 — Supplementary Information 1. [file 41598_2025_96918_MOESM1_ESM.zip › ALL_IDB Dataset/L3/Im003_41.jpg]

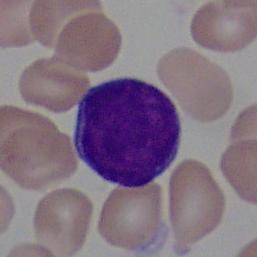

Supplement: Supplementary file 1 — Supplementary Information 1. [file 41598_2025_96918_MOESM1_ESM.zip › ALL_IDB Dataset/L3/Im003_42.jpg]

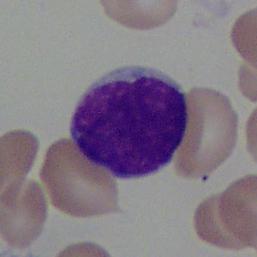

Supplement: Supplementary file 1 — Supplementary Information 1. [file 41598_2025_96918_MOESM1_ESM.zip › ALL_IDB Dataset/L3/Im003_43.jpg]

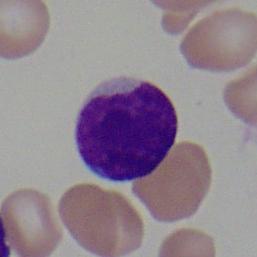

Supplement: Supplementary file 1 — Supplementary Information 1. [file 41598_2025_96918_MOESM1_ESM.zip › ALL_IDB Dataset/L3/Im003_44.jpg]

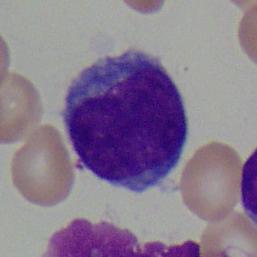

Supplement: Supplementary file 1 — Supplementary Information 1. [file 41598_2025_96918_MOESM1_ESM.zip › ALL_IDB Dataset/L3/Im003_45.jpg]

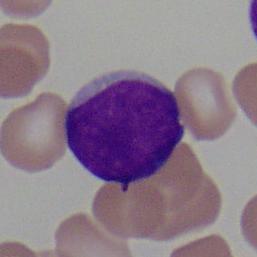

Supplement: Supplementary file 1 — Supplementary Information 1. [file 41598_2025_96918_MOESM1_ESM.zip › ALL_IDB Dataset/L3/Im003_46.jpg]

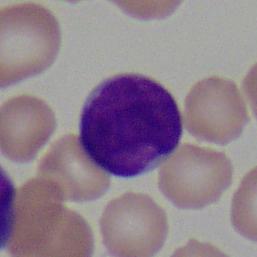

Supplement: Supplementary file 1 — Supplementary Information 1. [file 41598_2025_96918_MOESM1_ESM.zip › ALL_IDB Dataset/L3/Im003_47.jpg]

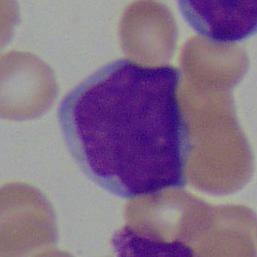

Supplement: Supplementary file 1 — Supplementary Information 1. [file 41598_2025_96918_MOESM1_ESM.zip › ALL_IDB Dataset/L3/Im003_48.jpg]

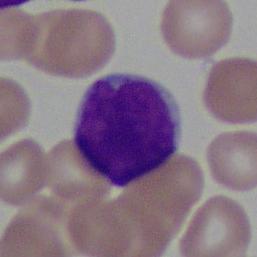

Supplement: Supplementary file 1 — Supplementary Information 1. [file 41598_2025_96918_MOESM1_ESM.zip › ALL_IDB Dataset/L3/Im003_49.jpg]

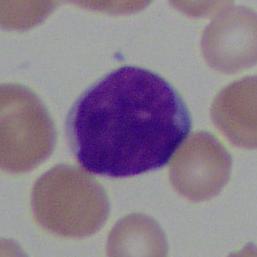

Supplement: Supplementary file 1 — Supplementary Information 1. [file 41598_2025_96918_MOESM1_ESM.zip › ALL_IDB Dataset/L3/Im003_50.jpg]

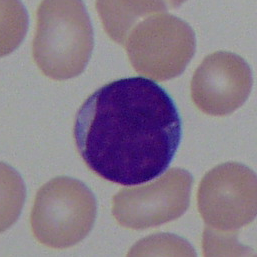

Supplement: Supplementary file 1 — Supplementary Information 1. [file 41598_2025_96918_MOESM1_ESM.zip › ALL_IDB Dataset/L3/Im004_1.tif]

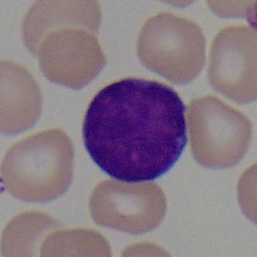

Supplement: Supplementary file 1 — Supplementary Information 1. [file 41598_2025_96918_MOESM1_ESM.zip › ALL_IDB Dataset/L3/Im005_1.tif]

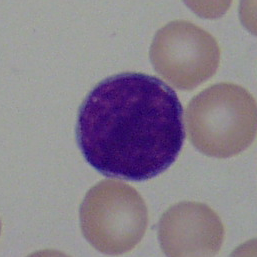

Supplement: Supplementary file 1 — Supplementary Information 1. [file 41598_2025_96918_MOESM1_ESM.zip › ALL_IDB Dataset/L3/Im006_1.tif]

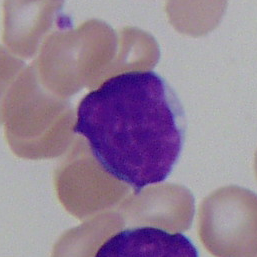

Supplement: Supplementary file 1 — Supplementary Information 1. [file 41598_2025_96918_MOESM1_ESM.zip › ALL_IDB Dataset/L3/Im008_1.tif]

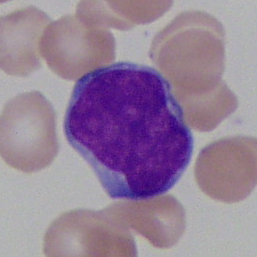

Supplement: Supplementary file 1 — Supplementary Information 1. [file 41598_2025_96918_MOESM1_ESM.zip › ALL_IDB Dataset/L3/Im009_1.tif]

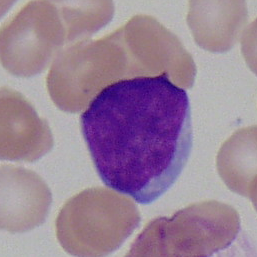

Supplement: Supplementary file 1 — Supplementary Information 1. [file 41598_2025_96918_MOESM1_ESM.zip › ALL_IDB Dataset/L3/Im010_1.tif]

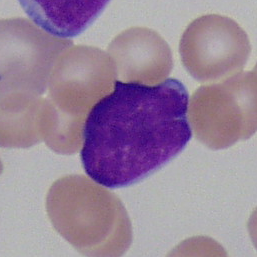

Supplement: Supplementary file 1 — Supplementary Information 1. [file 41598_2025_96918_MOESM1_ESM.zip › ALL_IDB Dataset/L3/Im011_1.tif]

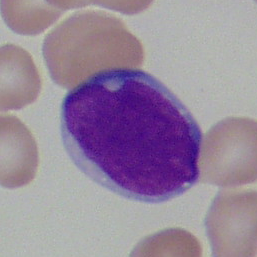

Supplement: Supplementary file 1 — Supplementary Information 1. [file 41598_2025_96918_MOESM1_ESM.zip › ALL_IDB Dataset/L3/Im012_1.tif]

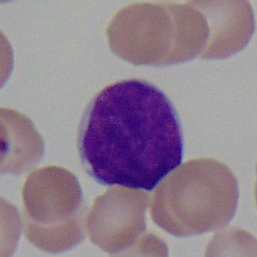

Supplement: Supplementary file 1 — Supplementary Information 1. [file 41598_2025_96918_MOESM1_ESM.zip › ALL_IDB Dataset/L3/Im013_1.tif]

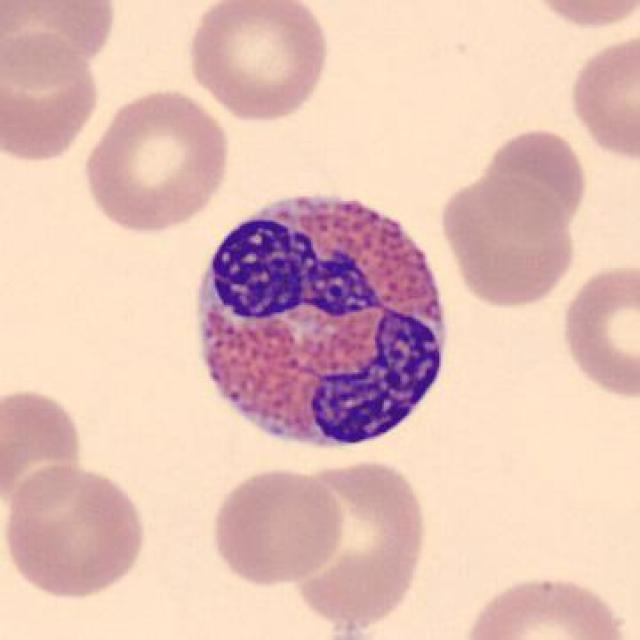

Supplement: Supplementary file 3 — Supplementary Information 3. [file 41598_2025_96918_MOESM3_ESM.zip › WBCs-v2.v2-v2.yolov8/test/images/EO_10150_jpg.rf.d4f0d38b76550c423363c5c8ddc6d801.jpg]

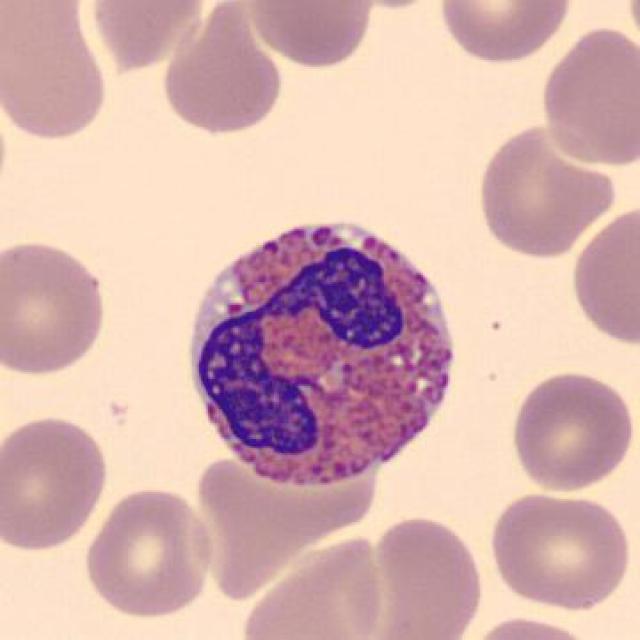

Supplement: Supplementary file 3 — Supplementary Information 3. [file 41598_2025_96918_MOESM3_ESM.zip › WBCs-v2.v2-v2.yolov8/test/images/EO_101852_jpg.rf.fb10e5b806534f5e9a25f3d84f2740da.jpg]

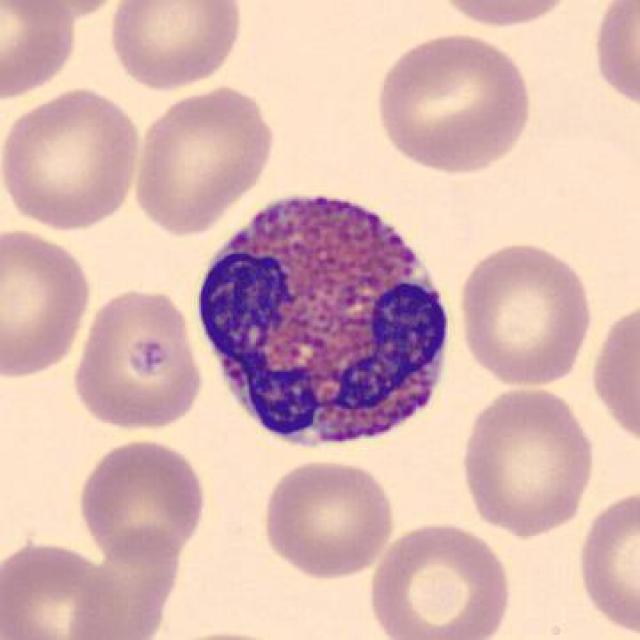

Supplement: Supplementary file 3 — Supplementary Information 3. [file 41598_2025_96918_MOESM3_ESM.zip › WBCs-v2.v2-v2.yolov8/test/images/EO_105010_jpg.rf.4054b9af3cbf0b0b9a6e1b30fdafaeac.jpg]

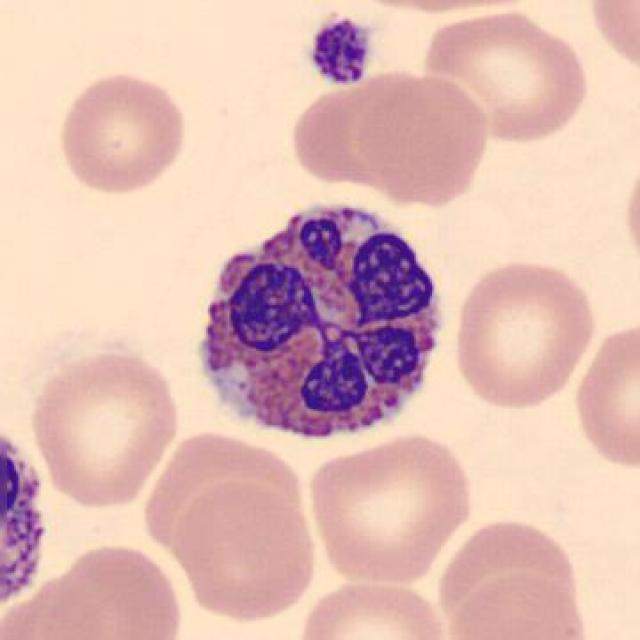

Supplement: Supplementary file 3 — Supplementary Information 3. [file 41598_2025_96918_MOESM3_ESM.zip › WBCs-v2.v2-v2.yolov8/test/images/EO_105961_jpg.rf.af0c72d6264e45b8476b5504ed9f6783.jpg]

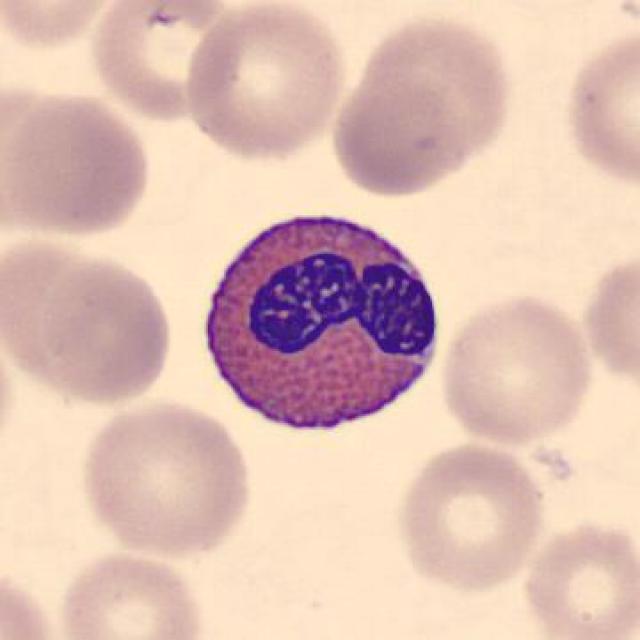

Supplement: Supplementary file 3 — Supplementary Information 3. [file 41598_2025_96918_MOESM3_ESM.zip › WBCs-v2.v2-v2.yolov8/test/images/EO_115792_jpg.rf.e8e03584efba6b2ffd5f505f6d80f5cb.jpg]

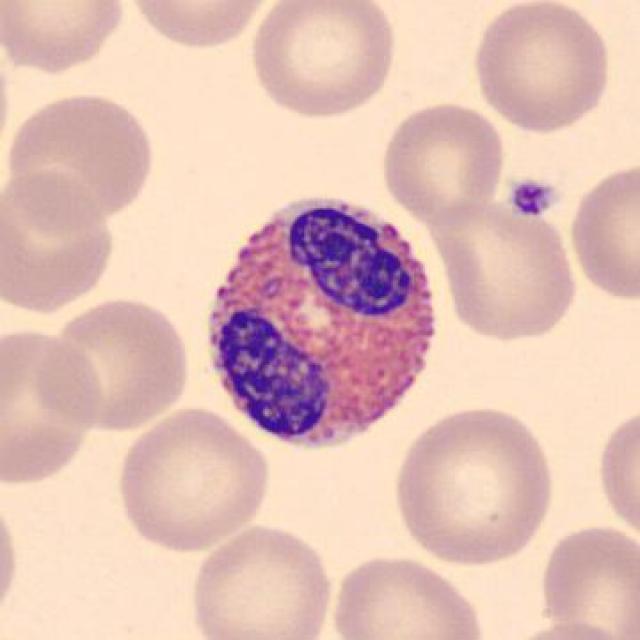

Supplement: Supplementary file 3 — Supplementary Information 3. [file 41598_2025_96918_MOESM3_ESM.zip › WBCs-v2.v2-v2.yolov8/test/images/EO_118338_jpg.rf.bc4c7e78bbda9534984373abd33ff3ac.jpg]

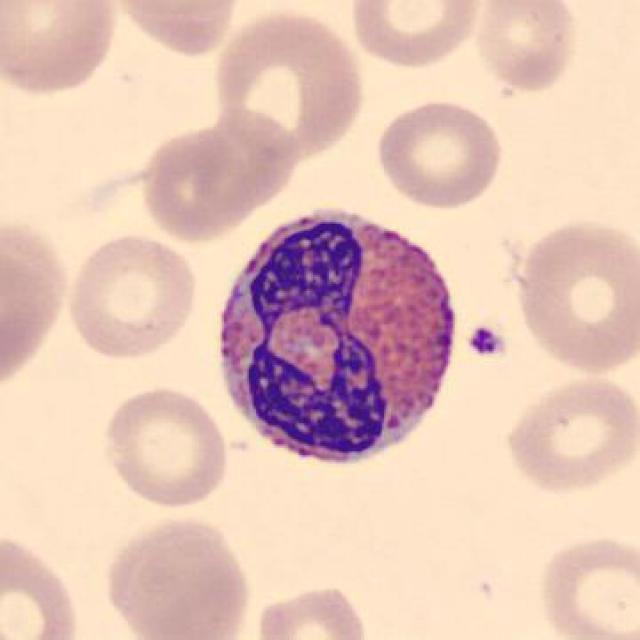

Supplement: Supplementary file 3 — Supplementary Information 3. [file 41598_2025_96918_MOESM3_ESM.zip › WBCs-v2.v2-v2.yolov8/test/images/EO_126623_jpg.rf.d8c22bde41492a3574c4be851c8085f8.jpg]

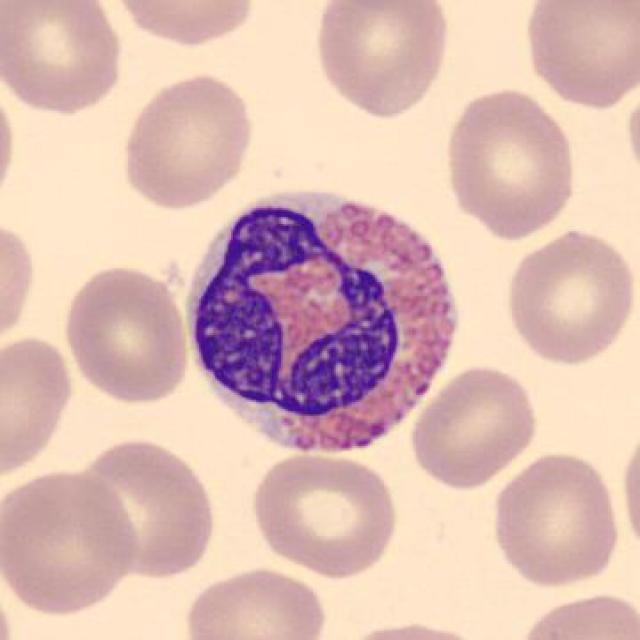

Supplement: Supplementary file 3 — Supplementary Information 3. [file 41598_2025_96918_MOESM3_ESM.zip › WBCs-v2.v2-v2.yolov8/test/images/EO_130195_jpg.rf.5558e88a78fdcc575cc6e8afd6bd0c64.jpg]

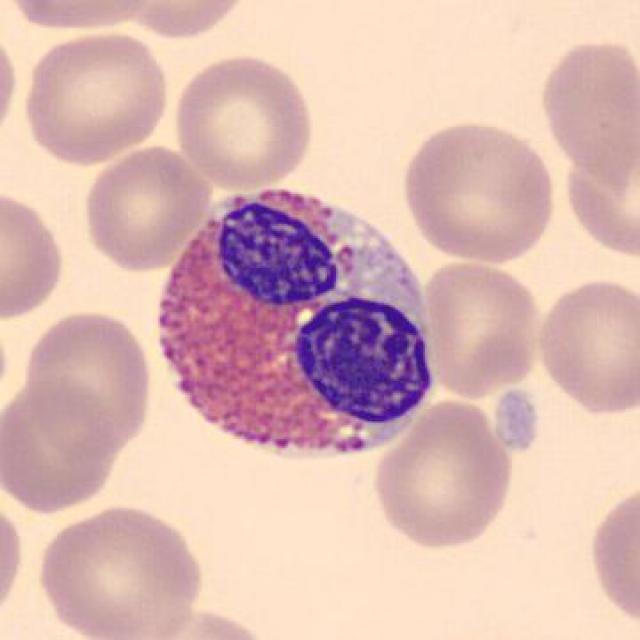

Supplement: Supplementary file 3 — Supplementary Information 3. [file 41598_2025_96918_MOESM3_ESM.zip › WBCs-v2.v2-v2.yolov8/test/images/EO_134423_jpg.rf.fb627e516439390b7c05b5f83beec0f9.jpg]

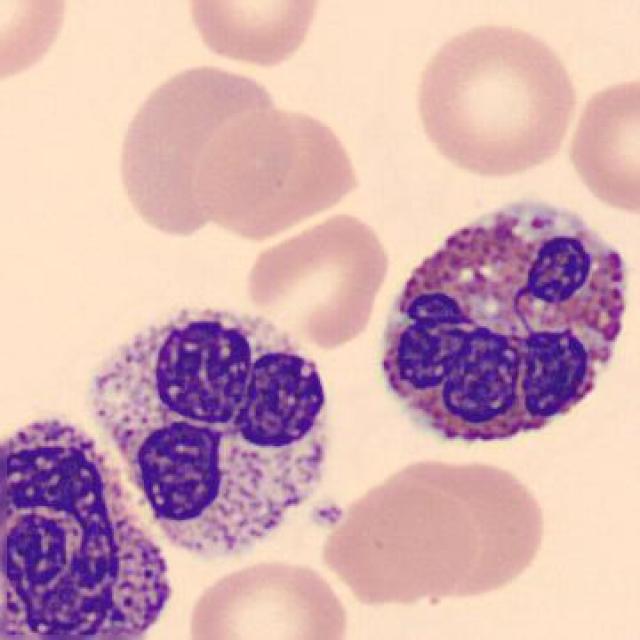

Supplement: Supplementary file 3 — Supplementary Information 3. [file 41598_2025_96918_MOESM3_ESM.zip › WBCs-v2.v2-v2.yolov8/test/images/EO_139723_jpg.rf.dc6af5fac172a98c0fea9ad7be3ec260.jpg]

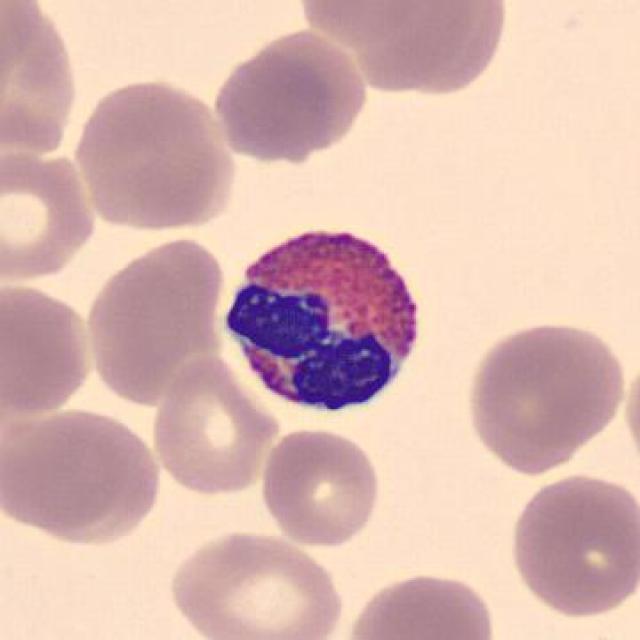

Supplement: Supplementary file 3 — Supplementary Information 3. [file 41598_2025_96918_MOESM3_ESM.zip › WBCs-v2.v2-v2.yolov8/test/images/EO_152922_jpg.rf.948ec9470ba8d840789e1fbfa49fc673.jpg]

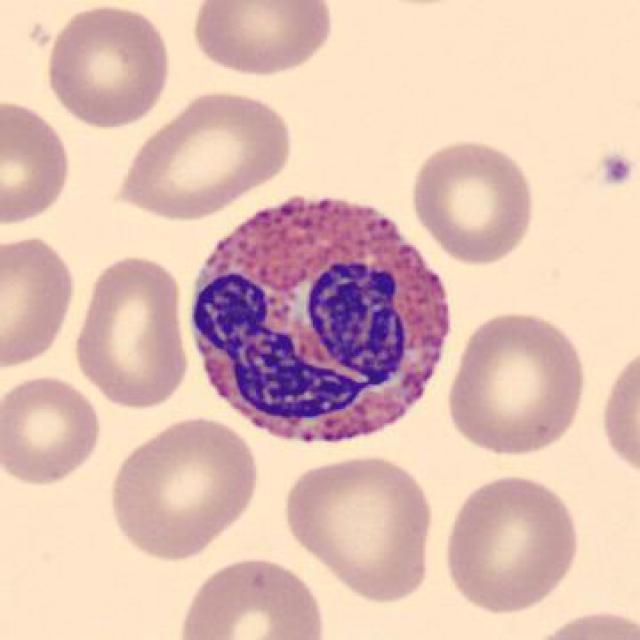

Supplement: Supplementary file 3 — Supplementary Information 3. [file 41598_2025_96918_MOESM3_ESM.zip › WBCs-v2.v2-v2.yolov8/test/images/EO_1554_jpg.rf.12e469eb3b856193cf57b4748658aad0.jpg]

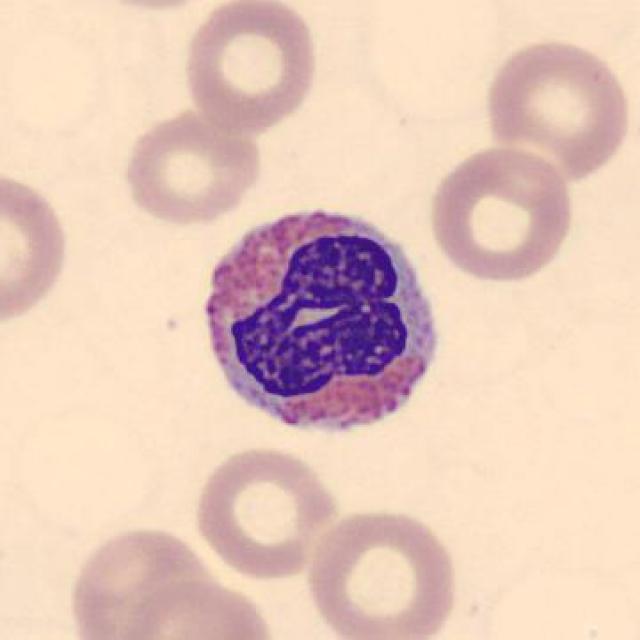

Supplement: Supplementary file 3 — Supplementary Information 3. [file 41598_2025_96918_MOESM3_ESM.zip › WBCs-v2.v2-v2.yolov8/test/images/EO_158113_jpg.rf.49e075ac0d2073f053dc1816dcc5b9c3.jpg]

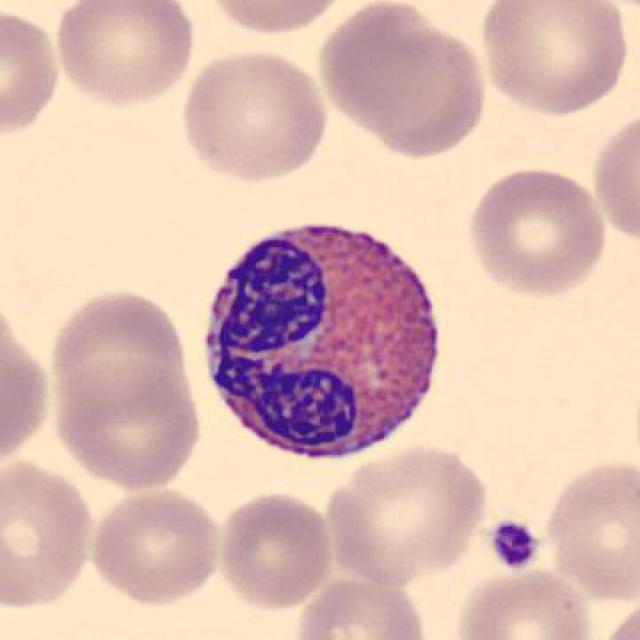

Supplement: Supplementary file 3 — Supplementary Information 3. [file 41598_2025_96918_MOESM3_ESM.zip › WBCs-v2.v2-v2.yolov8/test/images/EO_159306_jpg.rf.7abf6588279818bc06f994108d86d37d.jpg]

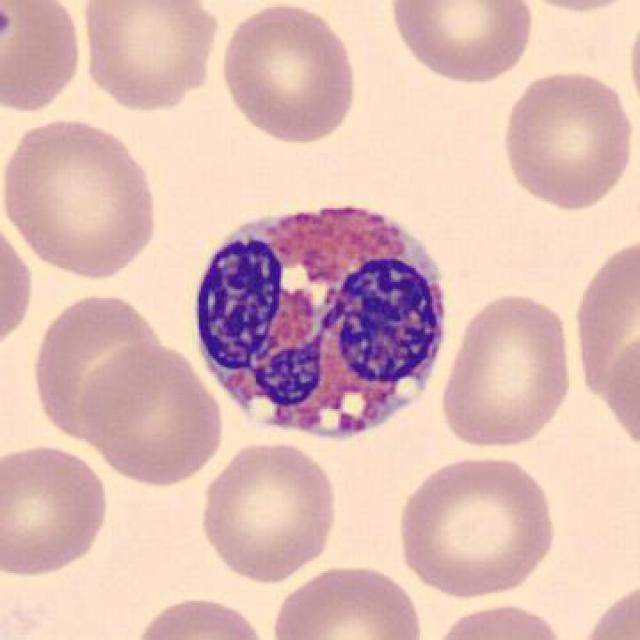

Supplement: Supplementary file 3 — Supplementary Information 3. [file 41598_2025_96918_MOESM3_ESM.zip › WBCs-v2.v2-v2.yolov8/test/images/EO_164571_jpg.rf.acd9eea4a80e8d85a20d5b6aa50bc925.jpg]

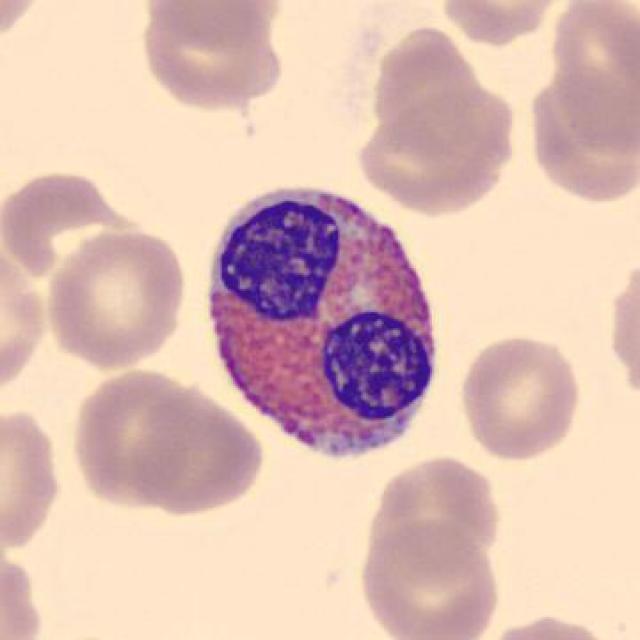

Supplement: Supplementary file 3 — Supplementary Information 3. [file 41598_2025_96918_MOESM3_ESM.zip › WBCs-v2.v2-v2.yolov8/test/images/EO_164591_jpg.rf.f31978a5ada0b4d92fb3d2e681736dea.jpg]

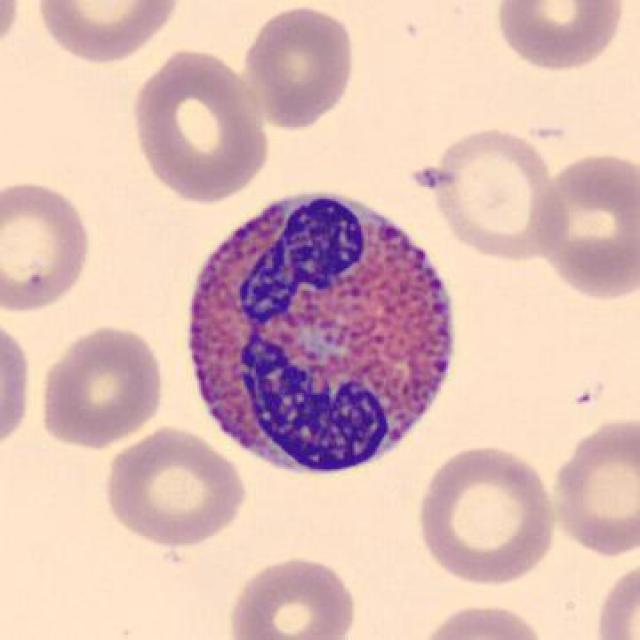

Supplement: Supplementary file 3 — Supplementary Information 3. [file 41598_2025_96918_MOESM3_ESM.zip › WBCs-v2.v2-v2.yolov8/test/images/EO_165140_jpg.rf.8b2a7273783698ca52d01f96b1502c5b.jpg]

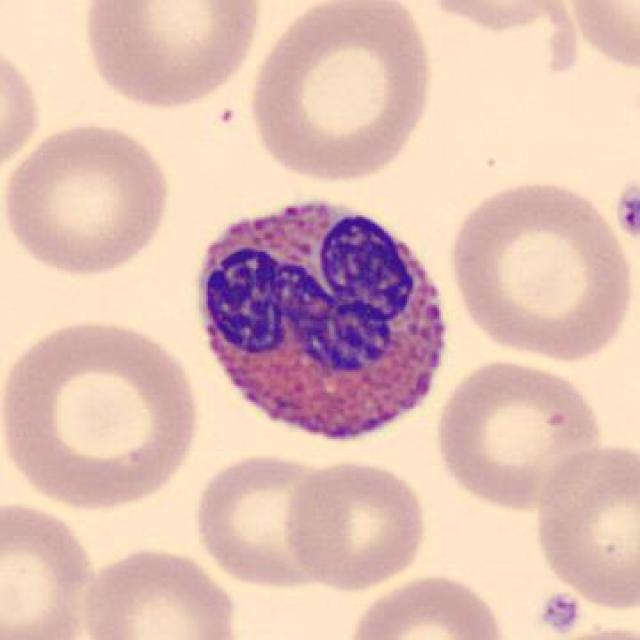

Supplement: Supplementary file 3 — Supplementary Information 3. [file 41598_2025_96918_MOESM3_ESM.zip › WBCs-v2.v2-v2.yolov8/test/images/EO_165152_jpg.rf.78f7cf10a20b0253fbb5eb15cce74b2f.jpg]

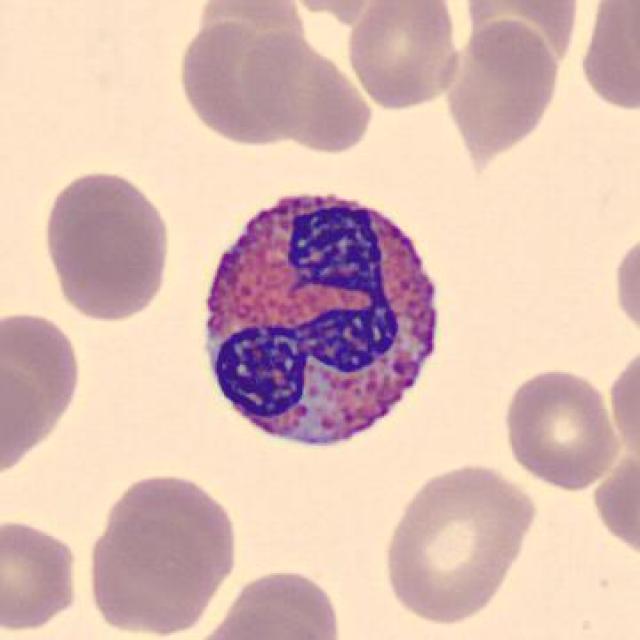

Supplement: Supplementary file 3 — Supplementary Information 3. [file 41598_2025_96918_MOESM3_ESM.zip › WBCs-v2.v2-v2.yolov8/test/images/EO_167179_jpg.rf.f29646a00b3d40a4611c86b0a8ba5ea2.jpg]

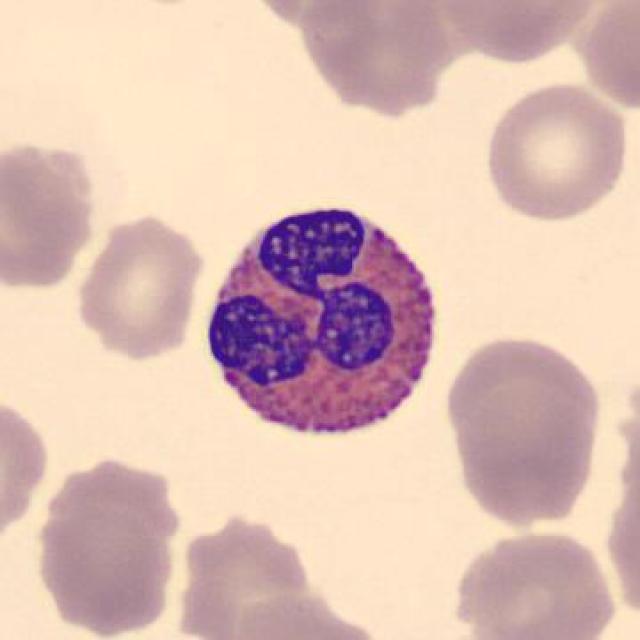

Supplement: Supplementary file 3 — Supplementary Information 3. [file 41598_2025_96918_MOESM3_ESM.zip › WBCs-v2.v2-v2.yolov8/test/images/EO_182479_jpg.rf.4ee6e2f05d63ed28393c00a9759299c1.jpg]

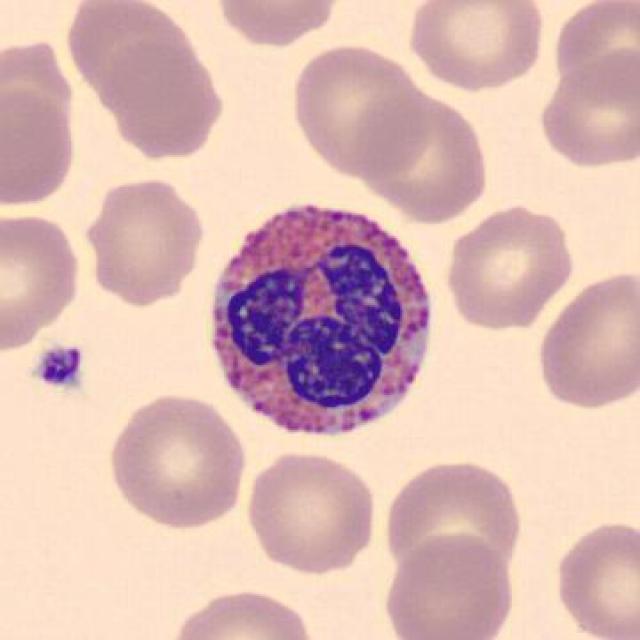

Supplement: Supplementary file 3 — Supplementary Information 3. [file 41598_2025_96918_MOESM3_ESM.zip › WBCs-v2.v2-v2.yolov8/test/images/EO_190201_jpg.rf.dfe37705e1d90fd6c5a1b6894947f850.jpg]

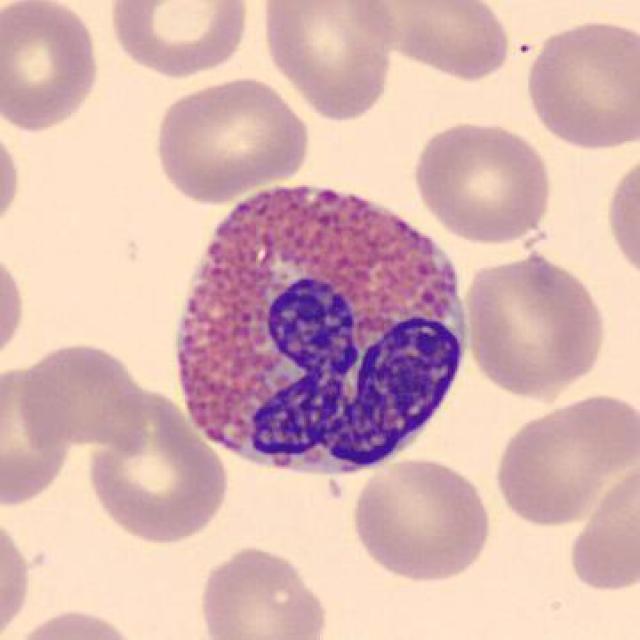

Supplement: Supplementary file 3 — Supplementary Information 3. [file 41598_2025_96918_MOESM3_ESM.zip › WBCs-v2.v2-v2.yolov8/test/images/EO_194014_jpg.rf.3f87a20c4d6d05aaca8b85de3ef28b1f.jpg]

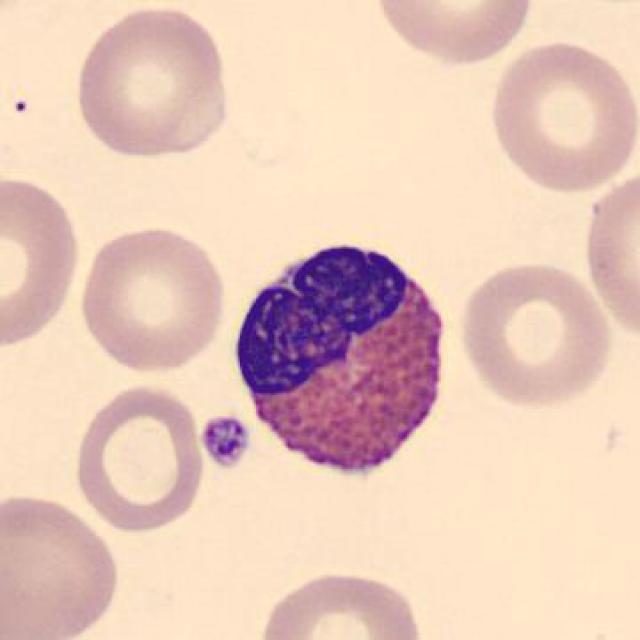

Supplement: Supplementary file 3 — Supplementary Information 3. [file 41598_2025_96918_MOESM3_ESM.zip › WBCs-v2.v2-v2.yolov8/test/images/EO_197001_jpg.rf.65c6cd31fc5cd92267a5834d2963f493.jpg]

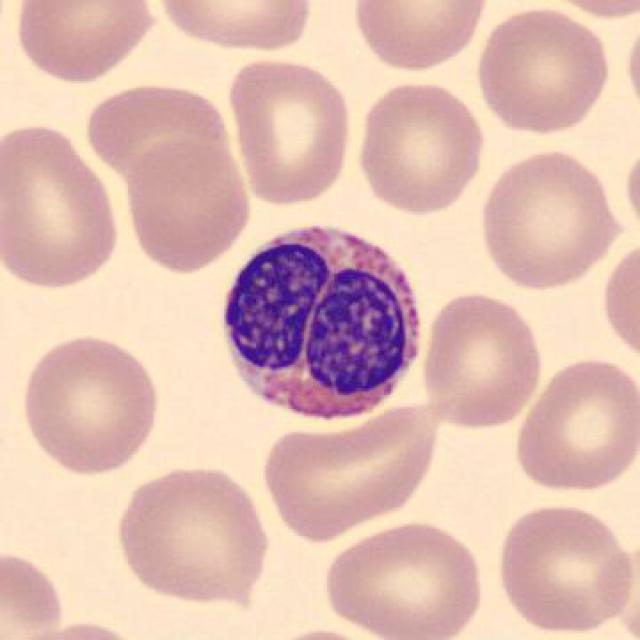

Supplement: Supplementary file 3 — Supplementary Information 3. [file 41598_2025_96918_MOESM3_ESM.zip › WBCs-v2.v2-v2.yolov8/test/images/EO_201647_jpg.rf.8c558191a8cdf91c6e569d4d573353a7.jpg]

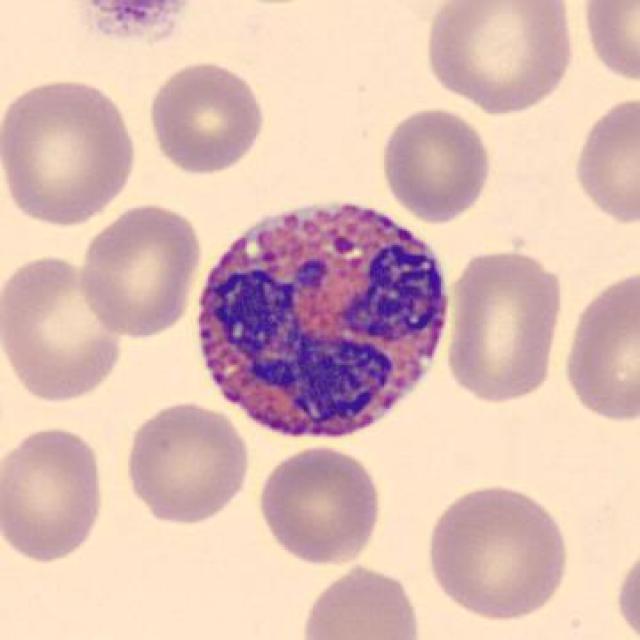

Supplement: Supplementary file 3 — Supplementary Information 3. [file 41598_2025_96918_MOESM3_ESM.zip › WBCs-v2.v2-v2.yolov8/test/images/EO_20293_jpg.rf.b0f4f02a5286d17a4326cb70b4cdb3d1.jpg]

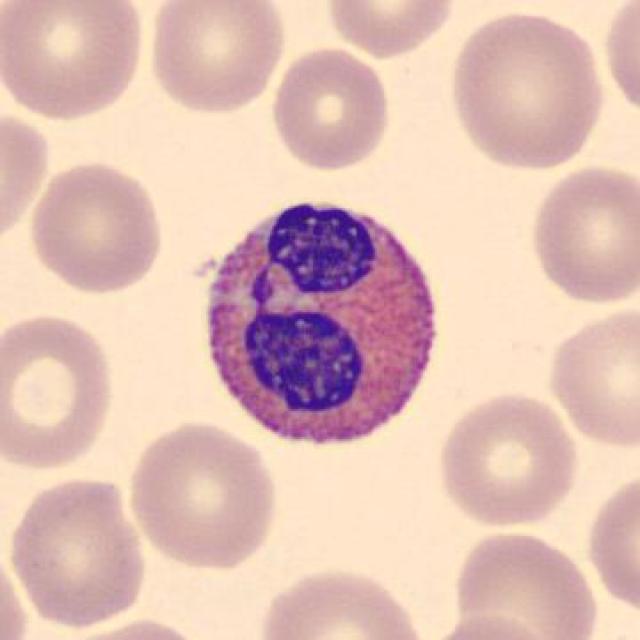

Supplement: Supplementary file 3 — Supplementary Information 3. [file 41598_2025_96918_MOESM3_ESM.zip › WBCs-v2.v2-v2.yolov8/test/images/EO_209322_jpg.rf.f86b94e0f11c9c804933992b24431e30.jpg]

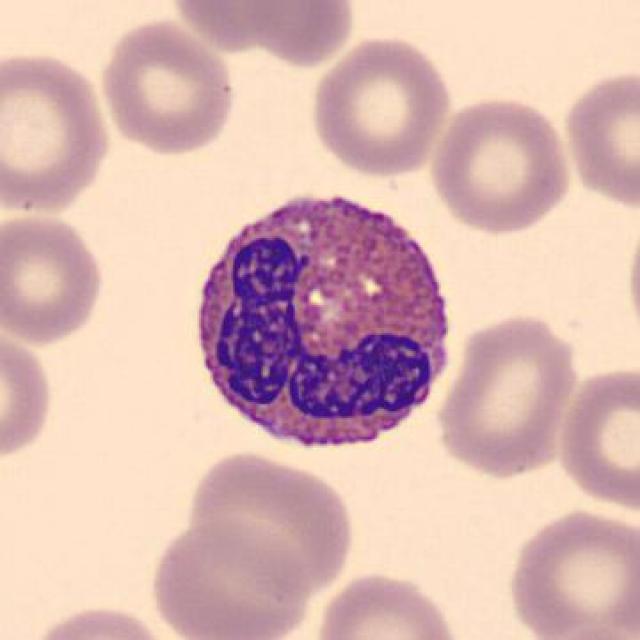

Supplement: Supplementary file 3 — Supplementary Information 3. [file 41598_2025_96918_MOESM3_ESM.zip › WBCs-v2.v2-v2.yolov8/test/images/EO_210086_jpg.rf.9eb8ce58951654946f8092b9f5e28a41.jpg]

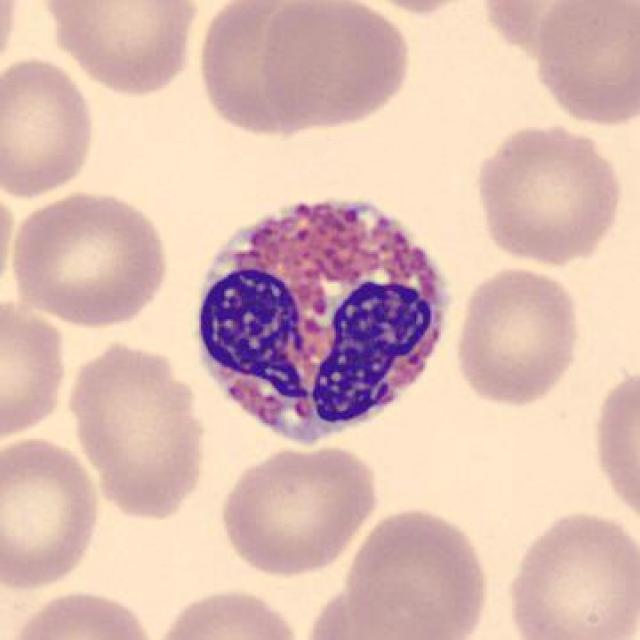

Supplement: Supplementary file 3 — Supplementary Information 3. [file 41598_2025_96918_MOESM3_ESM.zip › WBCs-v2.v2-v2.yolov8/test/images/EO_210291_jpg.rf.ec2262aea7bdb44ee97b488edde47a32.jpg]

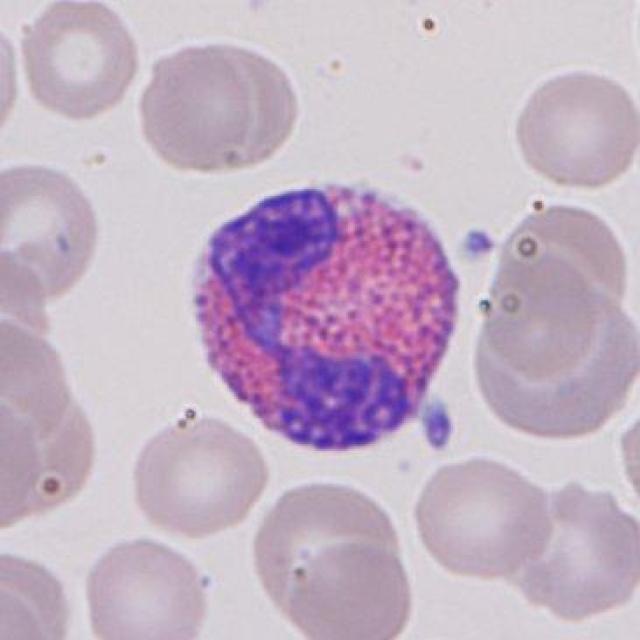

Supplement: Supplementary file 3 — Supplementary Information 3. [file 41598_2025_96918_MOESM3_ESM.zip › WBCs-v2.v2-v2.yolov8/test/images/EO_212014_jpg.rf.3b2b256ca780c57a4f43856965805ca8.jpg]

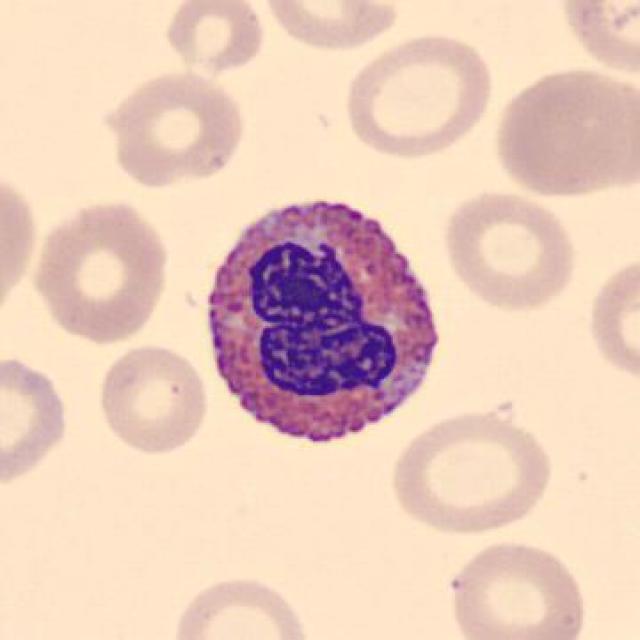

Supplement: Supplementary file 3 — Supplementary Information 3. [file 41598_2025_96918_MOESM3_ESM.zip › WBCs-v2.v2-v2.yolov8/test/images/EO_212509_jpg.rf.72433d040b93b8bedbb26de399ec433d.jpg]

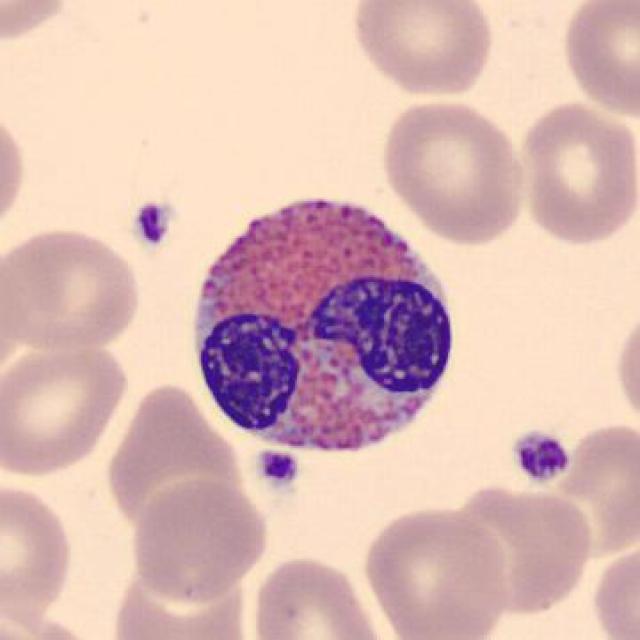

Supplement: Supplementary file 3 — Supplementary Information 3. [file 41598_2025_96918_MOESM3_ESM.zip › WBCs-v2.v2-v2.yolov8/test/images/EO_214340_jpg.rf.3aaec6bb9441c85c4e8bb36581cf1ee7.jpg]

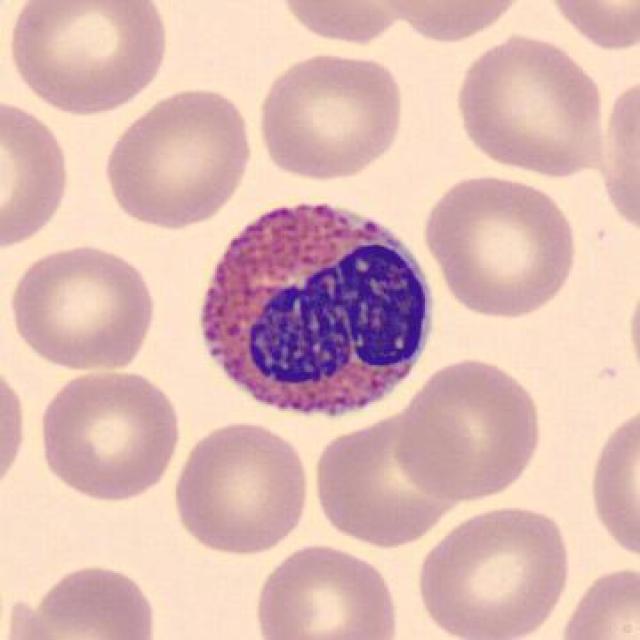

Supplement: Supplementary file 3 — Supplementary Information 3. [file 41598_2025_96918_MOESM3_ESM.zip › WBCs-v2.v2-v2.yolov8/test/images/EO_214664_jpg.rf.afeac62b12790570595e86c24fe1c5e8.jpg]

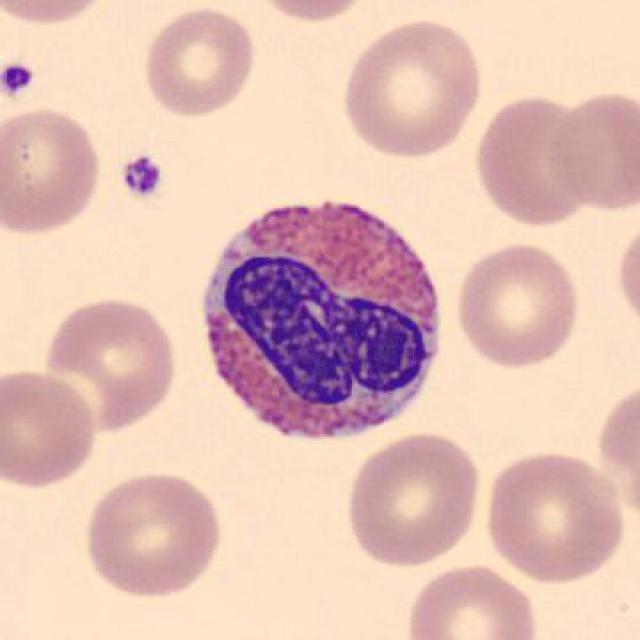

Supplement: Supplementary file 3 — Supplementary Information 3. [file 41598_2025_96918_MOESM3_ESM.zip › WBCs-v2.v2-v2.yolov8/test/images/EO_220632_jpg.rf.accbbb8379ee9eac859b157f0532cb7f.jpg]

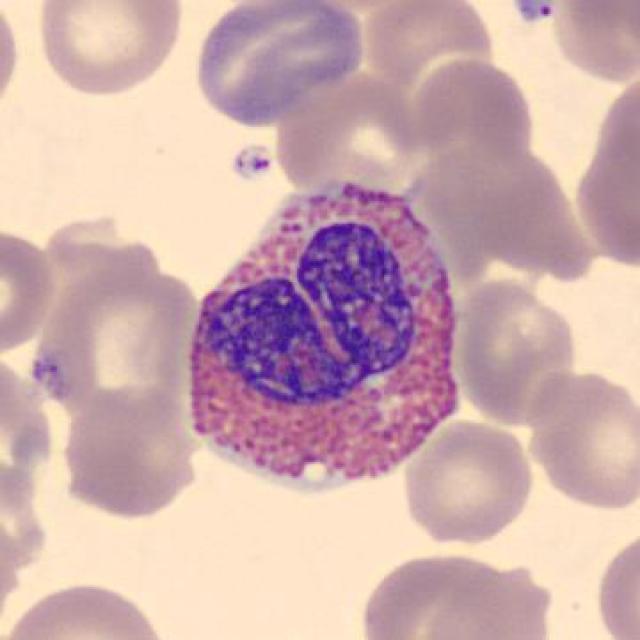

Supplement: Supplementary file 3 — Supplementary Information 3. [file 41598_2025_96918_MOESM3_ESM.zip › WBCs-v2.v2-v2.yolov8/test/images/EO_221996_jpg.rf.6ad2d964cb9dc656e3559db154dd4cea.jpg]

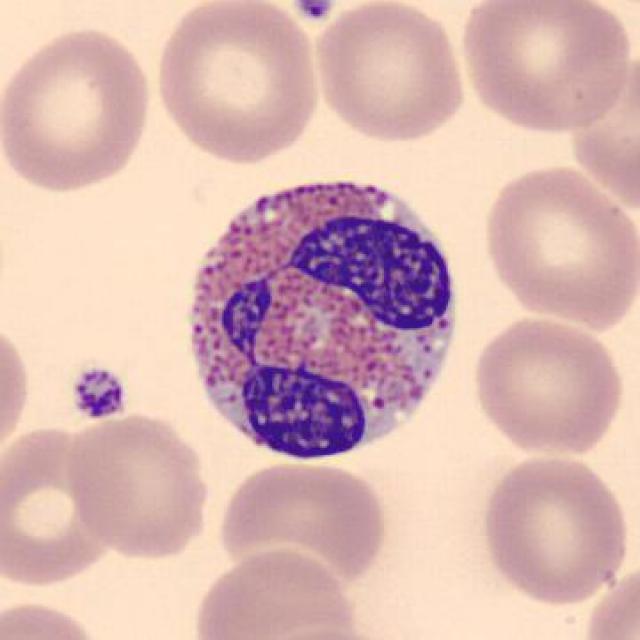

Supplement: Supplementary file 3 — Supplementary Information 3. [file 41598_2025_96918_MOESM3_ESM.zip › WBCs-v2.v2-v2.yolov8/test/images/EO_223866_jpg.rf.b517088bb44fe7869a535cc7eacf7022.jpg]

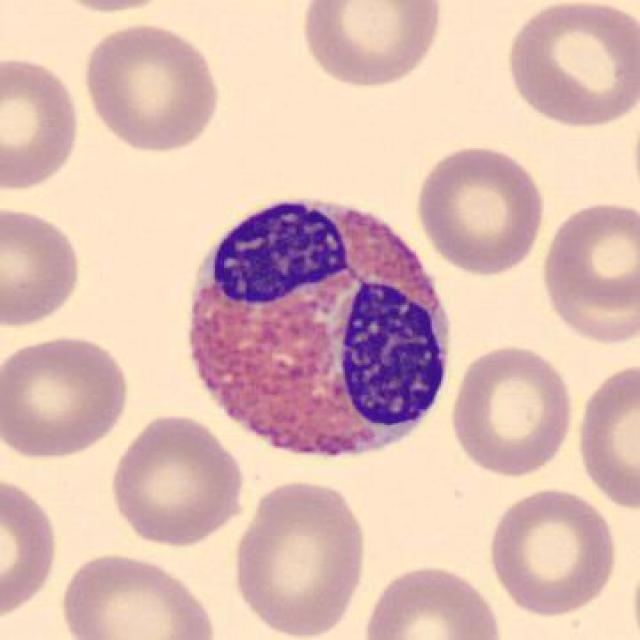

Supplement: Supplementary file 3 — Supplementary Information 3. [file 41598_2025_96918_MOESM3_ESM.zip › WBCs-v2.v2-v2.yolov8/test/images/EO_223868_jpg.rf.30b5637ba8661c86bcdbab3527f183d7.jpg]

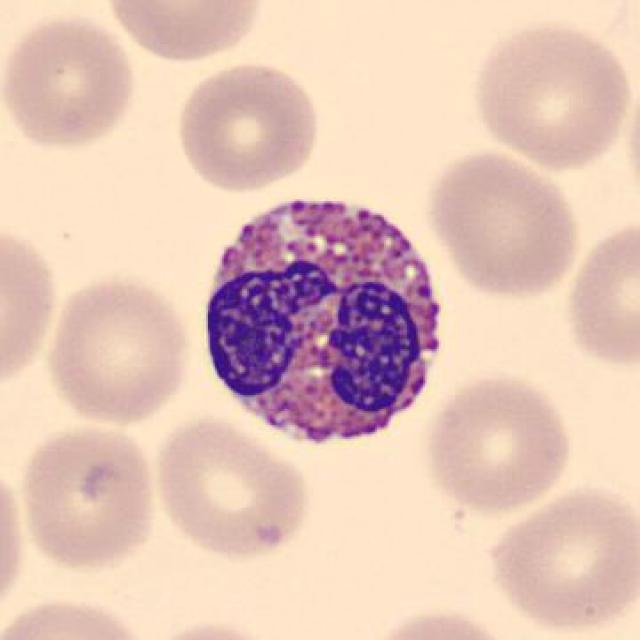

Supplement: Supplementary file 3 — Supplementary Information 3. [file 41598_2025_96918_MOESM3_ESM.zip › WBCs-v2.v2-v2.yolov8/test/images/EO_22814_jpg.rf.941cd9d2a3003f77936459e1c041dd3f.jpg]

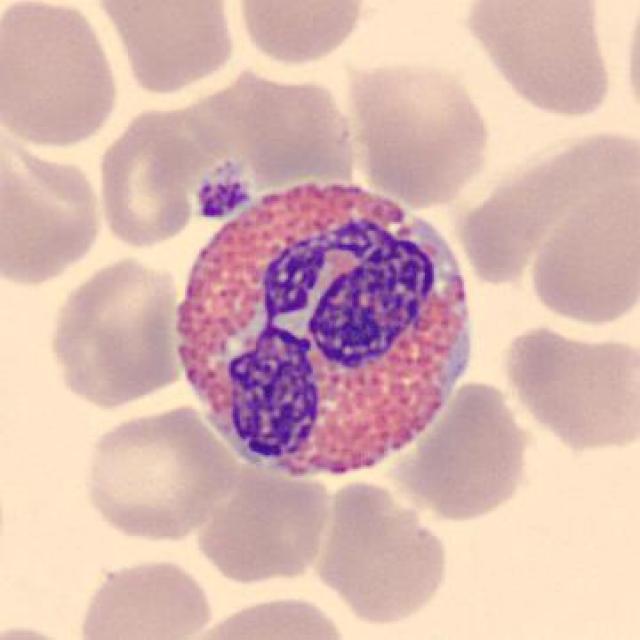

Supplement: Supplementary file 3 — Supplementary Information 3. [file 41598_2025_96918_MOESM3_ESM.zip › WBCs-v2.v2-v2.yolov8/test/images/EO_230357_jpg.rf.aaed17e0d01974b8b02e26834681907a.jpg]

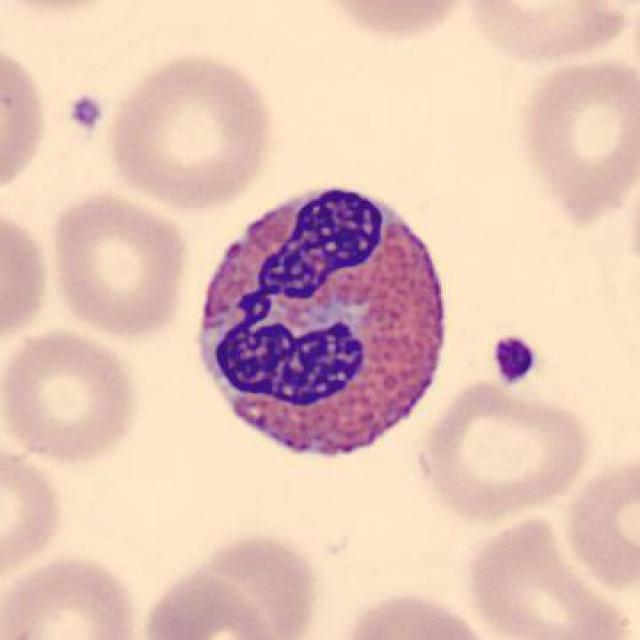

Supplement: Supplementary file 3 — Supplementary Information 3. [file 41598_2025_96918_MOESM3_ESM.zip › WBCs-v2.v2-v2.yolov8/test/images/EO_23089841_jpg.rf.3631f1e541f50d4e92122d532f64939b.jpg]

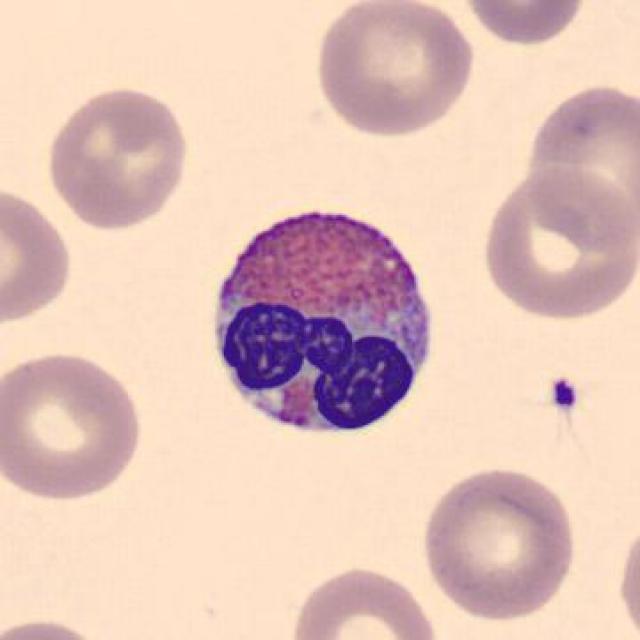

Supplement: Supplementary file 3 — Supplementary Information 3. [file 41598_2025_96918_MOESM3_ESM.zip › WBCs-v2.v2-v2.yolov8/test/images/EO_247089_jpg.rf.fdc412e1643a7553dbd9274931ca6ad0.jpg]

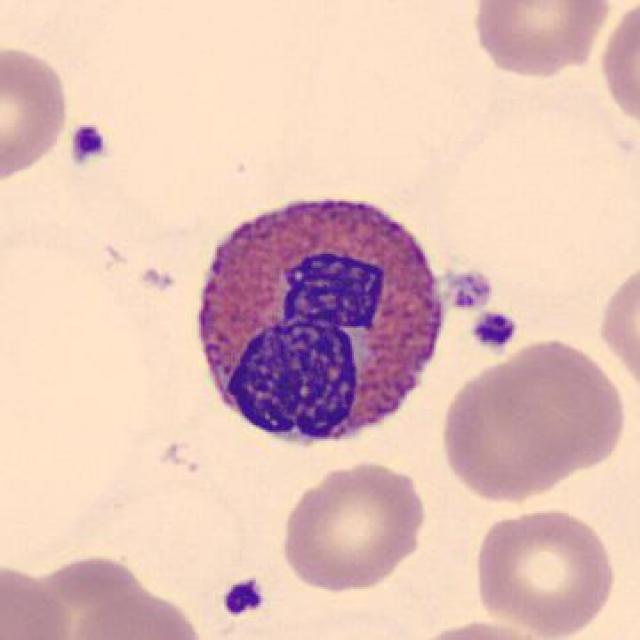

Supplement: Supplementary file 3 — Supplementary Information 3. [file 41598_2025_96918_MOESM3_ESM.zip › WBCs-v2.v2-v2.yolov8/test/images/EO_247533_jpg.rf.4a03797dfc0045393e92d5659ce615ad.jpg]

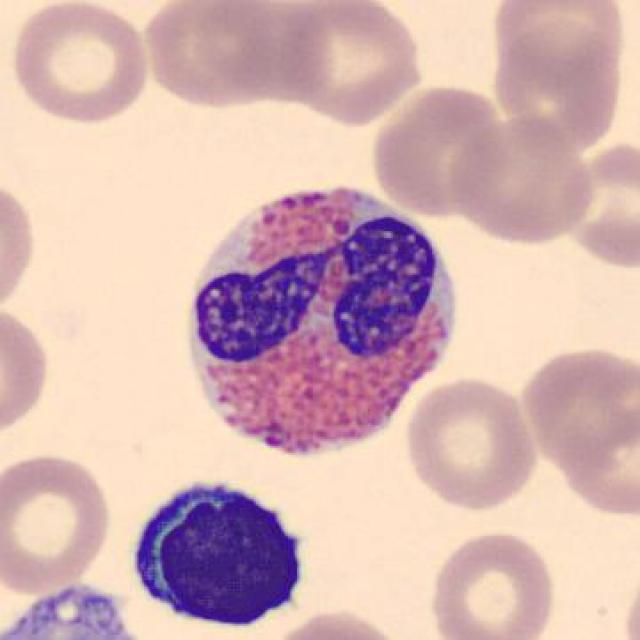

Supplement: Supplementary file 3 — Supplementary Information 3. [file 41598_2025_96918_MOESM3_ESM.zip › WBCs-v2.v2-v2.yolov8/test/images/EO_250252_jpg.rf.f2beef6a7ffb489d750173e706e1585e.jpg]

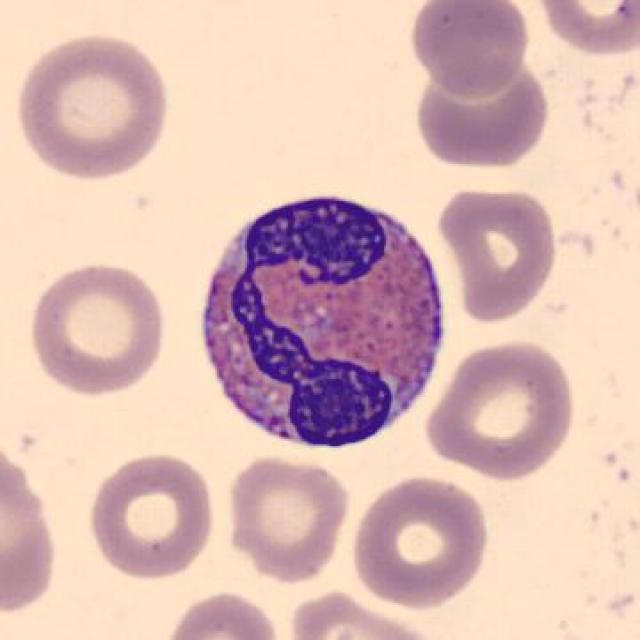

Supplement: Supplementary file 3 — Supplementary Information 3. [file 41598_2025_96918_MOESM3_ESM.zip › WBCs-v2.v2-v2.yolov8/test/images/EO_250649_jpg.rf.85a8a129964d0d34d7e93d4e0d32a2cc.jpg]

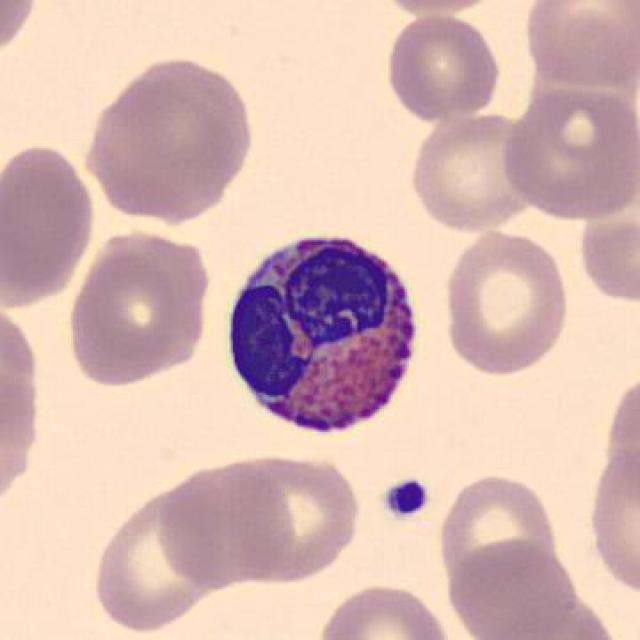

Supplement: Supplementary file 3 — Supplementary Information 3. [file 41598_2025_96918_MOESM3_ESM.zip › WBCs-v2.v2-v2.yolov8/test/images/EO_251668_jpg.rf.e154242bd8b36a991128cd07e1590647.jpg]

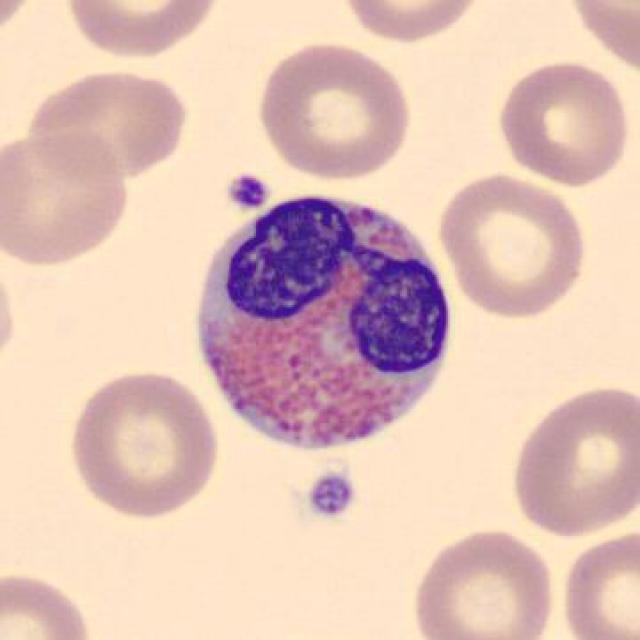

Supplement: Supplementary file 3 — Supplementary Information 3. [file 41598_2025_96918_MOESM3_ESM.zip › WBCs-v2.v2-v2.yolov8/test/images/EO_258490_jpg.rf.c7510dd86a1c31616271b46803a7b4de.jpg]

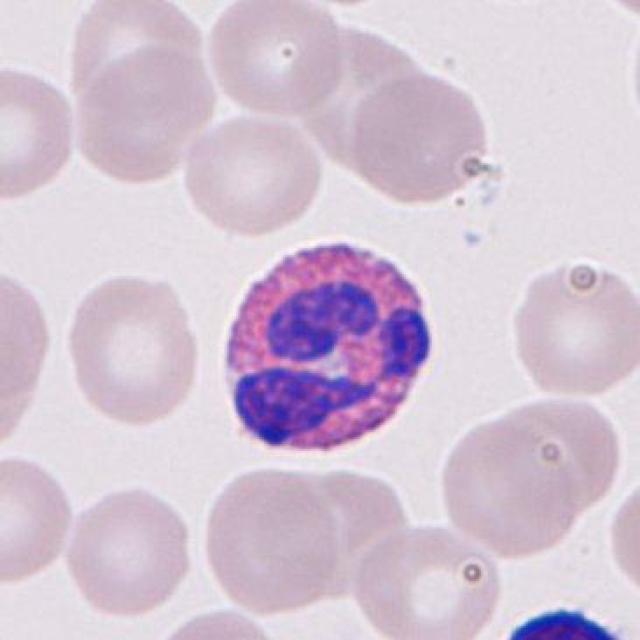

Supplement: Supplementary file 3 — Supplementary Information 3. [file 41598_2025_96918_MOESM3_ESM.zip › WBCs-v2.v2-v2.yolov8/test/images/EO_260857_jpg.rf.00da536757a23a25255ee199d3b3618e.jpg]

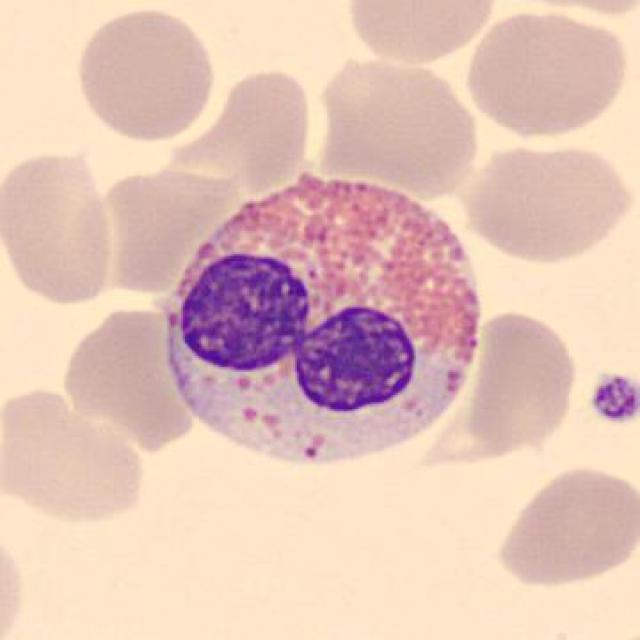

Supplement: Supplementary file 3 — Supplementary Information 3. [file 41598_2025_96918_MOESM3_ESM.zip › WBCs-v2.v2-v2.yolov8/test/images/EO_262501_jpg.rf.22f13754d5d8bbf4df6224819ffee795.jpg]

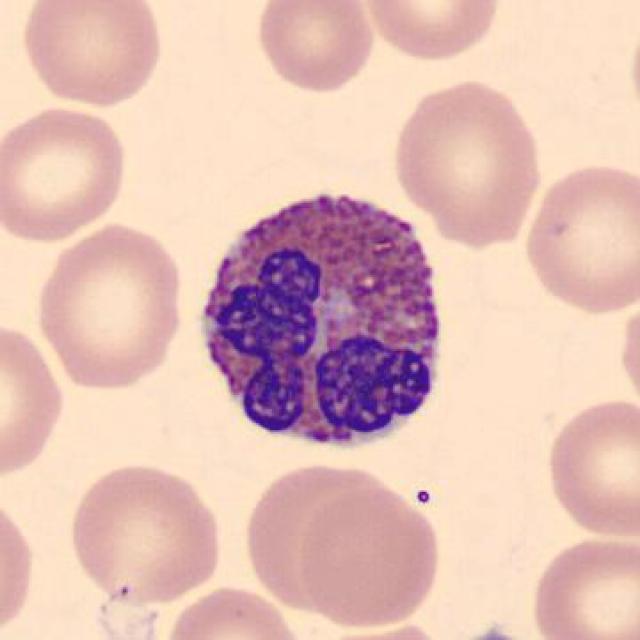

Supplement: Supplementary file 3 — Supplementary Information 3. [file 41598_2025_96918_MOESM3_ESM.zip › WBCs-v2.v2-v2.yolov8/test/images/EO_265516_jpg.rf.54c8ba8520394429019d498d20f81f8d.jpg]

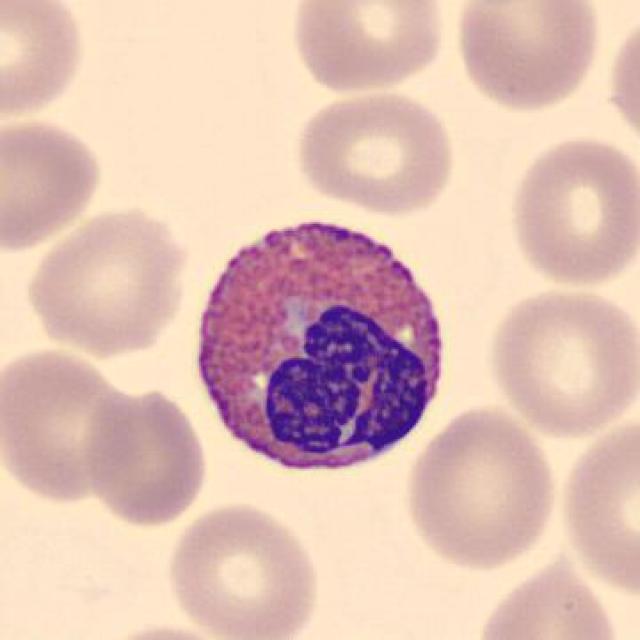

Supplement: Supplementary file 3 — Supplementary Information 3. [file 41598_2025_96918_MOESM3_ESM.zip › WBCs-v2.v2-v2.yolov8/test/images/EO_265598_jpg.rf.8f274789f309a219e3a45bb1d2ca4c8e.jpg]

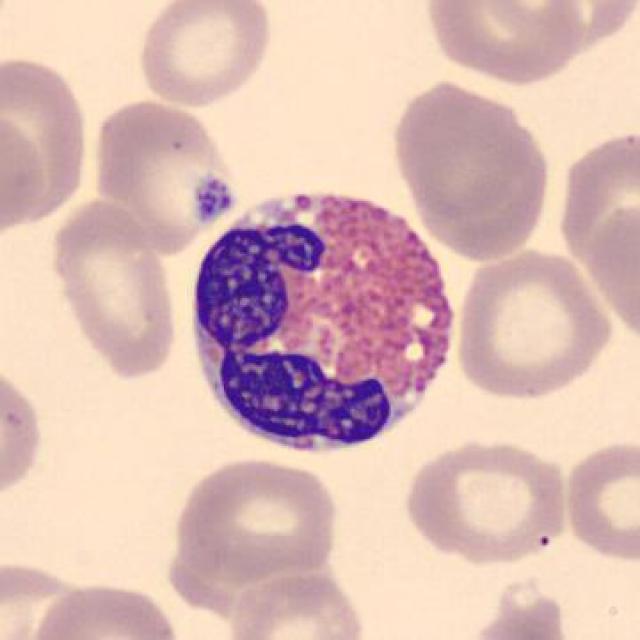

Supplement: Supplementary file 3 — Supplementary Information 3. [file 41598_2025_96918_MOESM3_ESM.zip › WBCs-v2.v2-v2.yolov8/test/images/EO_265718_jpg.rf.f9f7d9d1b68dd5b386130520a33c1ae6.jpg]

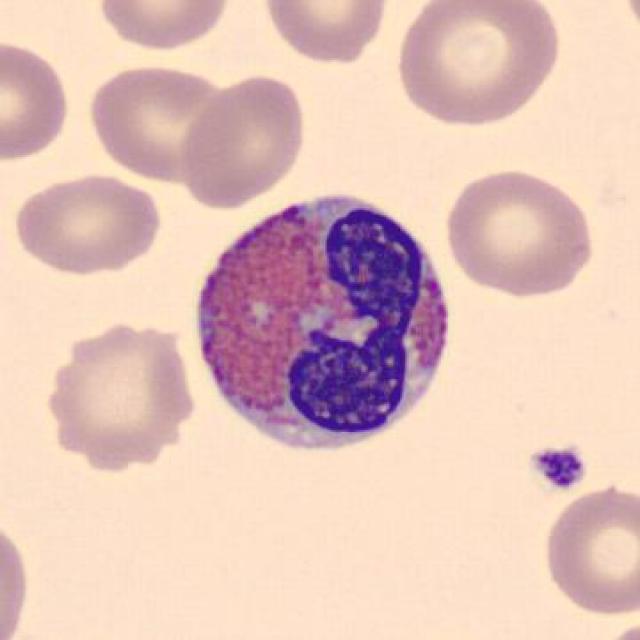

Supplement: Supplementary file 3 — Supplementary Information 3. [file 41598_2025_96918_MOESM3_ESM.zip › WBCs-v2.v2-v2.yolov8/test/images/EO_268984_jpg.rf.27e0c9acda95251b98ca0e6bcf991288.jpg]

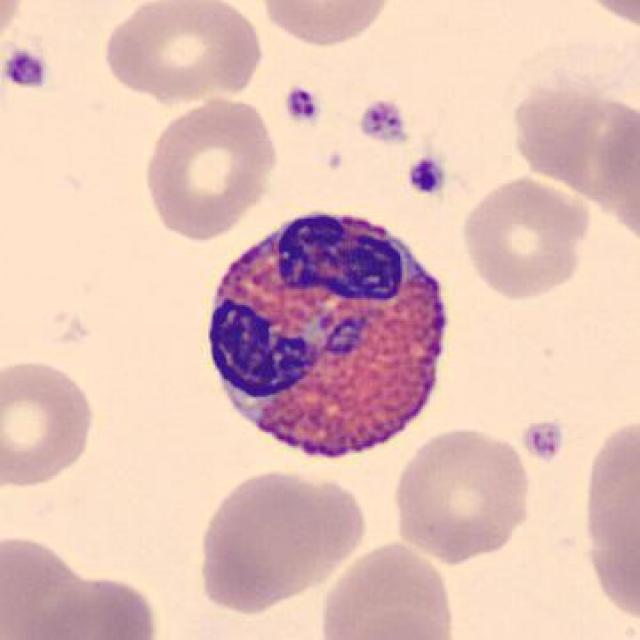

Supplement: Supplementary file 3 — Supplementary Information 3. [file 41598_2025_96918_MOESM3_ESM.zip › WBCs-v2.v2-v2.yolov8/test/images/EO_273049_jpg.rf.81ba98f2bffa560764f8971e7c9f8e72.jpg]

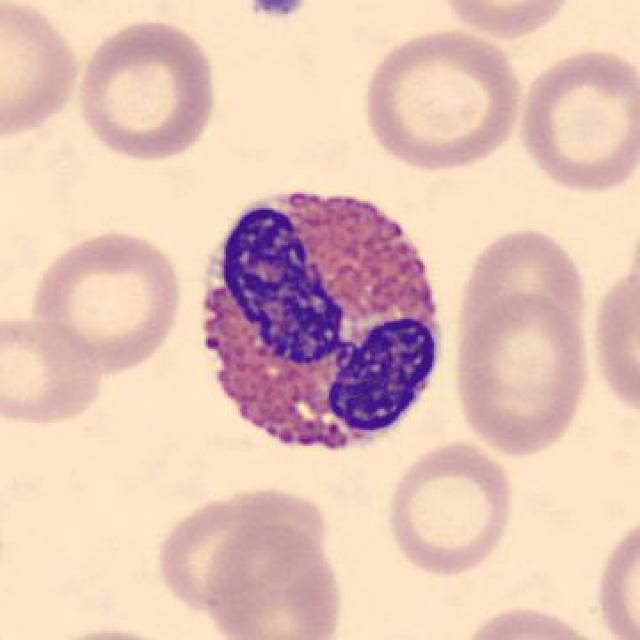

Supplement: Supplementary file 3 — Supplementary Information 3. [file 41598_2025_96918_MOESM3_ESM.zip › WBCs-v2.v2-v2.yolov8/test/images/EO_276119_jpg.rf.e71210c9d5ff0f919542ce22ab0ec008.jpg]

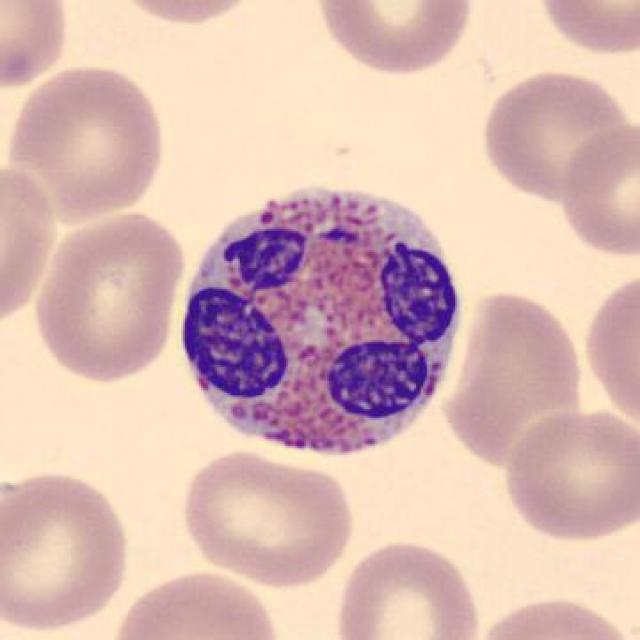

Supplement: Supplementary file 3 — Supplementary Information 3. [file 41598_2025_96918_MOESM3_ESM.zip › WBCs-v2.v2-v2.yolov8/test/images/EO_277497_jpg.rf.4fddbfc669860a1f7f3e9bd44973c14e.jpg]

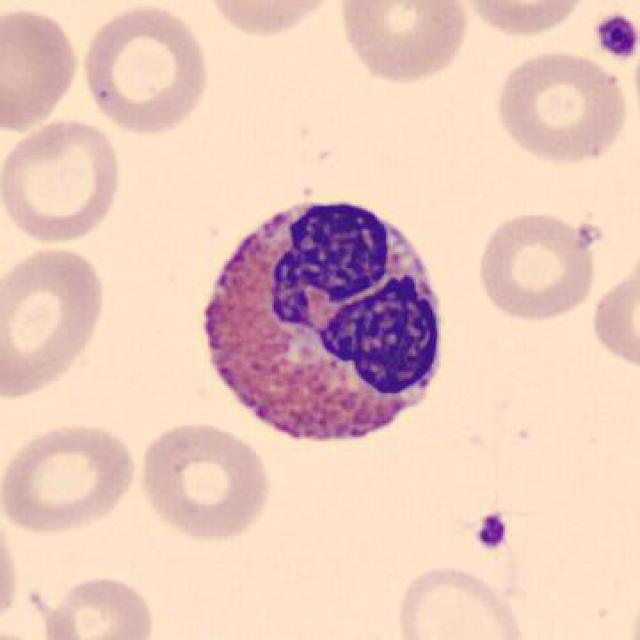

Supplement: Supplementary file 3 — Supplementary Information 3. [file 41598_2025_96918_MOESM3_ESM.zip › WBCs-v2.v2-v2.yolov8/test/images/EO_286161_jpg.rf.cf9d6de73ff9d0949667bfd6e37236f2.jpg]

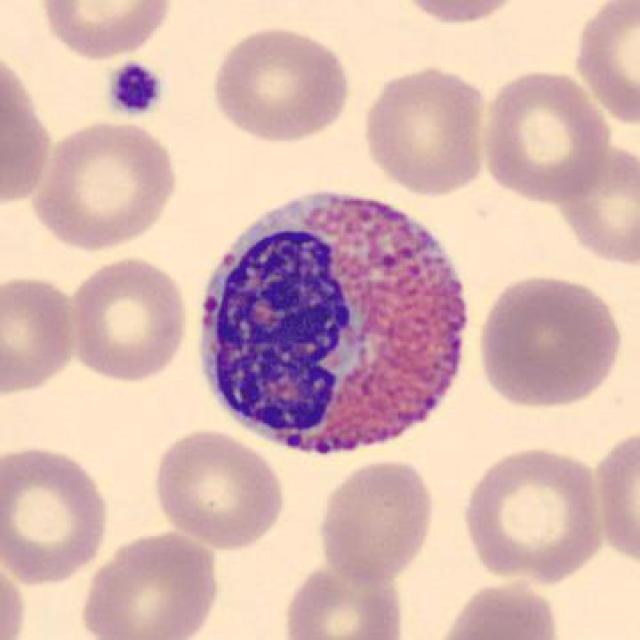

Supplement: Supplementary file 3 — Supplementary Information 3. [file 41598_2025_96918_MOESM3_ESM.zip › WBCs-v2.v2-v2.yolov8/test/images/EO_287394_jpg.rf.56d56d419be42e6542aa63585410d605.jpg]

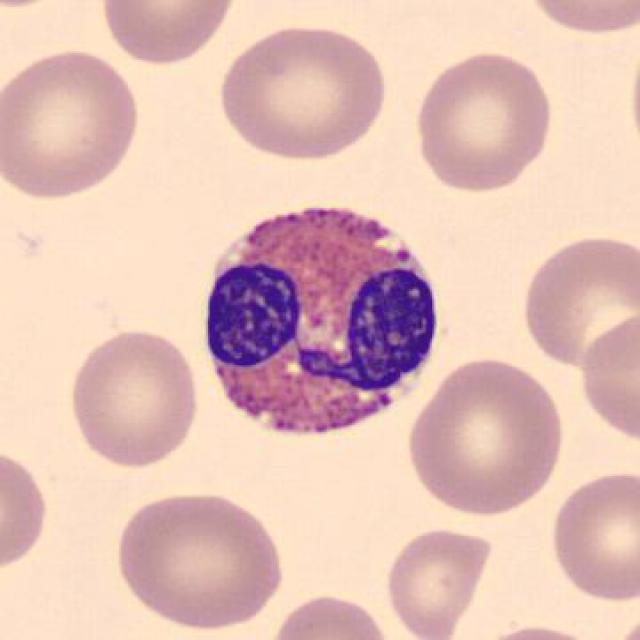

Supplement: Supplementary file 3 — Supplementary Information 3. [file 41598_2025_96918_MOESM3_ESM.zip › WBCs-v2.v2-v2.yolov8/test/images/EO_287859_jpg.rf.a38f64790710364ebeb1e3b648e068ff.jpg]

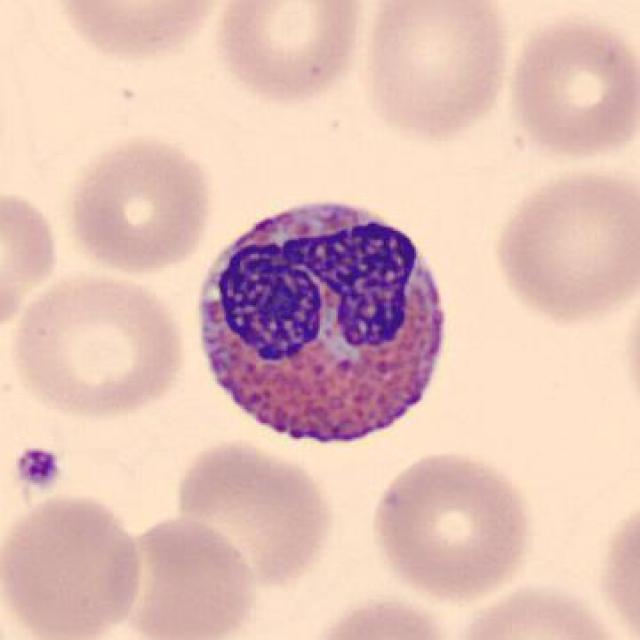

Supplement: Supplementary file 3 — Supplementary Information 3. [file 41598_2025_96918_MOESM3_ESM.zip › WBCs-v2.v2-v2.yolov8/test/images/EO_288126_jpg.rf.ebf09d10ce59e363c3cd28392da8ec28.jpg]

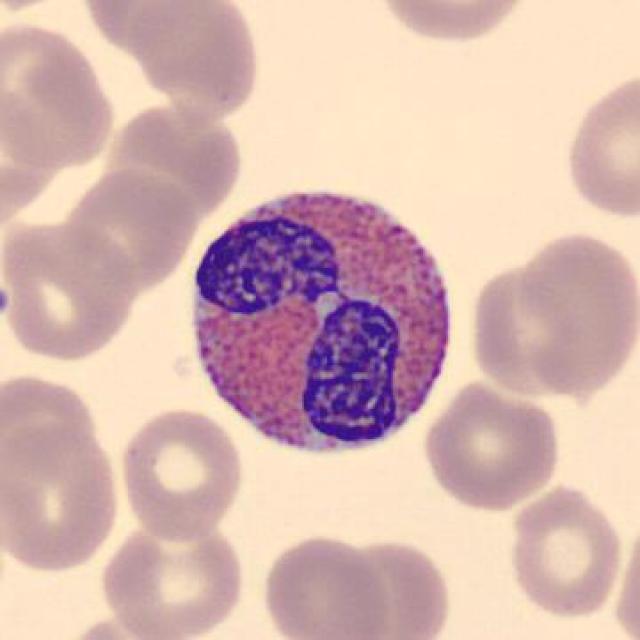

Supplement: Supplementary file 3 — Supplementary Information 3. [file 41598_2025_96918_MOESM3_ESM.zip › WBCs-v2.v2-v2.yolov8/test/images/EO_290013_jpg.rf.1e1e98ef64f378d4376f8e1f322304bb.jpg]

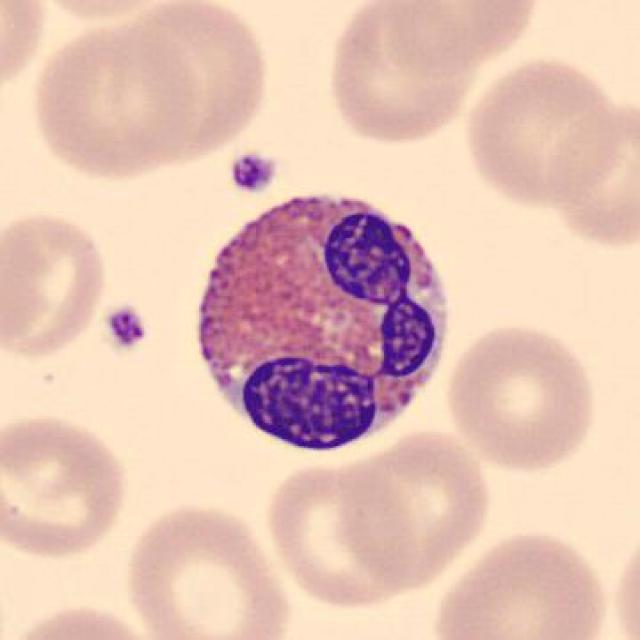

Supplement: Supplementary file 3 — Supplementary Information 3. [file 41598_2025_96918_MOESM3_ESM.zip › WBCs-v2.v2-v2.yolov8/test/images/EO_301146_jpg.rf.312e77883dd4938e0db8e3060ef5e0a1.jpg]

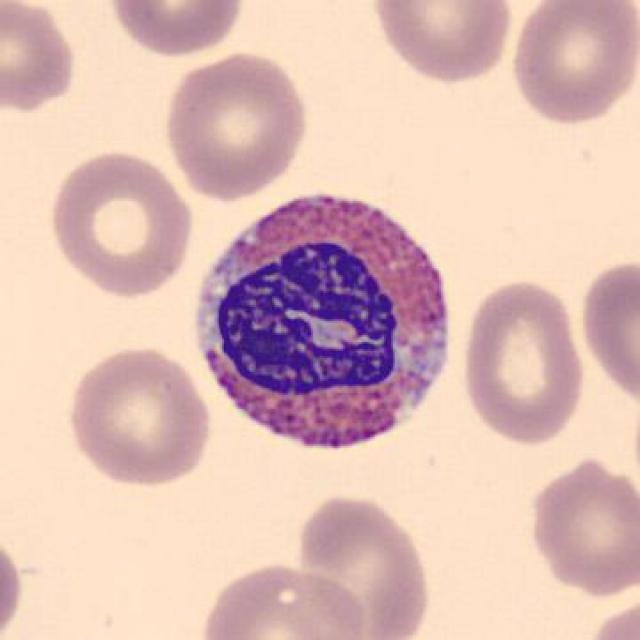

Supplement: Supplementary file 3 — Supplementary Information 3. [file 41598_2025_96918_MOESM3_ESM.zip › WBCs-v2.v2-v2.yolov8/test/images/EO_305691_jpg.rf.49767f6beb93c0e1f05baf0457433319.jpg]

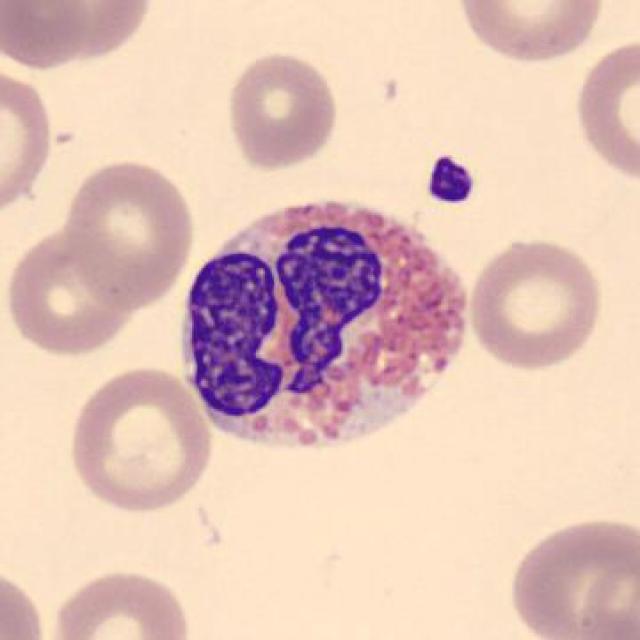

Supplement: Supplementary file 3 — Supplementary Information 3. [file 41598_2025_96918_MOESM3_ESM.zip › WBCs-v2.v2-v2.yolov8/test/images/EO_312103_jpg.rf.95e9b2b4cff038855da3195446b2e4df.jpg]

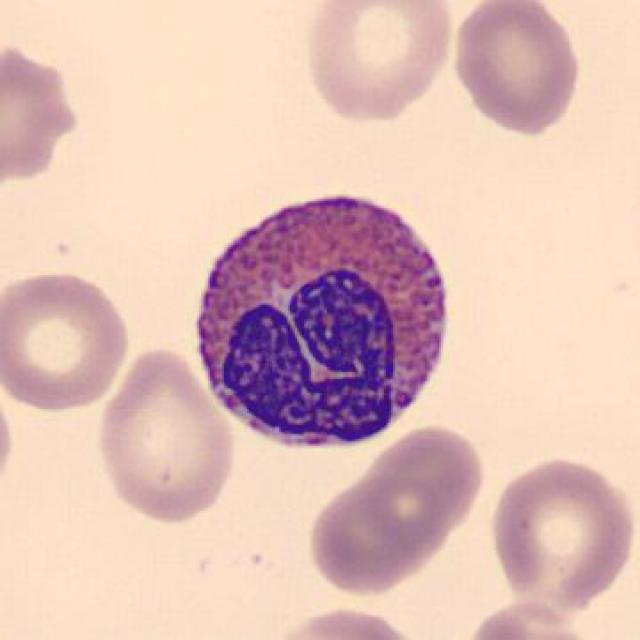

Supplement: Supplementary file 3 — Supplementary Information 3. [file 41598_2025_96918_MOESM3_ESM.zip › WBCs-v2.v2-v2.yolov8/test/images/EO_315883_jpg.rf.ea37c4063980eba7301c7d030cf9a00a.jpg]

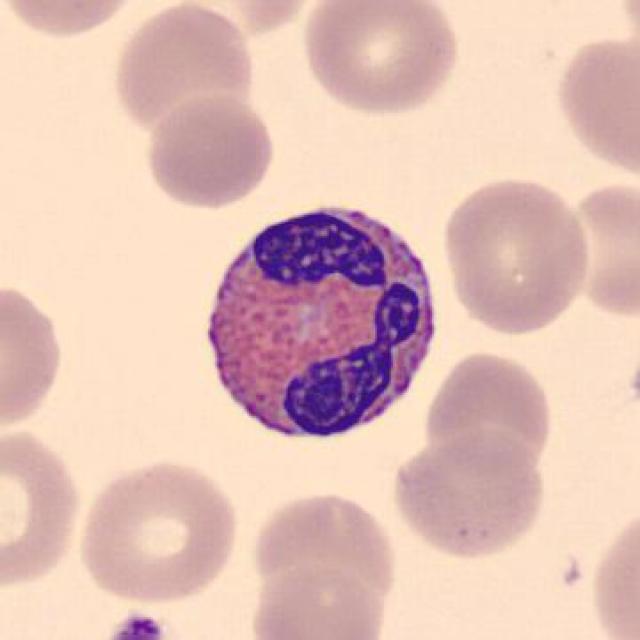

Supplement: Supplementary file 3 — Supplementary Information 3. [file 41598_2025_96918_MOESM3_ESM.zip › WBCs-v2.v2-v2.yolov8/test/images/EO_316082_jpg.rf.05a8c51cec170361839bcb8368d5cc98.jpg]

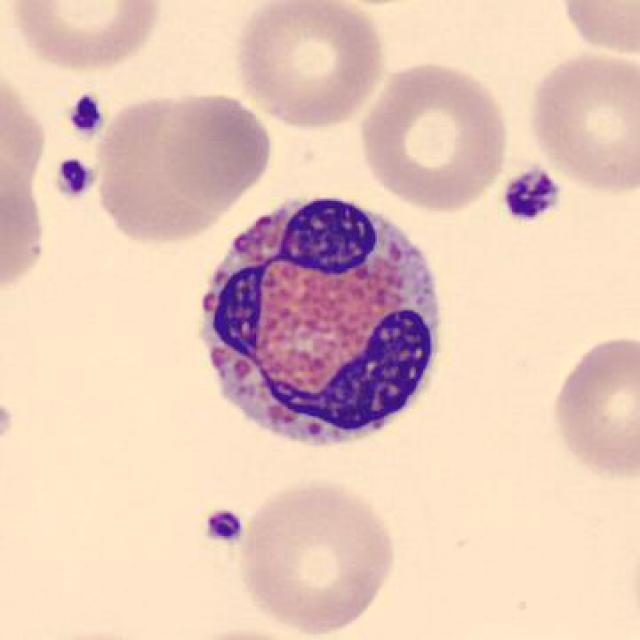

Supplement: Supplementary file 3 — Supplementary Information 3. [file 41598_2025_96918_MOESM3_ESM.zip › WBCs-v2.v2-v2.yolov8/test/images/EO_319771_jpg.rf.b58f7db3f0fe1e5de8ac9534fed878d3.jpg]

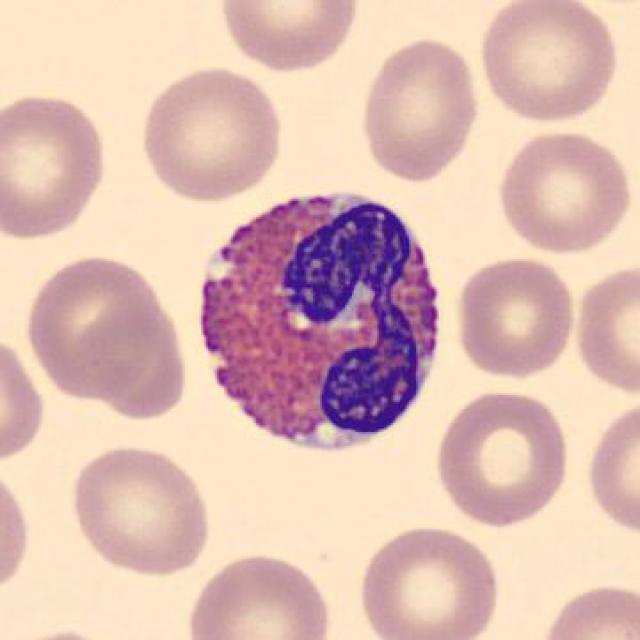

Supplement: Supplementary file 3 — Supplementary Information 3. [file 41598_2025_96918_MOESM3_ESM.zip › WBCs-v2.v2-v2.yolov8/test/images/EO_326816_jpg.rf.2700e9714f76a5fa94973db4c5f4e456.jpg]

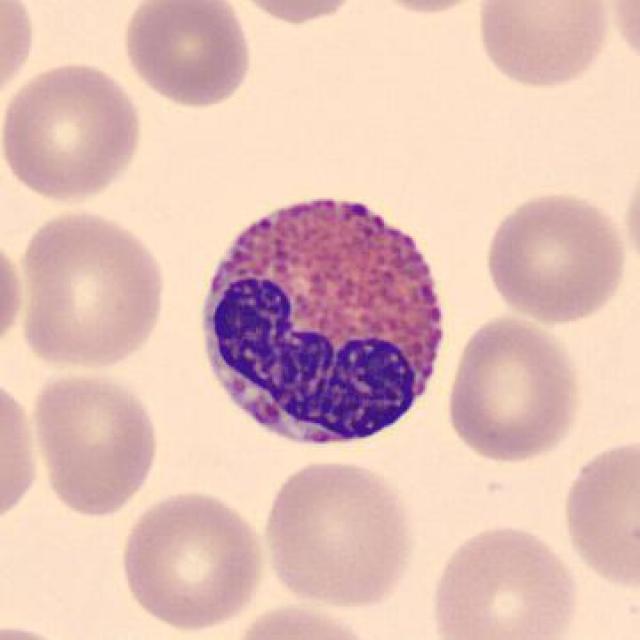

Supplement: Supplementary file 3 — Supplementary Information 3. [file 41598_2025_96918_MOESM3_ESM.zip › WBCs-v2.v2-v2.yolov8/test/images/EO_333532_jpg.rf.261f757a57ee10071cdcd53cade5df3c.jpg]

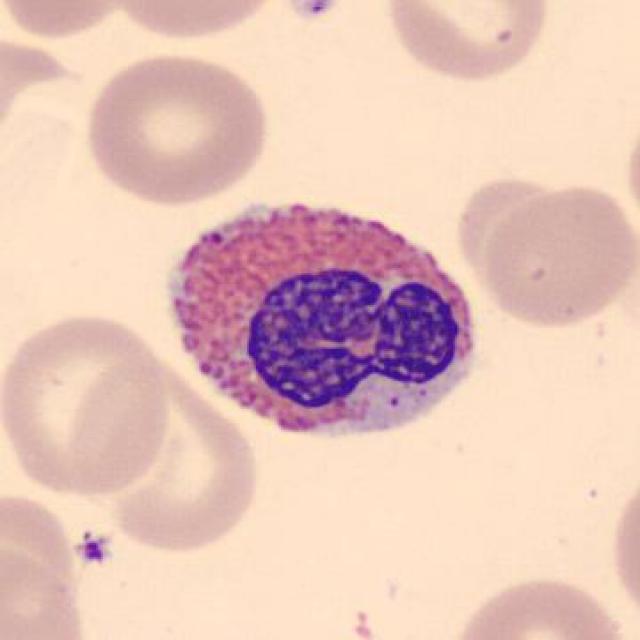

Supplement: Supplementary file 3 — Supplementary Information 3. [file 41598_2025_96918_MOESM3_ESM.zip › WBCs-v2.v2-v2.yolov8/test/images/EO_333712_jpg.rf.f02c5b48dd04c238913f7719dffca6f7.jpg]

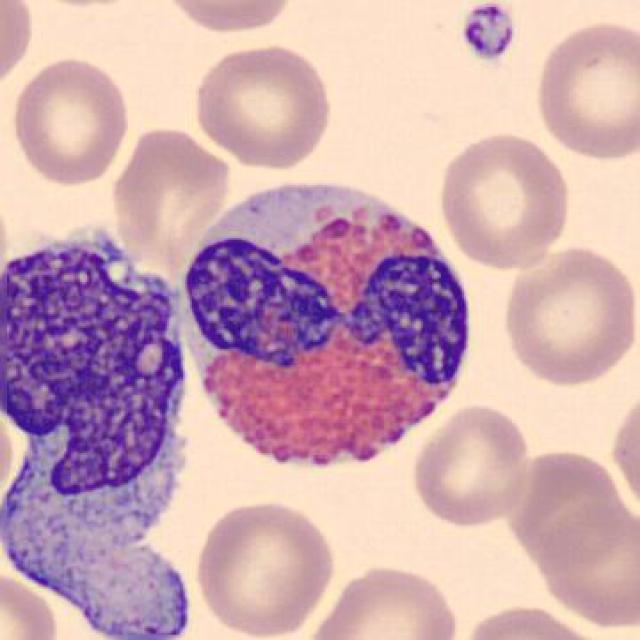

Supplement: Supplementary file 3 — Supplementary Information 3. [file 41598_2025_96918_MOESM3_ESM.zip › WBCs-v2.v2-v2.yolov8/test/images/EO_340997_jpg.rf.8c57f2a5ec0689de553a22231155cb01.jpg]

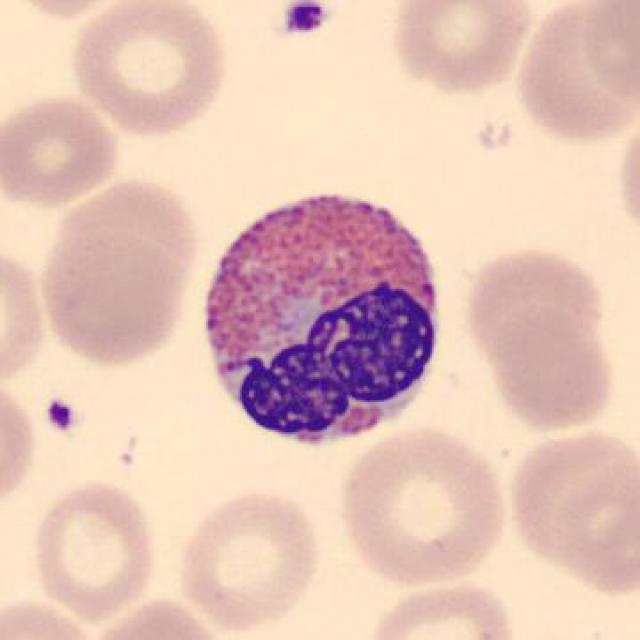

Supplement: Supplementary file 3 — Supplementary Information 3. [file 41598_2025_96918_MOESM3_ESM.zip › WBCs-v2.v2-v2.yolov8/test/images/EO_343324_jpg.rf.634ada10a09513ad525dc32c5571de05.jpg]
