# Supplementary figures and images for: Advanced leukocyte classification using attention mechanisms and dual channel U-Net architecture (part 1 of 4)
Source: Sci Rep. 2025 Apr 22;15:13825. doi: 10.1038/s41598-025-96918-3 (PMC12015285; doi:10.1038/s41598-025-96918-3)

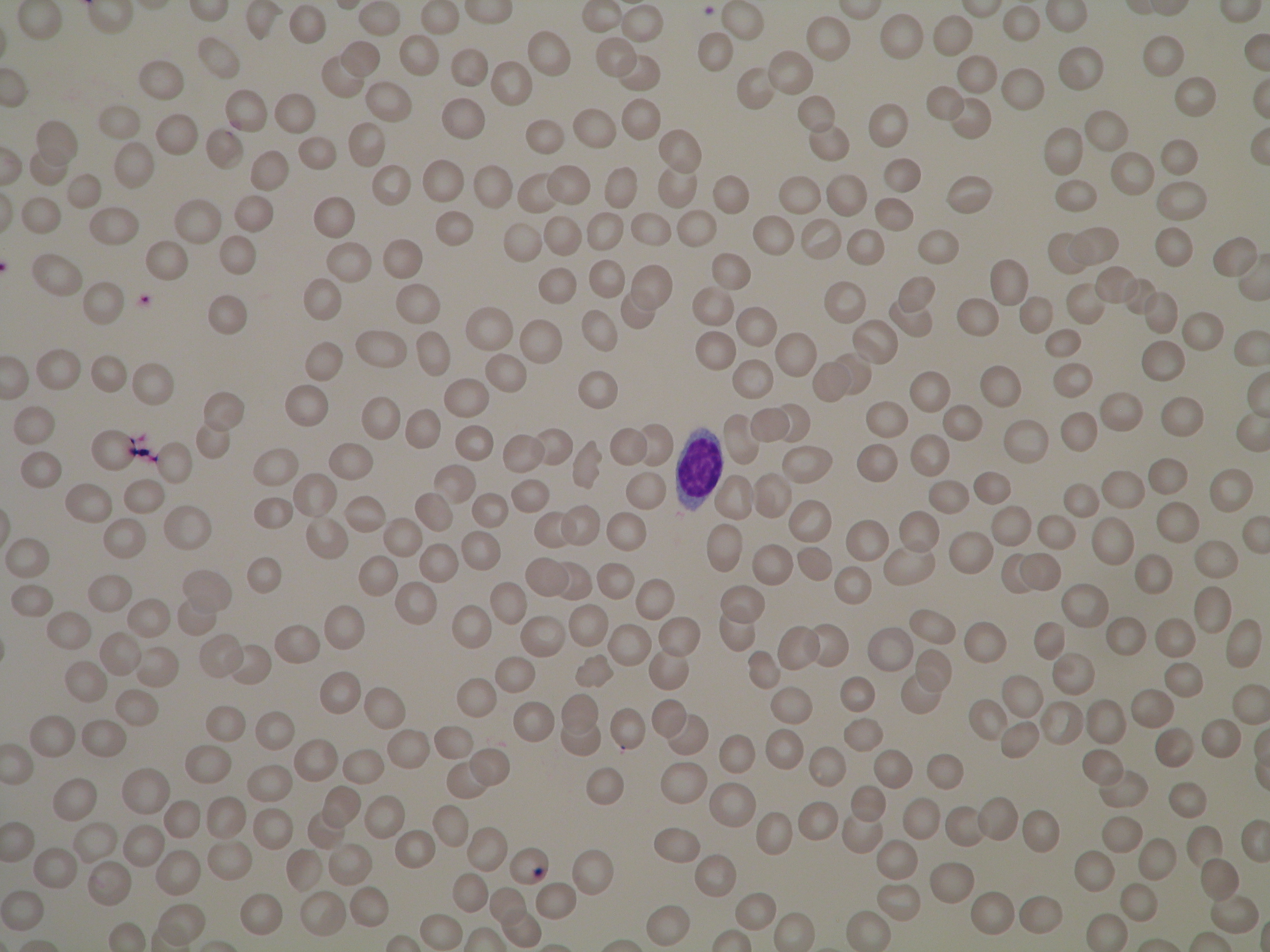

Supplement: Supplementary file 1 — Supplementary Information 1. [file 41598_2025_96918_MOESM1_ESM.zip › ALL_IDB Dataset/L1/Im064_0.jpg]

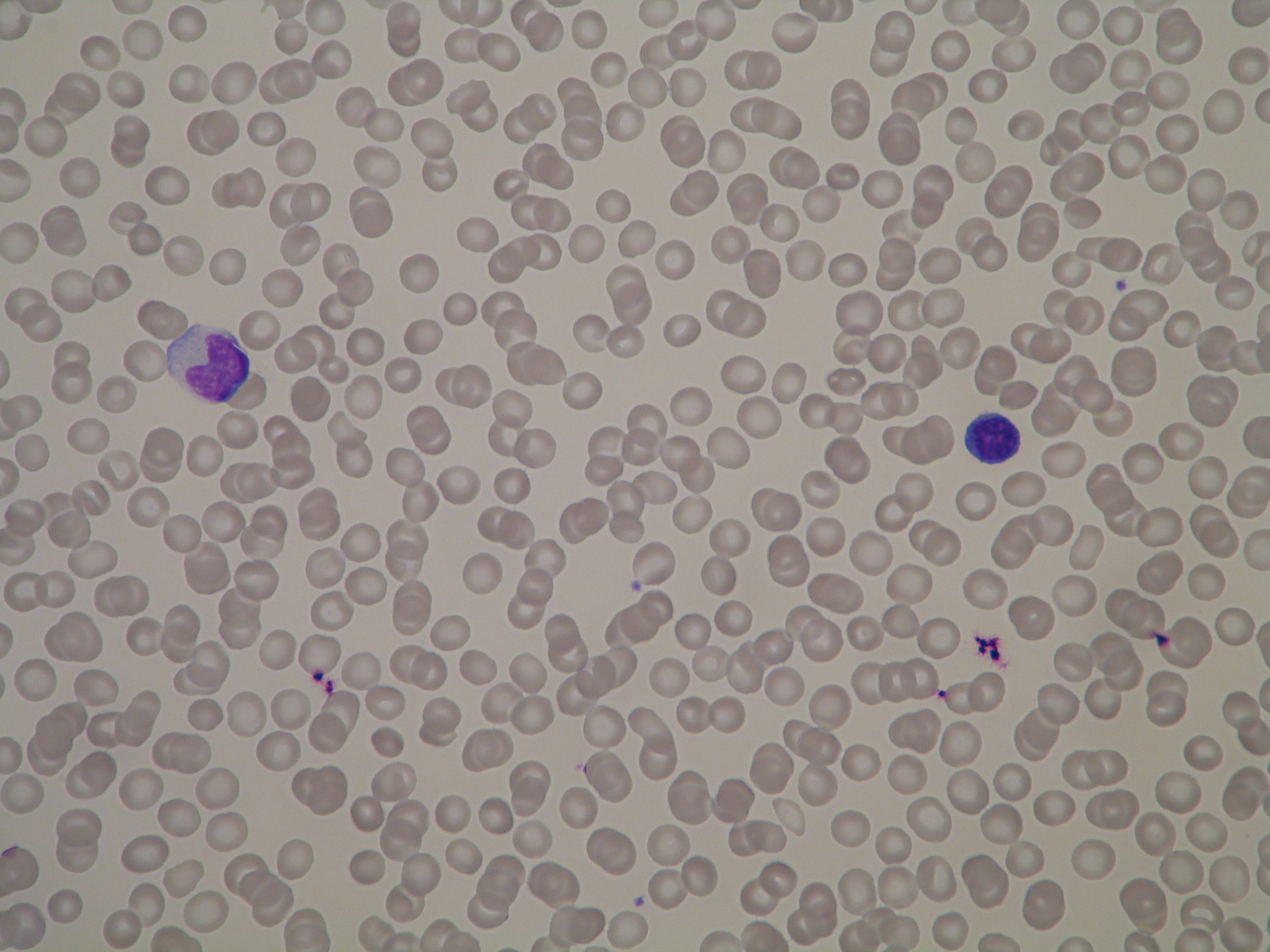

Supplement: Supplementary file 1 — Supplementary Information 1. [file 41598_2025_96918_MOESM1_ESM.zip › ALL_IDB Dataset/L1/Im065_0.jpg]

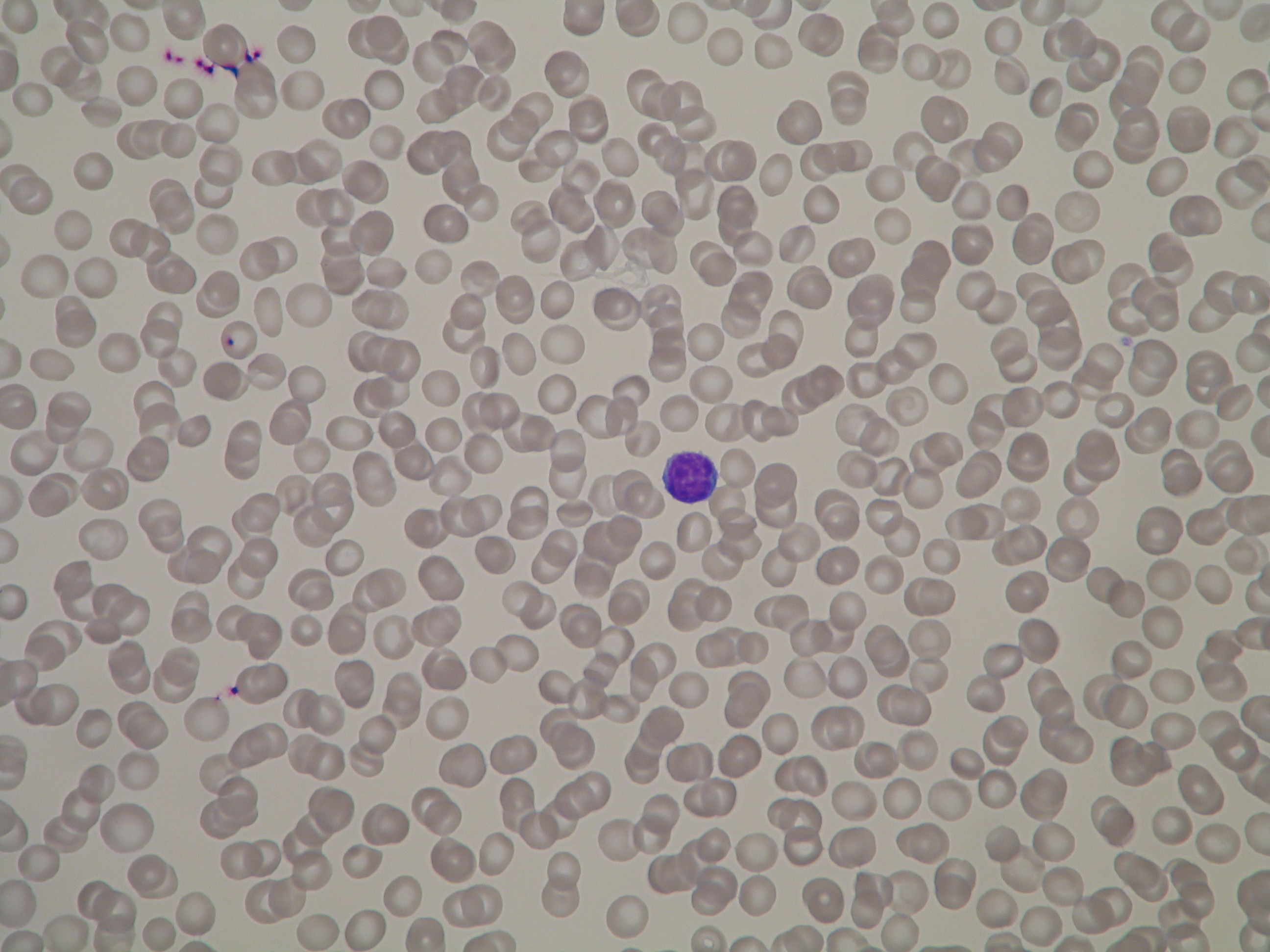

Supplement: Supplementary file 1 — Supplementary Information 1. [file 41598_2025_96918_MOESM1_ESM.zip › ALL_IDB Dataset/L1/Im066_0.jpg]

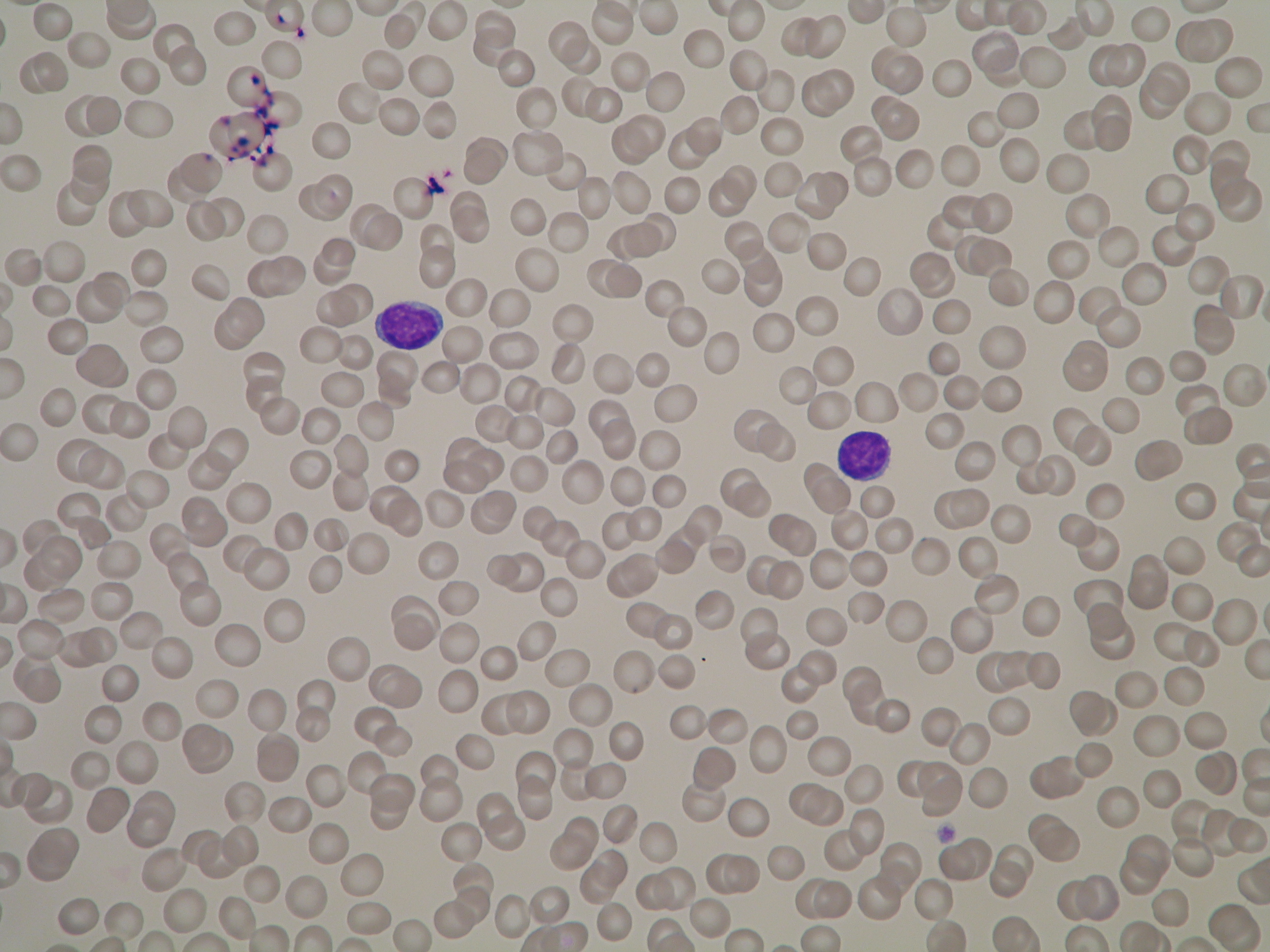

Supplement: Supplementary file 1 — Supplementary Information 1. [file 41598_2025_96918_MOESM1_ESM.zip › ALL_IDB Dataset/L1/Im0671_0.jpg]

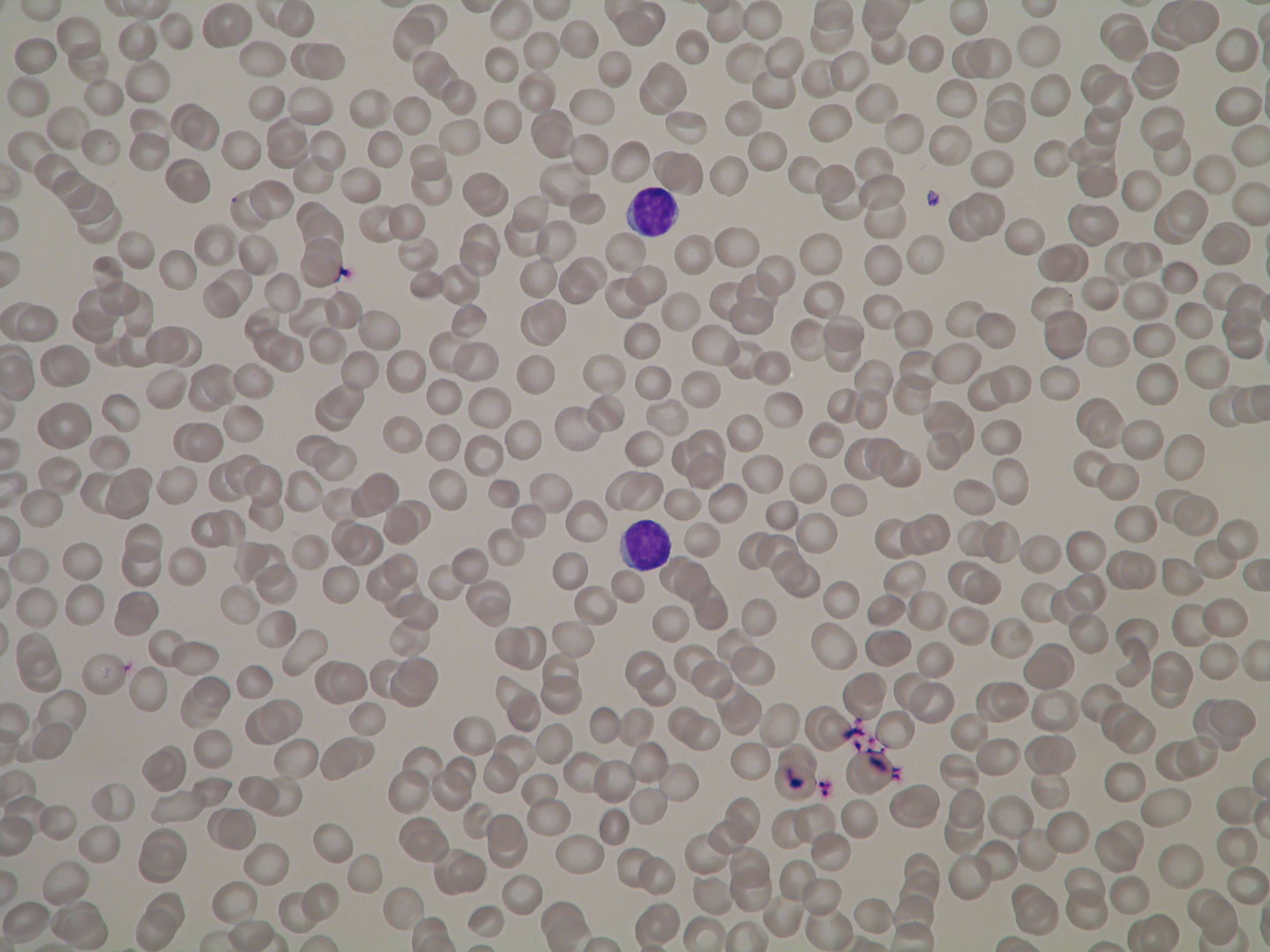

Supplement: Supplementary file 1 — Supplementary Information 1. [file 41598_2025_96918_MOESM1_ESM.zip › ALL_IDB Dataset/L1/Im068_0.jpg]

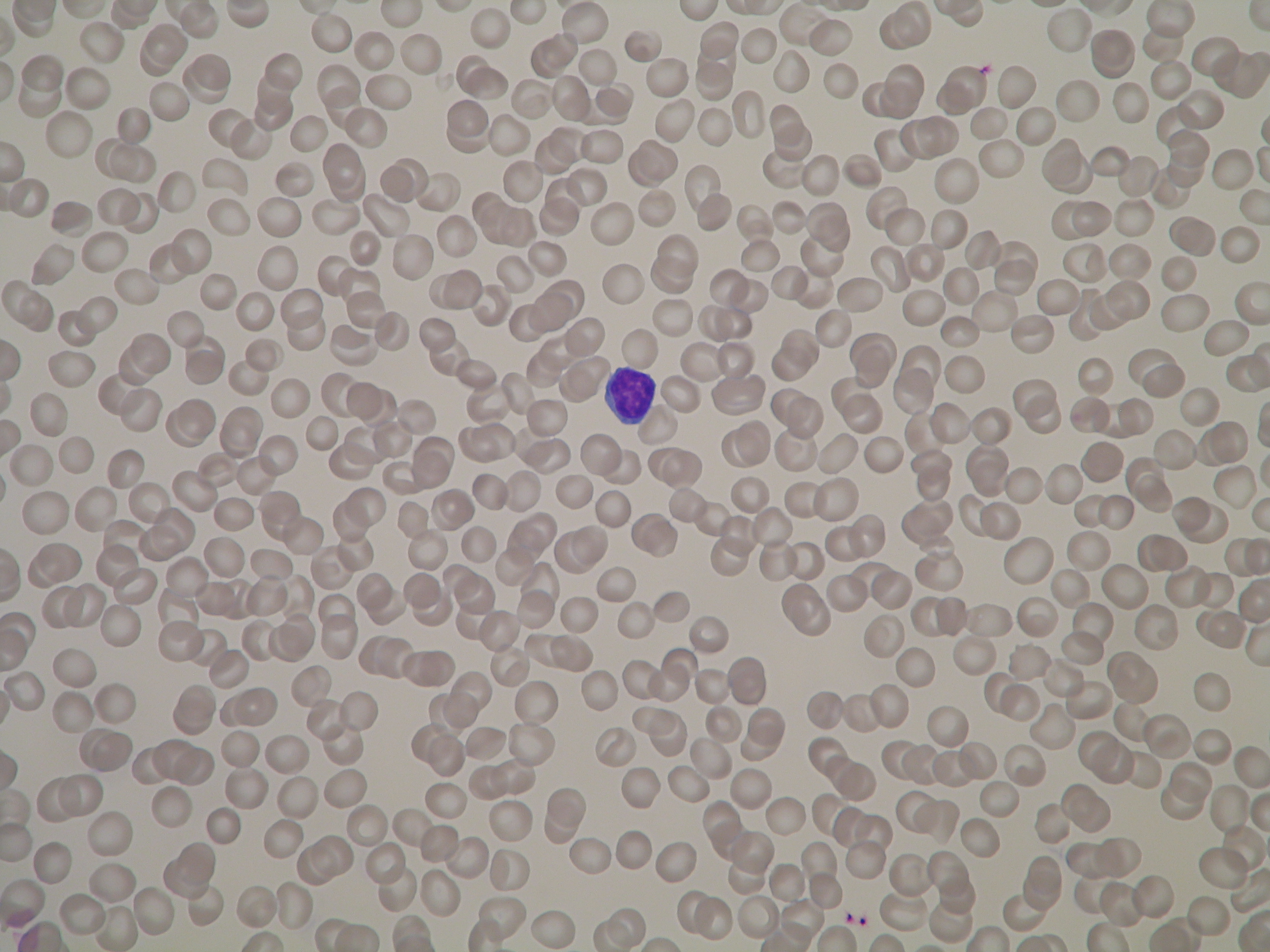

Supplement: Supplementary file 1 — Supplementary Information 1. [file 41598_2025_96918_MOESM1_ESM.zip › ALL_IDB Dataset/L1/Im069_0.jpg]

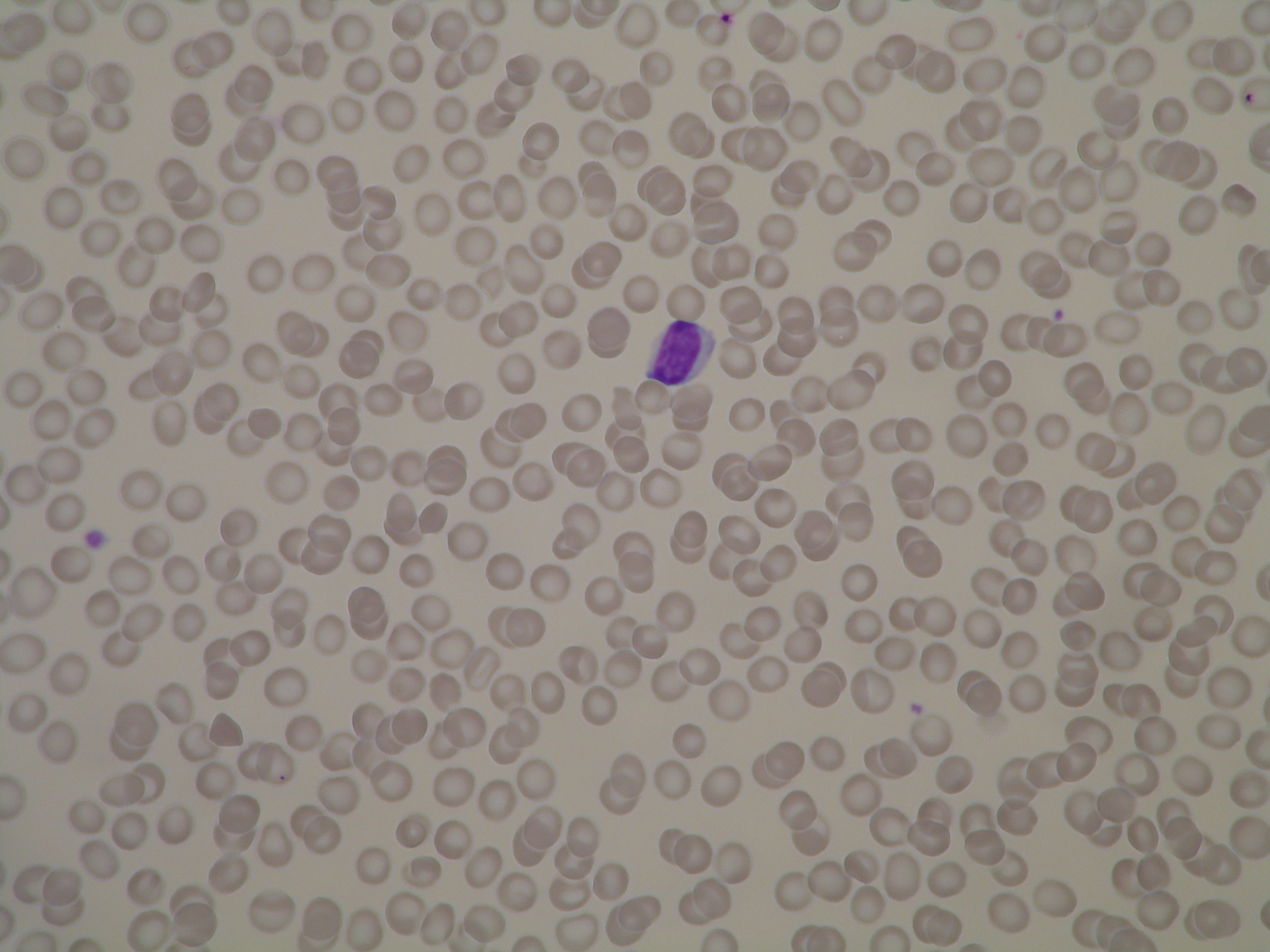

Supplement: Supplementary file 1 — Supplementary Information 1. [file 41598_2025_96918_MOESM1_ESM.zip › ALL_IDB Dataset/L1/Im070_0.jpg]

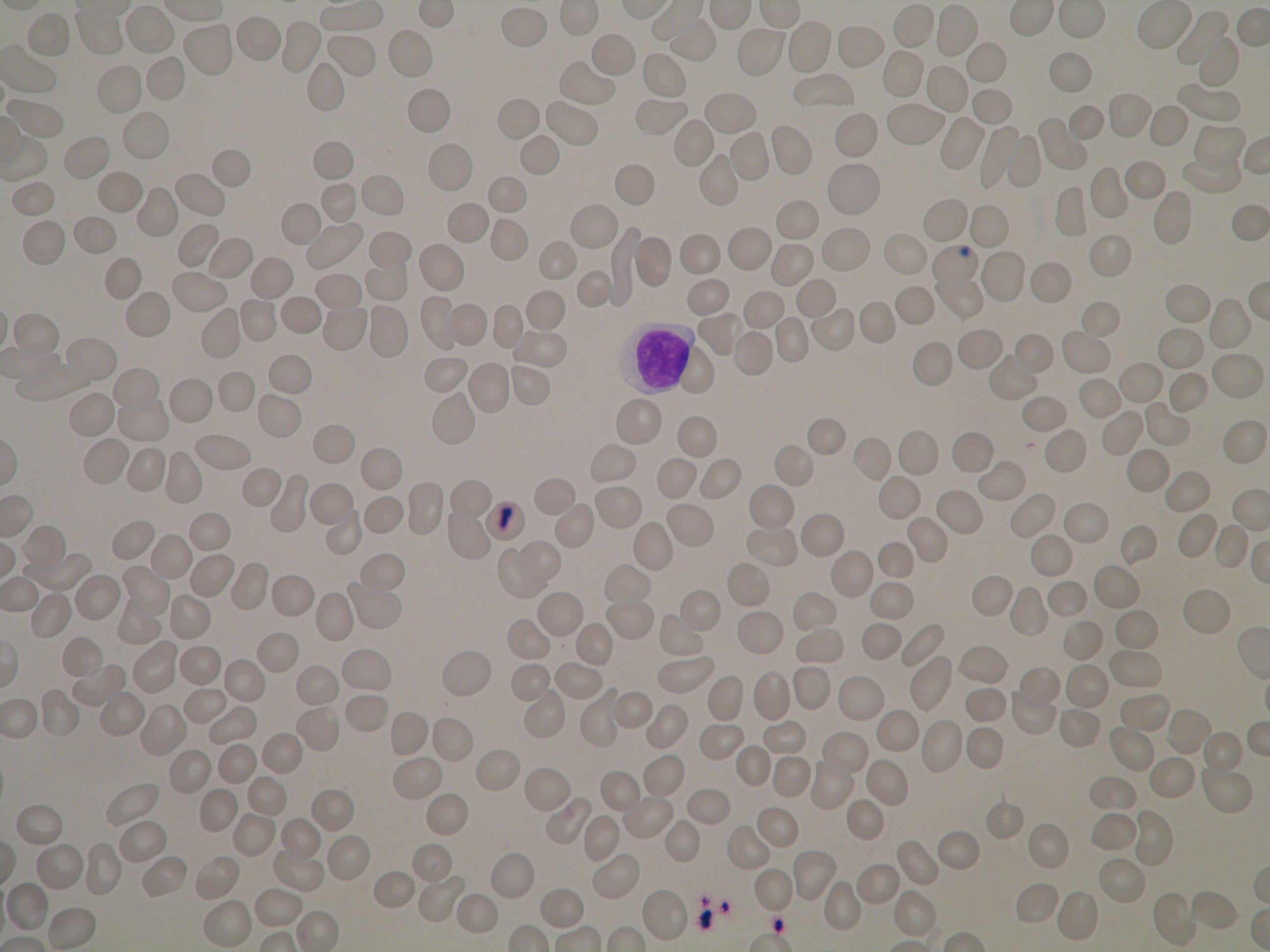

Supplement: Supplementary file 1 — Supplementary Information 1. [file 41598_2025_96918_MOESM1_ESM.zip › ALL_IDB Dataset/L1/Im071_0.jpg]

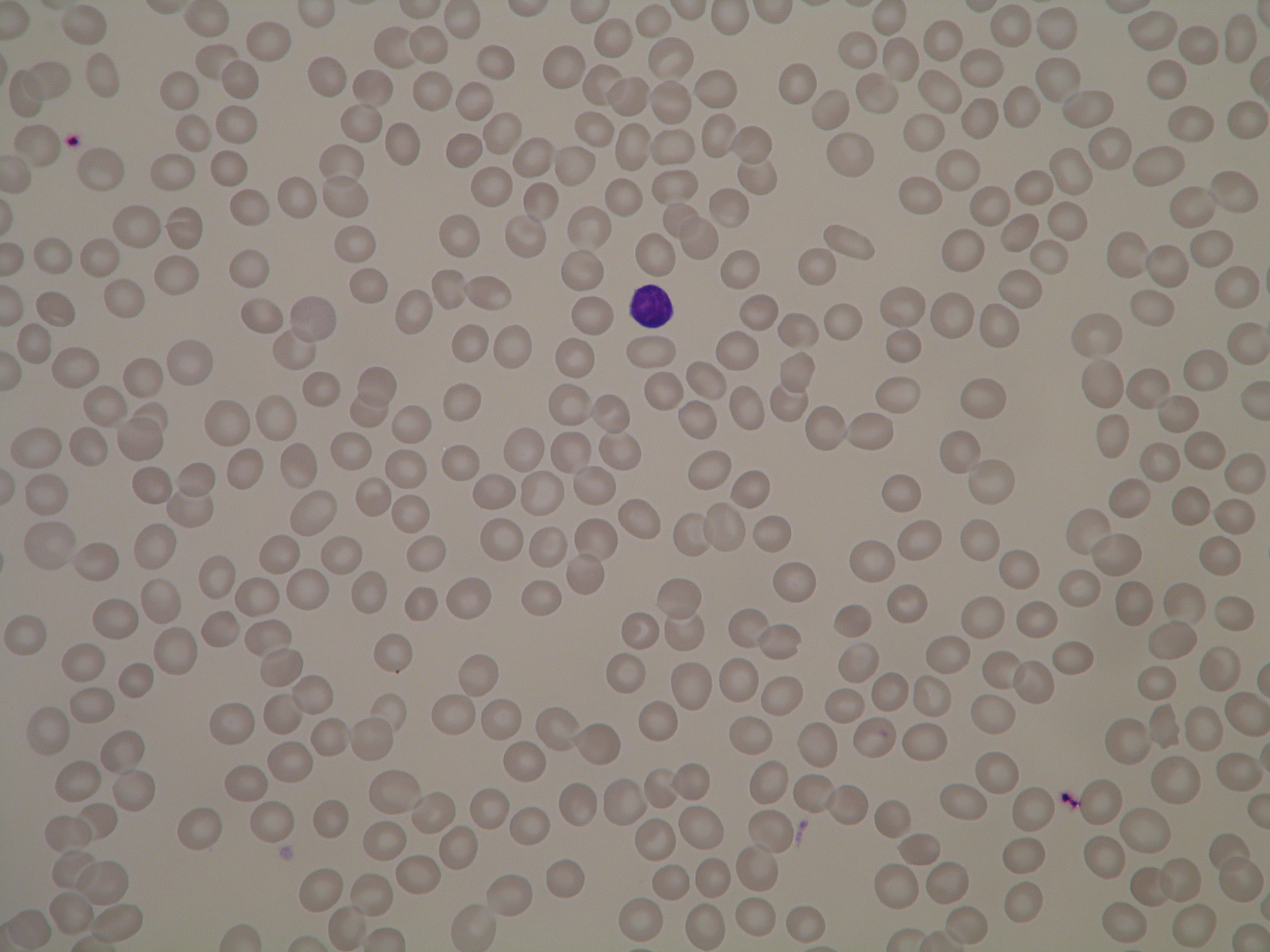

Supplement: Supplementary file 1 — Supplementary Information 1. [file 41598_2025_96918_MOESM1_ESM.zip › ALL_IDB Dataset/L1/Im072_0.jpg]

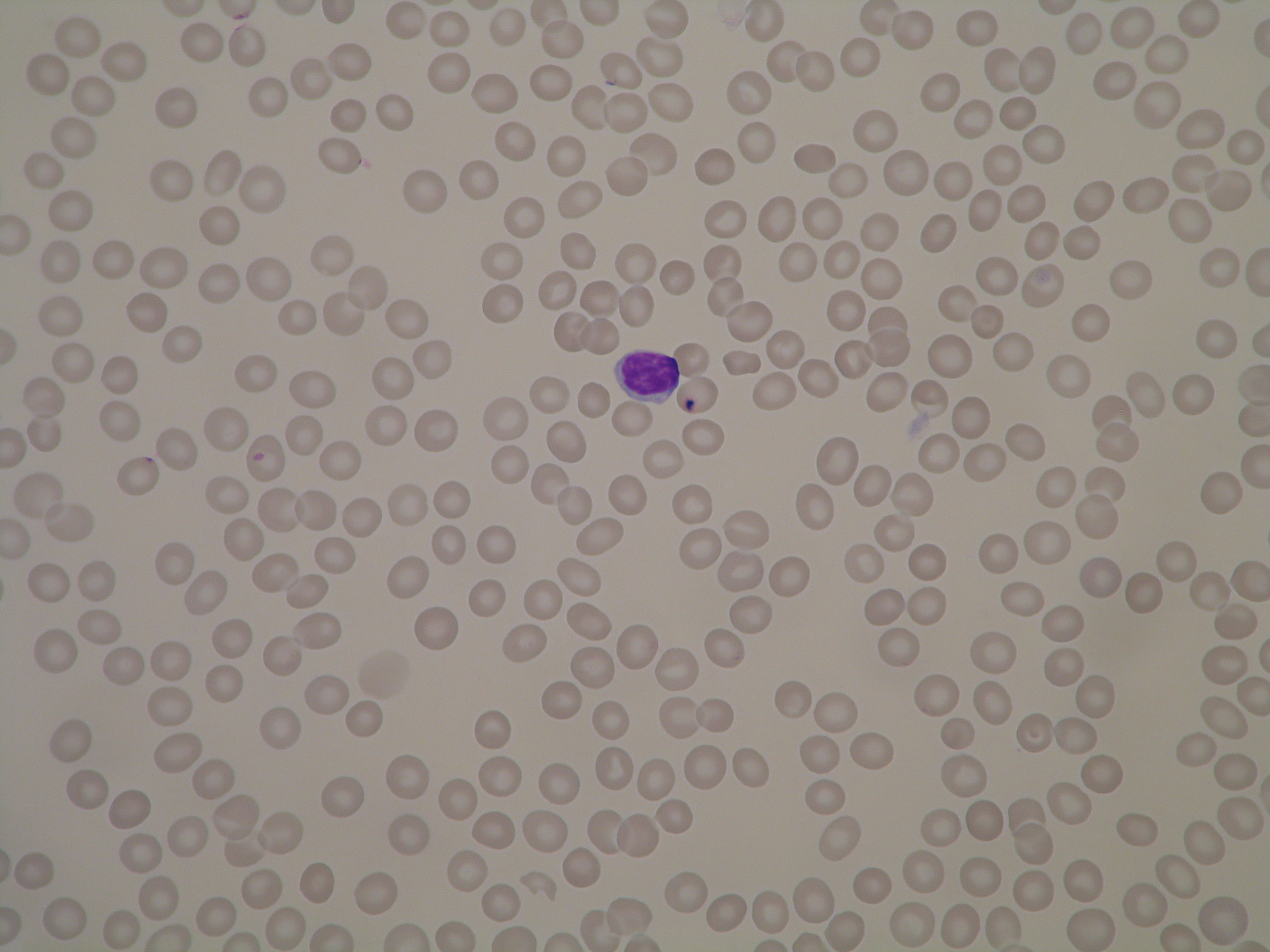

Supplement: Supplementary file 1 — Supplementary Information 1. [file 41598_2025_96918_MOESM1_ESM.zip › ALL_IDB Dataset/L1/Im073_0.jpg]

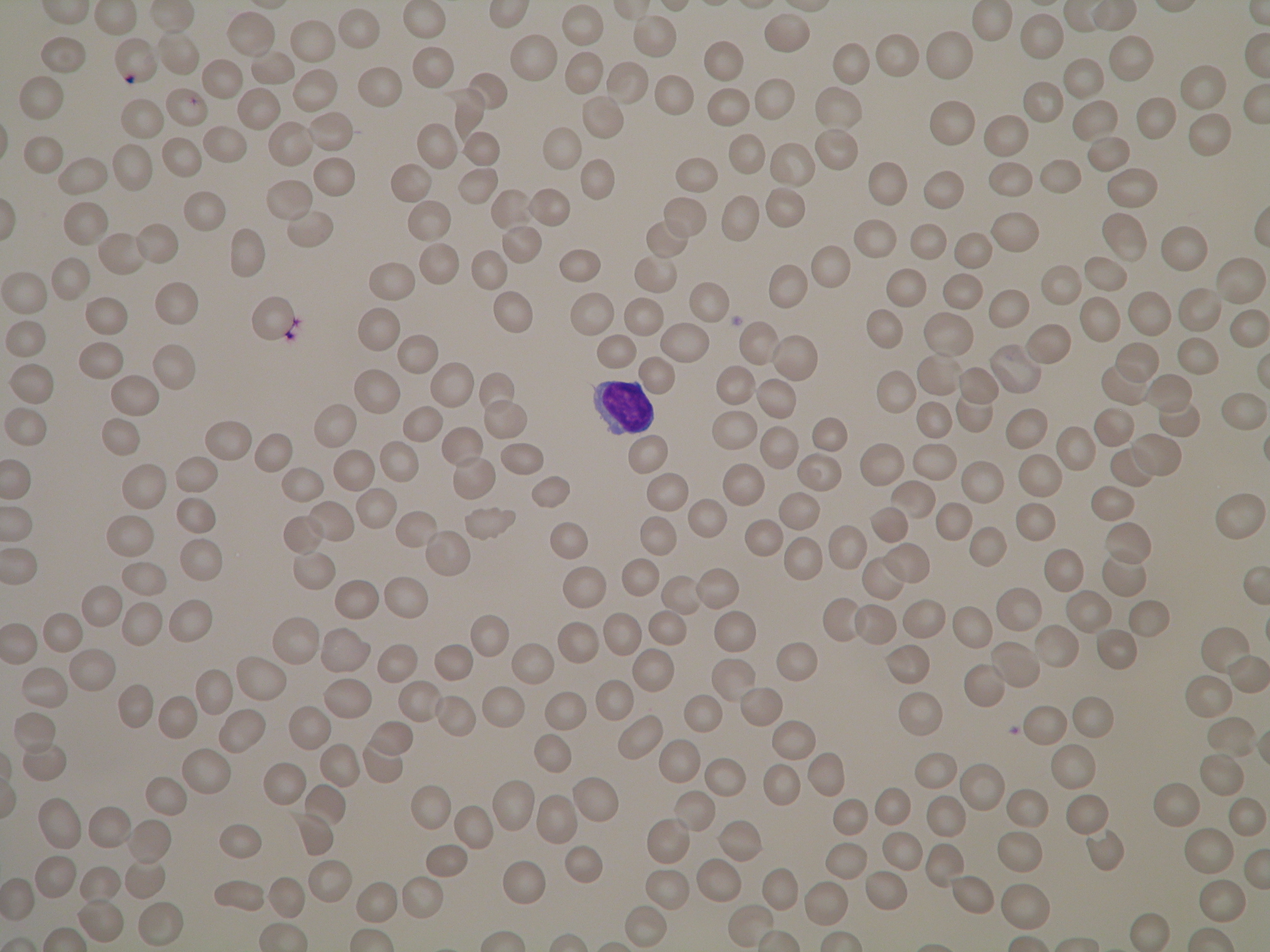

Supplement: Supplementary file 1 — Supplementary Information 1. [file 41598_2025_96918_MOESM1_ESM.zip › ALL_IDB Dataset/L1/Im074_0.jpg]

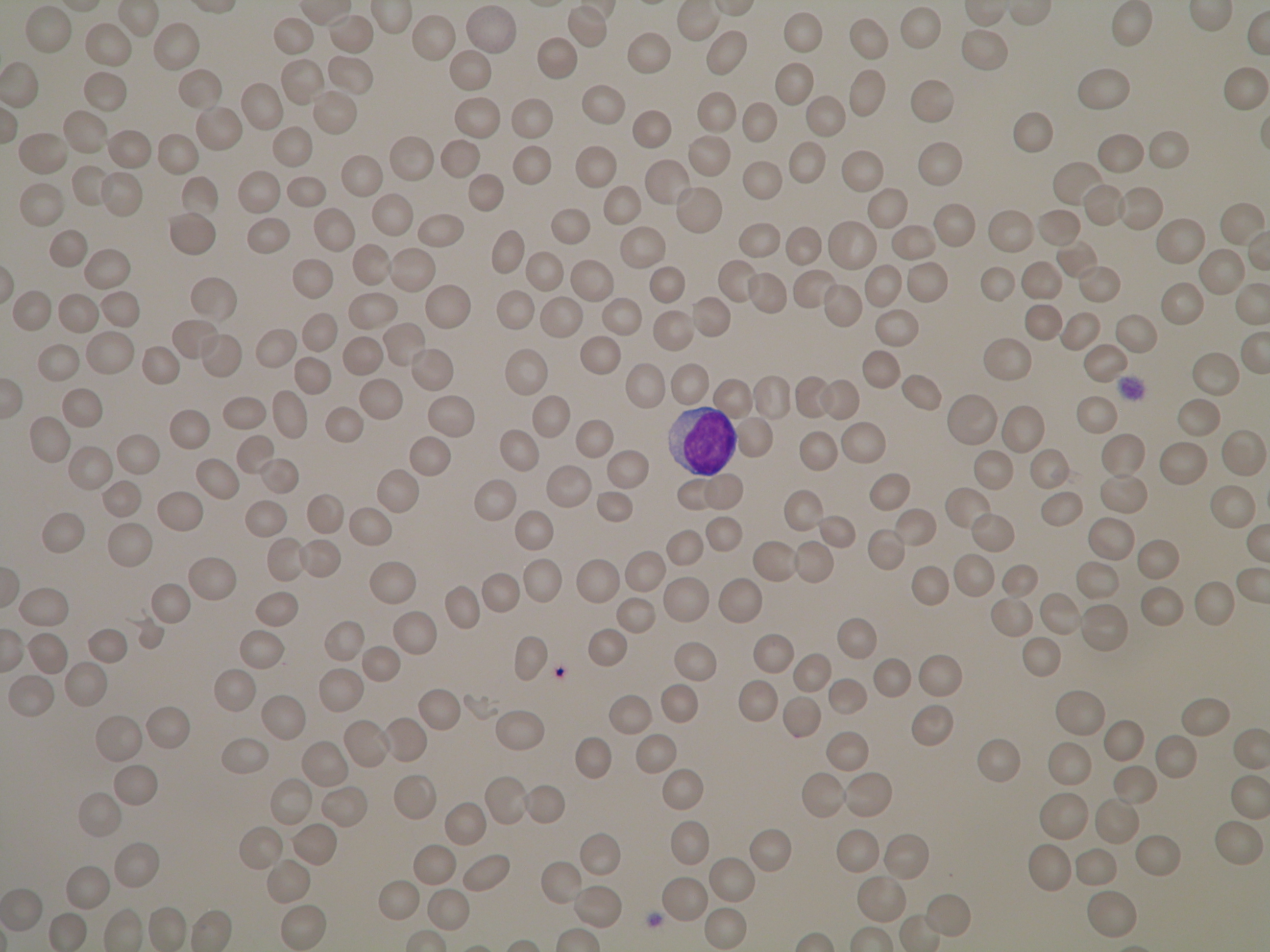

Supplement: Supplementary file 1 — Supplementary Information 1. [file 41598_2025_96918_MOESM1_ESM.zip › ALL_IDB Dataset/L1/Im075_0.jpg]

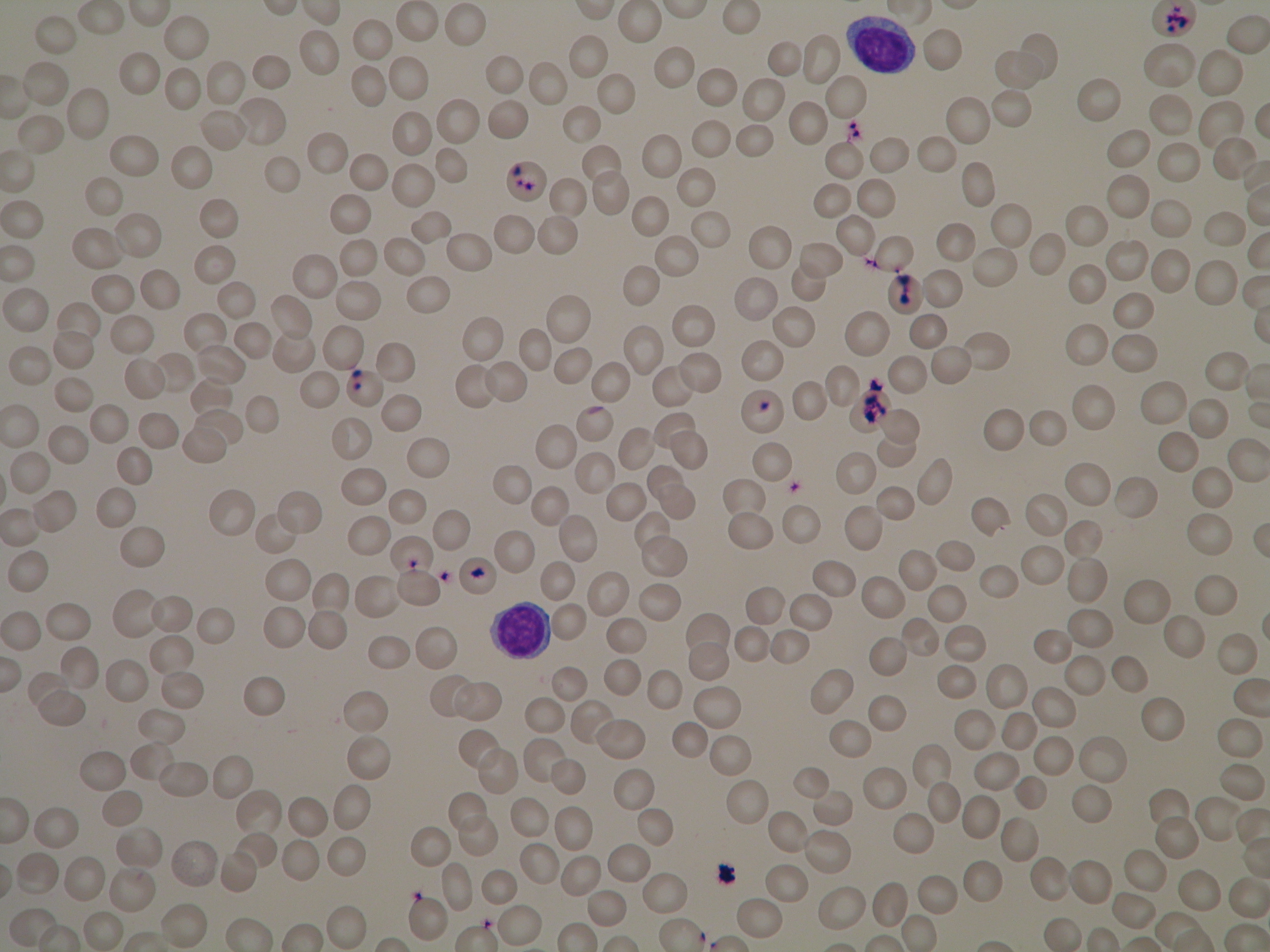

Supplement: Supplementary file 1 — Supplementary Information 1. [file 41598_2025_96918_MOESM1_ESM.zip › ALL_IDB Dataset/L1/Im076_0.jpg]

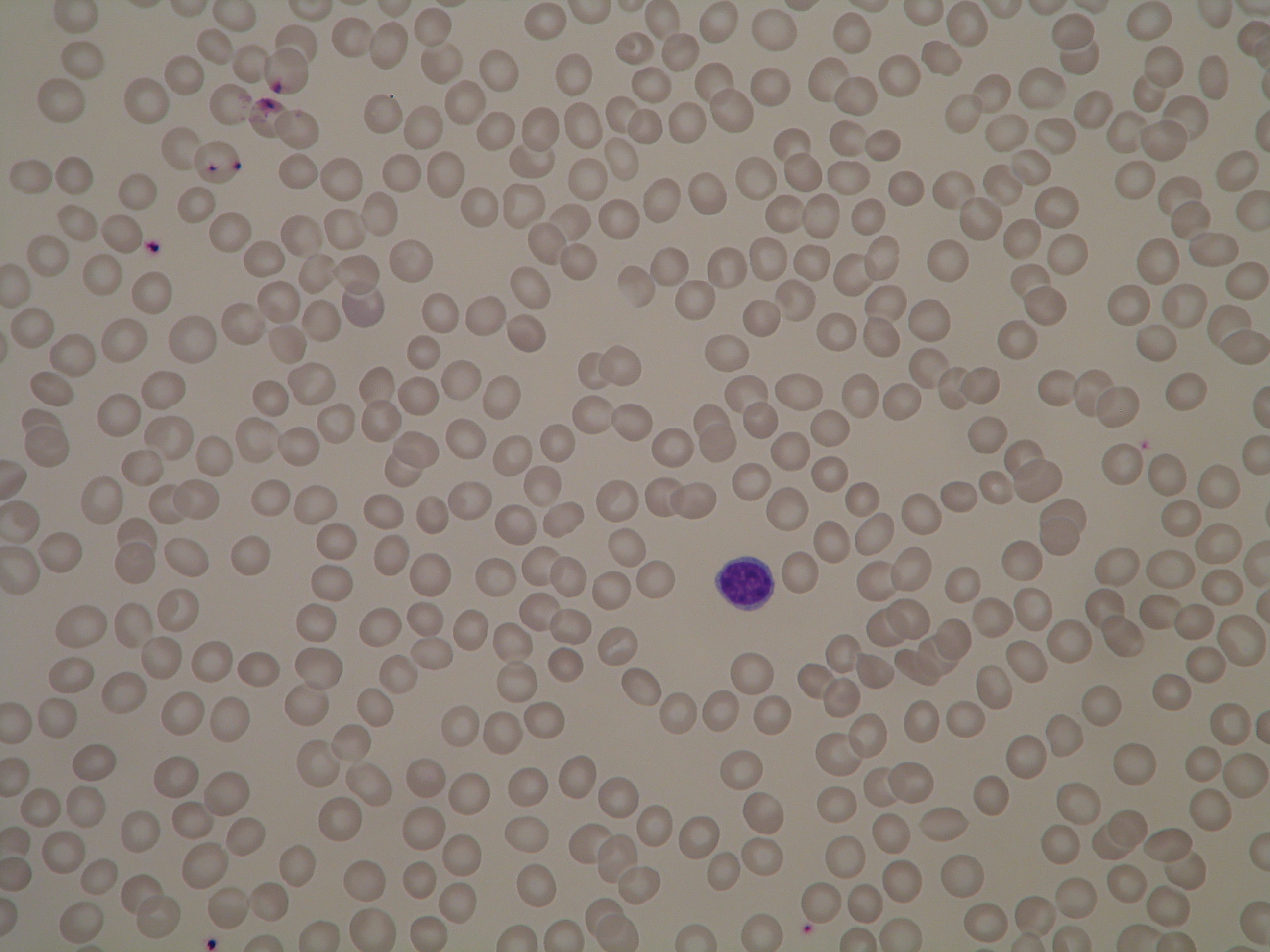

Supplement: Supplementary file 1 — Supplementary Information 1. [file 41598_2025_96918_MOESM1_ESM.zip › ALL_IDB Dataset/L1/Im077_0.jpg]

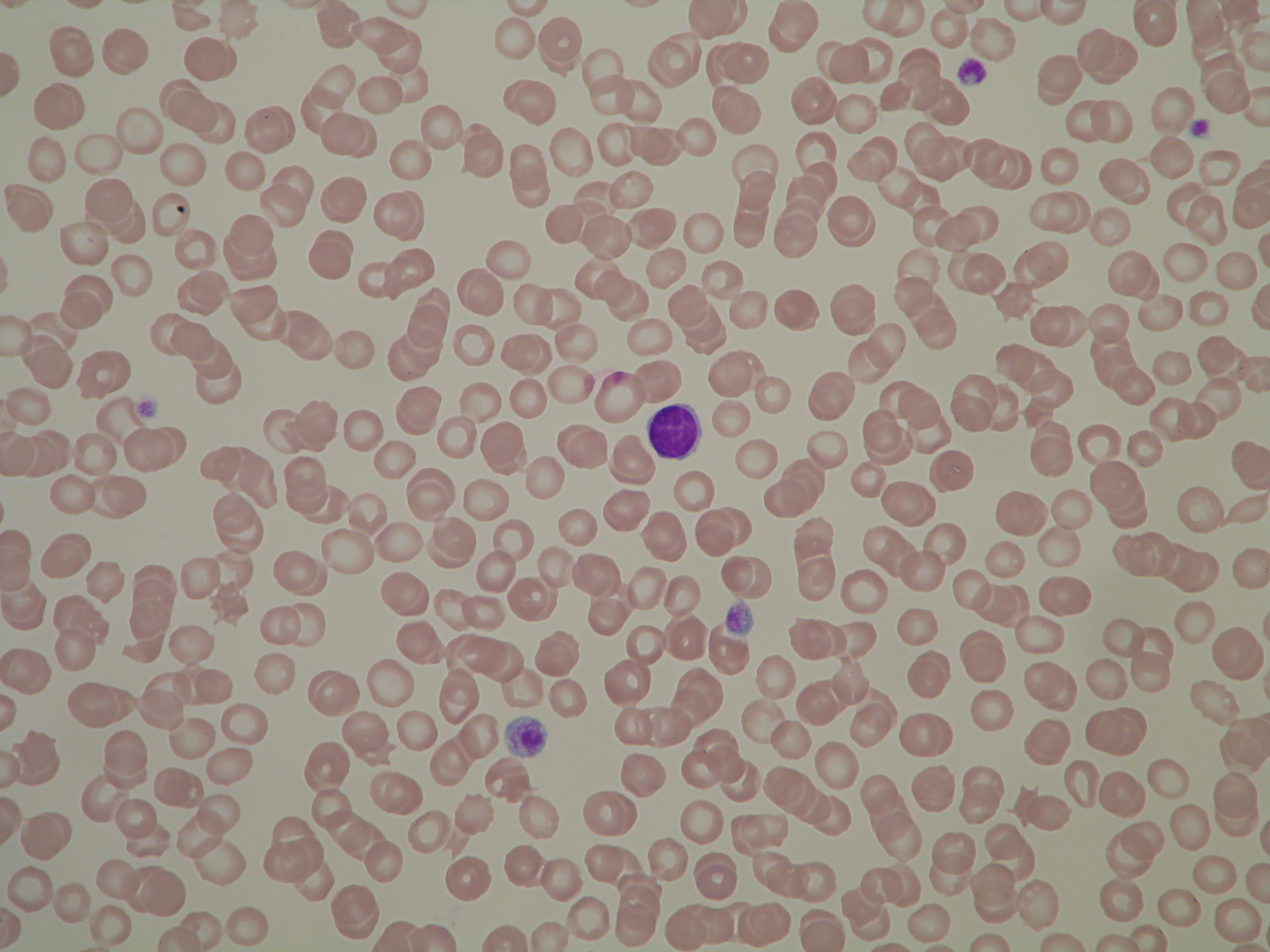

Supplement: Supplementary file 1 — Supplementary Information 1. [file 41598_2025_96918_MOESM1_ESM.zip › ALL_IDB Dataset/L1/Im078_0.jpg]

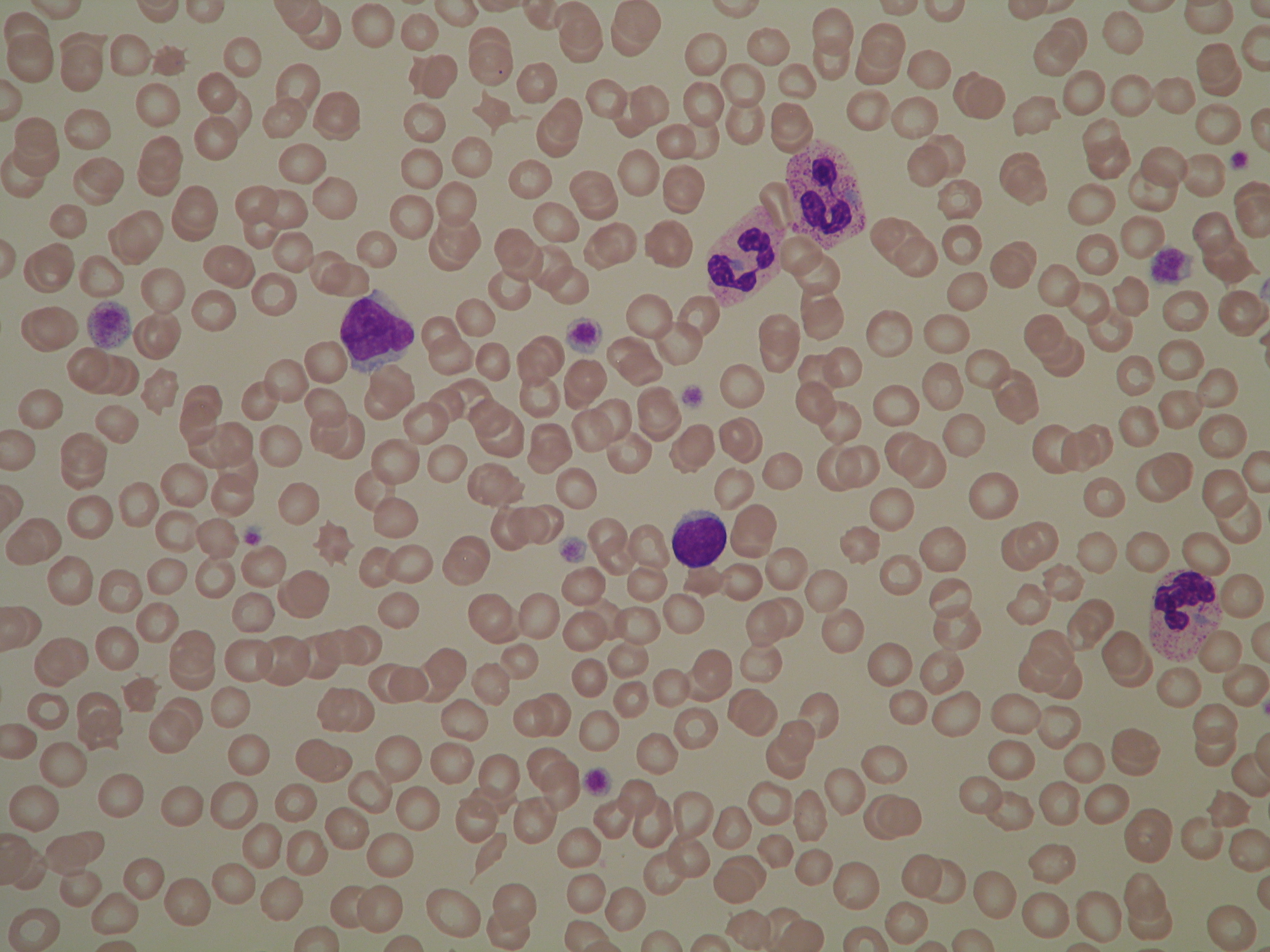

Supplement: Supplementary file 1 — Supplementary Information 1. [file 41598_2025_96918_MOESM1_ESM.zip › ALL_IDB Dataset/L1/Im079_0.jpg]

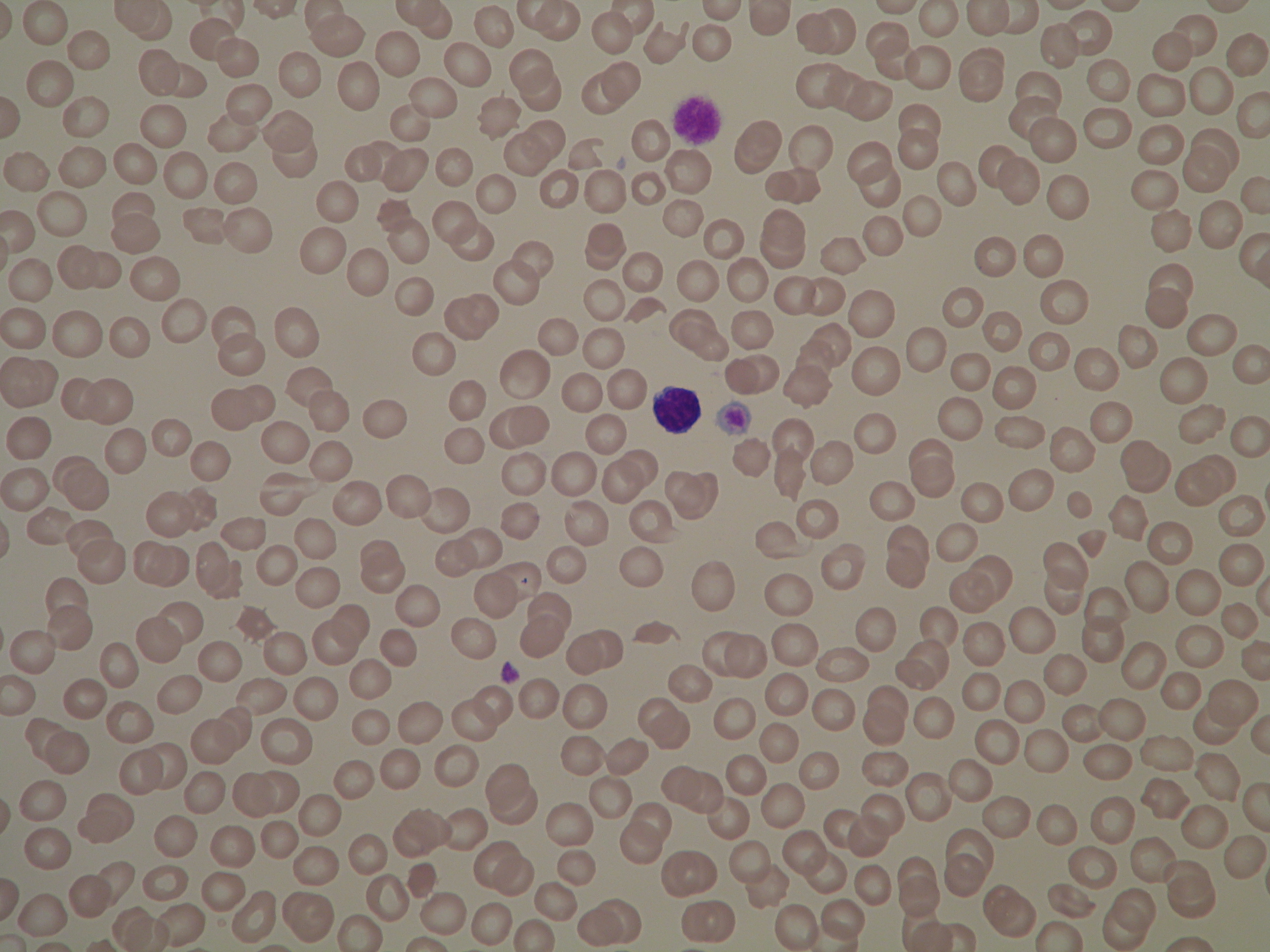

Supplement: Supplementary file 1 — Supplementary Information 1. [file 41598_2025_96918_MOESM1_ESM.zip › ALL_IDB Dataset/L1/Im0801_0.jpg]

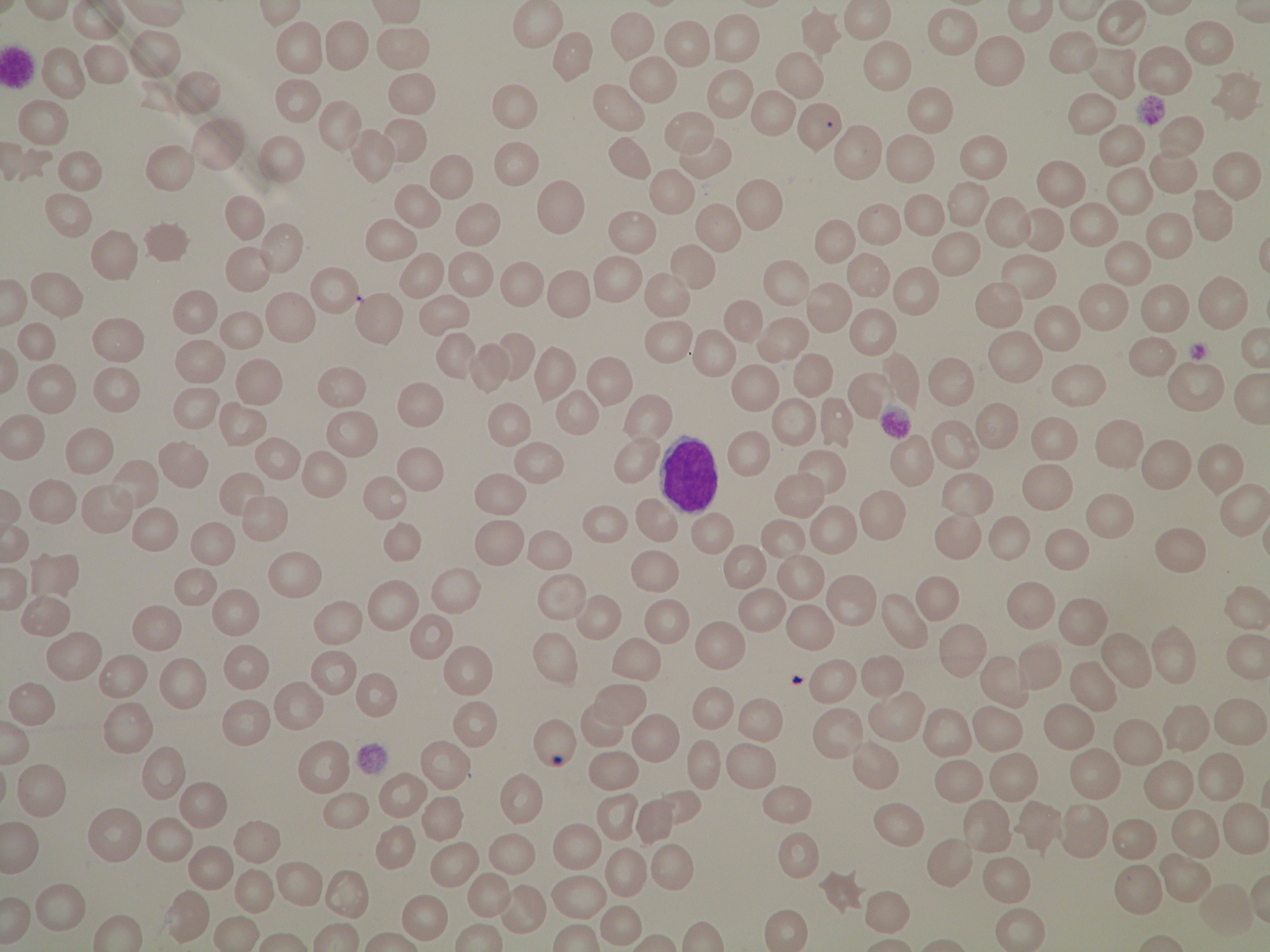

Supplement: Supplementary file 1 — Supplementary Information 1. [file 41598_2025_96918_MOESM1_ESM.zip › ALL_IDB Dataset/L1/Im0811_0.jpg]

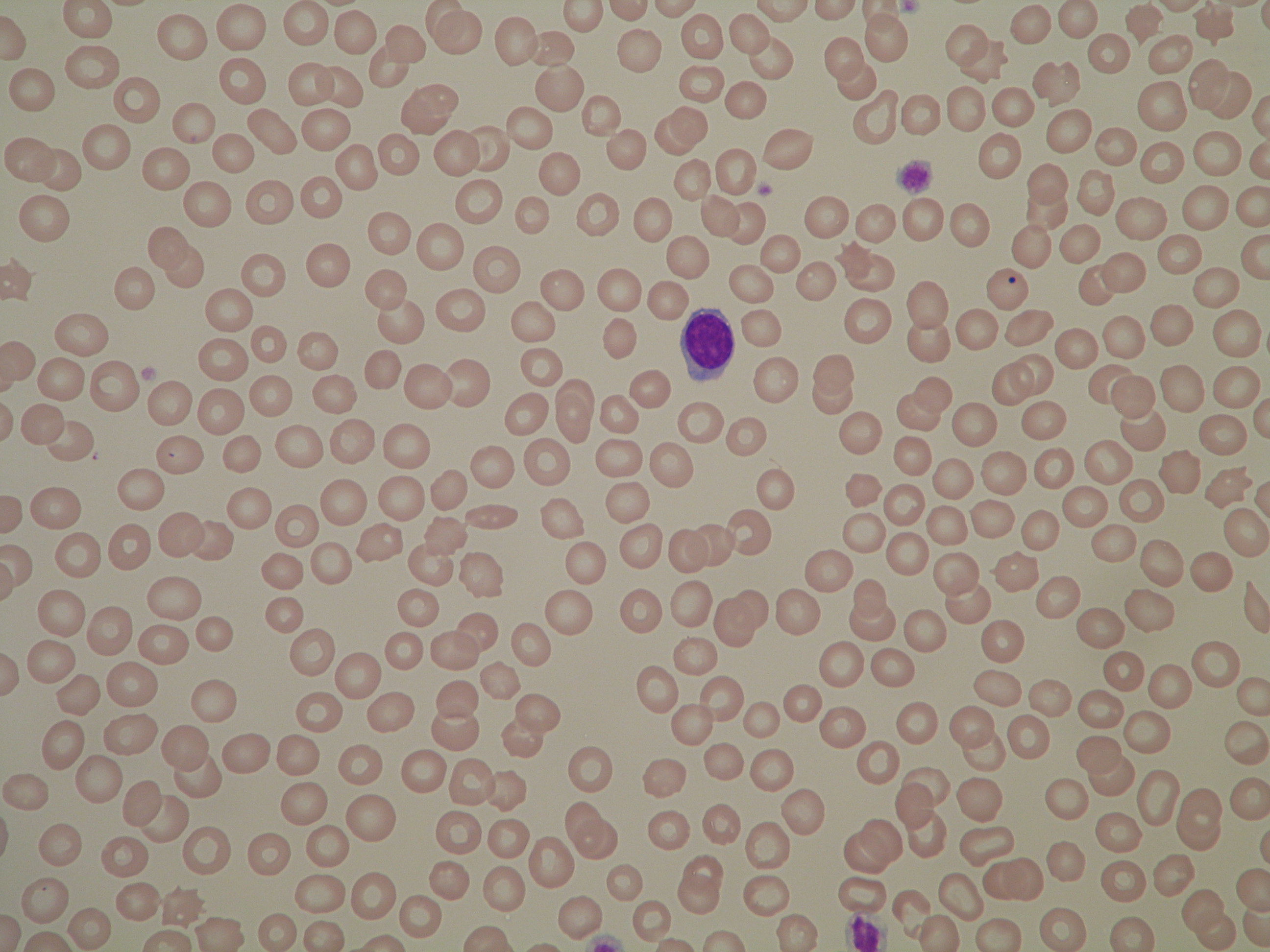

Supplement: Supplementary file 1 — Supplementary Information 1. [file 41598_2025_96918_MOESM1_ESM.zip › ALL_IDB Dataset/L1/Im082_0.jpg]

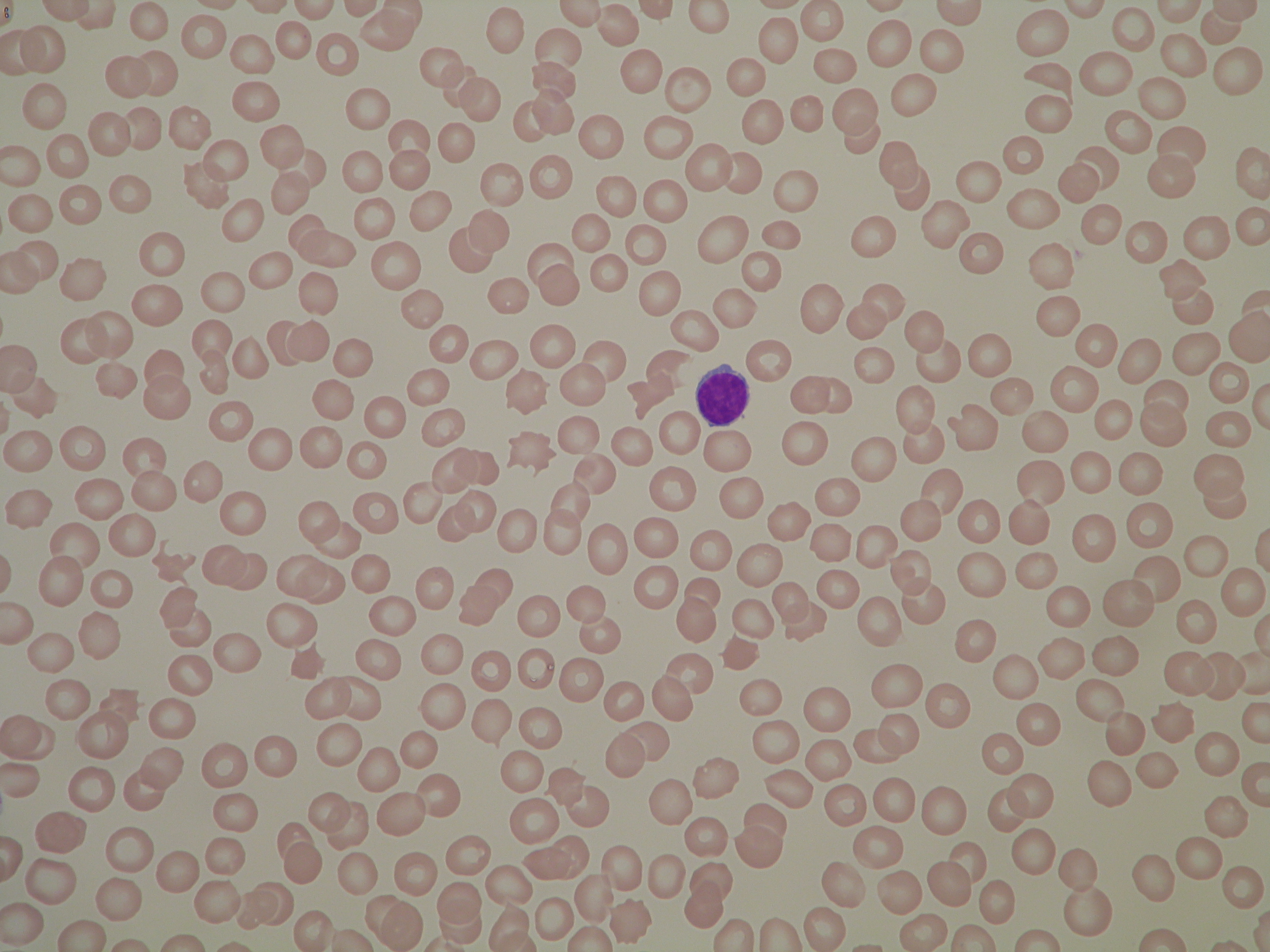

Supplement: Supplementary file 1 — Supplementary Information 1. [file 41598_2025_96918_MOESM1_ESM.zip › ALL_IDB Dataset/L1/Im083_0.jpg]

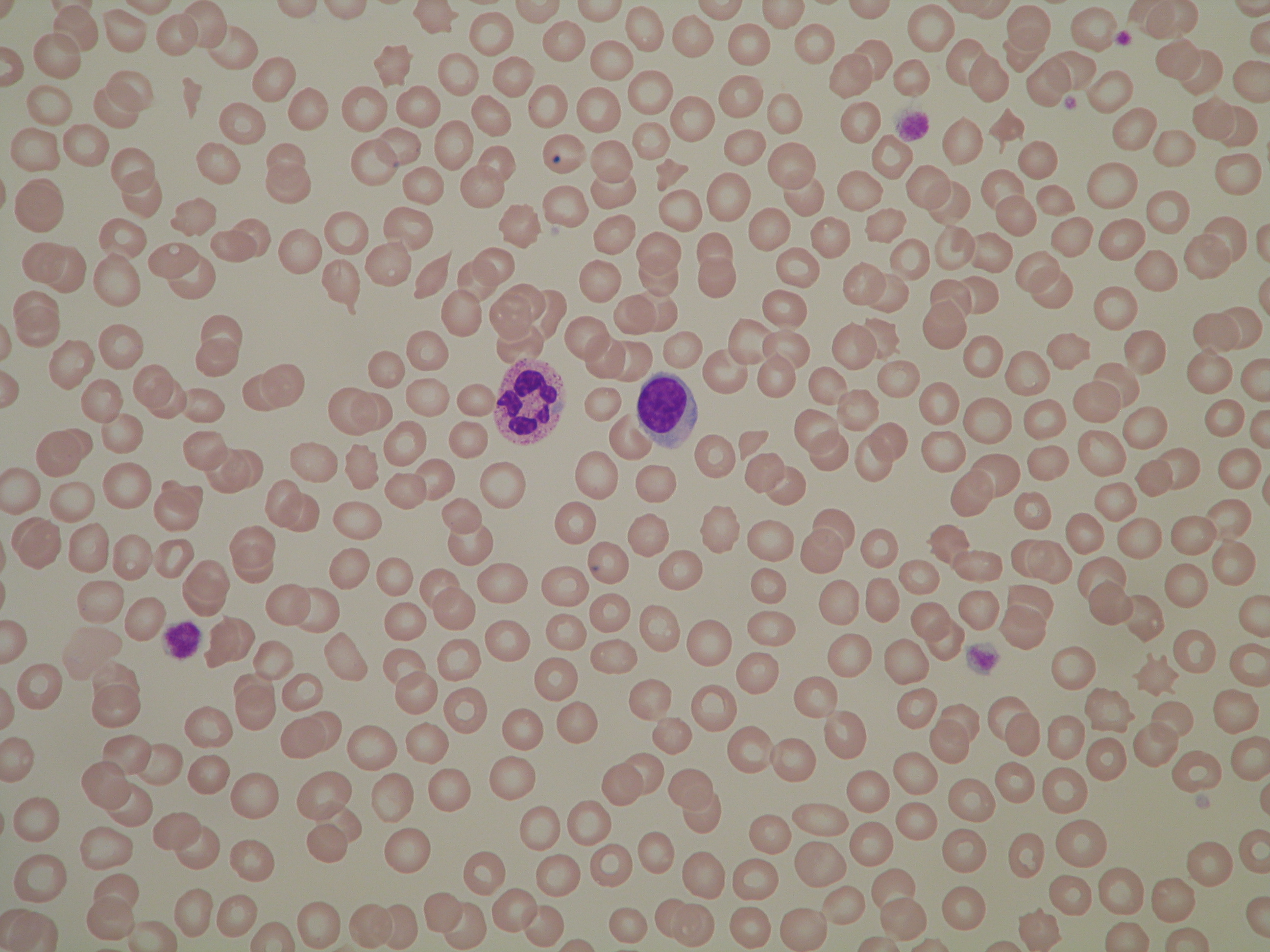

Supplement: Supplementary file 1 — Supplementary Information 1. [file 41598_2025_96918_MOESM1_ESM.zip › ALL_IDB Dataset/L1/Im084_0.jpg]

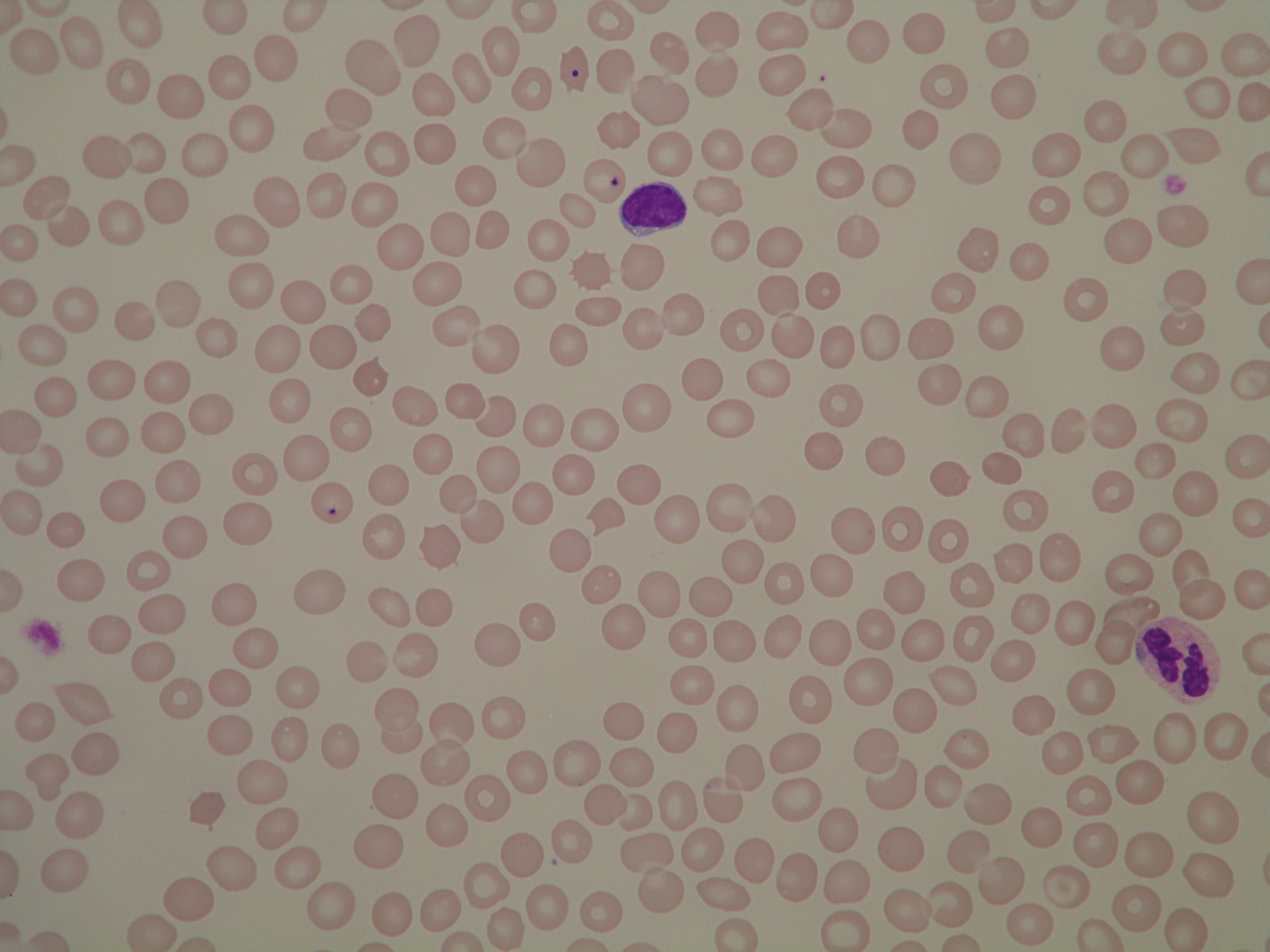

Supplement: Supplementary file 1 — Supplementary Information 1. [file 41598_2025_96918_MOESM1_ESM.zip › ALL_IDB Dataset/L1/Im085_0.jpg]

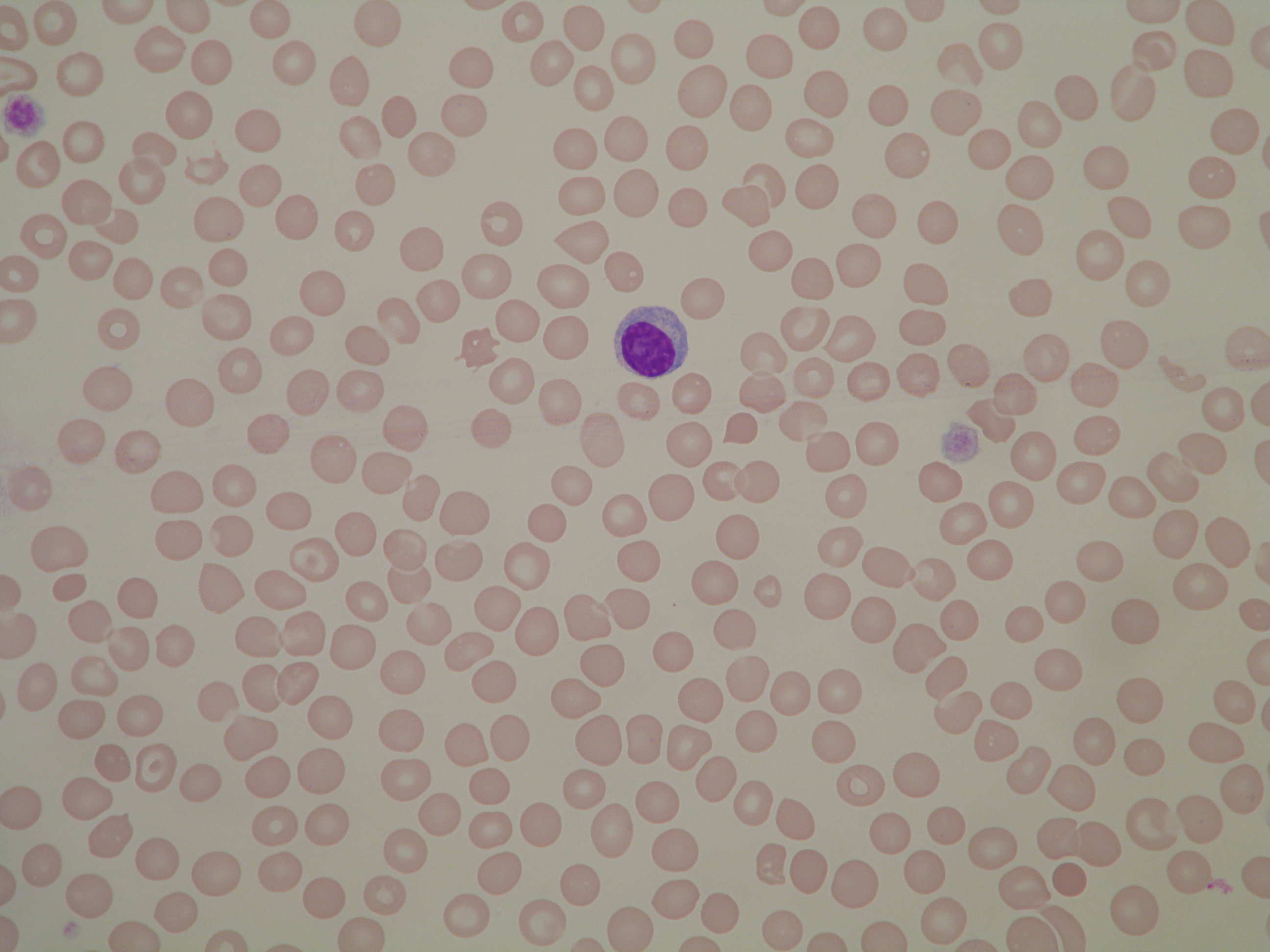

Supplement: Supplementary file 1 — Supplementary Information 1. [file 41598_2025_96918_MOESM1_ESM.zip › ALL_IDB Dataset/L1/Im086_0.jpg]

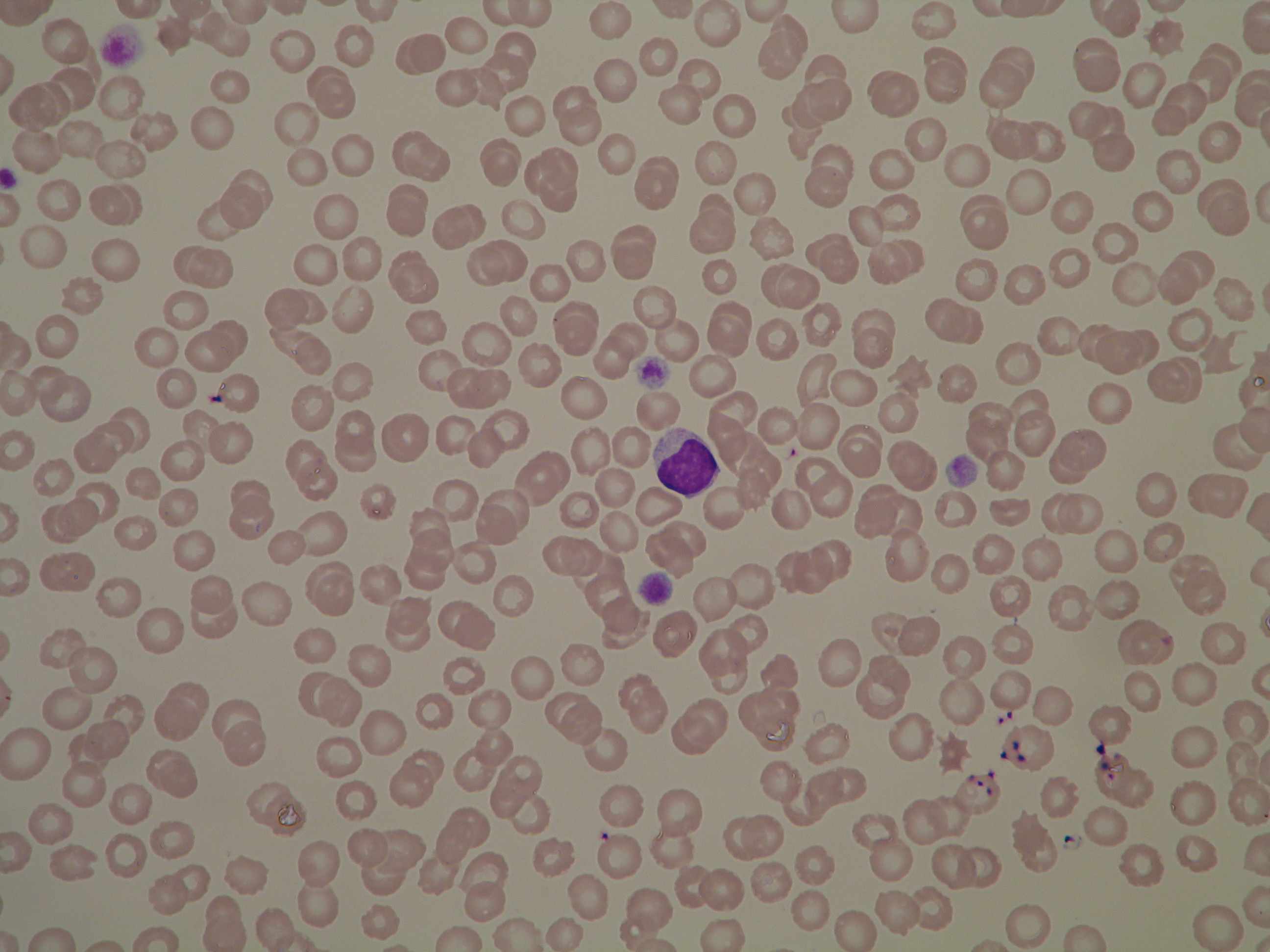

Supplement: Supplementary file 1 — Supplementary Information 1. [file 41598_2025_96918_MOESM1_ESM.zip › ALL_IDB Dataset/L1/Im087_0.jpg]

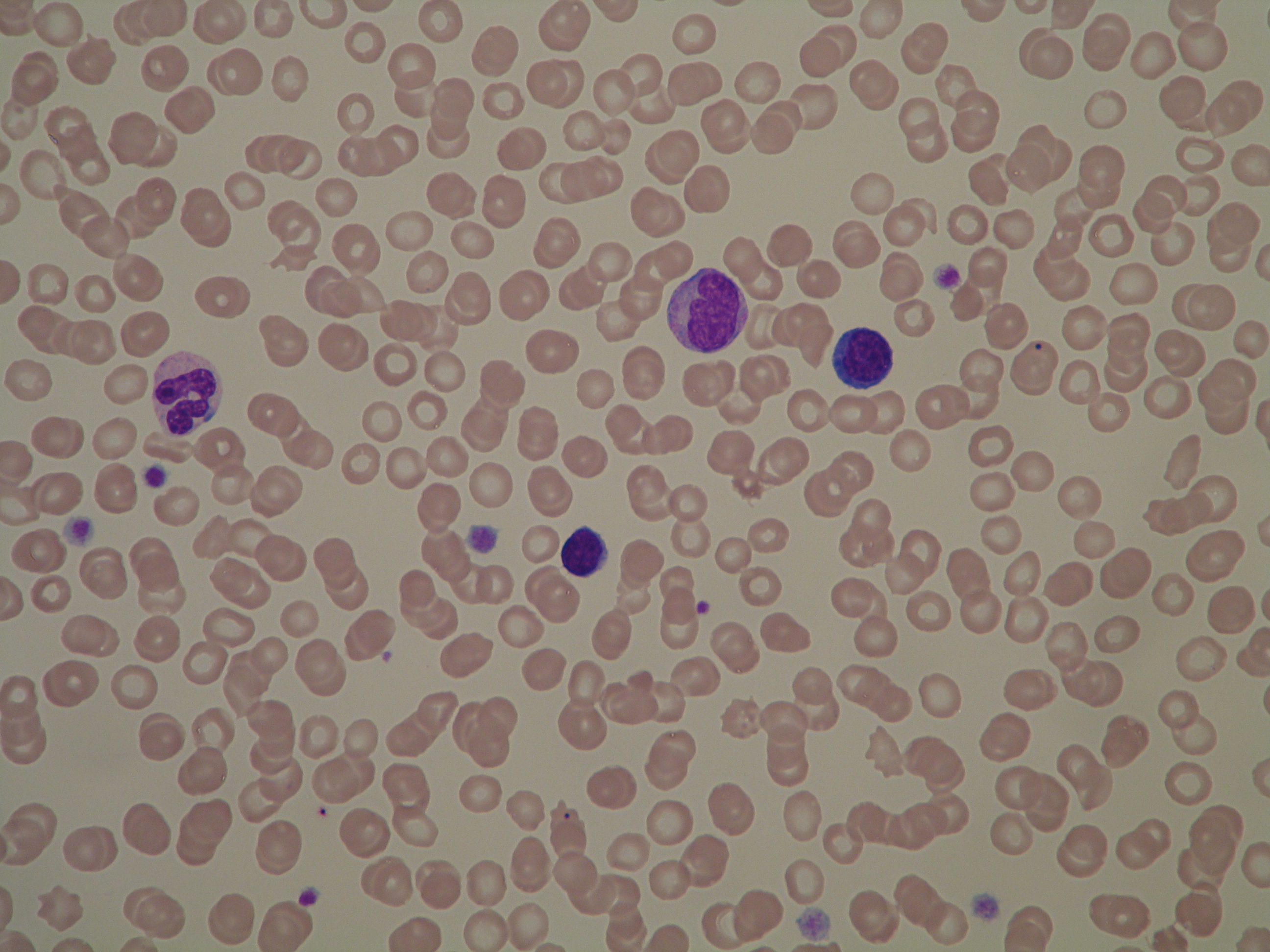

Supplement: Supplementary file 1 — Supplementary Information 1. [file 41598_2025_96918_MOESM1_ESM.zip › ALL_IDB Dataset/L1/Im088_0.jpg]

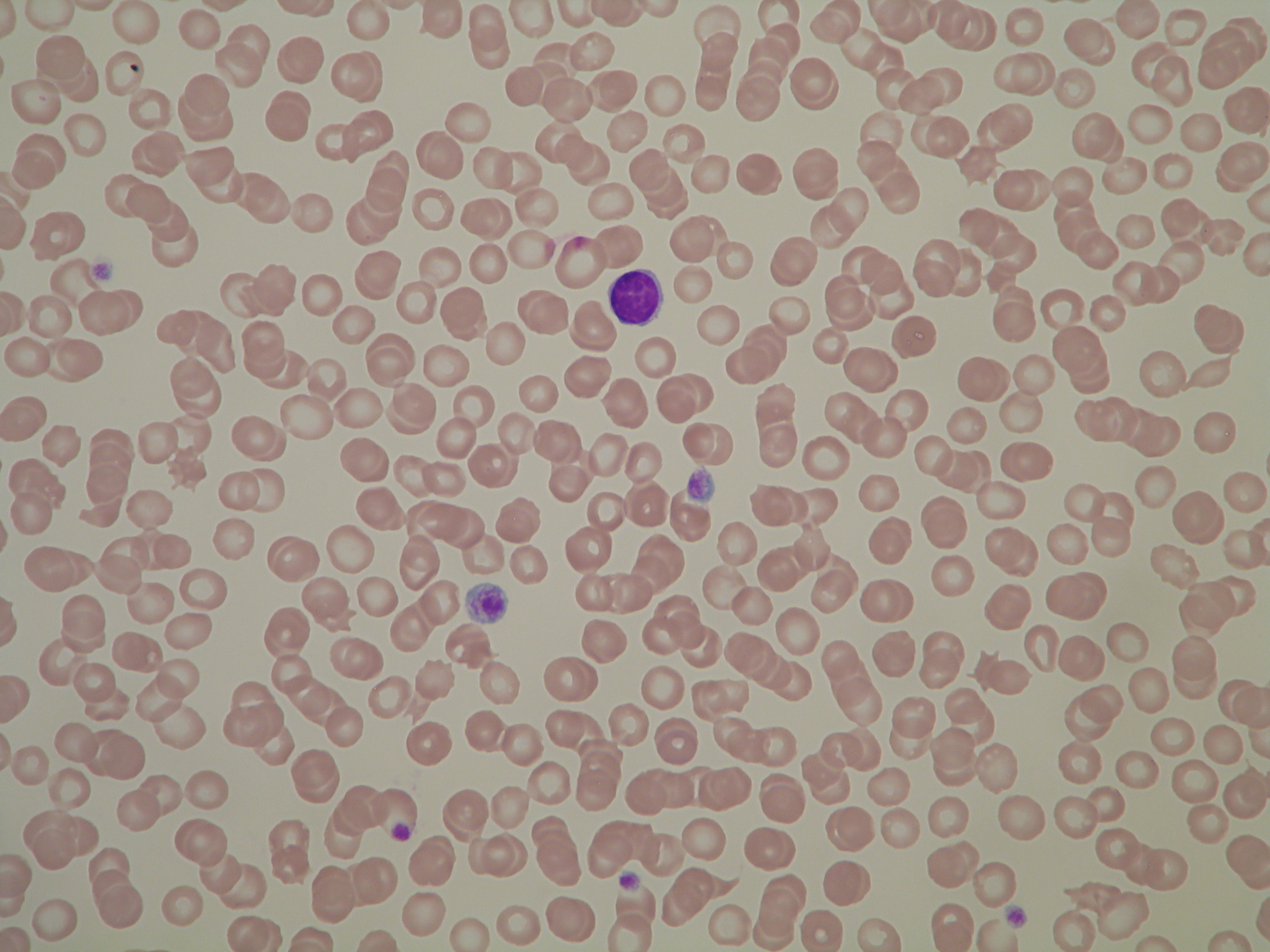

Supplement: Supplementary file 1 — Supplementary Information 1. [file 41598_2025_96918_MOESM1_ESM.zip › ALL_IDB Dataset/L1/Im089_0.jpg]

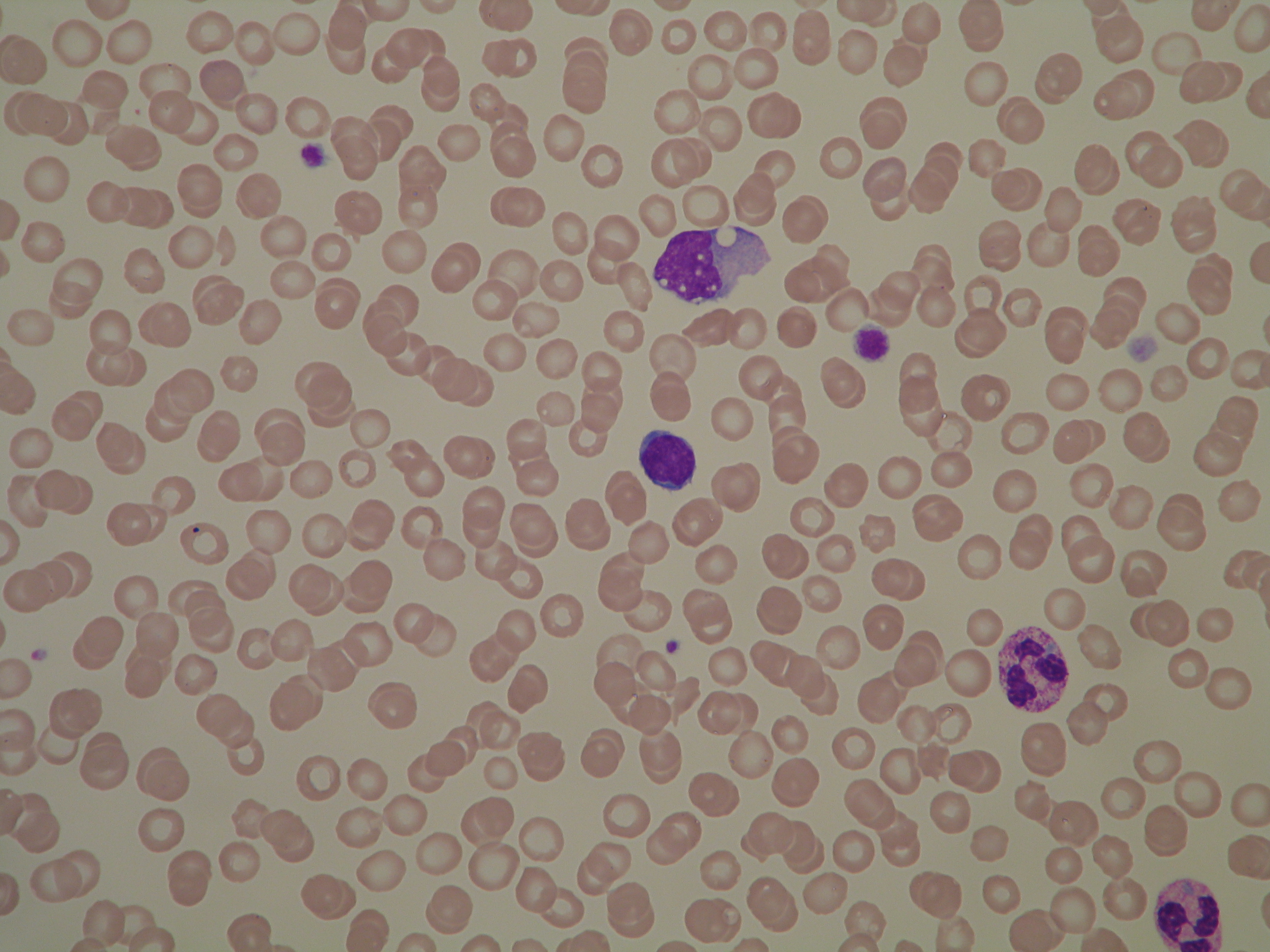

Supplement: Supplementary file 1 — Supplementary Information 1. [file 41598_2025_96918_MOESM1_ESM.zip › ALL_IDB Dataset/L1/Im090_0.jpg]

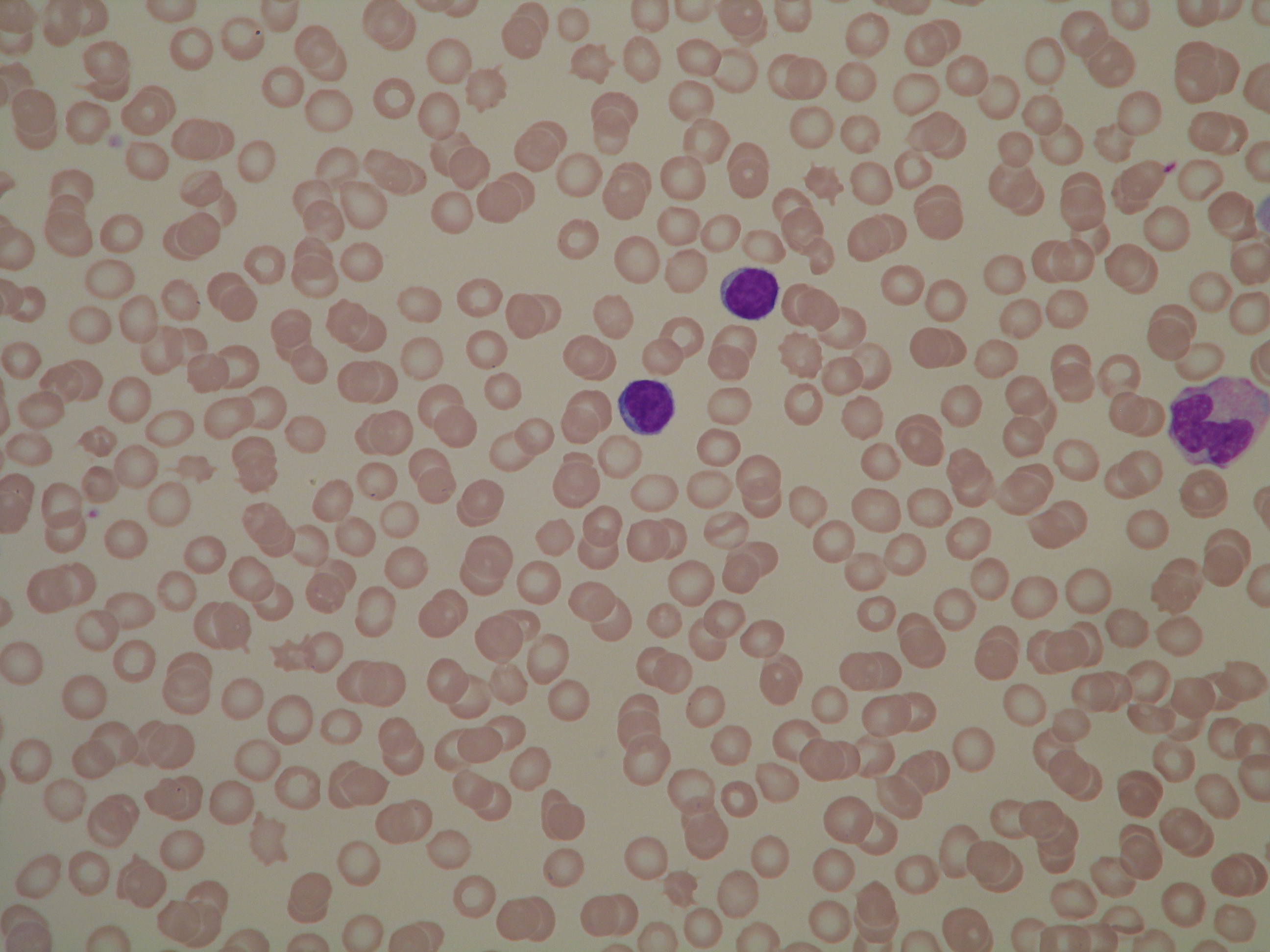

Supplement: Supplementary file 1 — Supplementary Information 1. [file 41598_2025_96918_MOESM1_ESM.zip › ALL_IDB Dataset/L1/Im091_0.jpg]

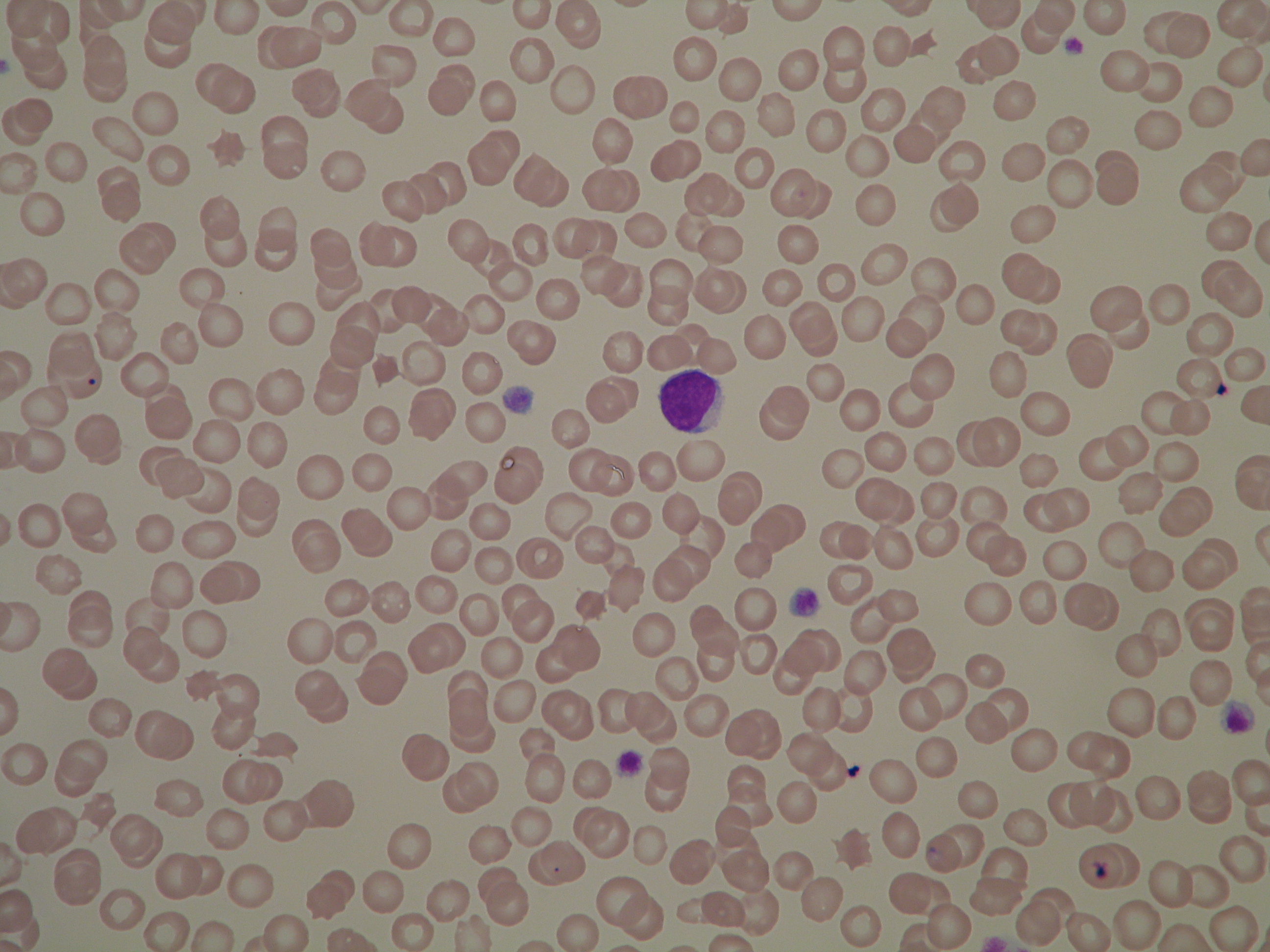

Supplement: Supplementary file 1 — Supplementary Information 1. [file 41598_2025_96918_MOESM1_ESM.zip › ALL_IDB Dataset/L1/Im0921_0.jpg]

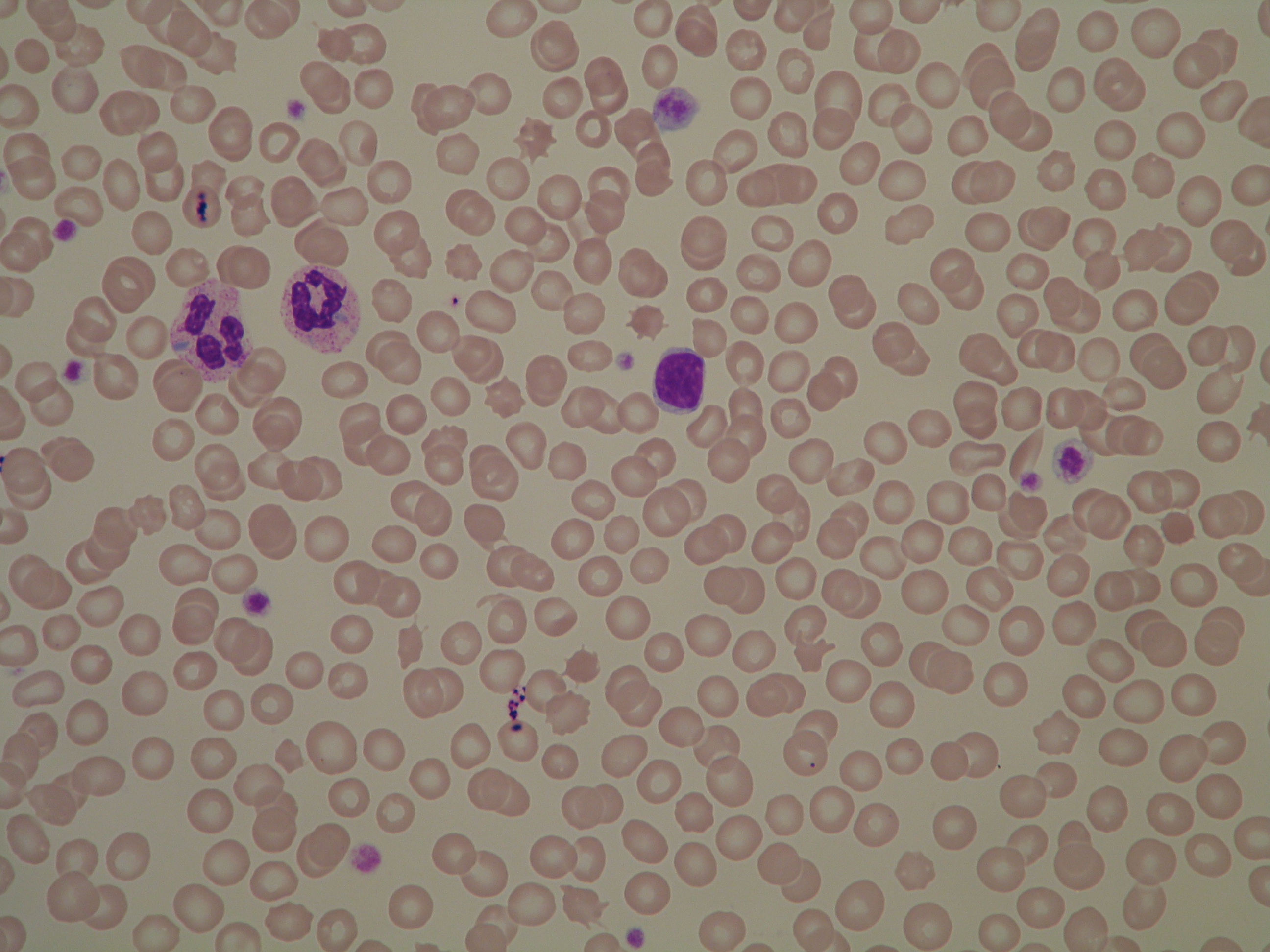

Supplement: Supplementary file 1 — Supplementary Information 1. [file 41598_2025_96918_MOESM1_ESM.zip › ALL_IDB Dataset/L1/Im093_0.jpg]

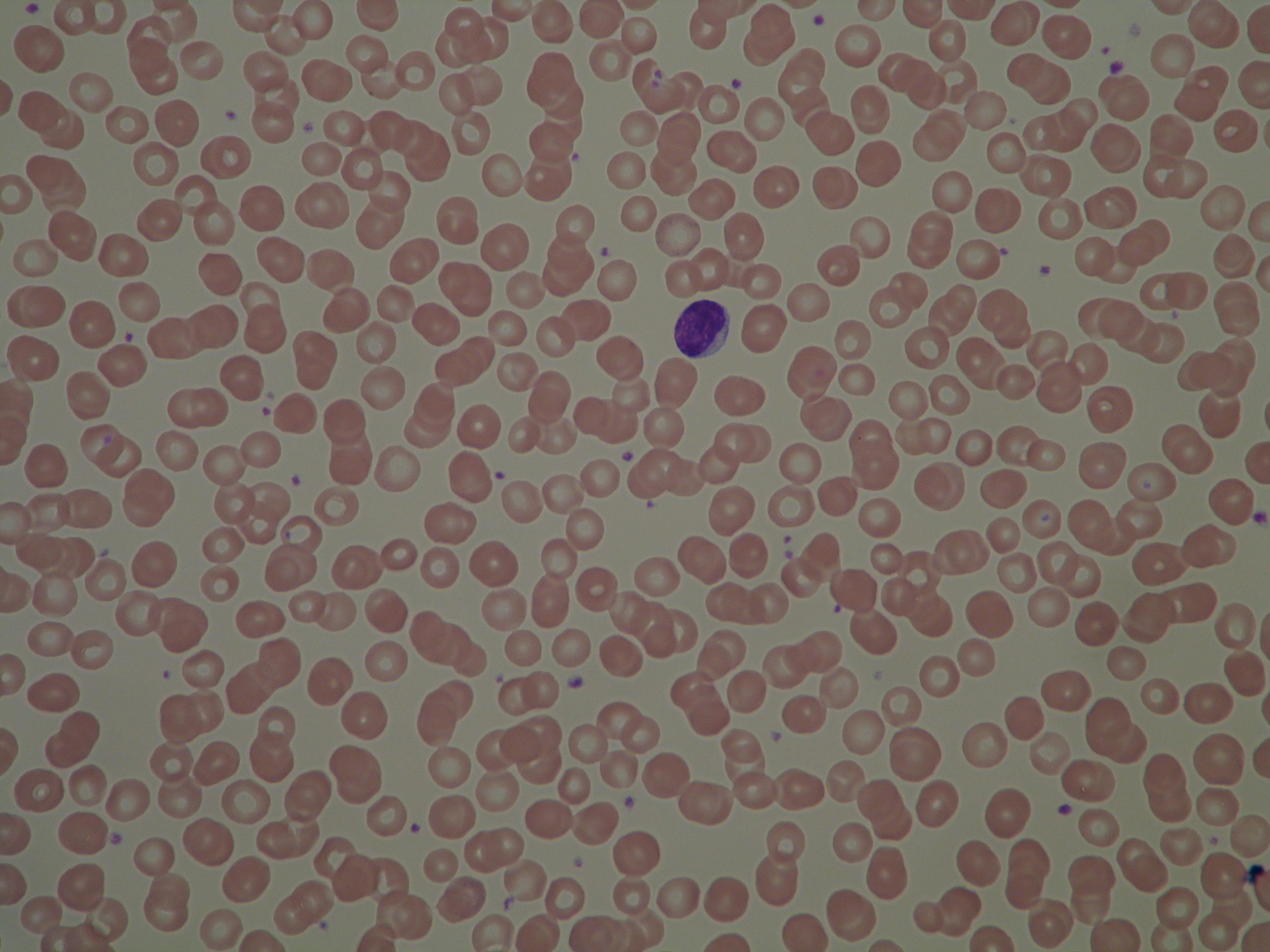

Supplement: Supplementary file 1 — Supplementary Information 1. [file 41598_2025_96918_MOESM1_ESM.zip › ALL_IDB Dataset/L1/Im098_0.jpg]

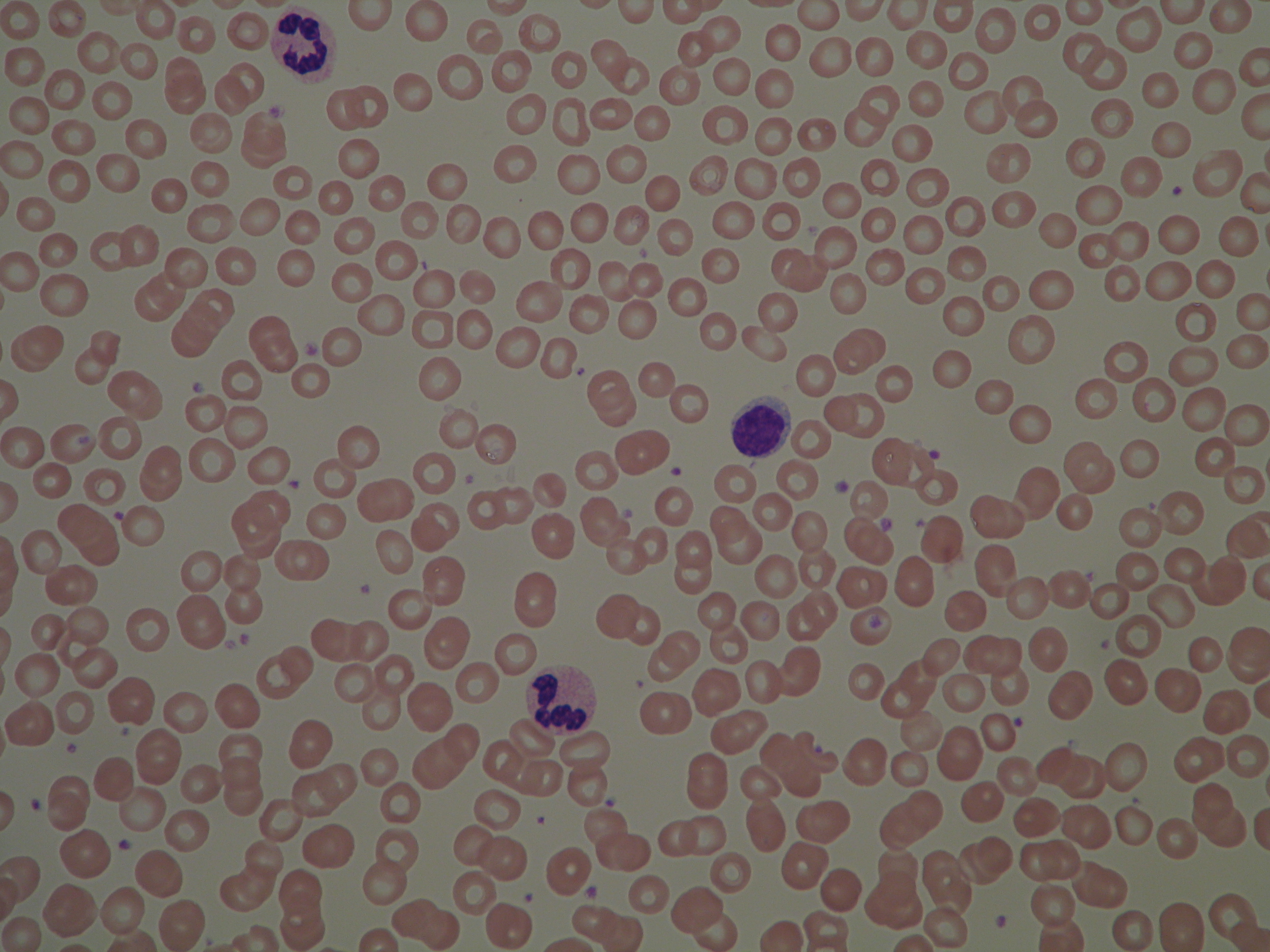

Supplement: Supplementary file 1 — Supplementary Information 1. [file 41598_2025_96918_MOESM1_ESM.zip › ALL_IDB Dataset/L1/Im099_0.jpg]

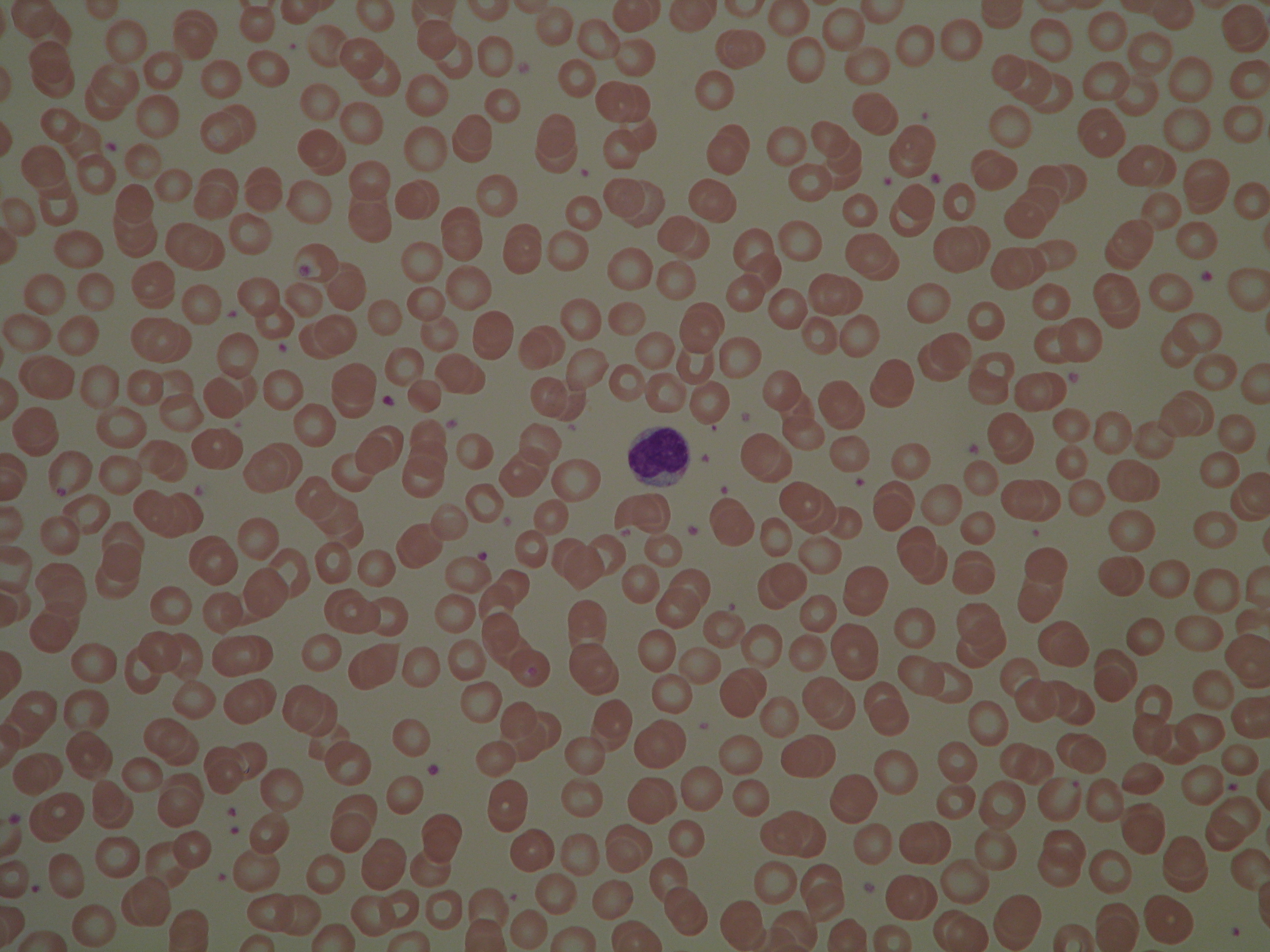

Supplement: Supplementary file 1 — Supplementary Information 1. [file 41598_2025_96918_MOESM1_ESM.zip › ALL_IDB Dataset/L1/Im100_0.jpg]

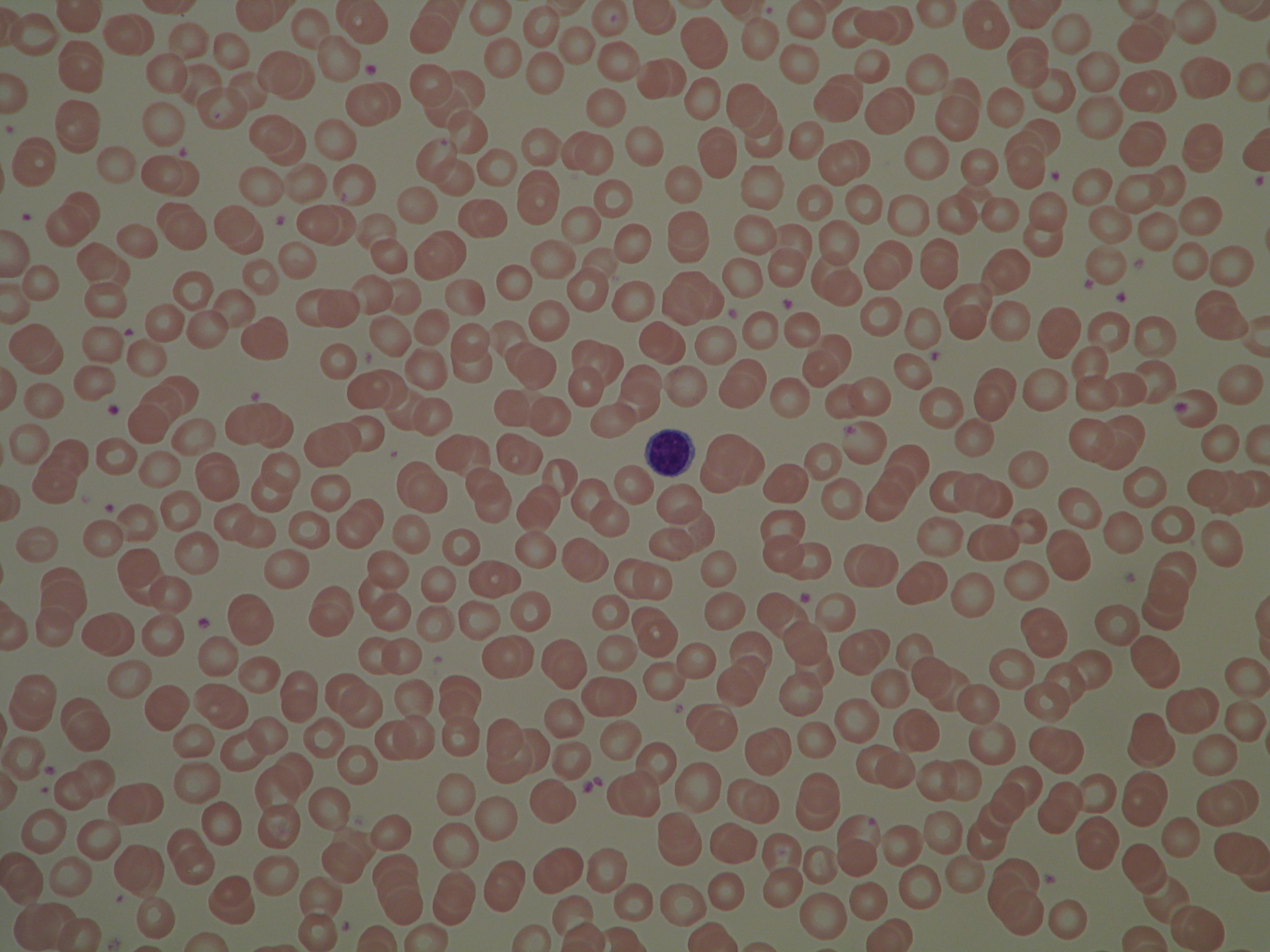

Supplement: Supplementary file 1 — Supplementary Information 1. [file 41598_2025_96918_MOESM1_ESM.zip › ALL_IDB Dataset/L1/Im101_0.jpg]

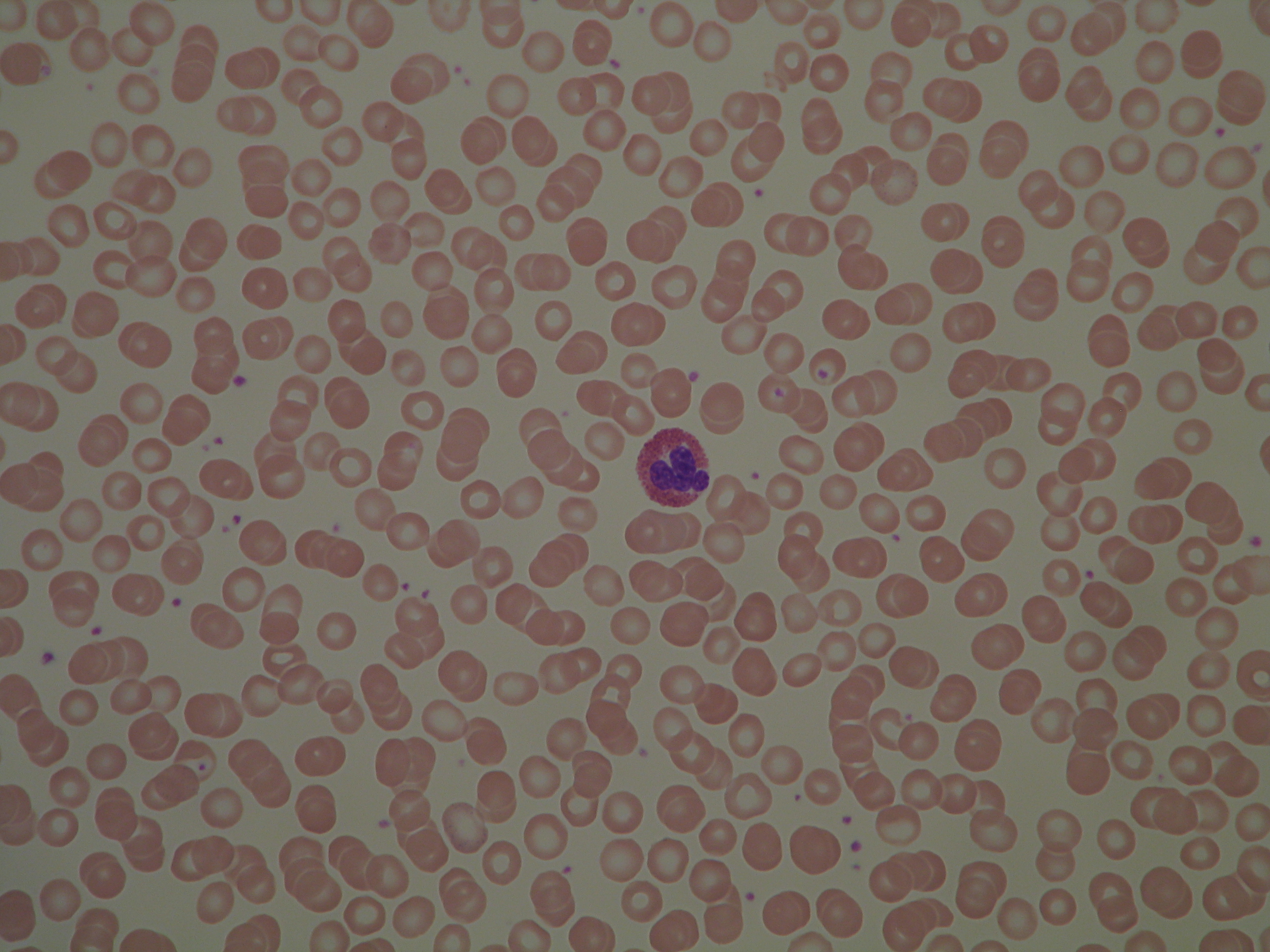

Supplement: Supplementary file 1 — Supplementary Information 1. [file 41598_2025_96918_MOESM1_ESM.zip › ALL_IDB Dataset/L1/Im1021_0.jpg]

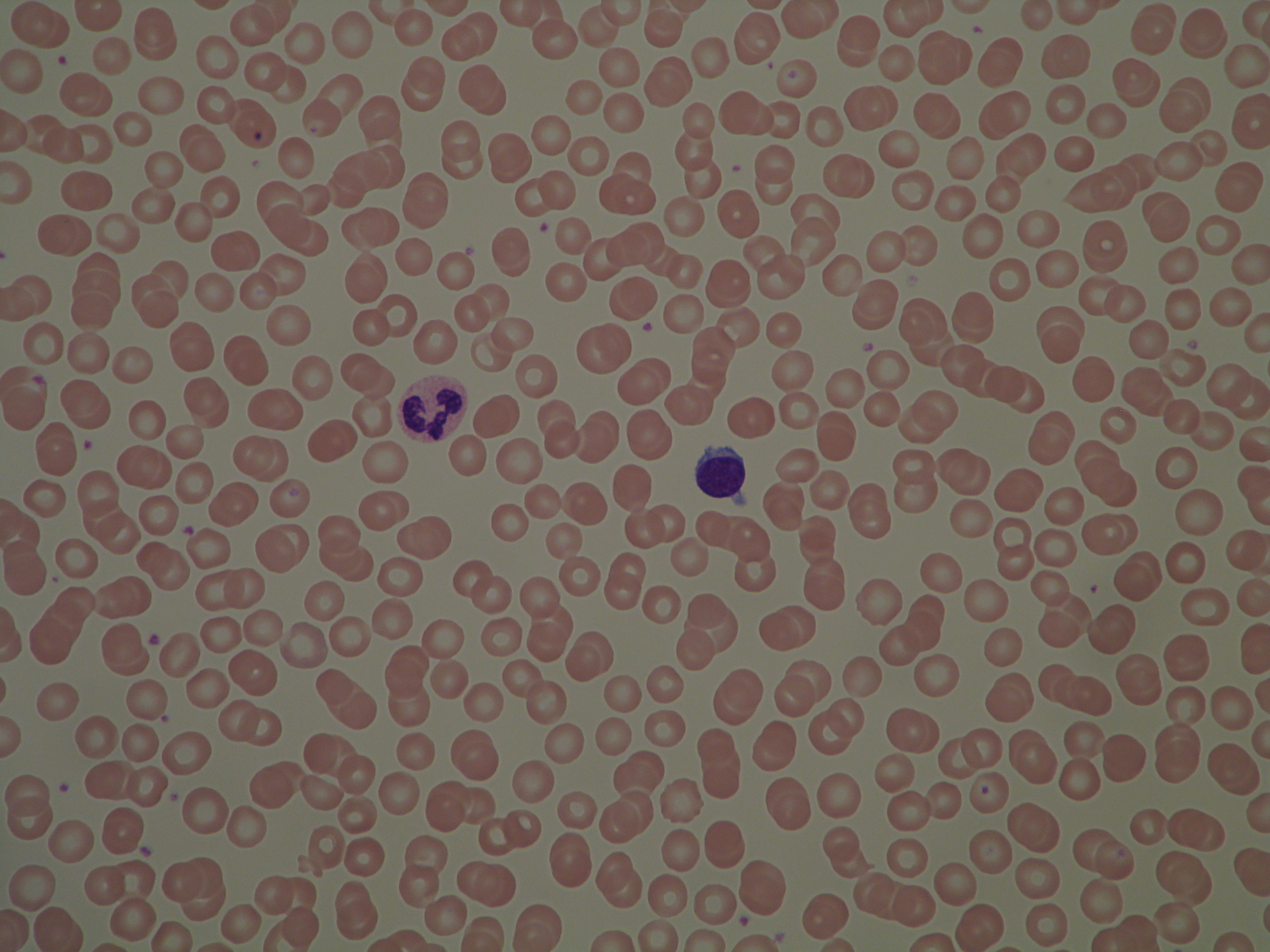

Supplement: Supplementary file 1 — Supplementary Information 1. [file 41598_2025_96918_MOESM1_ESM.zip › ALL_IDB Dataset/L1/Im103_0.jpg]

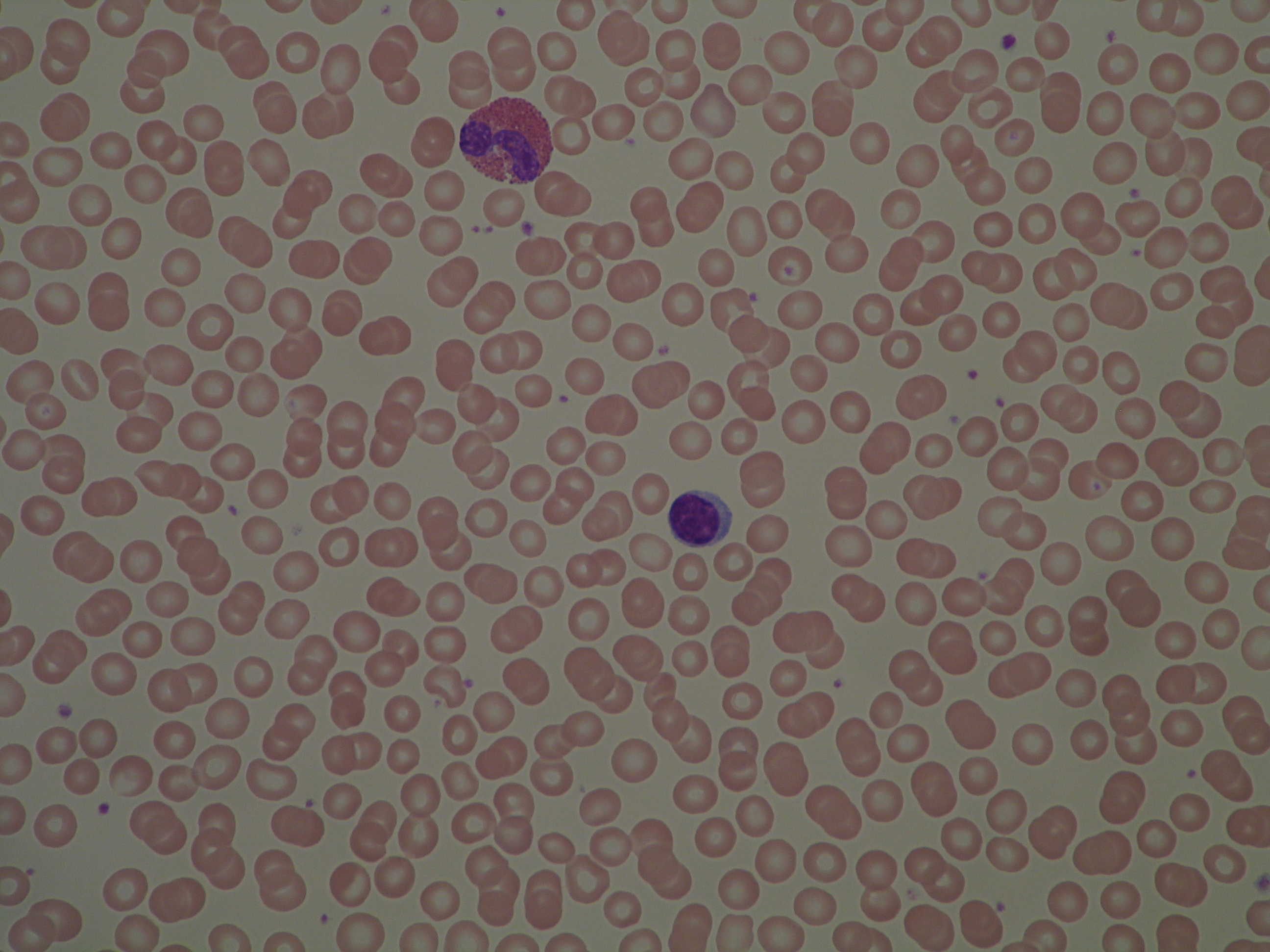

Supplement: Supplementary file 1 — Supplementary Information 1. [file 41598_2025_96918_MOESM1_ESM.zip › ALL_IDB Dataset/L1/Im104_0.jpg]

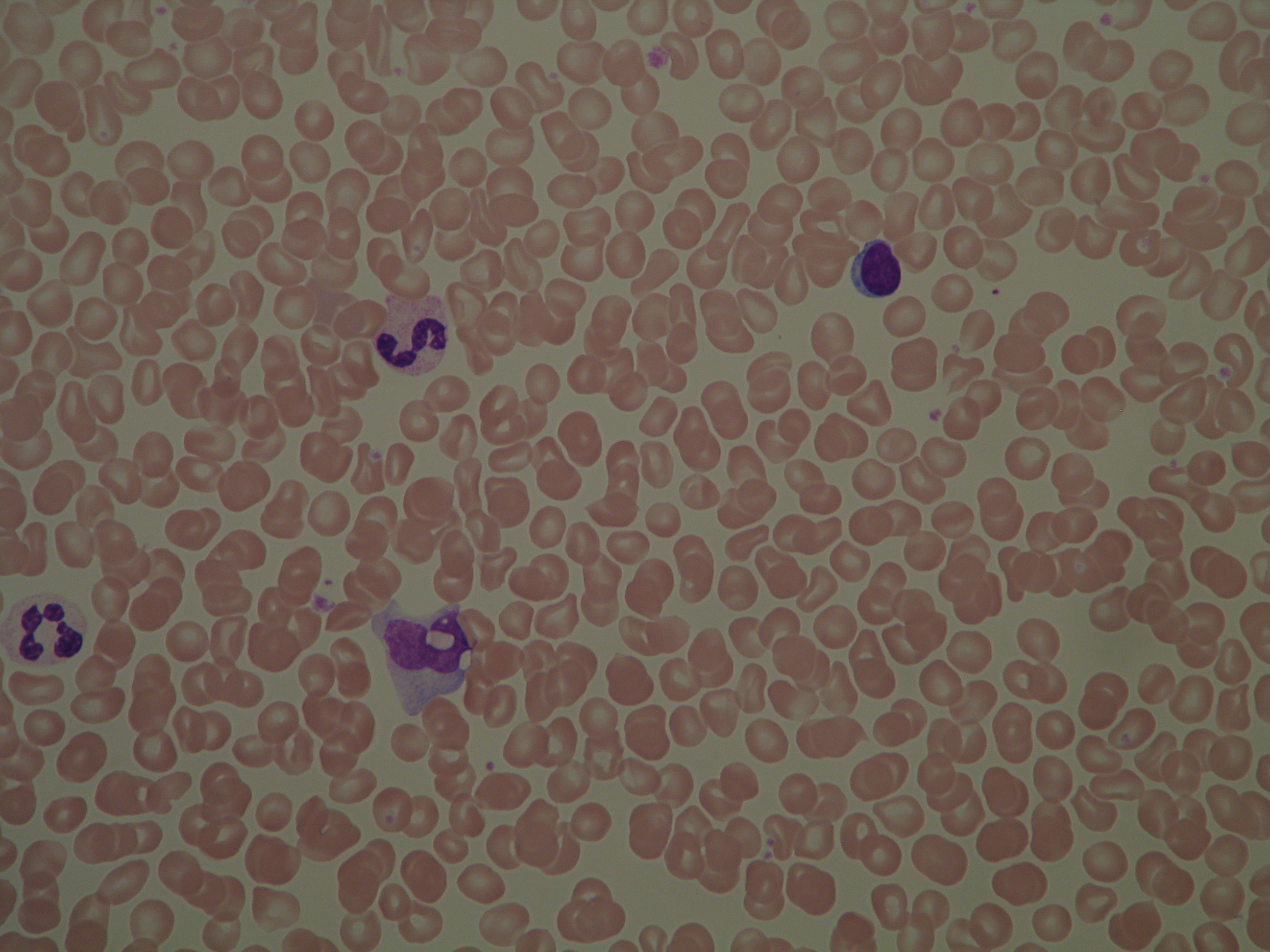

Supplement: Supplementary file 1 — Supplementary Information 1. [file 41598_2025_96918_MOESM1_ESM.zip › ALL_IDB Dataset/L1/Im105_0.jpg]

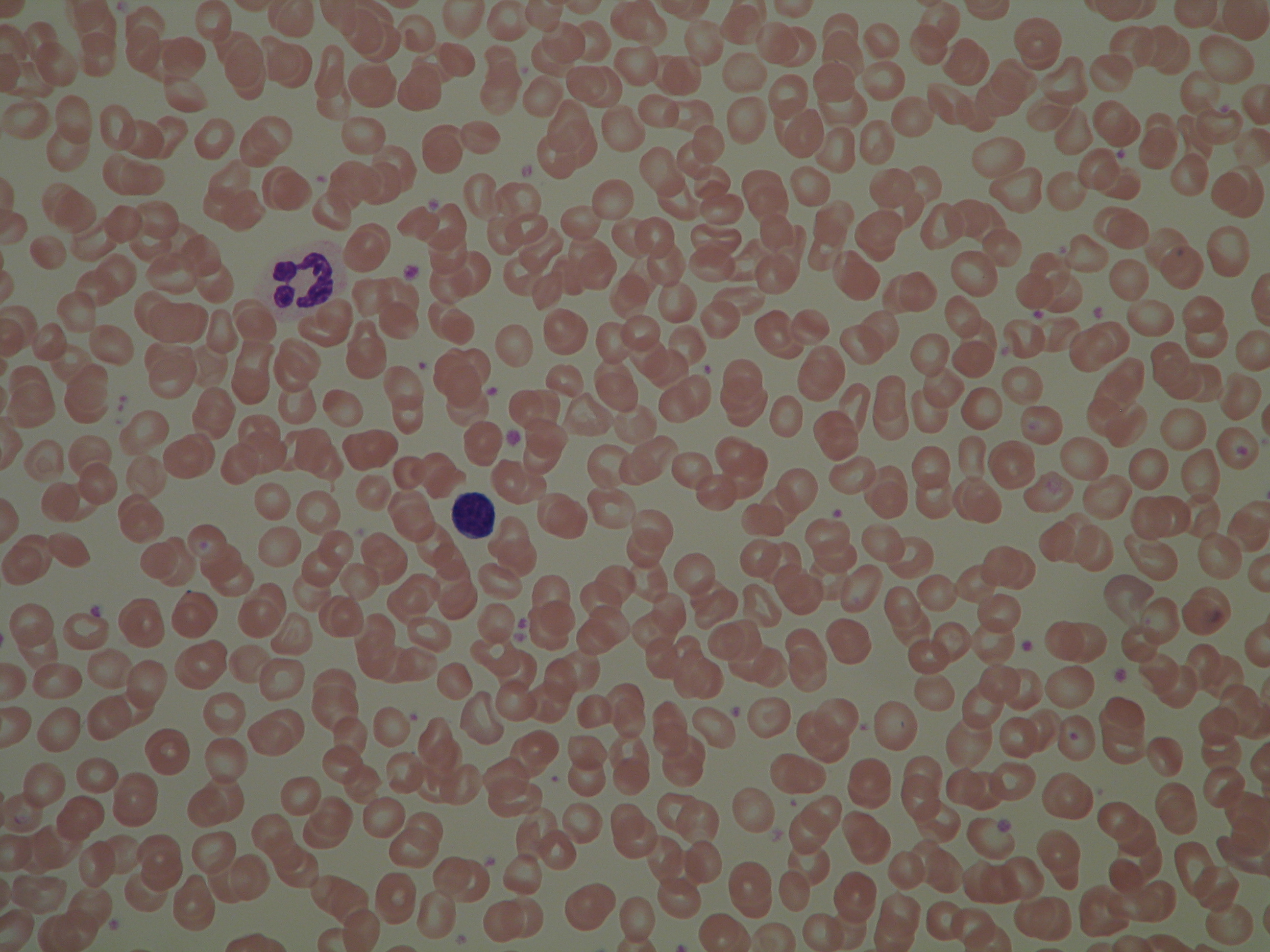

Supplement: Supplementary file 1 — Supplementary Information 1. [file 41598_2025_96918_MOESM1_ESM.zip › ALL_IDB Dataset/L1/Im106_0.jpg]

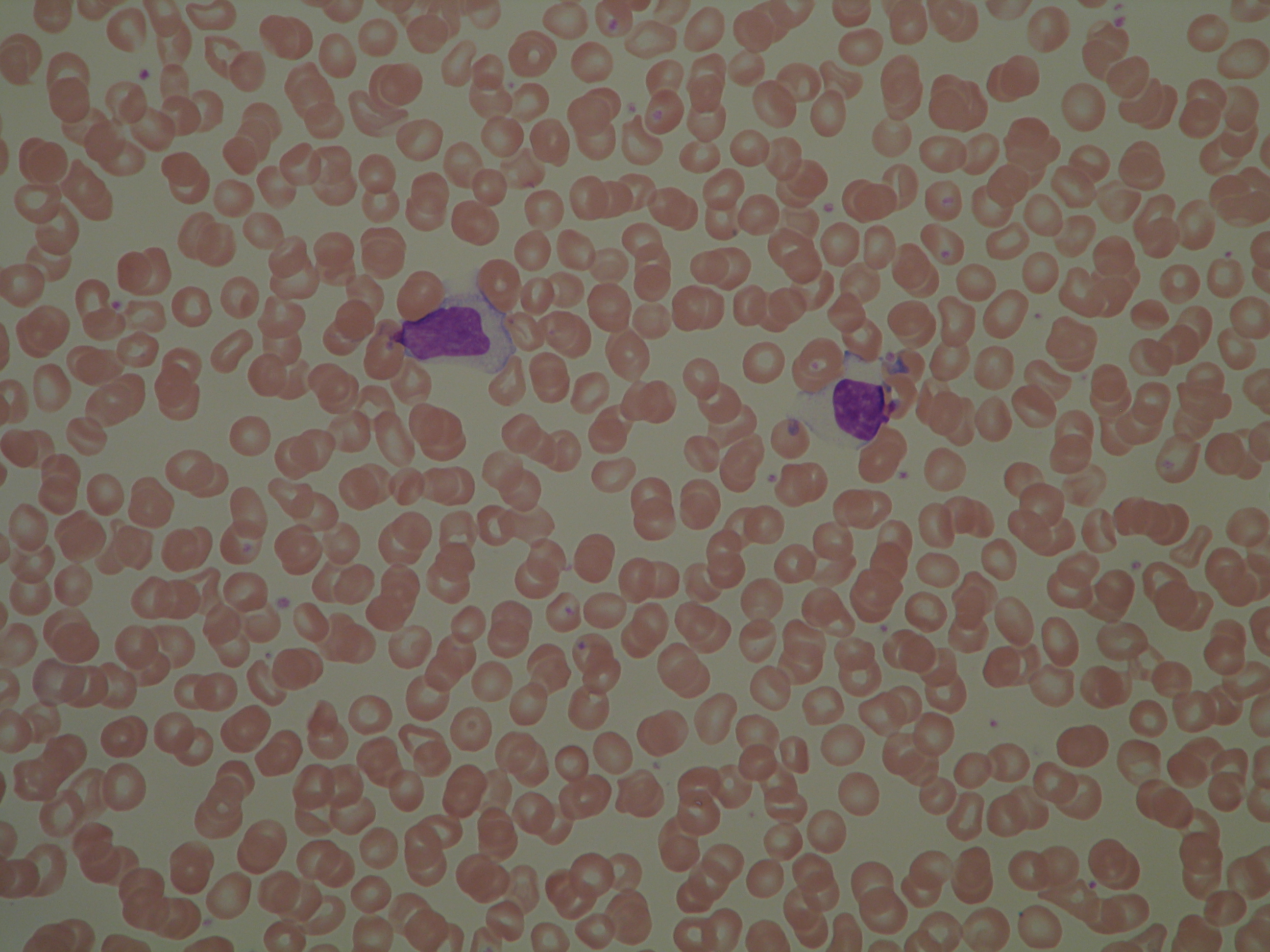

Supplement: Supplementary file 1 — Supplementary Information 1. [file 41598_2025_96918_MOESM1_ESM.zip › ALL_IDB Dataset/L1/Im107_0.jpg]

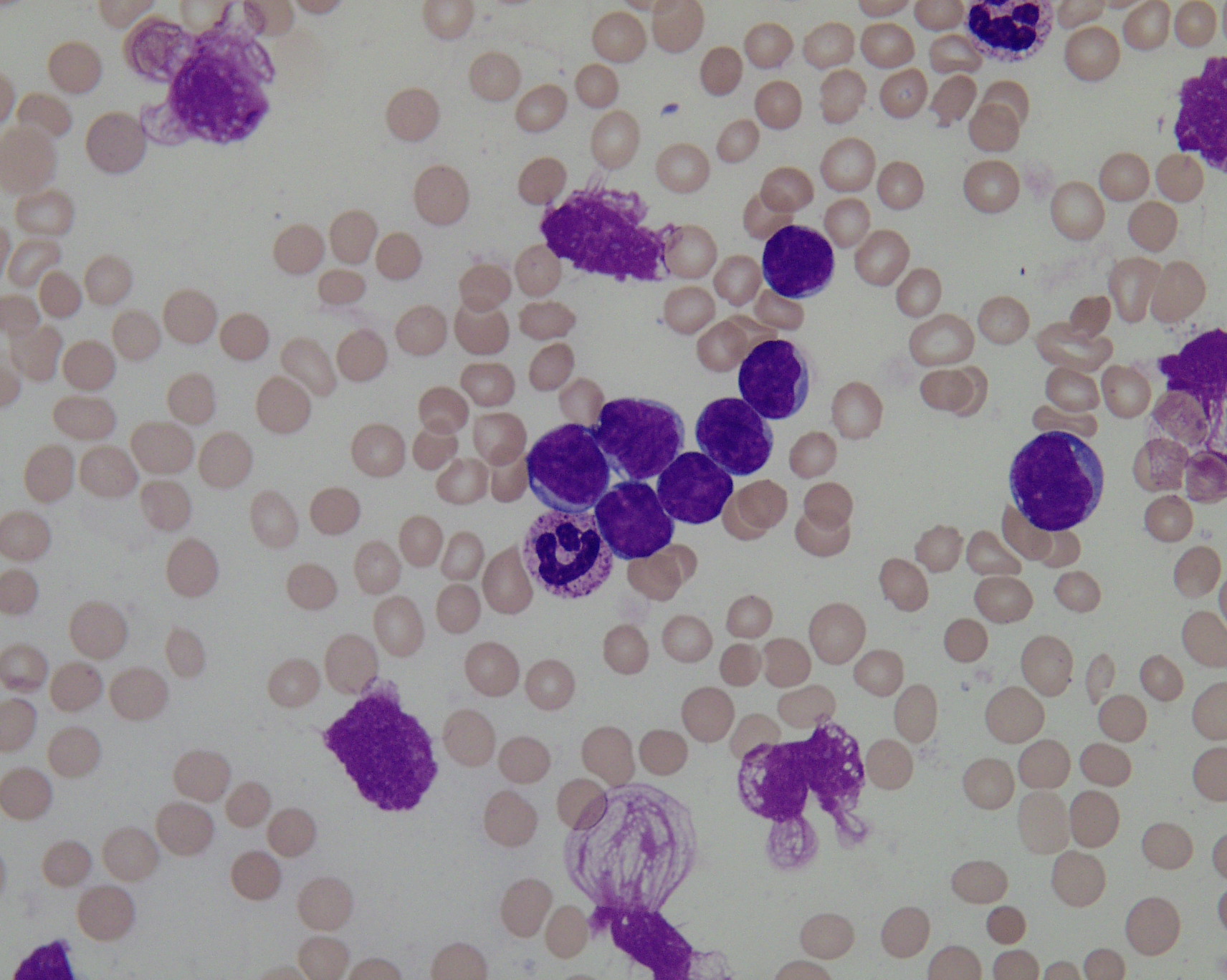

Supplement: Supplementary file 1 — Supplementary Information 1. [file 41598_2025_96918_MOESM1_ESM.zip › ALL_IDB Dataset/L2/Im0011_1.jpg]

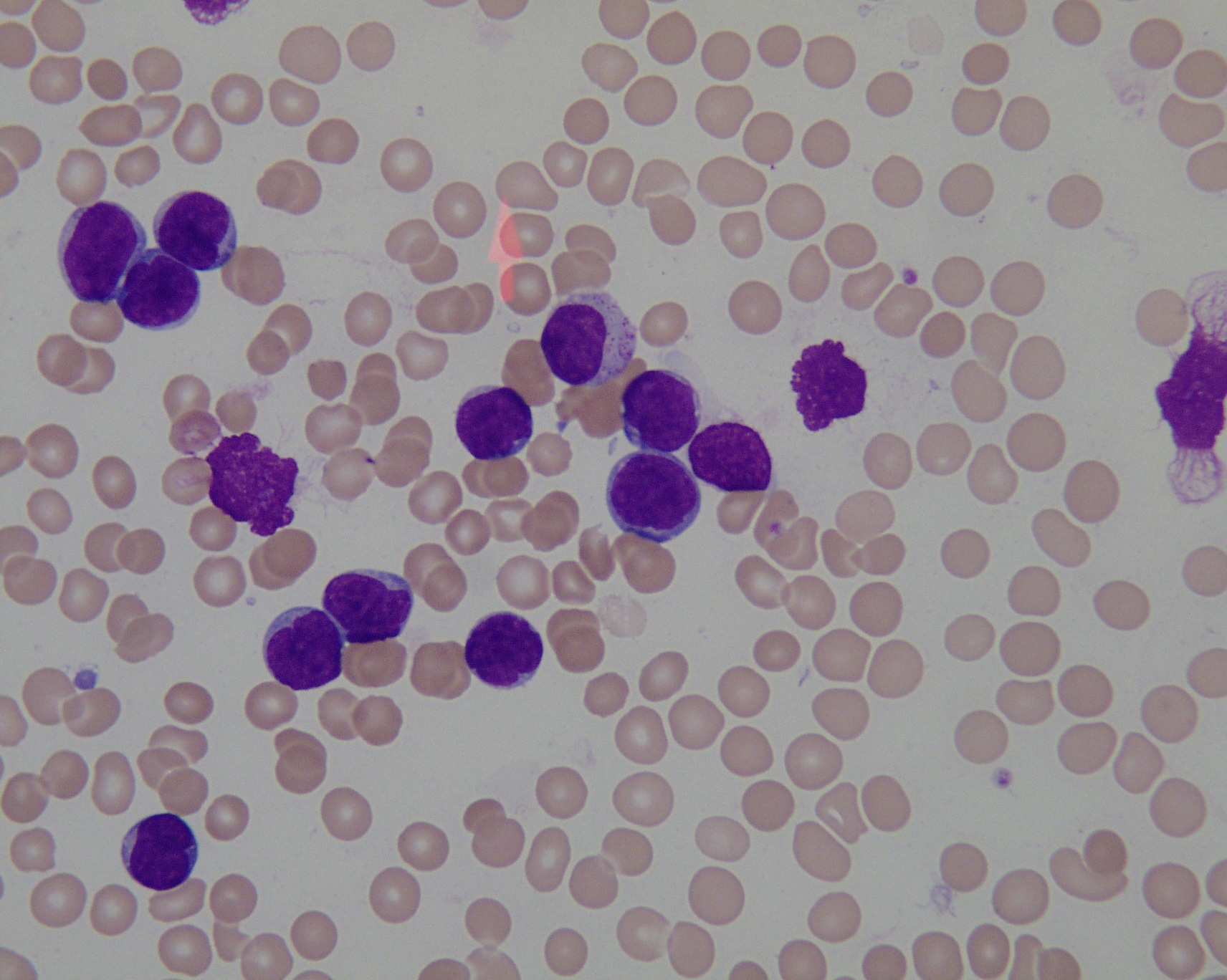

Supplement: Supplementary file 1 — Supplementary Information 1. [file 41598_2025_96918_MOESM1_ESM.zip › ALL_IDB Dataset/L2/Im0031_1.jpg]

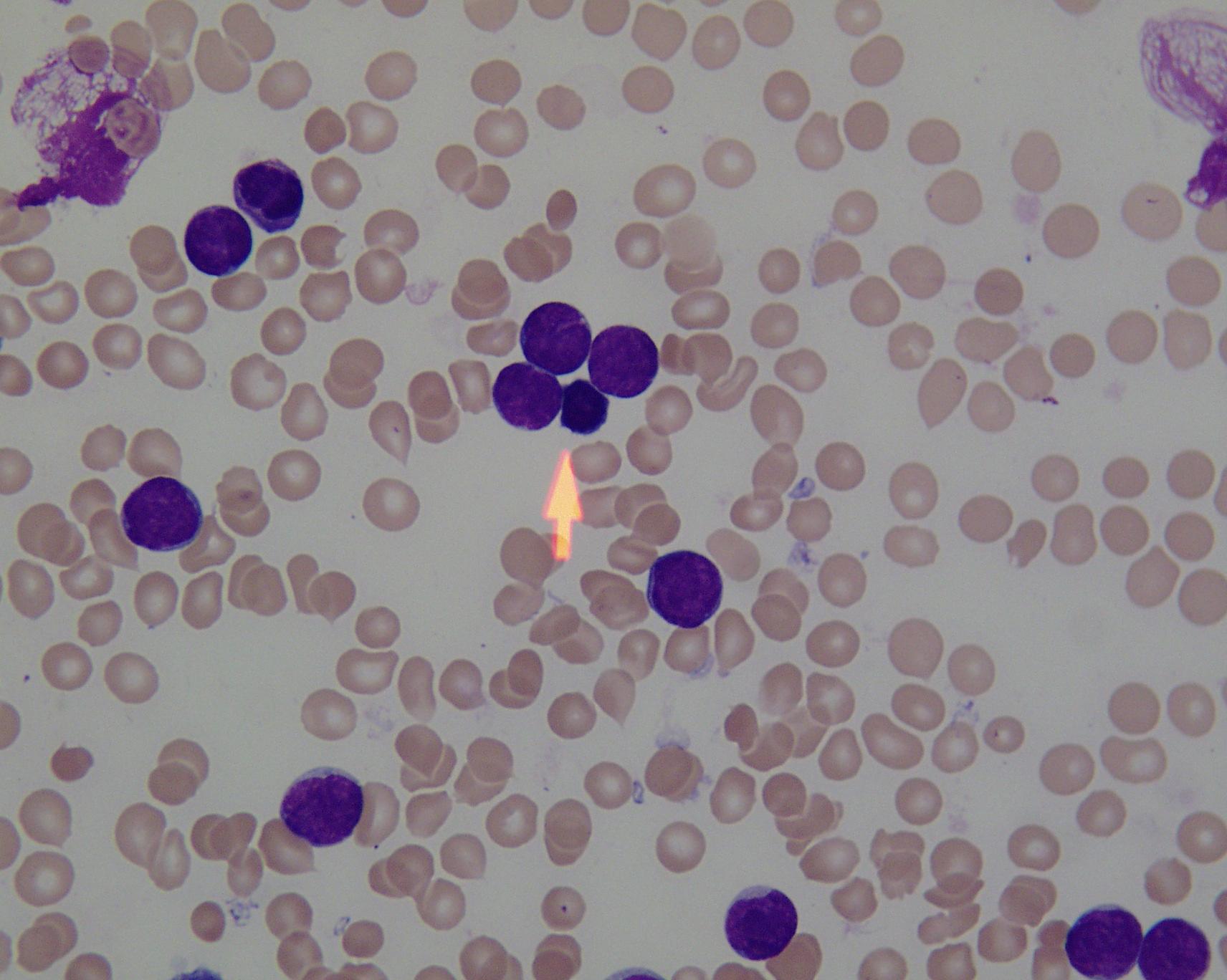

Supplement: Supplementary file 1 — Supplementary Information 1. [file 41598_2025_96918_MOESM1_ESM.zip › ALL_IDB Dataset/L2/Im003_10.jpg]

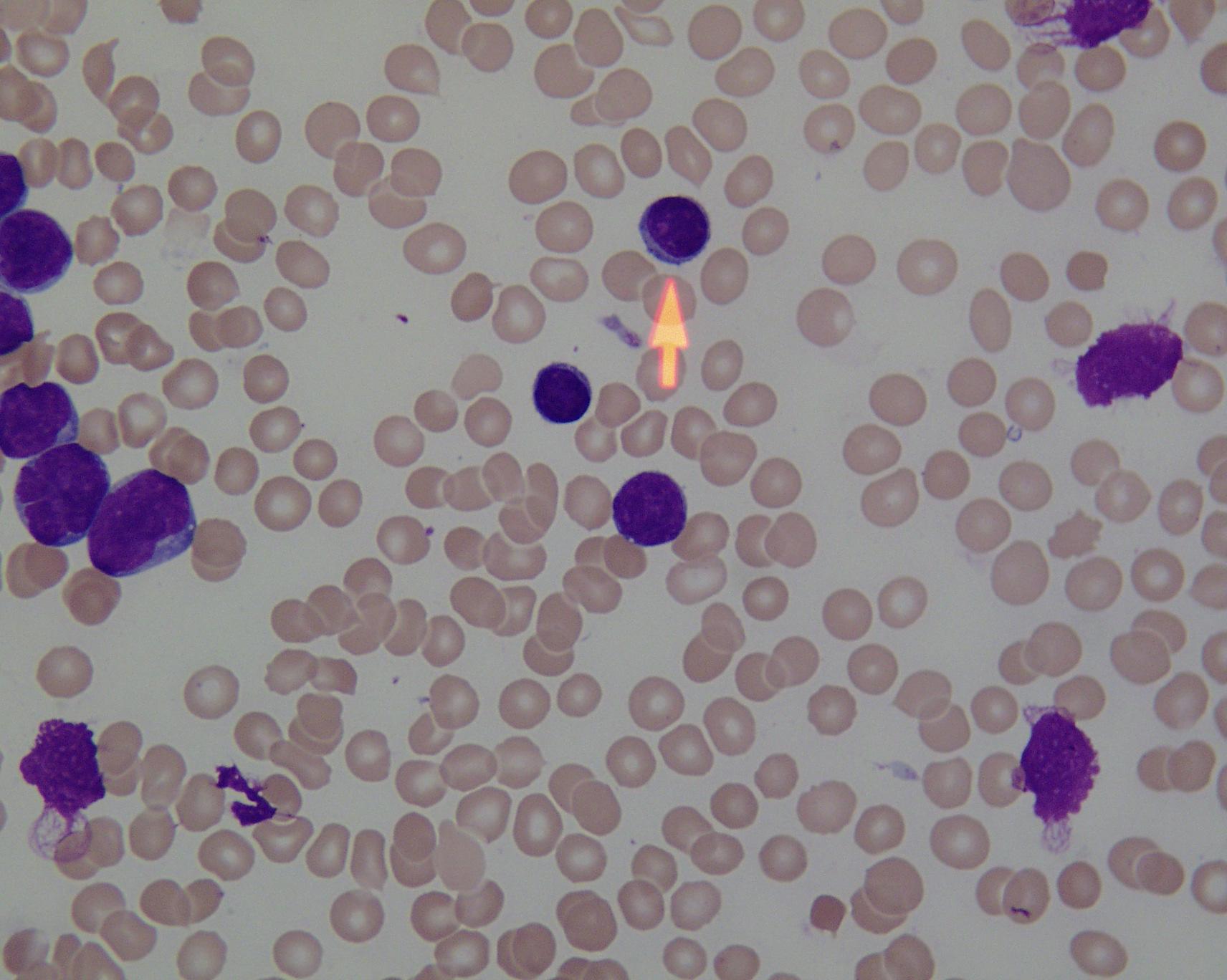

Supplement: Supplementary file 1 — Supplementary Information 1. [file 41598_2025_96918_MOESM1_ESM.zip › ALL_IDB Dataset/L2/Im003_11.jpg]

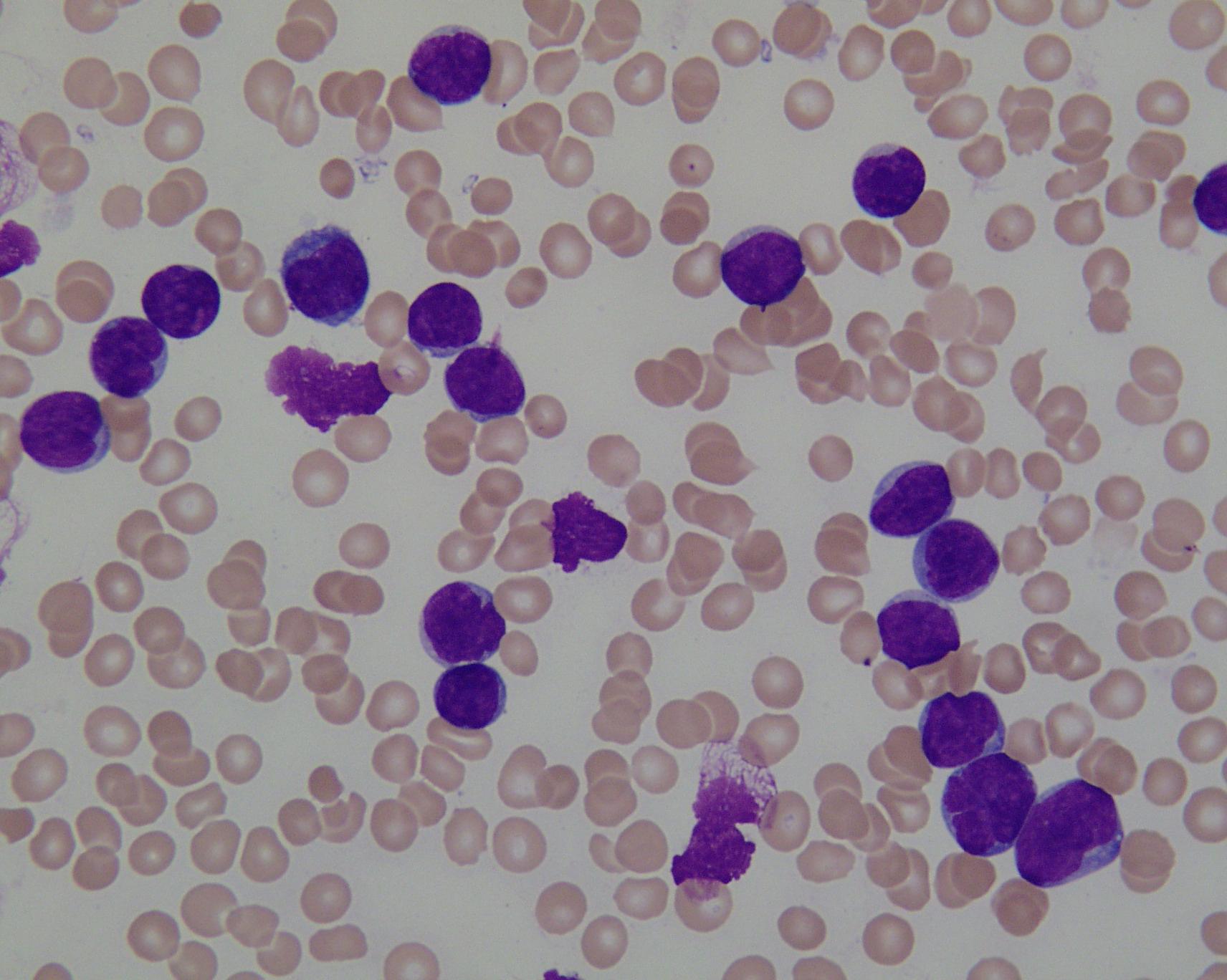

Supplement: Supplementary file 1 — Supplementary Information 1. [file 41598_2025_96918_MOESM1_ESM.zip › ALL_IDB Dataset/L2/Im003_12.jpg]

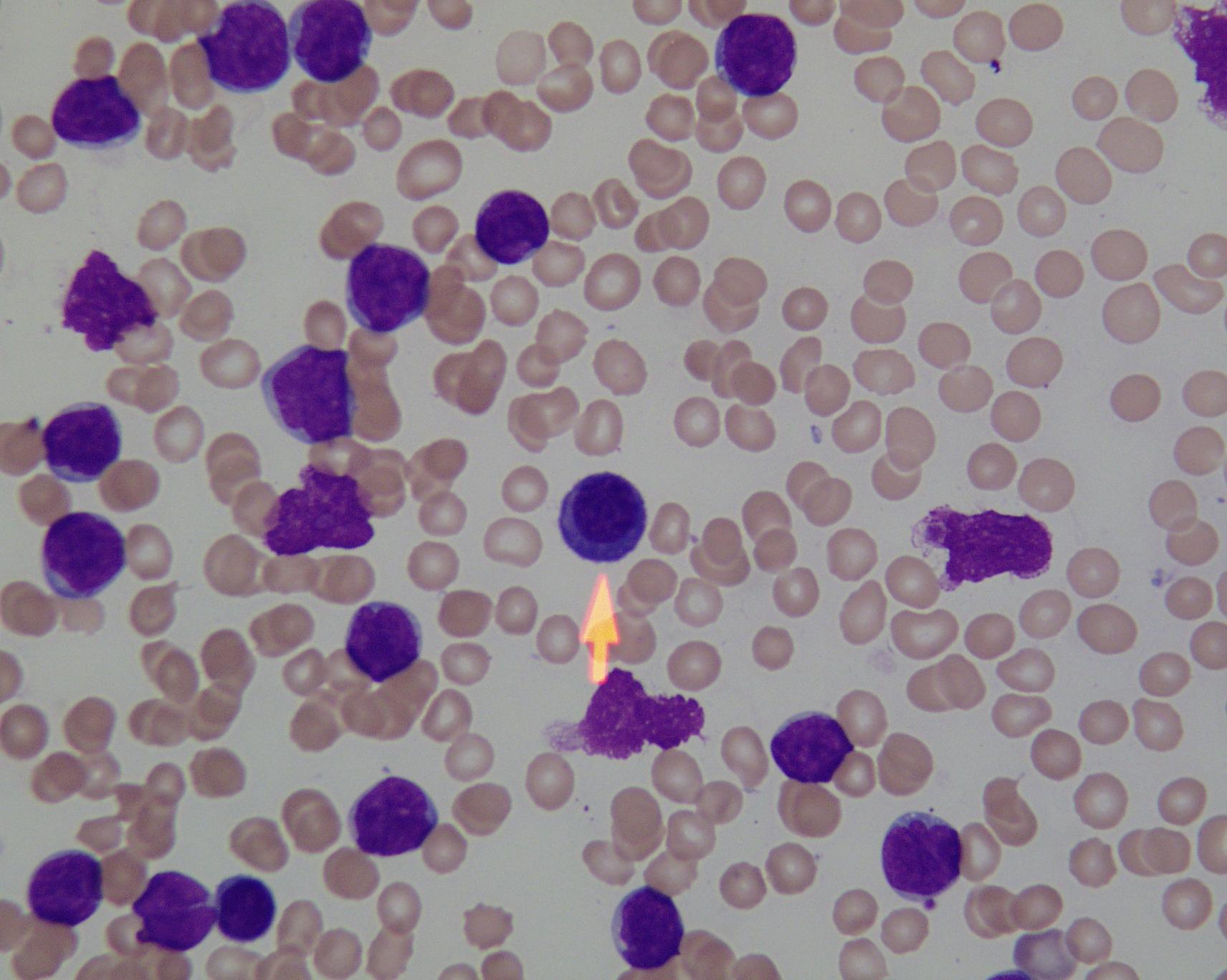

Supplement: Supplementary file 1 — Supplementary Information 1. [file 41598_2025_96918_MOESM1_ESM.zip › ALL_IDB Dataset/L2/Im003_13.jpg]

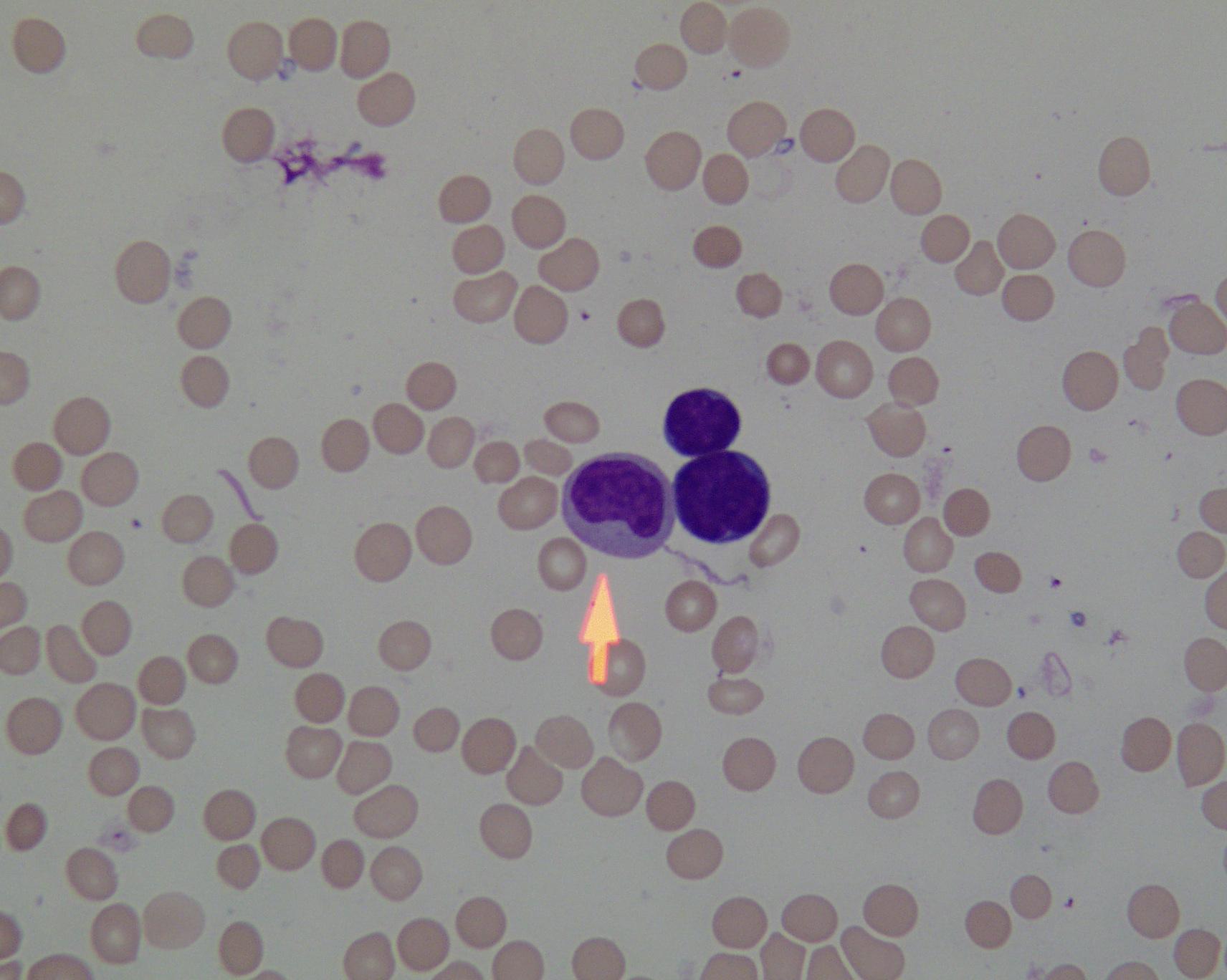

Supplement: Supplementary file 1 — Supplementary Information 1. [file 41598_2025_96918_MOESM1_ESM.zip › ALL_IDB Dataset/L2/Im003_14.jpg]

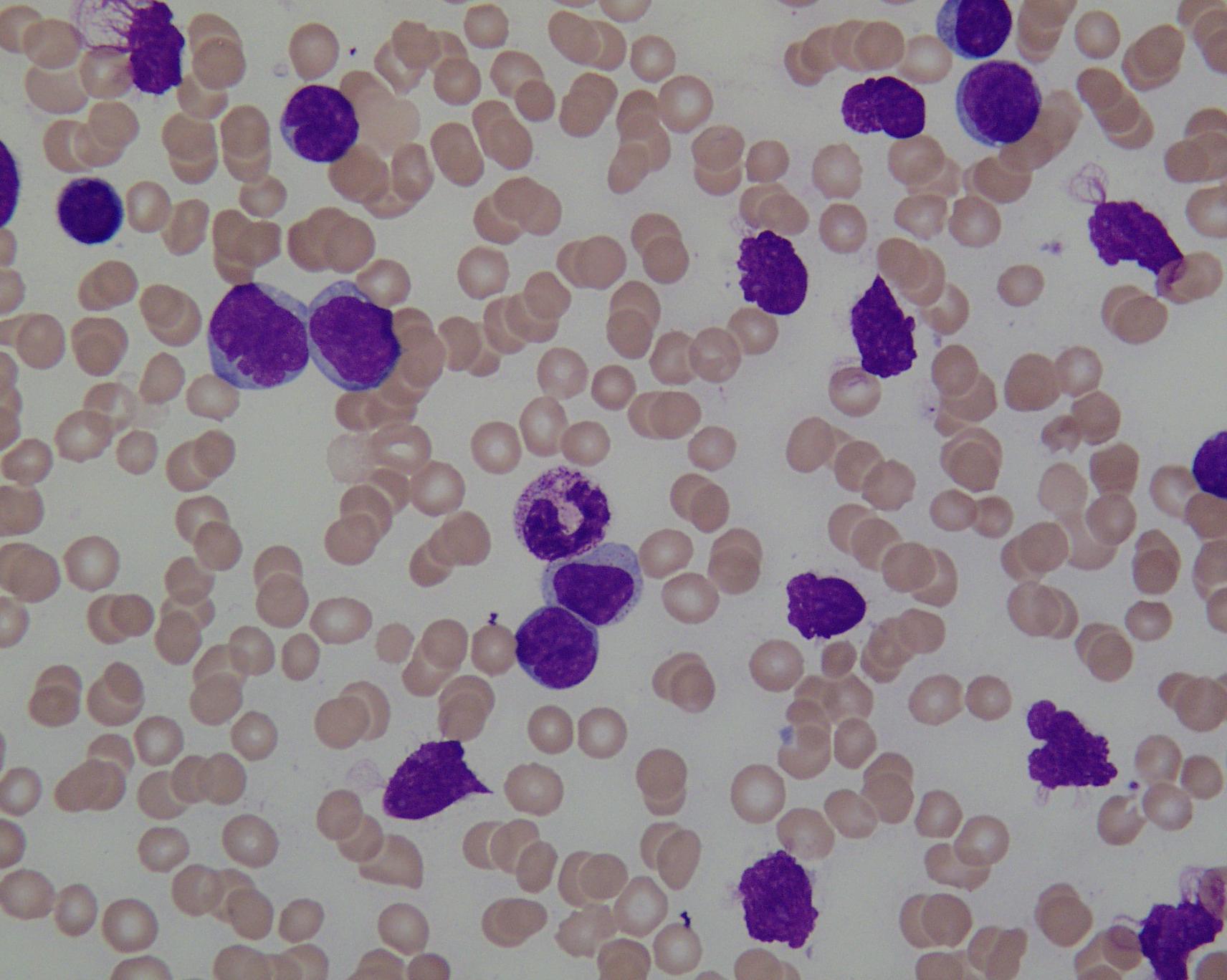

Supplement: Supplementary file 1 — Supplementary Information 1. [file 41598_2025_96918_MOESM1_ESM.zip › ALL_IDB Dataset/L2/Im003_15.jpg]

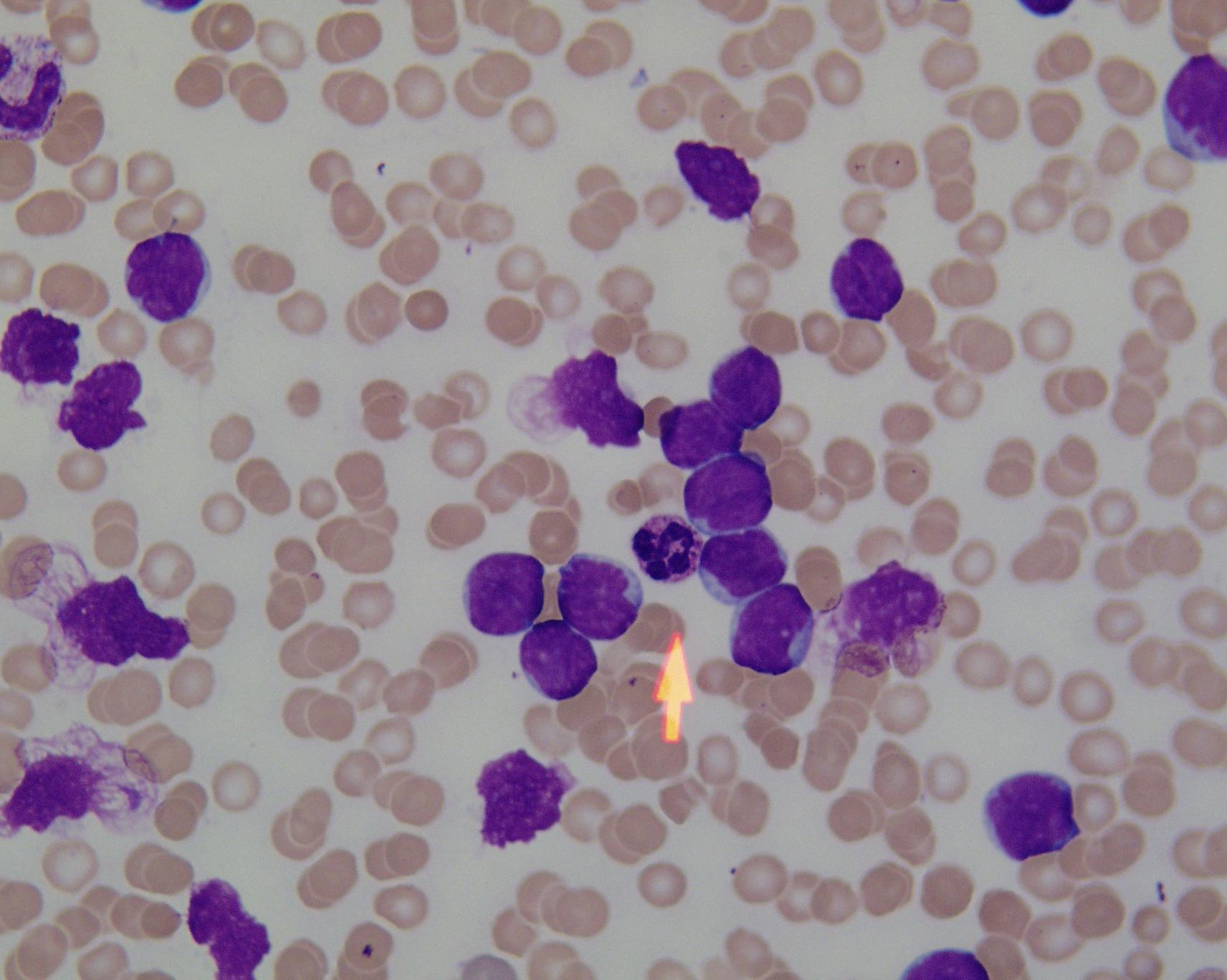

Supplement: Supplementary file 1 — Supplementary Information 1. [file 41598_2025_96918_MOESM1_ESM.zip › ALL_IDB Dataset/L2/Im003_16.jpg]

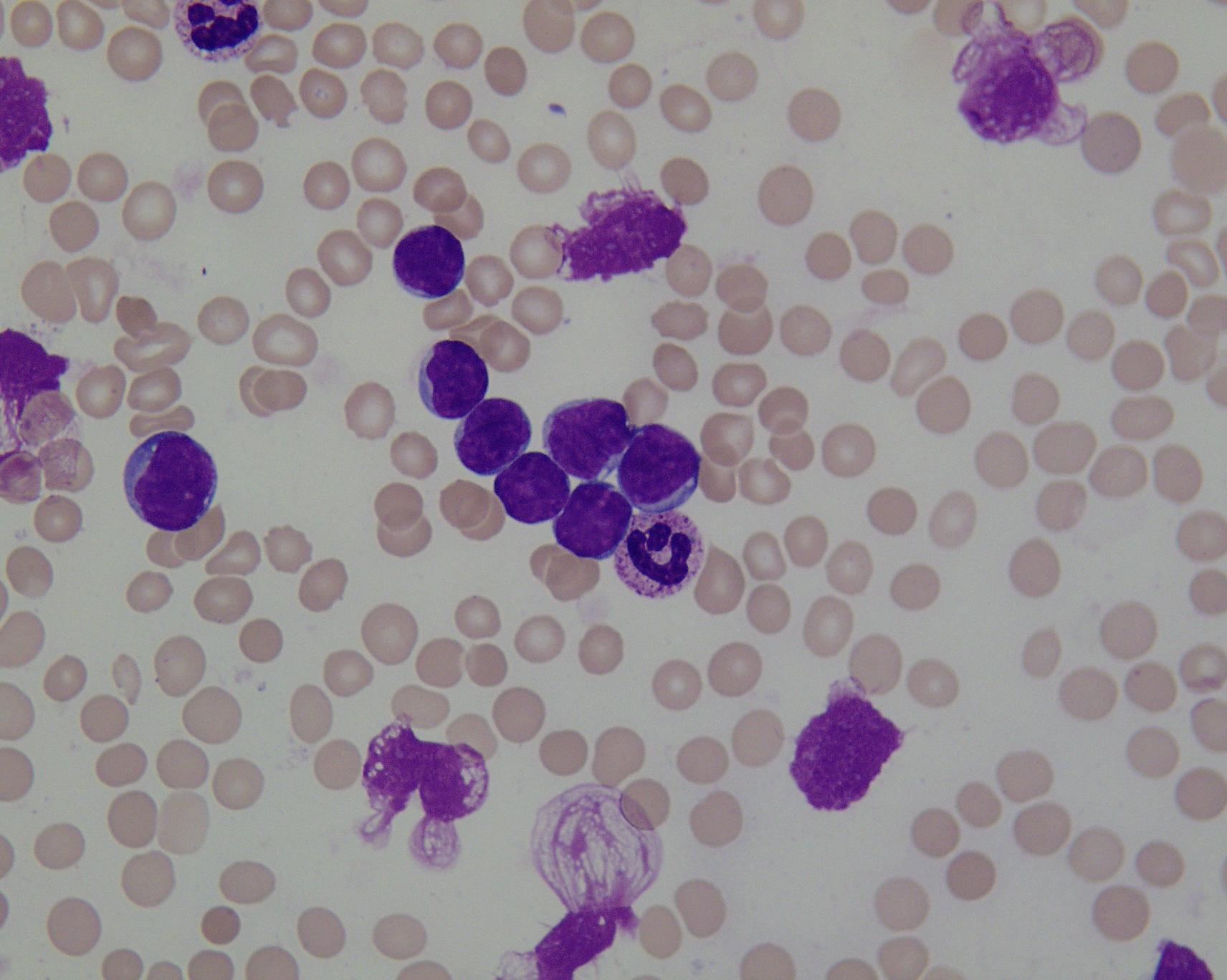

Supplement: Supplementary file 1 — Supplementary Information 1. [file 41598_2025_96918_MOESM1_ESM.zip › ALL_IDB Dataset/L2/Im003_2.jpg]

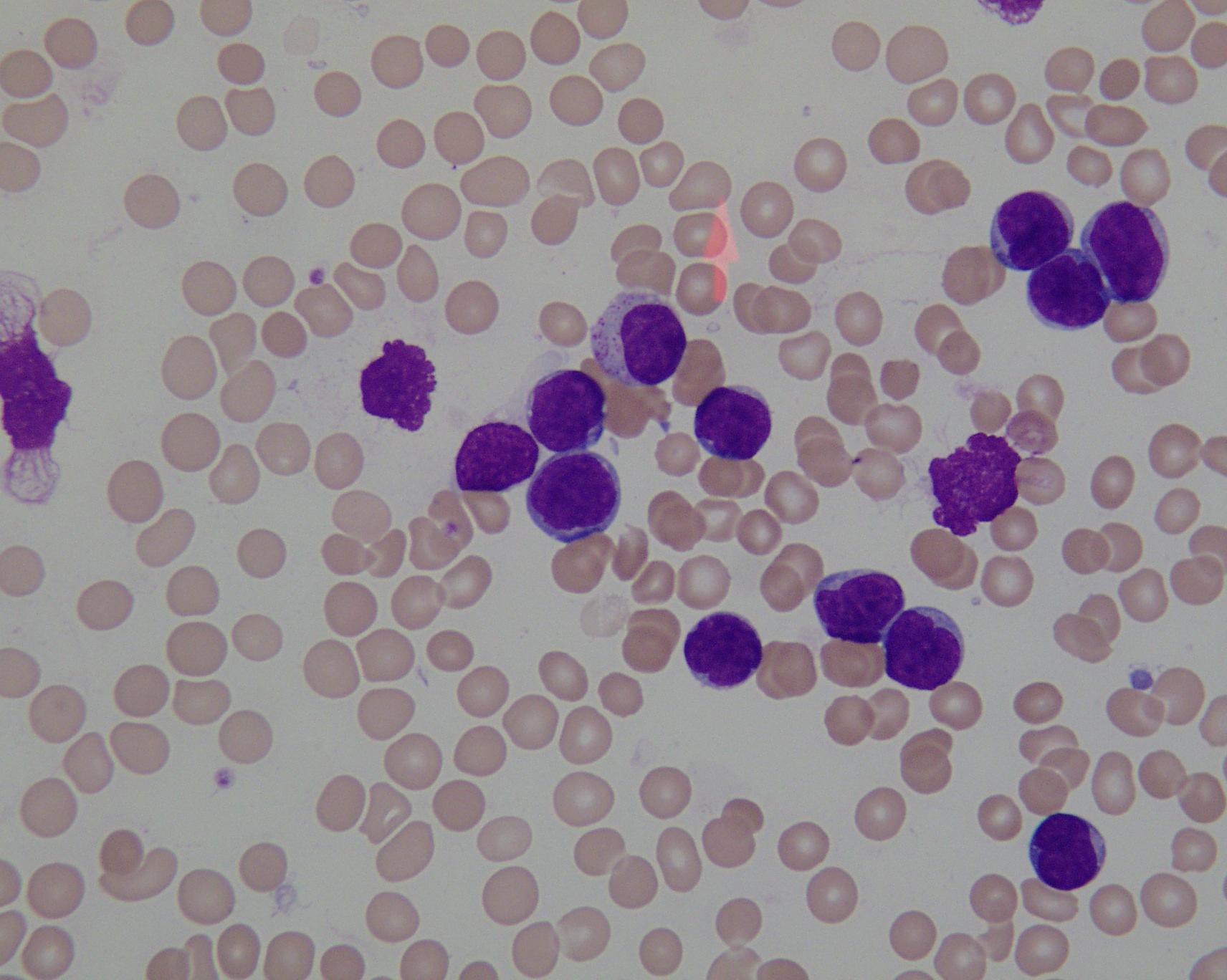

Supplement: Supplementary file 1 — Supplementary Information 1. [file 41598_2025_96918_MOESM1_ESM.zip › ALL_IDB Dataset/L2/Im003_4.jpg]

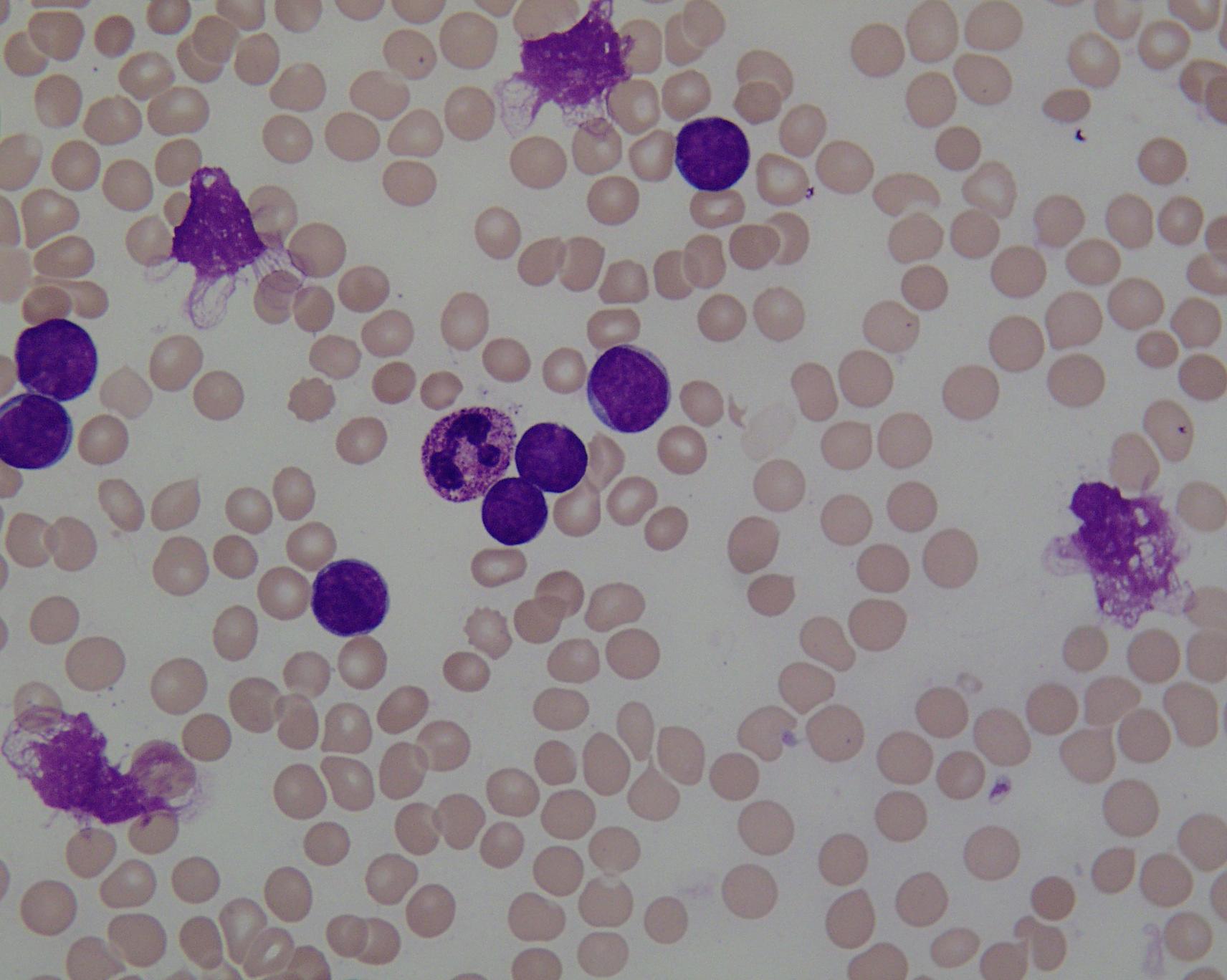

Supplement: Supplementary file 1 — Supplementary Information 1. [file 41598_2025_96918_MOESM1_ESM.zip › ALL_IDB Dataset/L2/Im003_5.jpg]

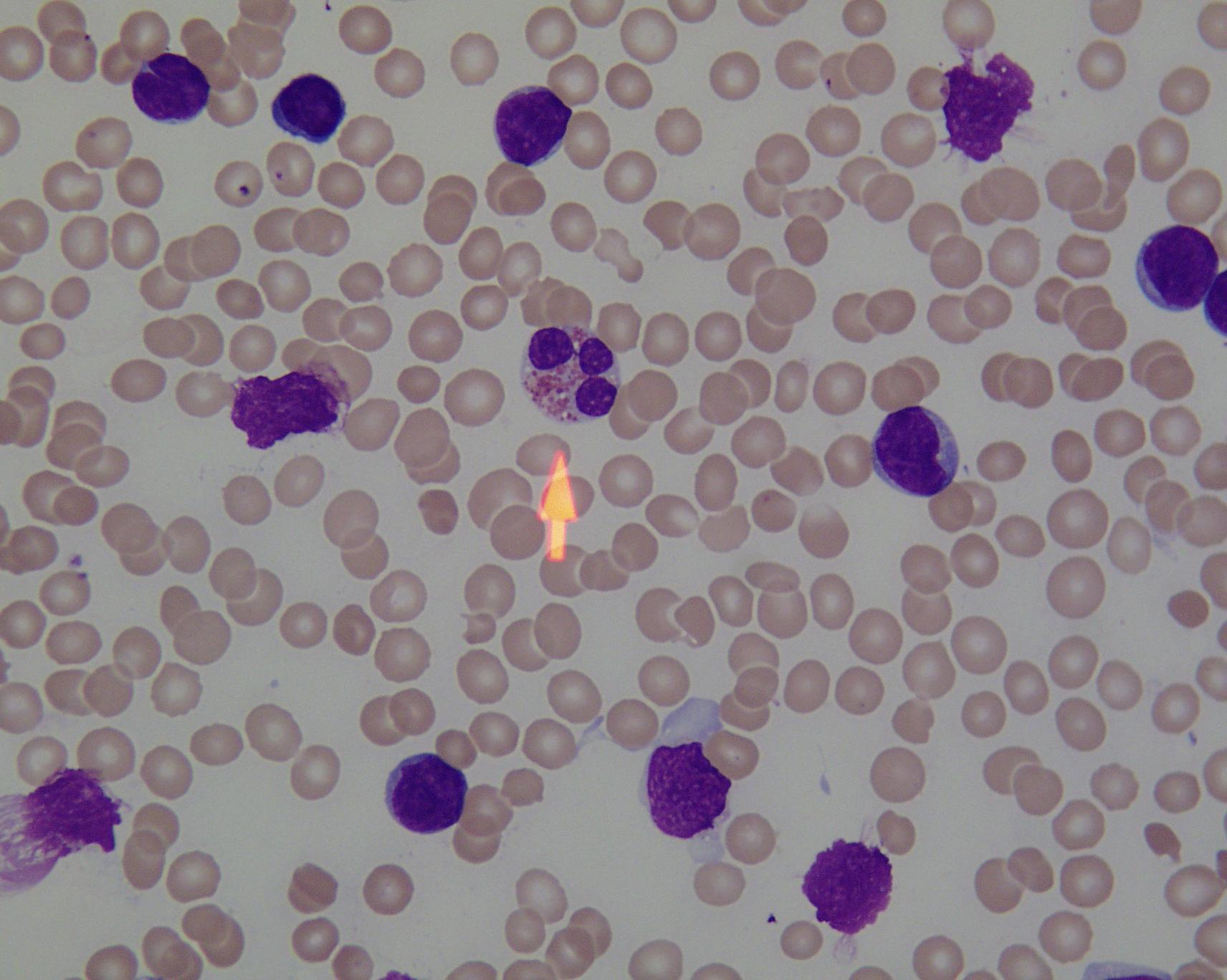

Supplement: Supplementary file 1 — Supplementary Information 1. [file 41598_2025_96918_MOESM1_ESM.zip › ALL_IDB Dataset/L2/Im003_6.jpg]

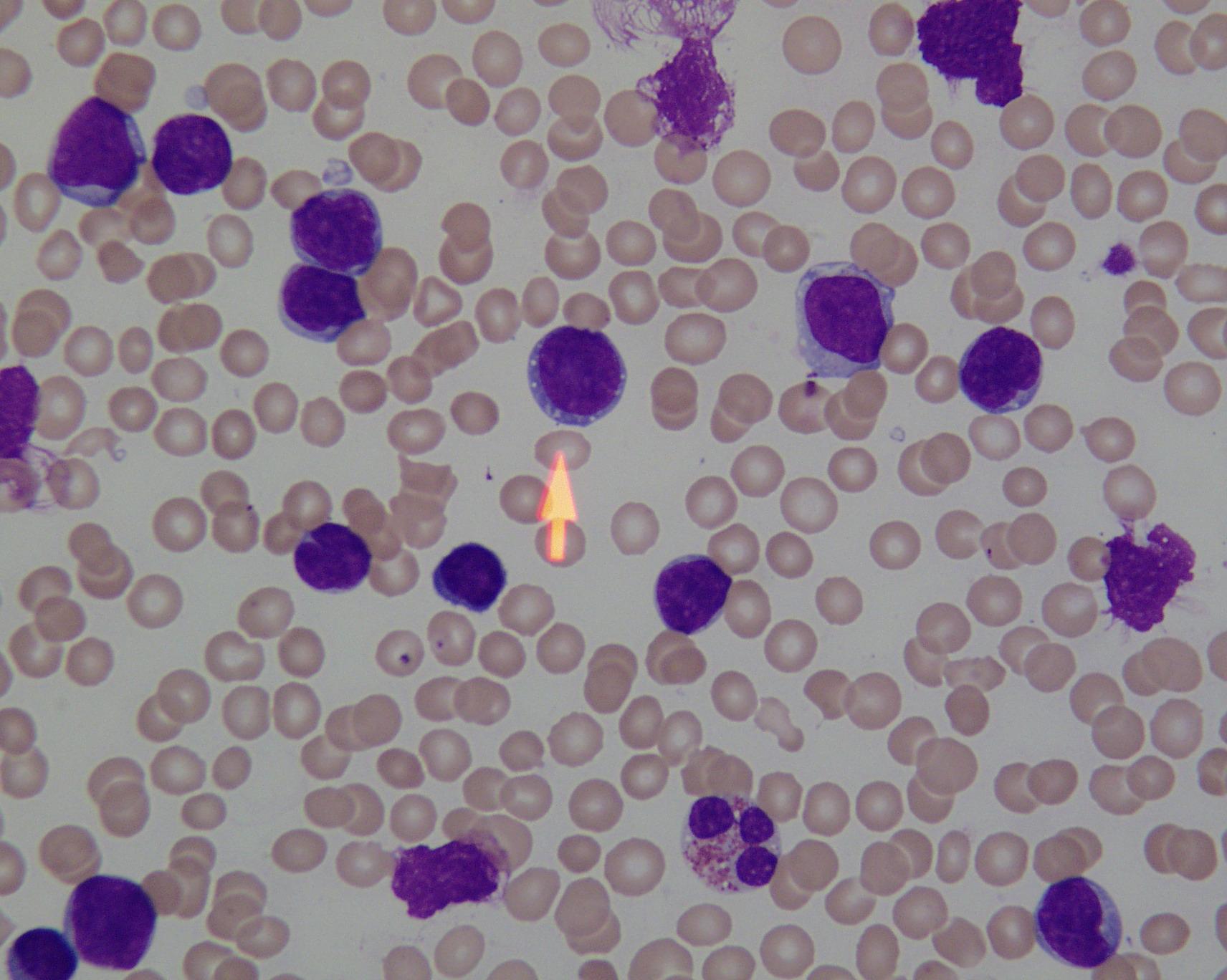

Supplement: Supplementary file 1 — Supplementary Information 1. [file 41598_2025_96918_MOESM1_ESM.zip › ALL_IDB Dataset/L2/Im003_7.jpg]

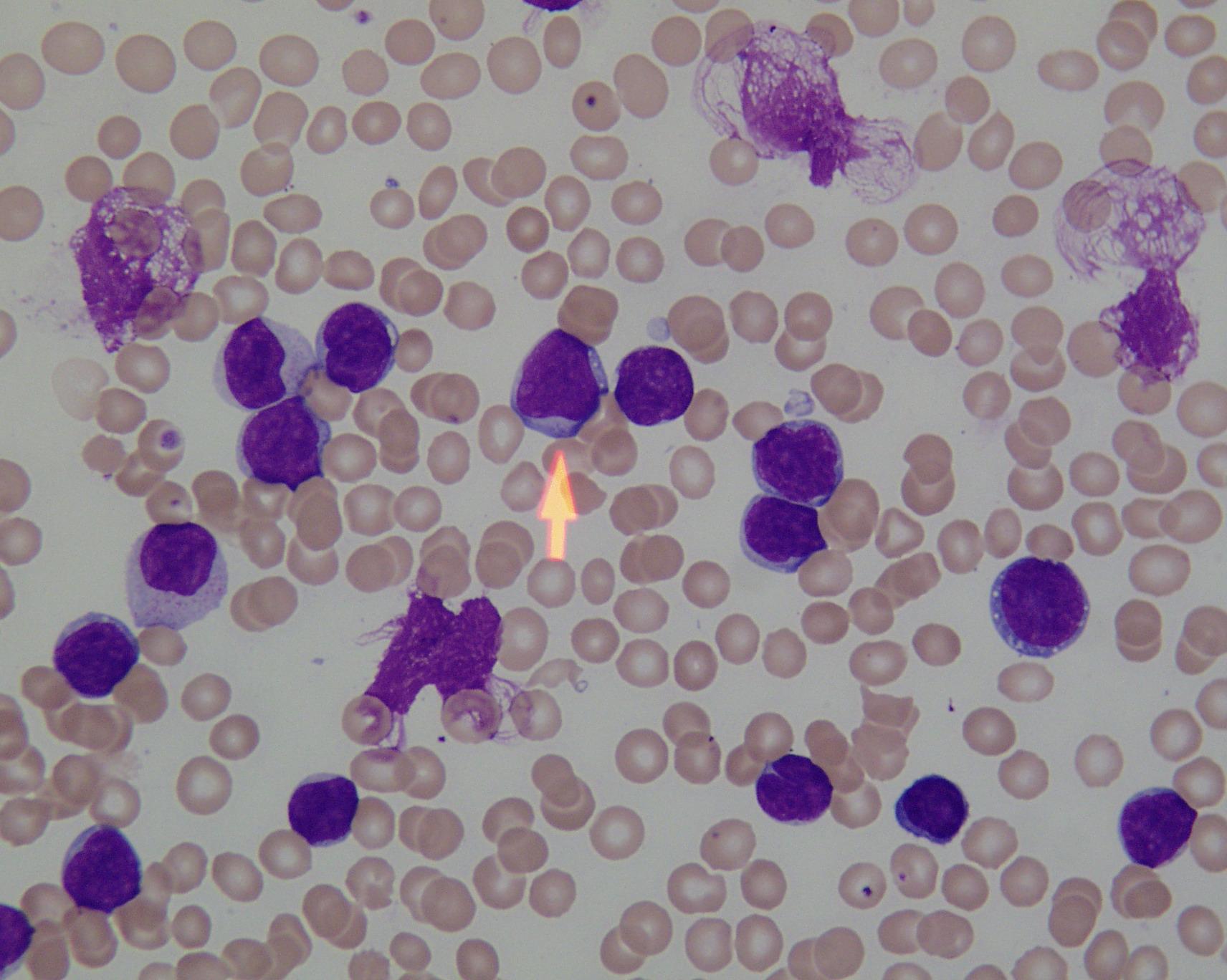

Supplement: Supplementary file 1 — Supplementary Information 1. [file 41598_2025_96918_MOESM1_ESM.zip › ALL_IDB Dataset/L2/Im003_8.jpg]

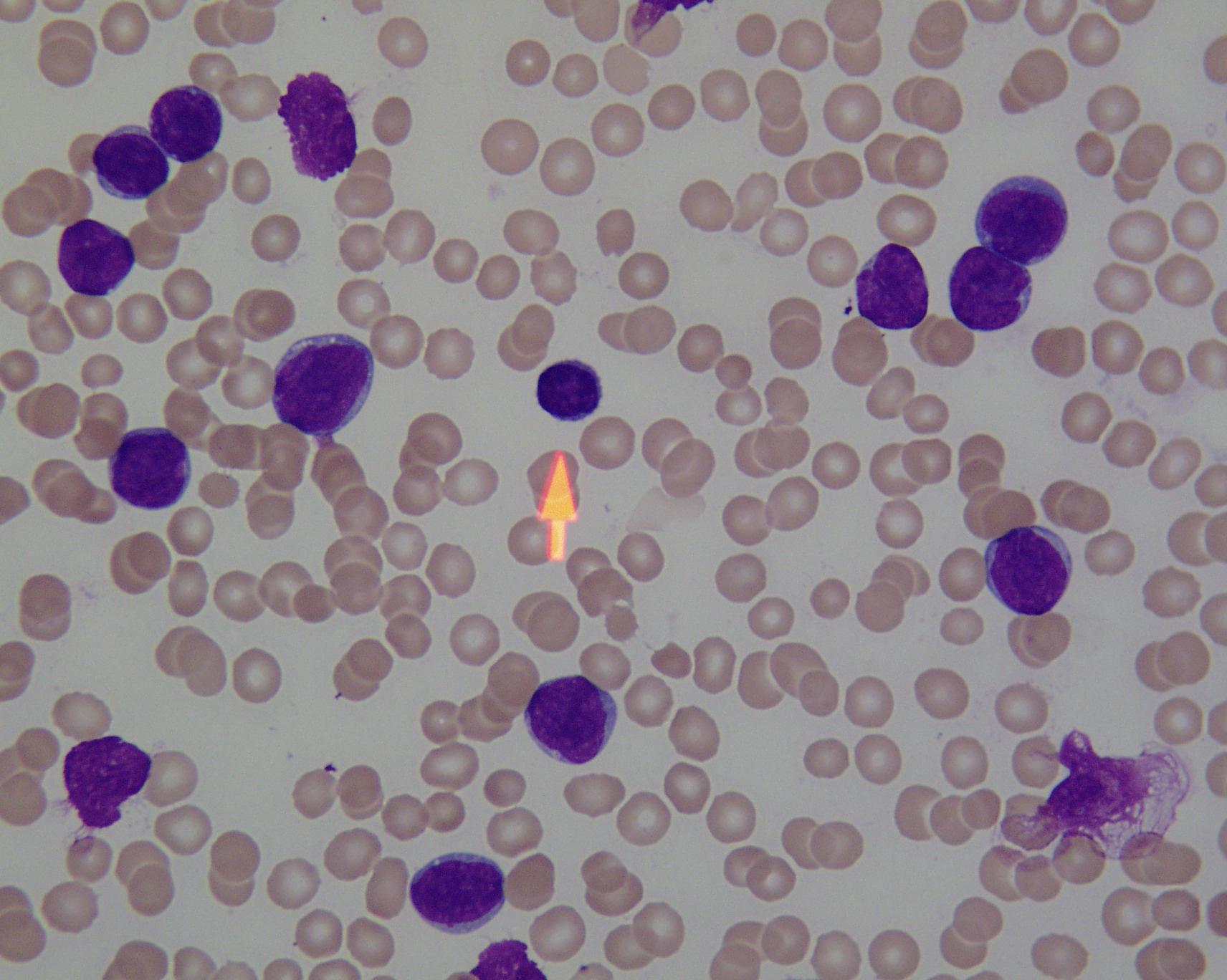

Supplement: Supplementary file 1 — Supplementary Information 1. [file 41598_2025_96918_MOESM1_ESM.zip › ALL_IDB Dataset/L2/Im003_9.jpg]

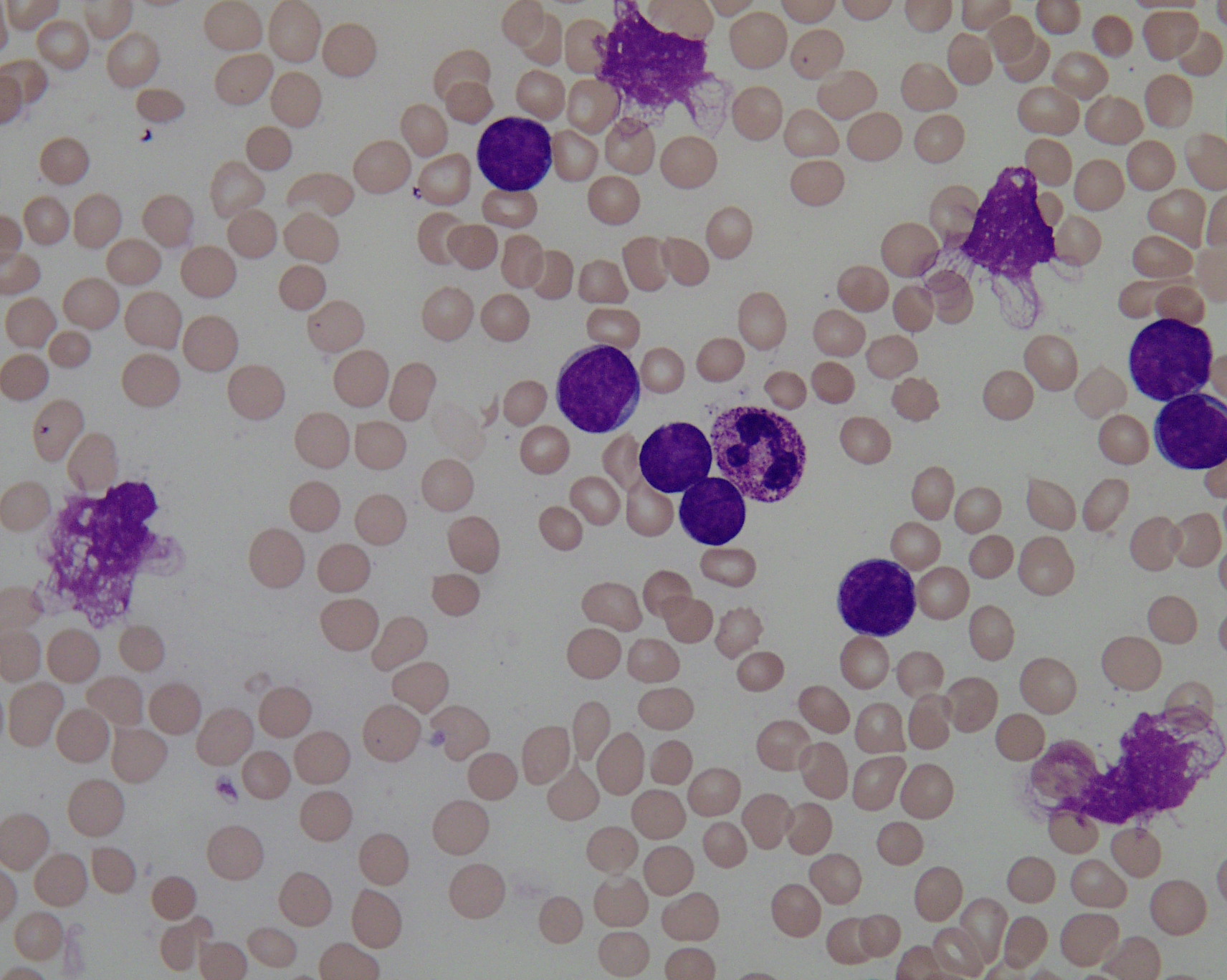

Supplement: Supplementary file 1 — Supplementary Information 1. [file 41598_2025_96918_MOESM1_ESM.zip › ALL_IDB Dataset/L2/Im004_1.jpg]

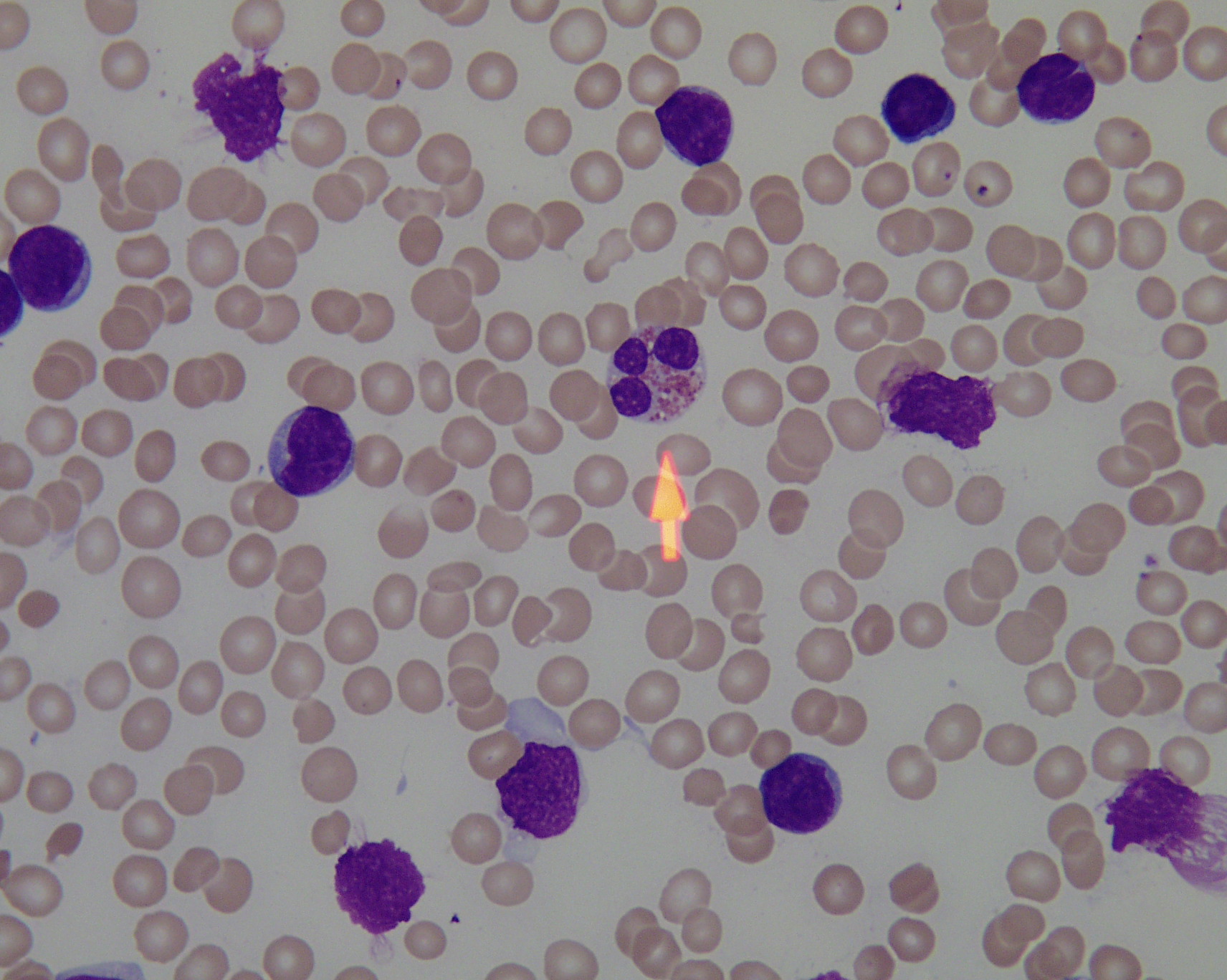

Supplement: Supplementary file 1 — Supplementary Information 1. [file 41598_2025_96918_MOESM1_ESM.zip › ALL_IDB Dataset/L2/Im009_1.jpg]

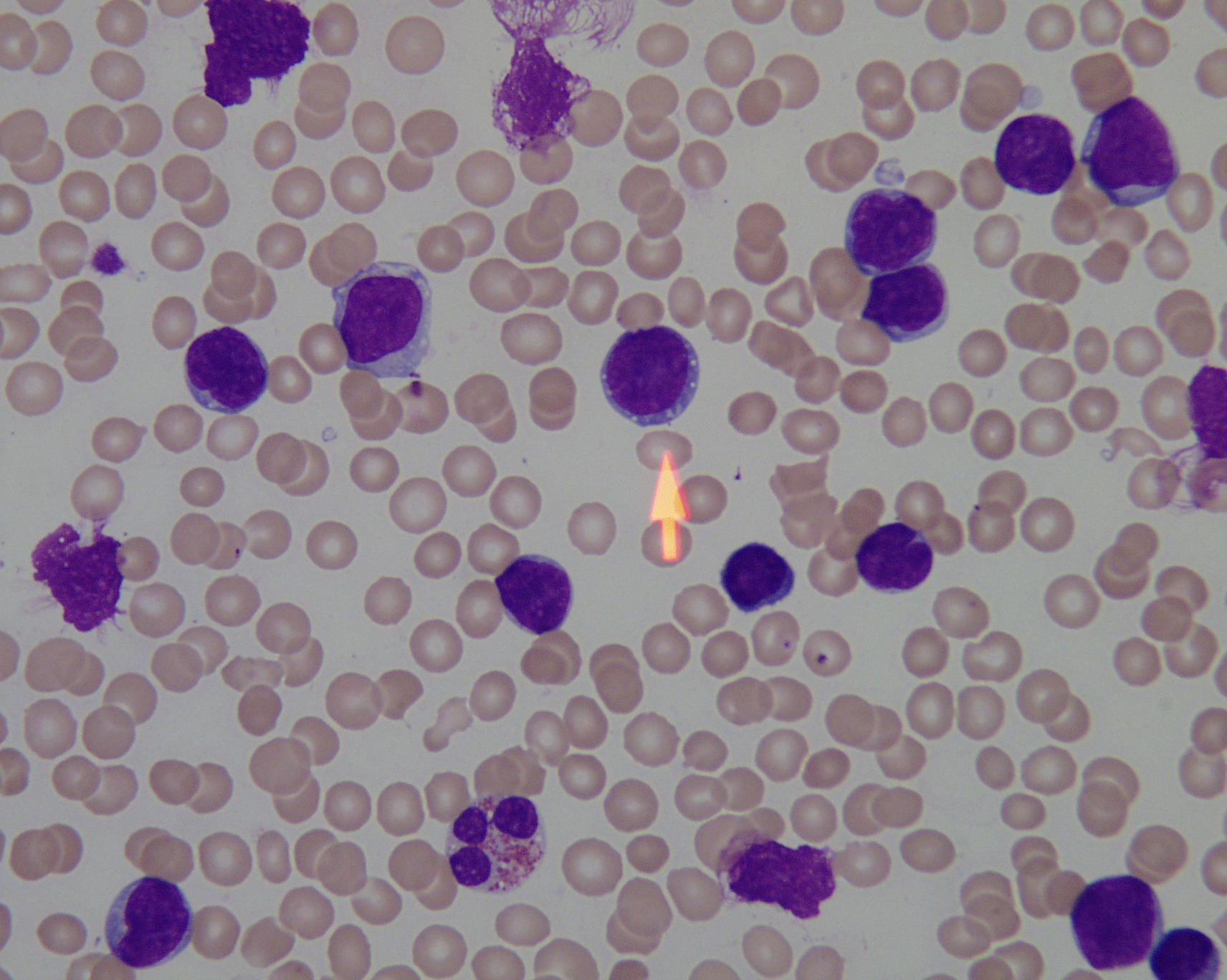

Supplement: Supplementary file 1 — Supplementary Information 1. [file 41598_2025_96918_MOESM1_ESM.zip › ALL_IDB Dataset/L2/Im010_1.jpg]

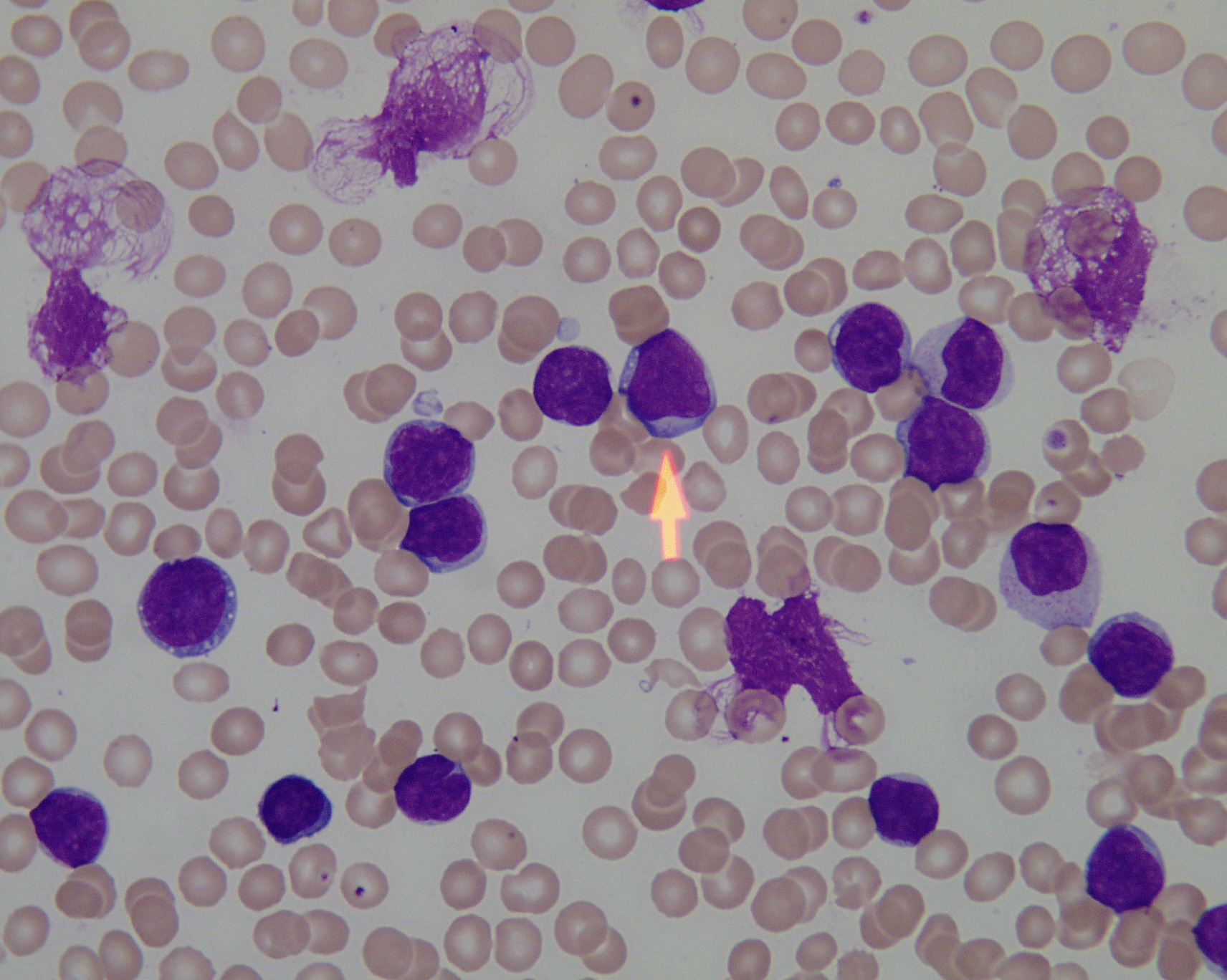

Supplement: Supplementary file 1 — Supplementary Information 1. [file 41598_2025_96918_MOESM1_ESM.zip › ALL_IDB Dataset/L2/Im011_1.jpg]

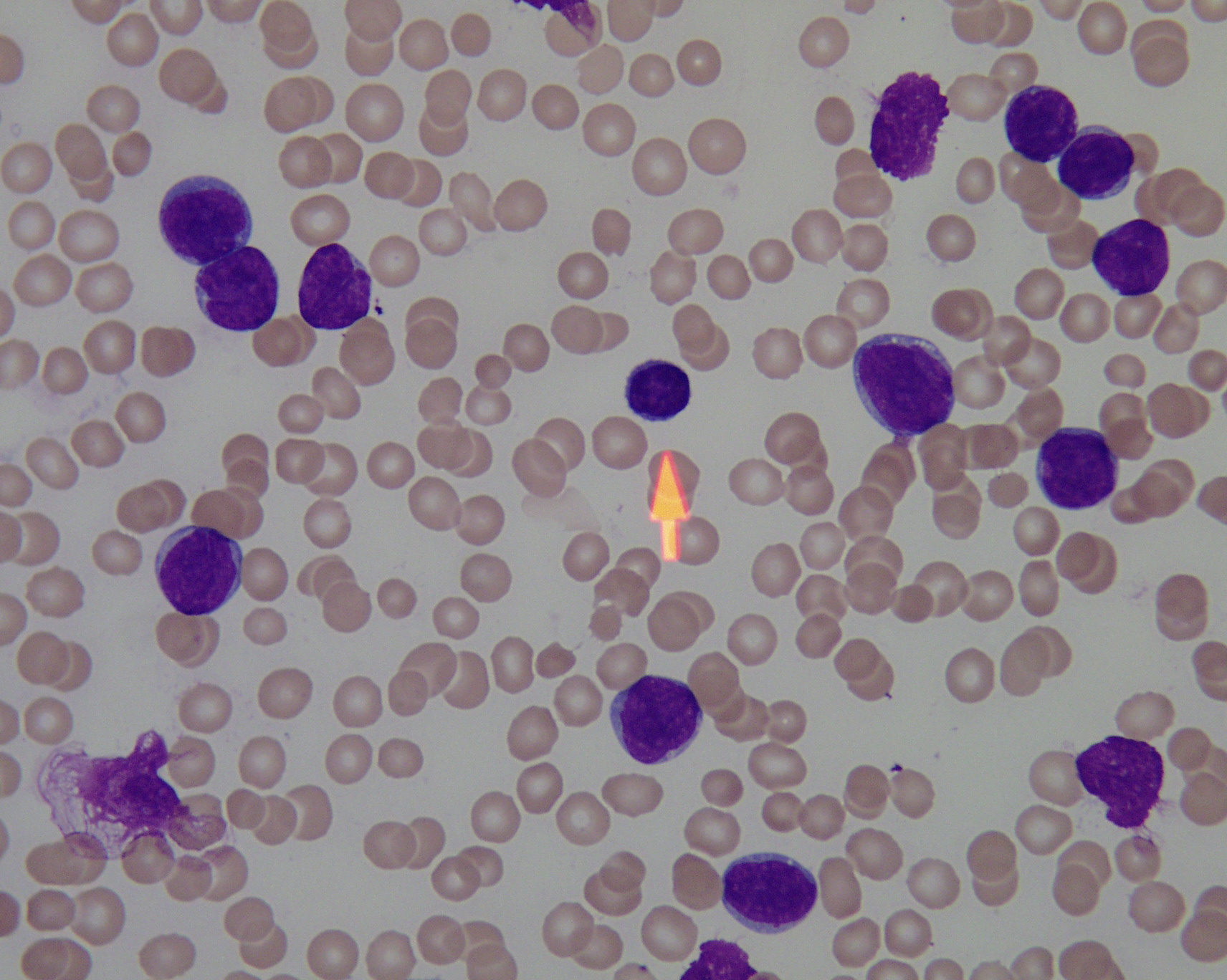

Supplement: Supplementary file 1 — Supplementary Information 1. [file 41598_2025_96918_MOESM1_ESM.zip › ALL_IDB Dataset/L2/Im012_1.jpg]

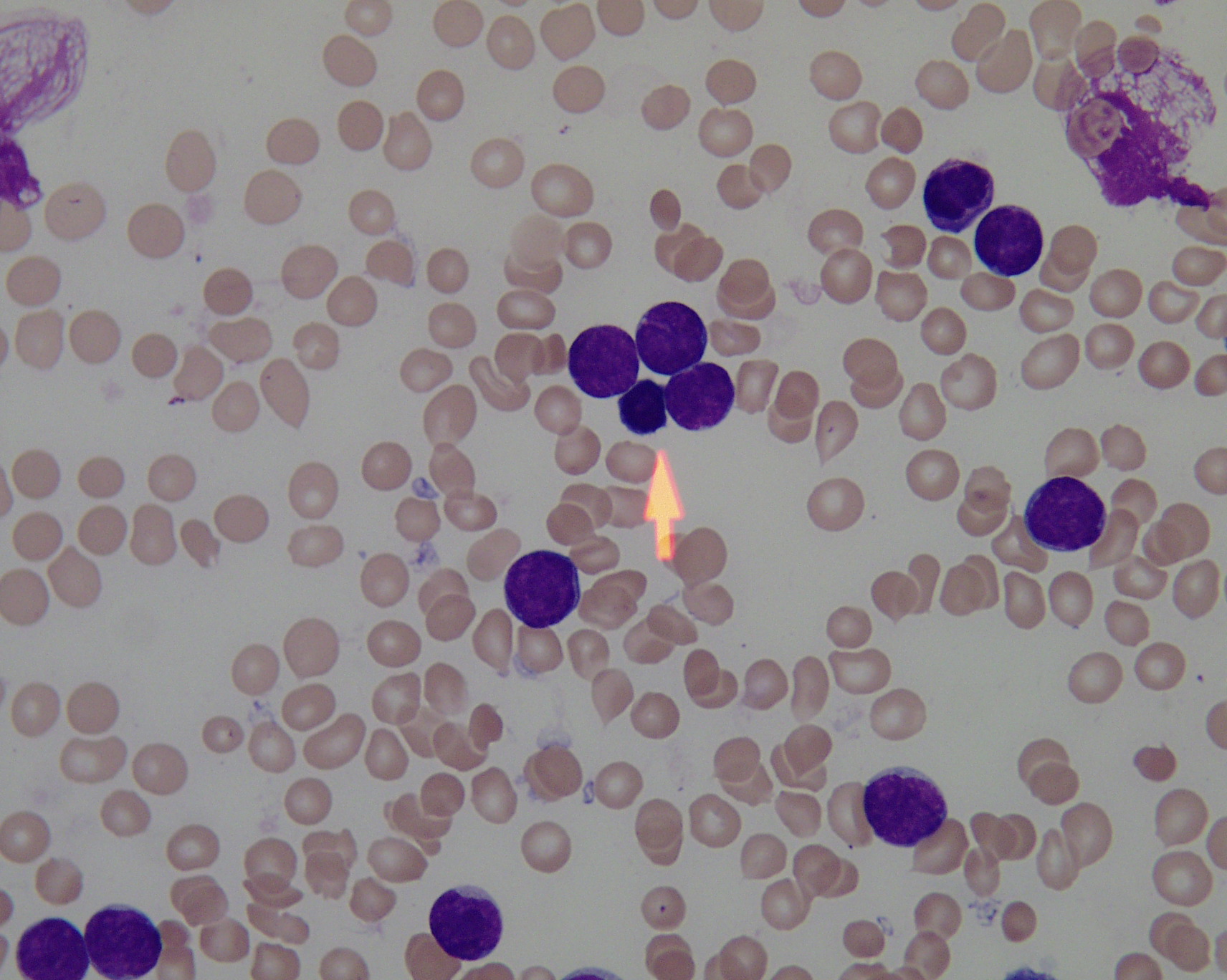

Supplement: Supplementary file 1 — Supplementary Information 1. [file 41598_2025_96918_MOESM1_ESM.zip › ALL_IDB Dataset/L2/Im013_1.jpg]

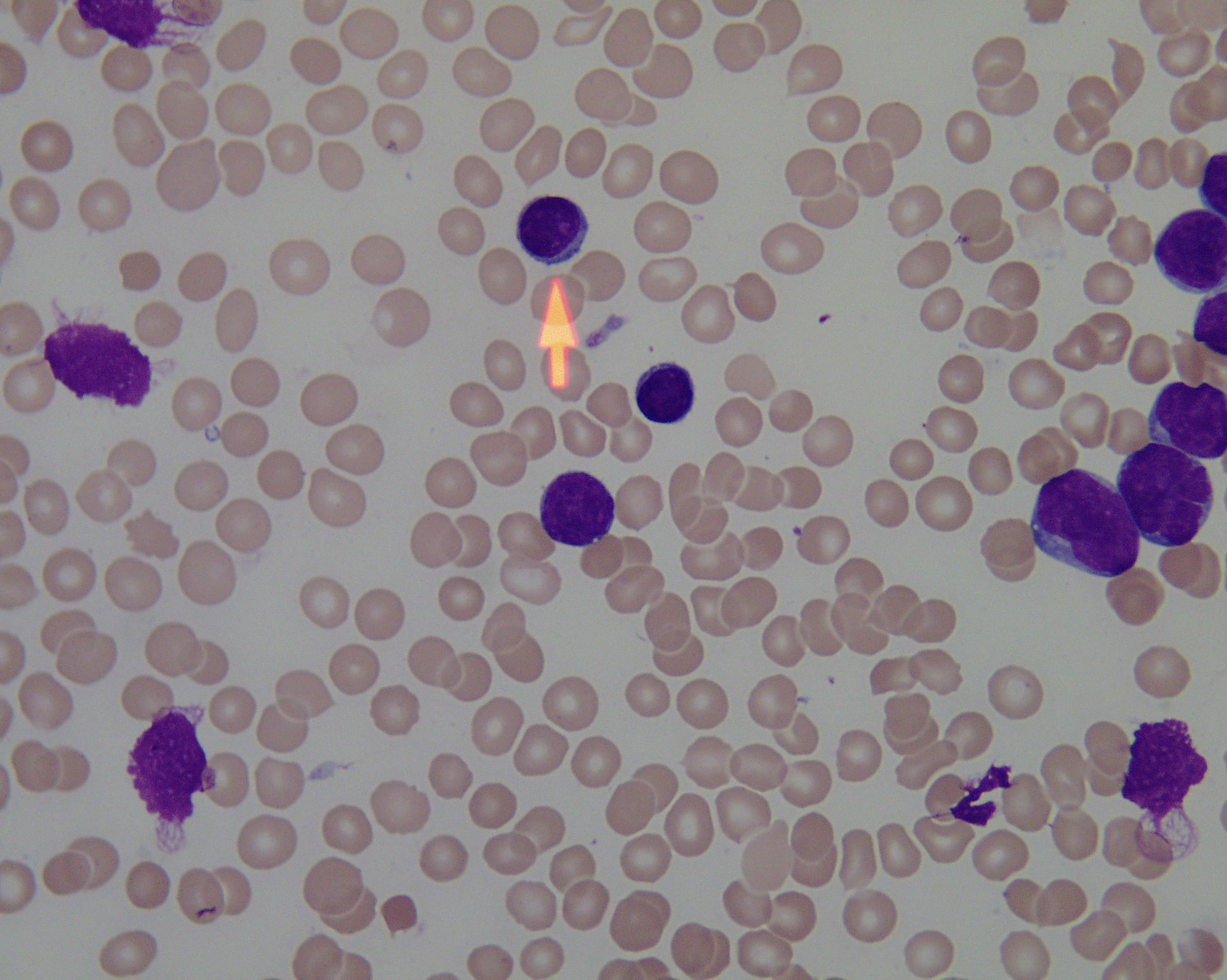

Supplement: Supplementary file 1 — Supplementary Information 1. [file 41598_2025_96918_MOESM1_ESM.zip › ALL_IDB Dataset/L2/Im014_1.jpg]

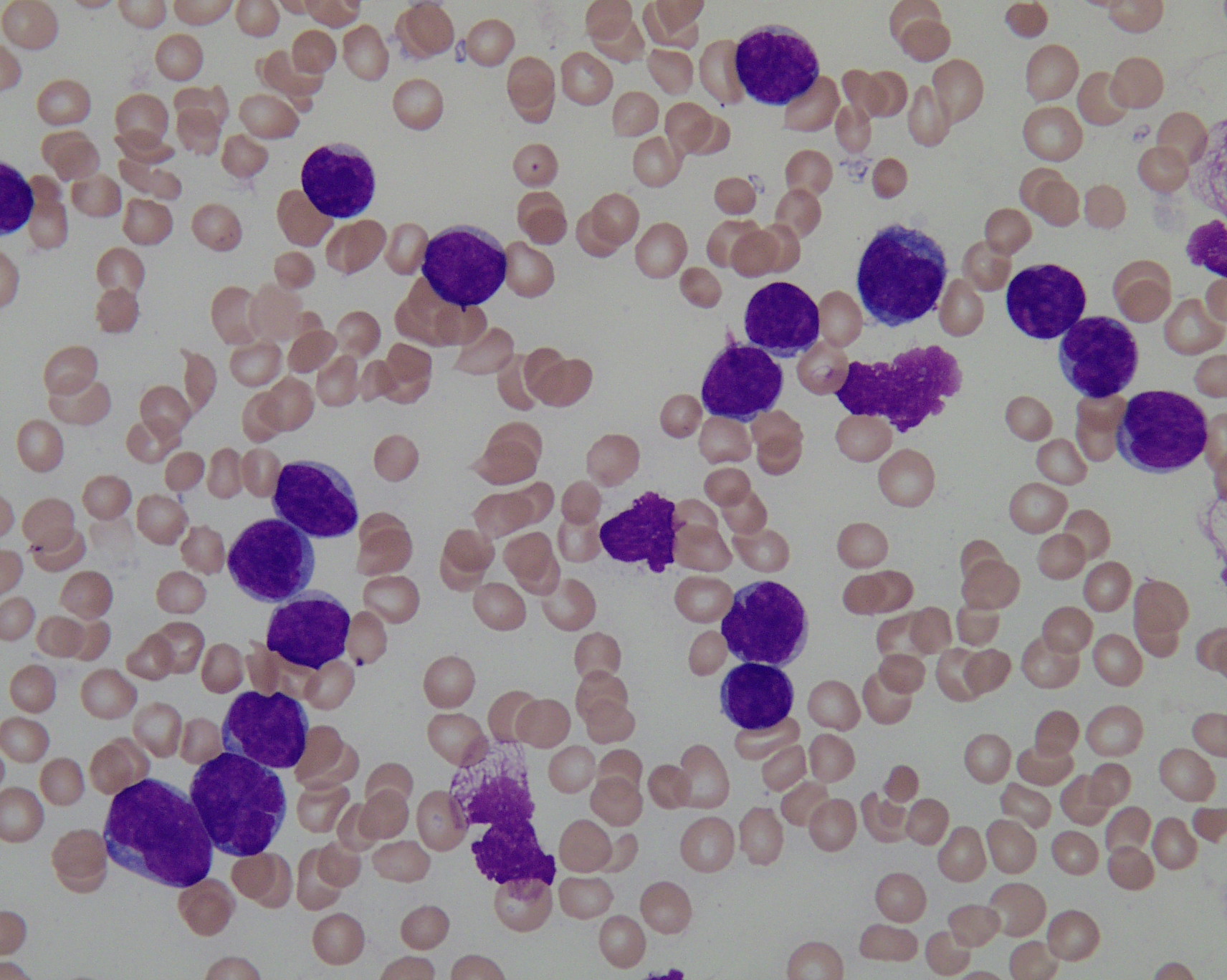

Supplement: Supplementary file 1 — Supplementary Information 1. [file 41598_2025_96918_MOESM1_ESM.zip › ALL_IDB Dataset/L2/Im015_1.jpg]

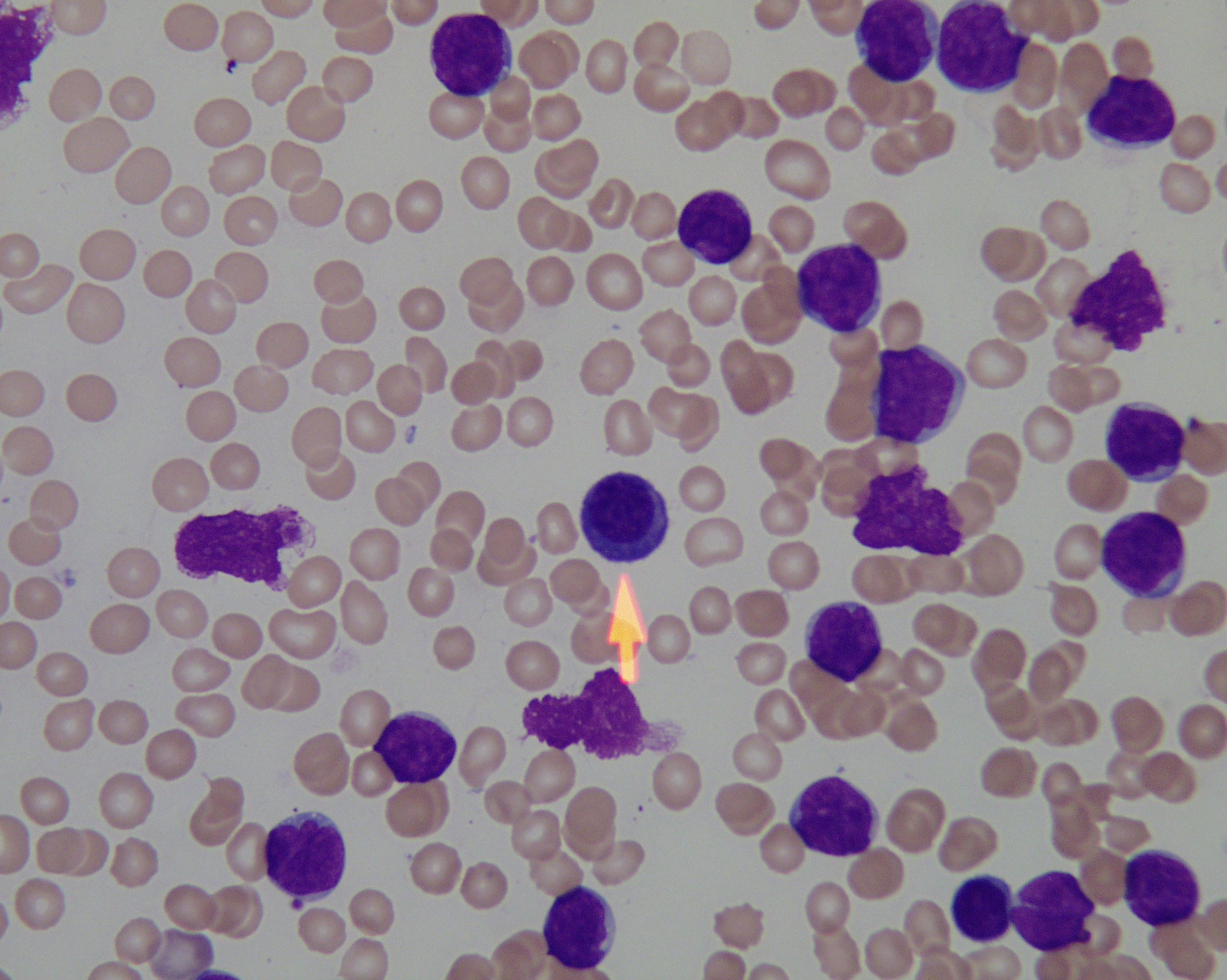

Supplement: Supplementary file 1 — Supplementary Information 1. [file 41598_2025_96918_MOESM1_ESM.zip › ALL_IDB Dataset/L2/Im016_1.jpg]

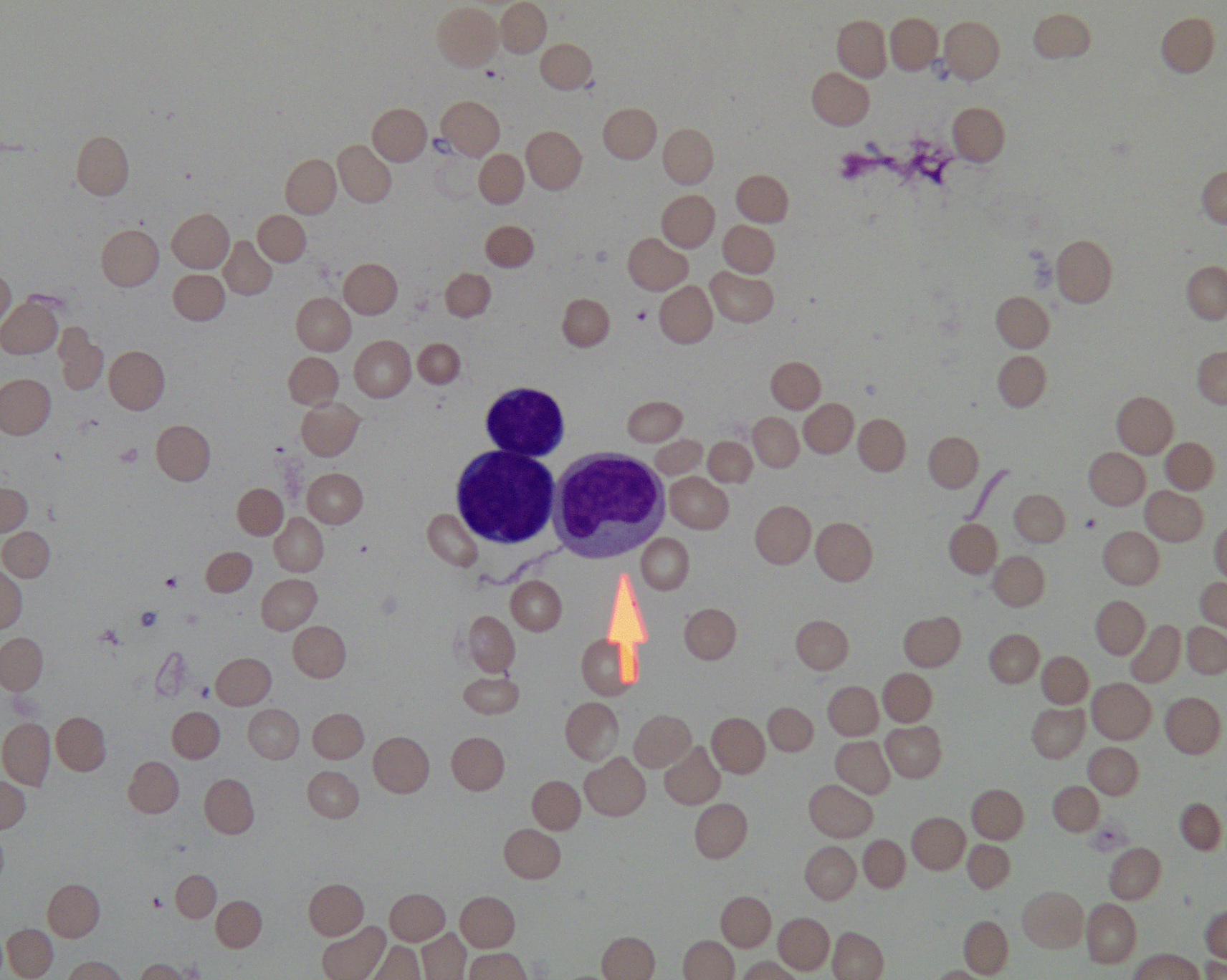

Supplement: Supplementary file 1 — Supplementary Information 1. [file 41598_2025_96918_MOESM1_ESM.zip › ALL_IDB Dataset/L2/Im017_1.jpg]

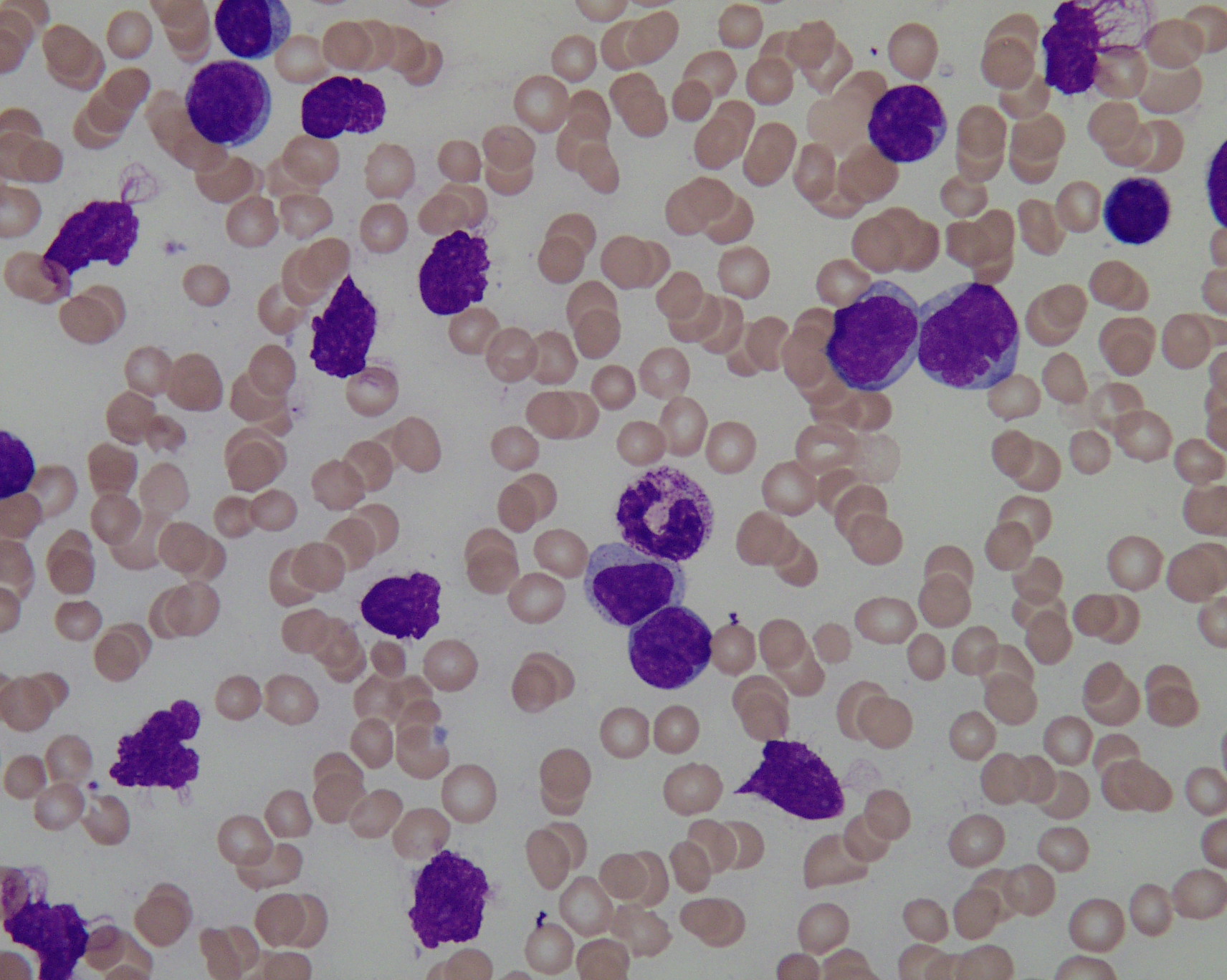

Supplement: Supplementary file 1 — Supplementary Information 1. [file 41598_2025_96918_MOESM1_ESM.zip › ALL_IDB Dataset/L2/Im018_1.jpg]

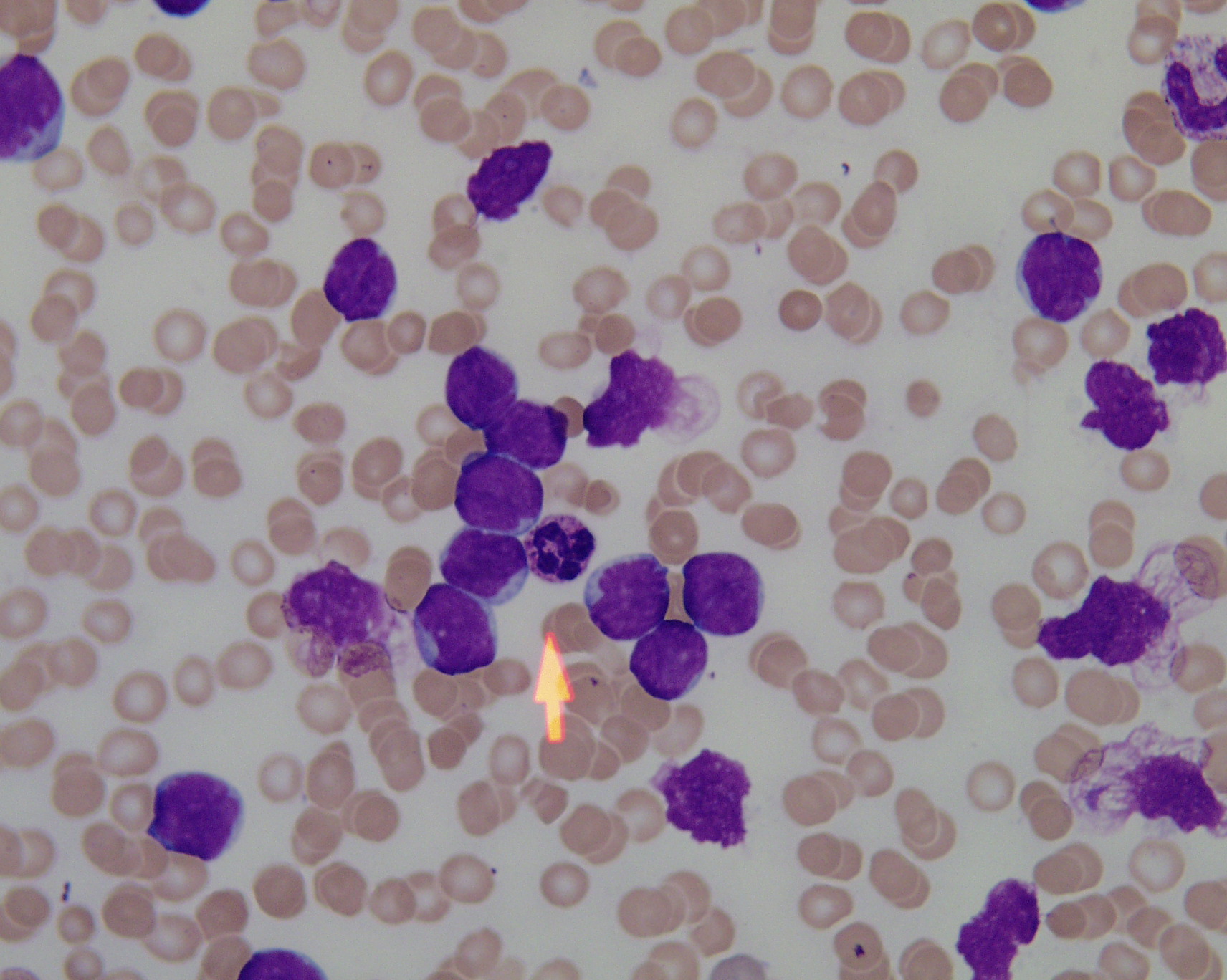

Supplement: Supplementary file 1 — Supplementary Information 1. [file 41598_2025_96918_MOESM1_ESM.zip › ALL_IDB Dataset/L2/Im019_1.jpg]

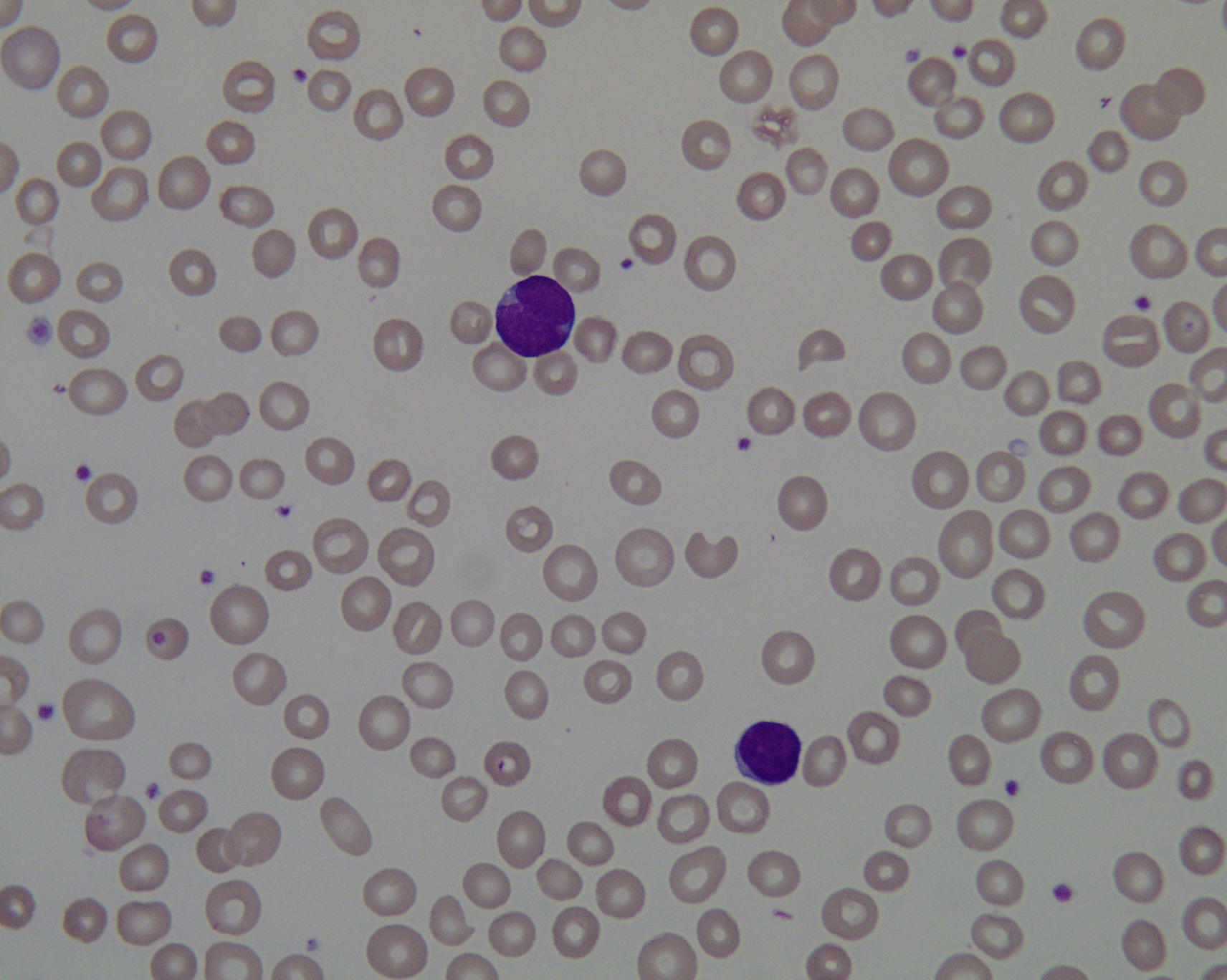

Supplement: Supplementary file 1 — Supplementary Information 1. [file 41598_2025_96918_MOESM1_ESM.zip › ALL_IDB Dataset/L2/Im020_1.jpg]

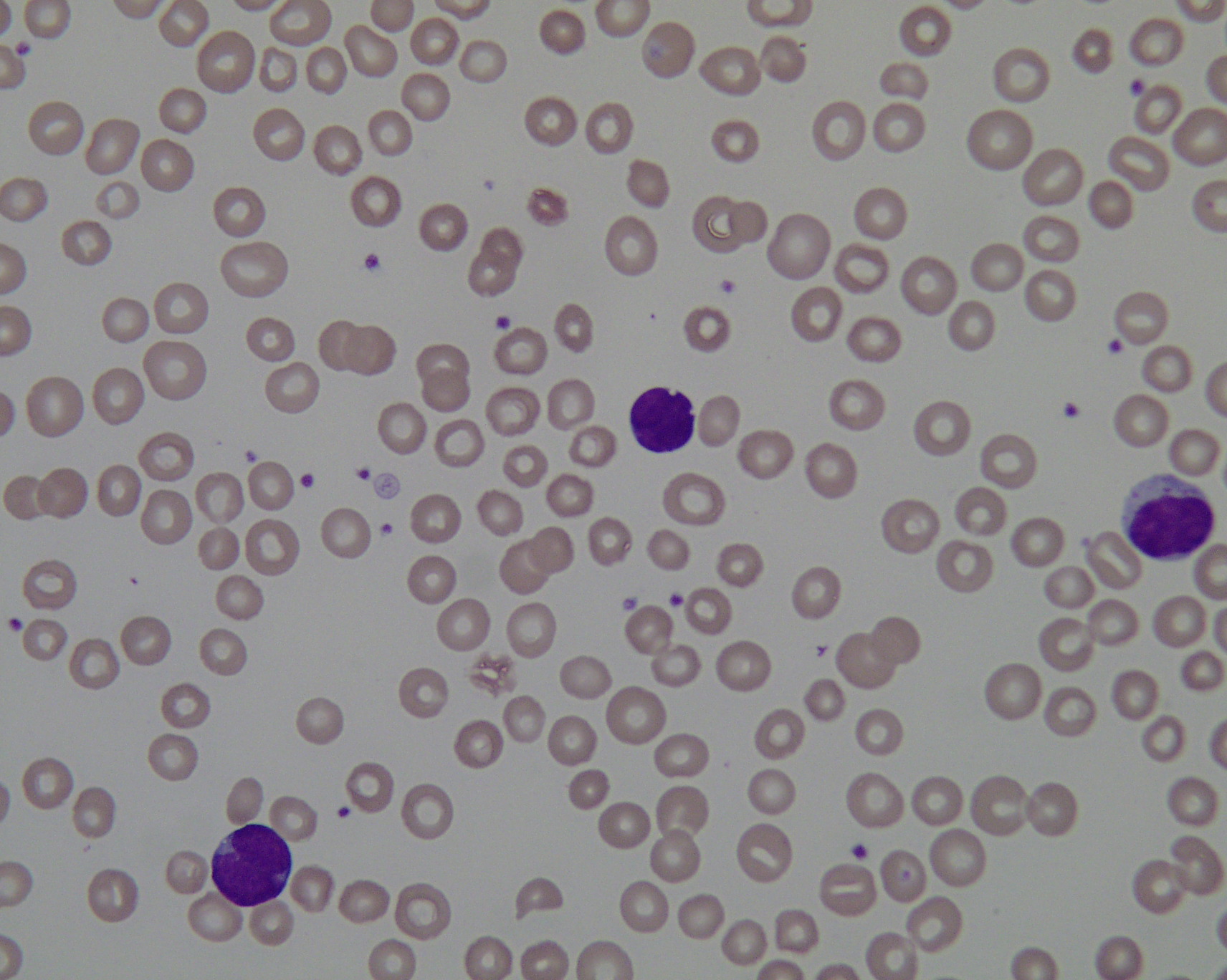

Supplement: Supplementary file 1 — Supplementary Information 1. [file 41598_2025_96918_MOESM1_ESM.zip › ALL_IDB Dataset/L2/Im0210_1.jpg]

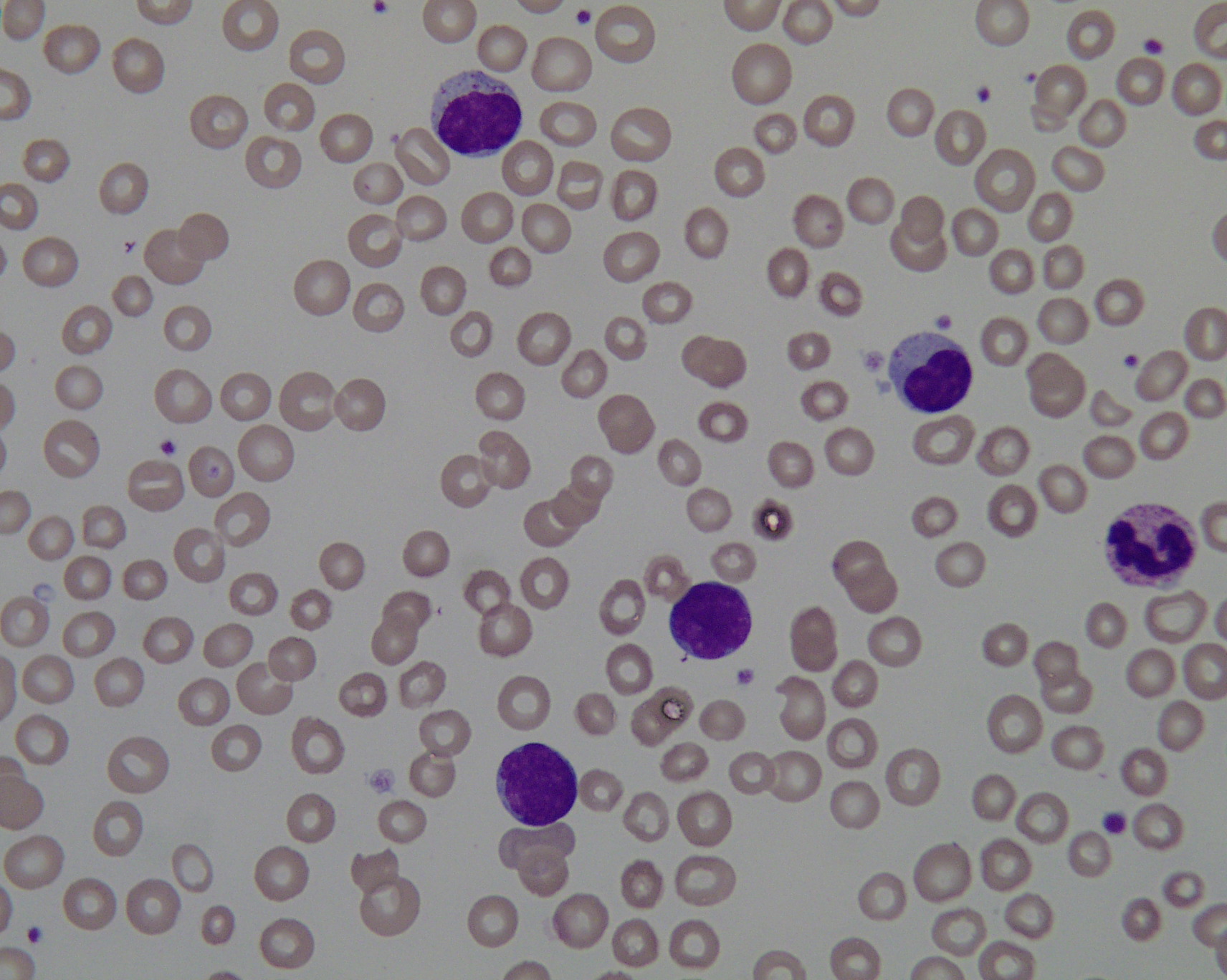

Supplement: Supplementary file 1 — Supplementary Information 1. [file 41598_2025_96918_MOESM1_ESM.zip › ALL_IDB Dataset/L2/Im022_1.jpg]

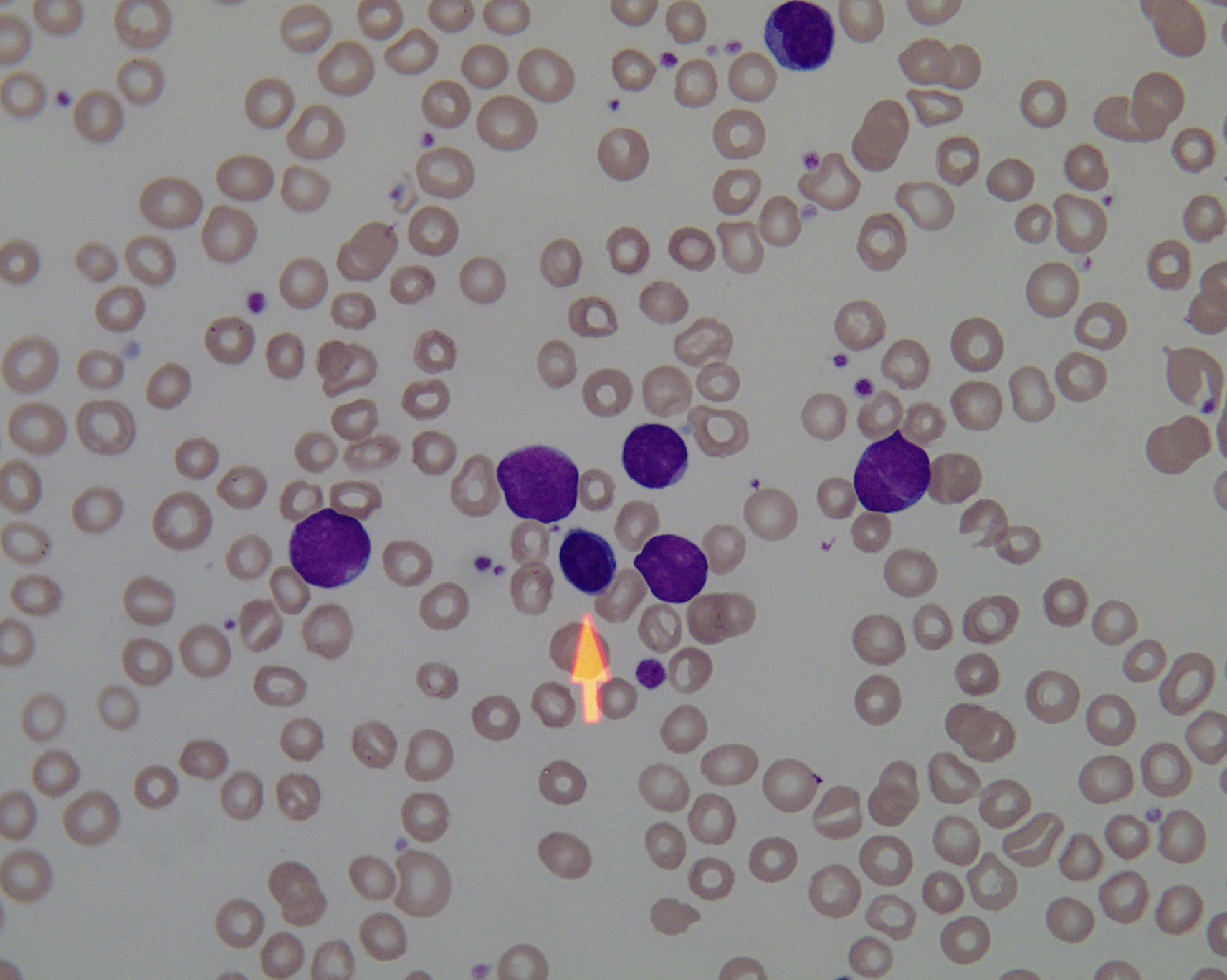

Supplement: Supplementary file 1 — Supplementary Information 1. [file 41598_2025_96918_MOESM1_ESM.zip › ALL_IDB Dataset/L2/Im0230_1.jpg]

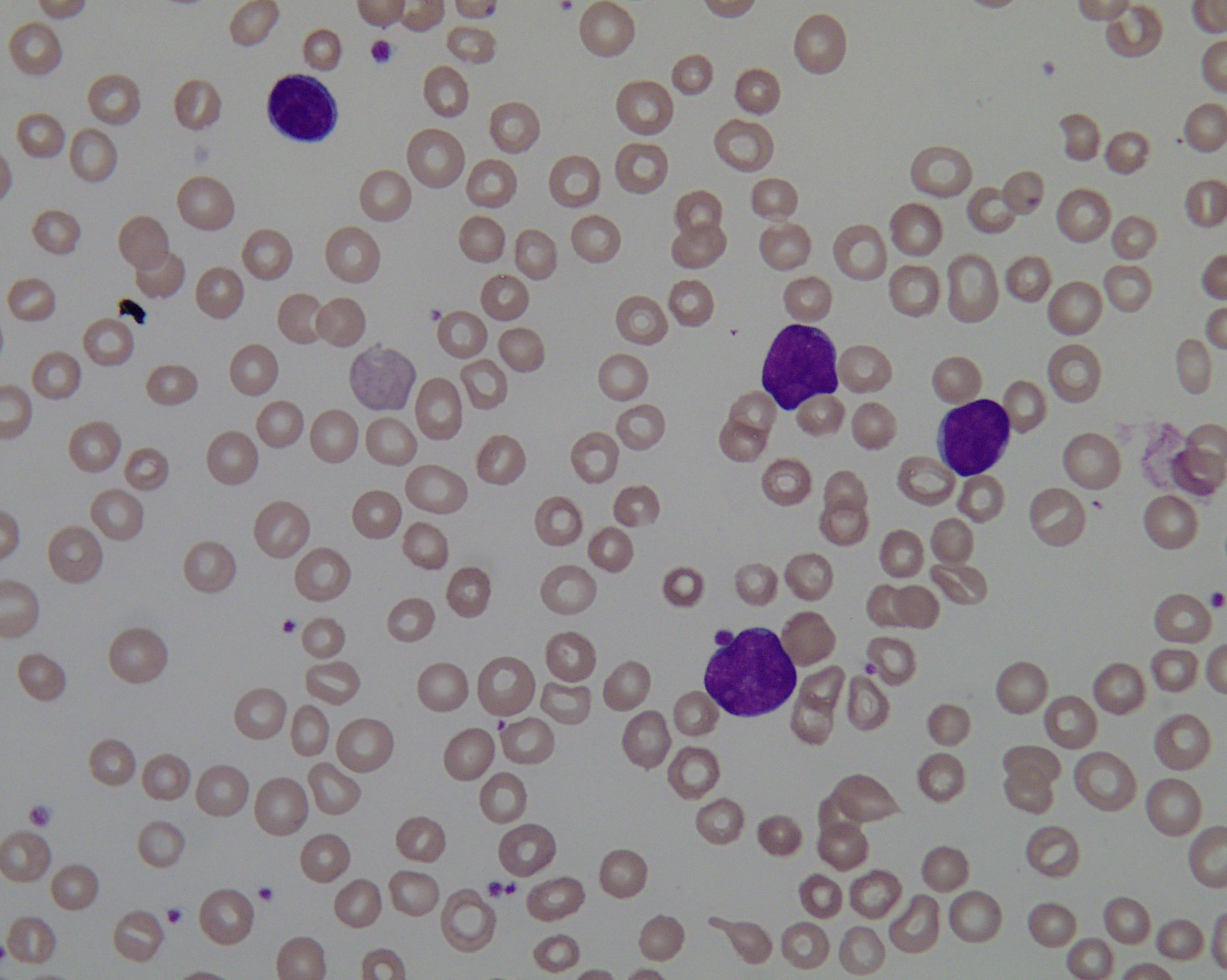

Supplement: Supplementary file 1 — Supplementary Information 1. [file 41598_2025_96918_MOESM1_ESM.zip › ALL_IDB Dataset/L2/Im0241_1.jpg]

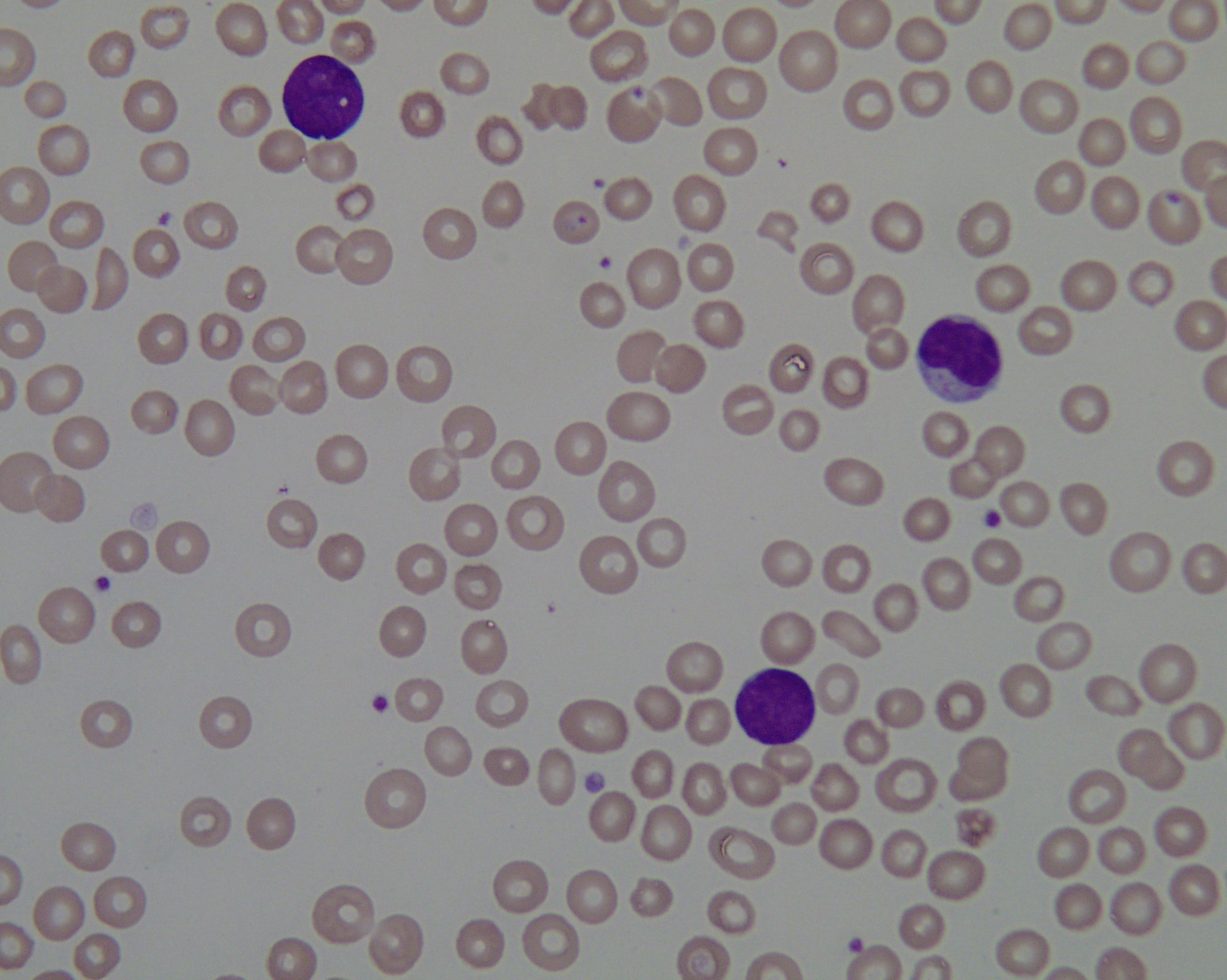

Supplement: Supplementary file 1 — Supplementary Information 1. [file 41598_2025_96918_MOESM1_ESM.zip › ALL_IDB Dataset/L2/Im0251_1.jpg]

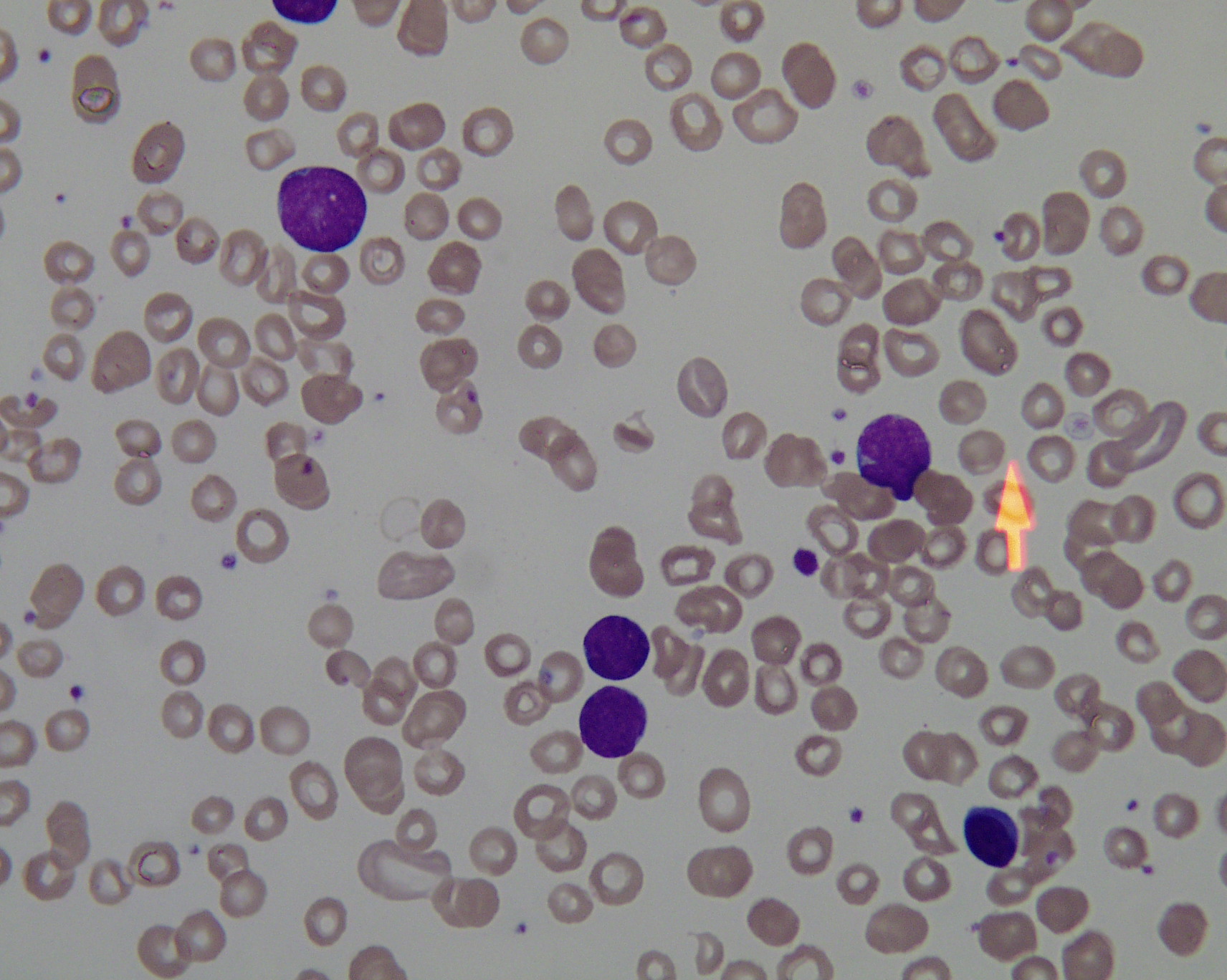

Supplement: Supplementary file 1 — Supplementary Information 1. [file 41598_2025_96918_MOESM1_ESM.zip › ALL_IDB Dataset/L2/Im026_1.jpg]

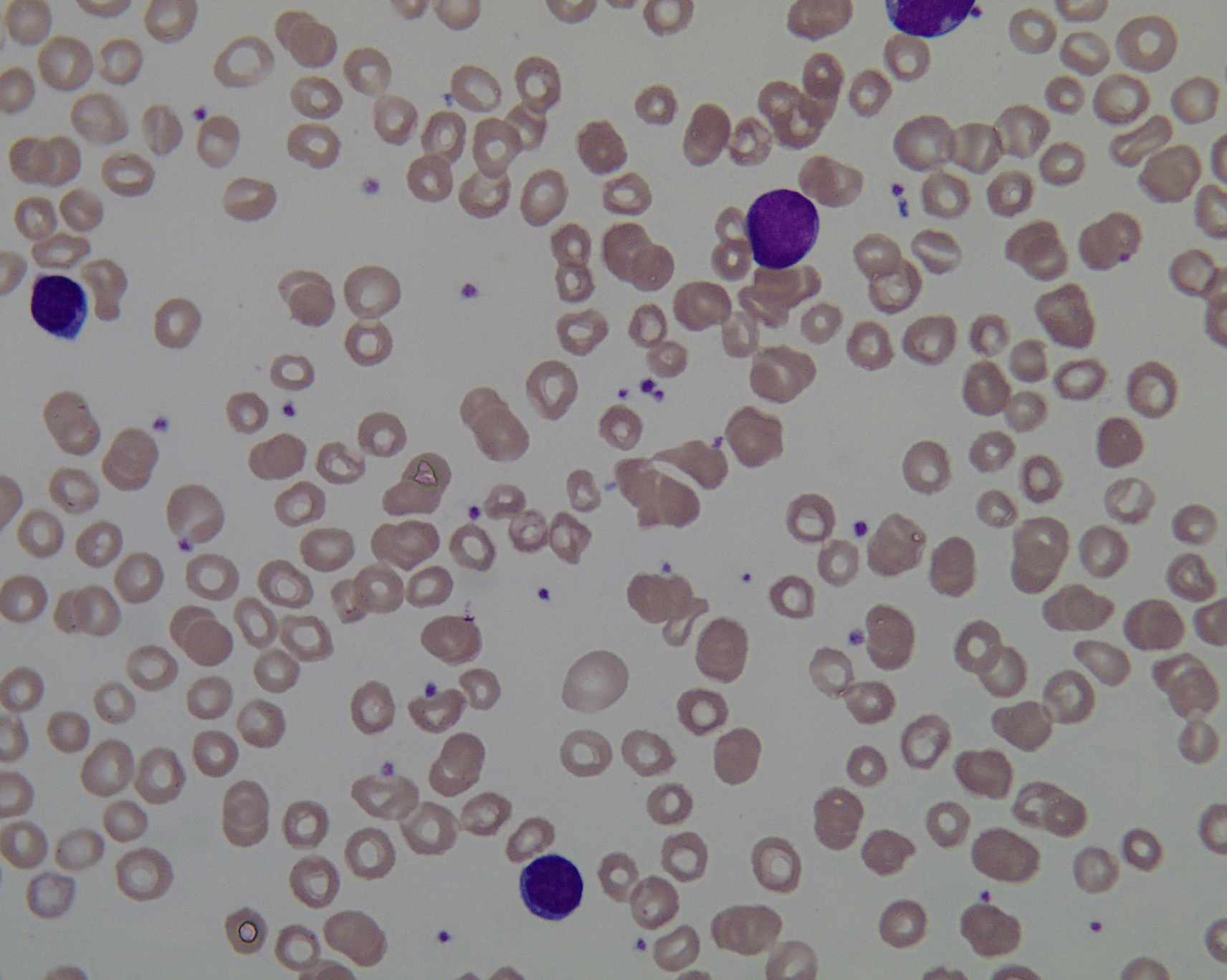

Supplement: Supplementary file 1 — Supplementary Information 1. [file 41598_2025_96918_MOESM1_ESM.zip › ALL_IDB Dataset/L2/Im027_1.jpg]

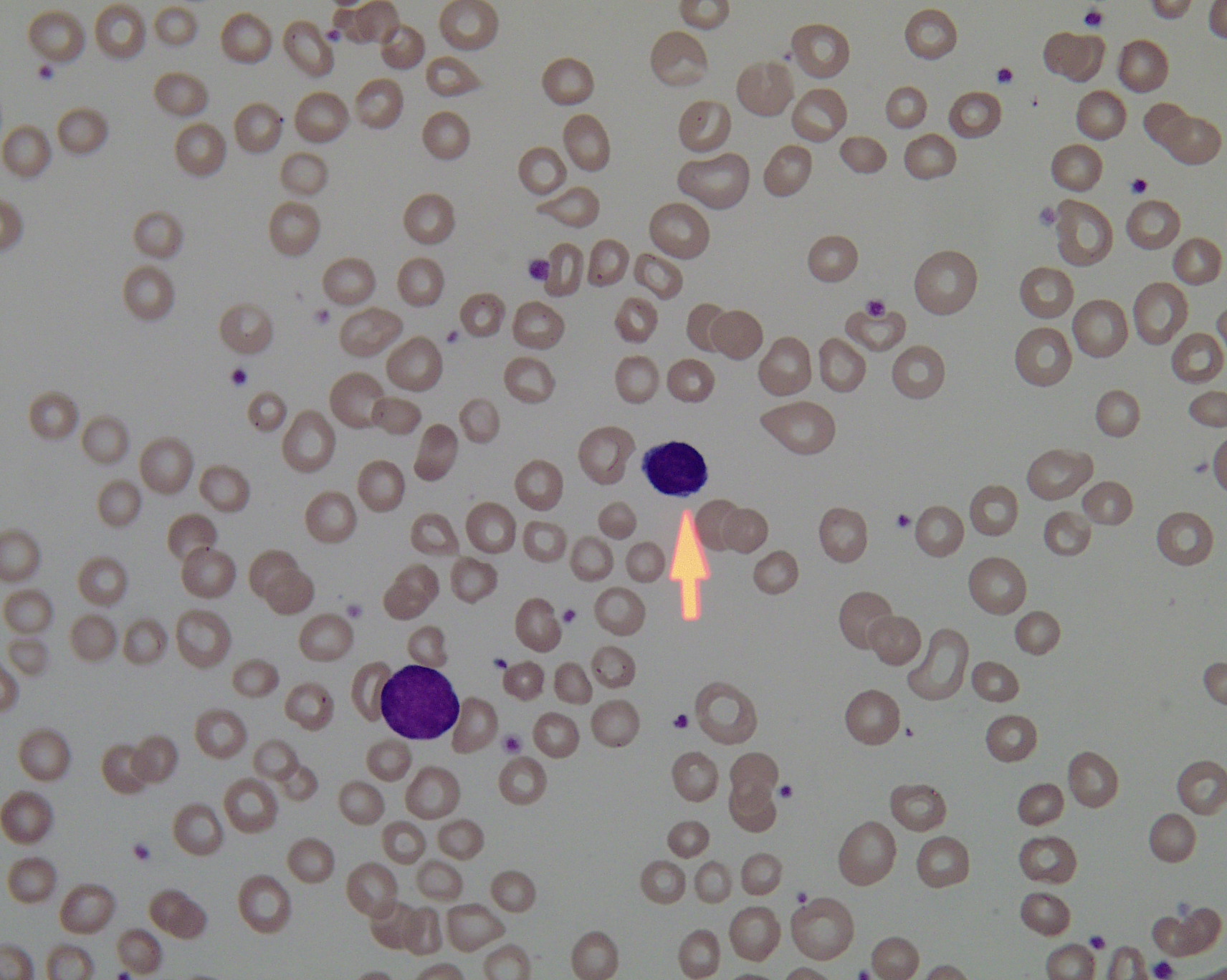

Supplement: Supplementary file 1 — Supplementary Information 1. [file 41598_2025_96918_MOESM1_ESM.zip › ALL_IDB Dataset/L2/Im028_1.jpg]

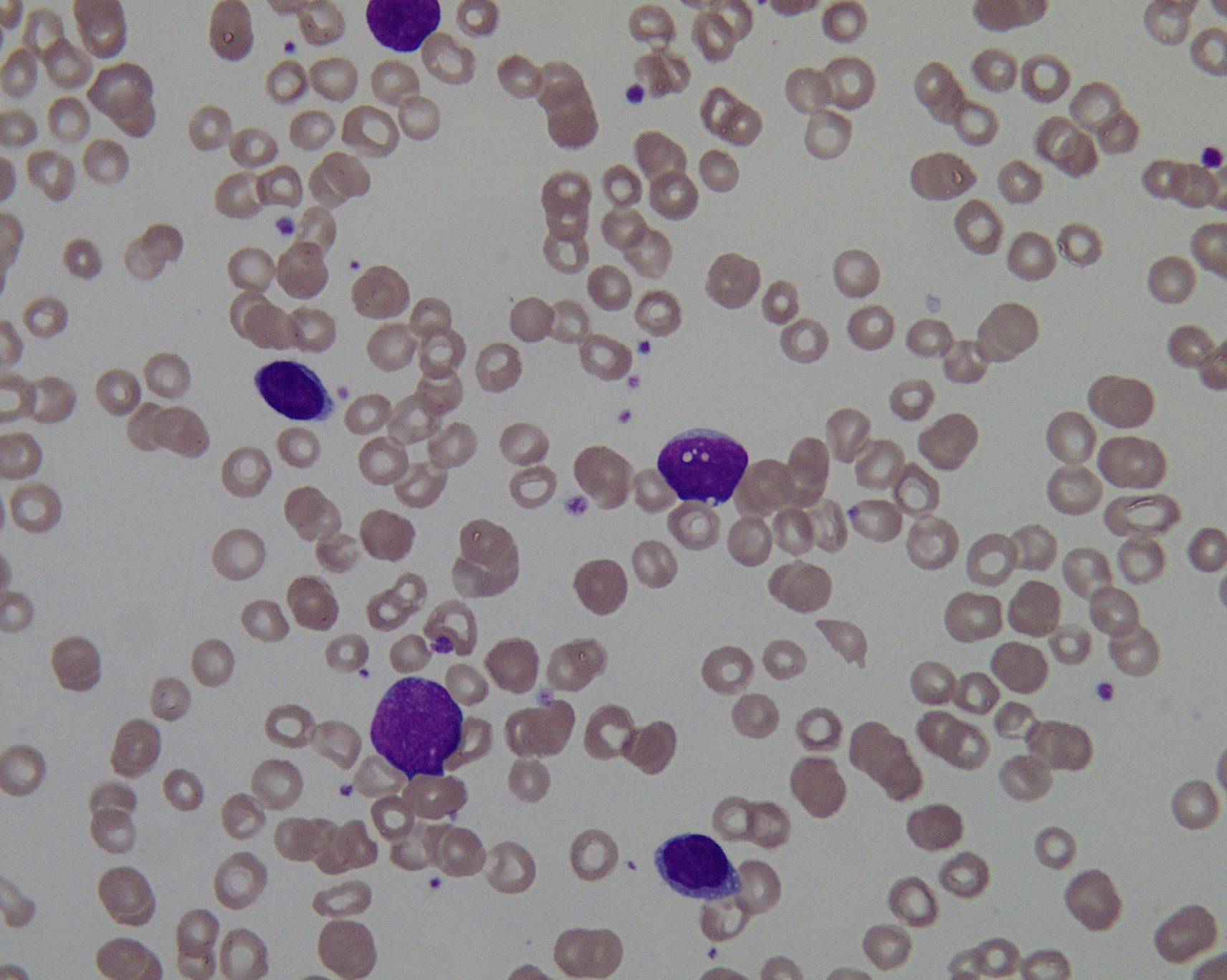

Supplement: Supplementary file 1 — Supplementary Information 1. [file 41598_2025_96918_MOESM1_ESM.zip › ALL_IDB Dataset/L2/Im029_1.jpg]

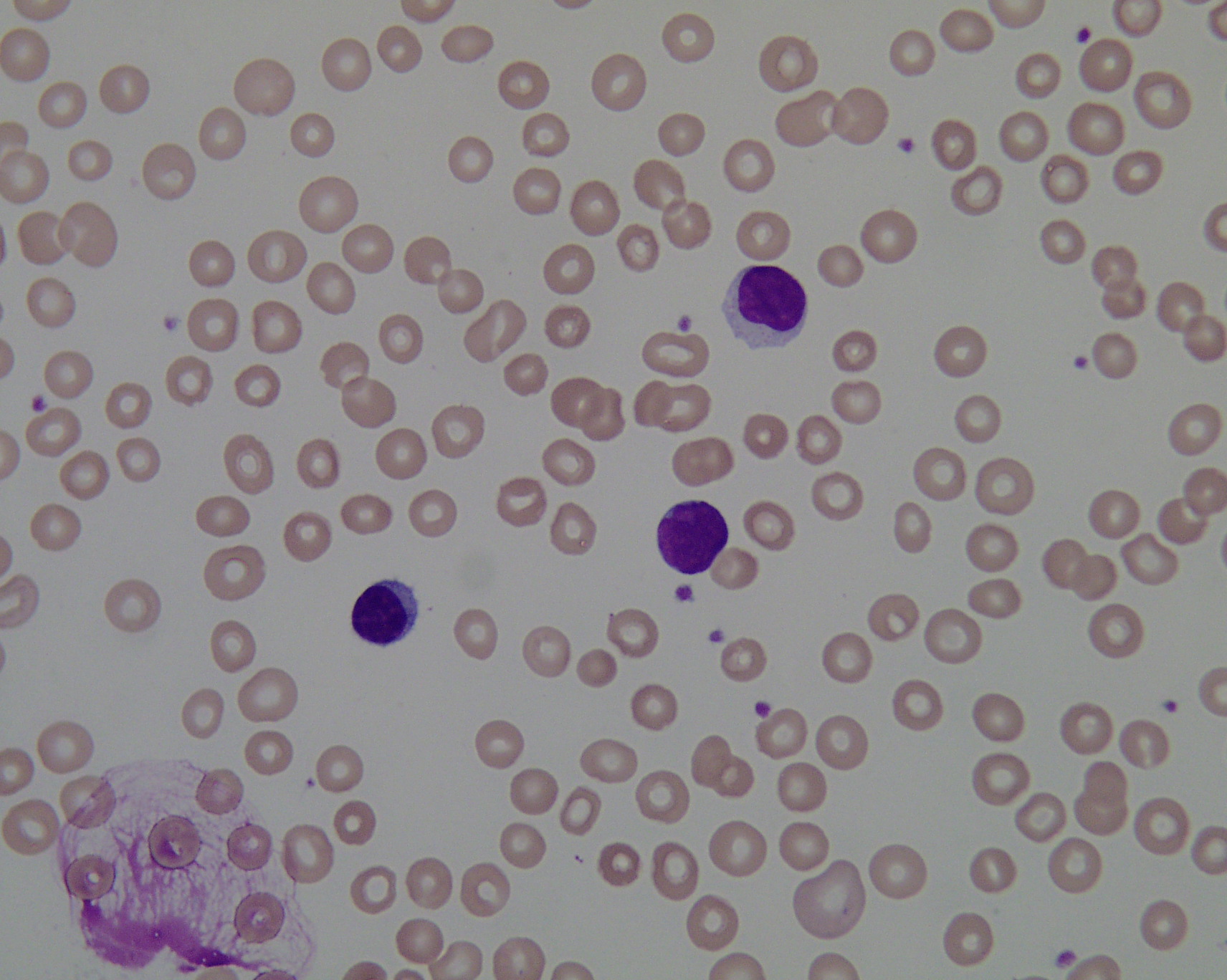

Supplement: Supplementary file 1 — Supplementary Information 1. [file 41598_2025_96918_MOESM1_ESM.zip › ALL_IDB Dataset/L2/Im0300_1.jpg]

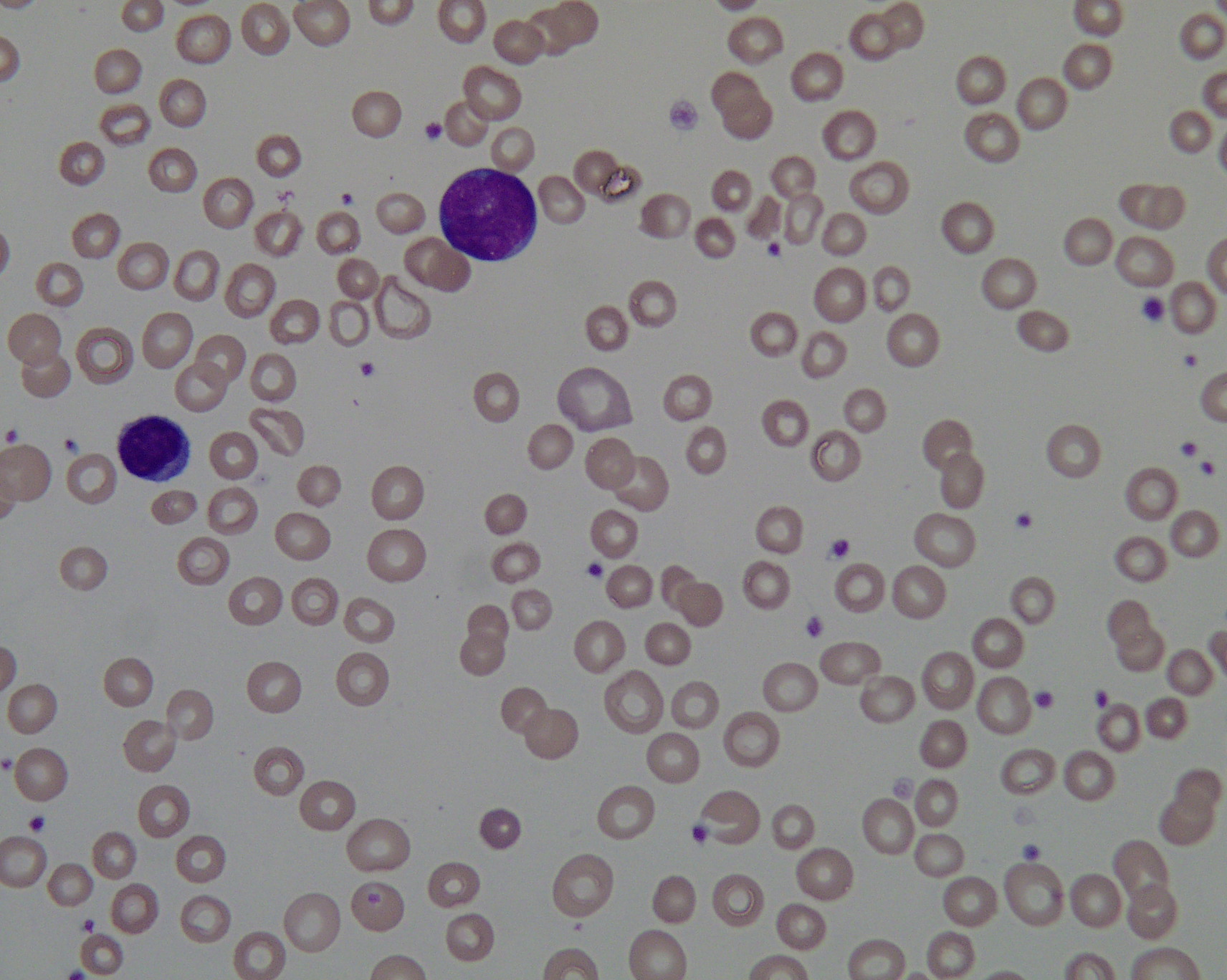

Supplement: Supplementary file 1 — Supplementary Information 1. [file 41598_2025_96918_MOESM1_ESM.zip › ALL_IDB Dataset/L2/Im0310_1.jpg]

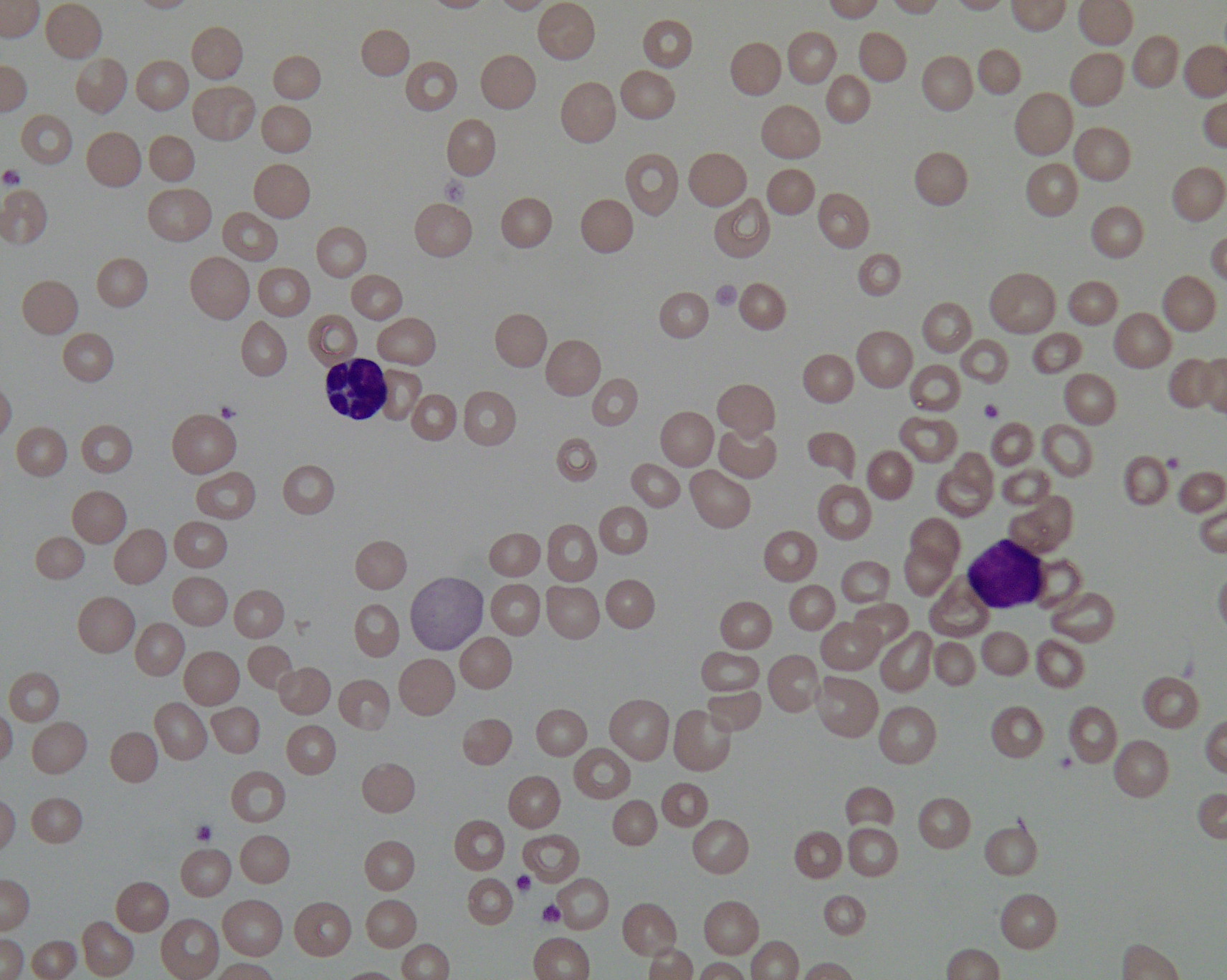

Supplement: Supplementary file 1 — Supplementary Information 1. [file 41598_2025_96918_MOESM1_ESM.zip › ALL_IDB Dataset/L2/Im0321_1.jpg]

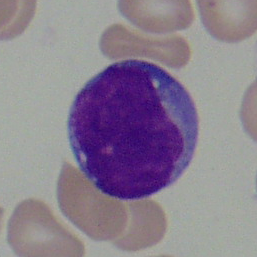

Supplement: Supplementary file 1 — Supplementary Information 1. [file 41598_2025_96918_MOESM1_ESM.zip › ALL_IDB Dataset/L3/Im001_1.tif]

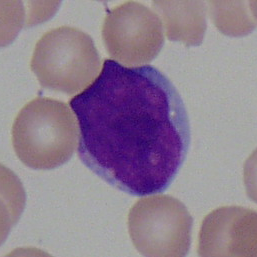

Supplement: Supplementary file 1 — Supplementary Information 1. [file 41598_2025_96918_MOESM1_ESM.zip › ALL_IDB Dataset/L3/Im002_1.tif]

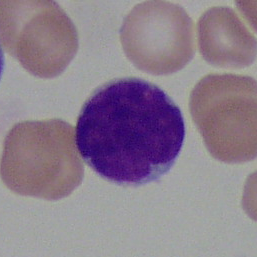

Supplement: Supplementary file 1 — Supplementary Information 1. [file 41598_2025_96918_MOESM1_ESM.zip › ALL_IDB Dataset/L3/Im003_1.tif]

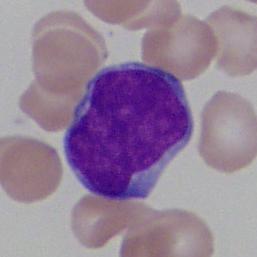

Supplement: Supplementary file 1 — Supplementary Information 1. [file 41598_2025_96918_MOESM1_ESM.zip › ALL_IDB Dataset/L3/Im003_10.jpg]

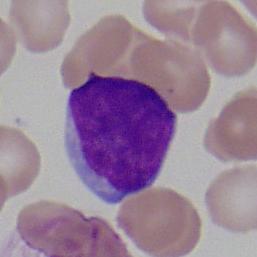

Supplement: Supplementary file 1 — Supplementary Information 1. [file 41598_2025_96918_MOESM1_ESM.zip › ALL_IDB Dataset/L3/Im003_11.jpg]

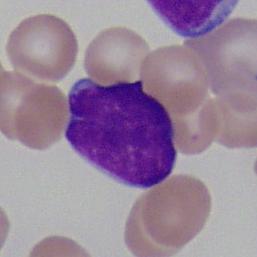

Supplement: Supplementary file 1 — Supplementary Information 1. [file 41598_2025_96918_MOESM1_ESM.zip › ALL_IDB Dataset/L3/Im003_12.jpg]

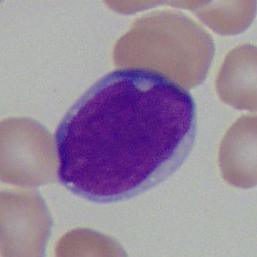

Supplement: Supplementary file 1 — Supplementary Information 1. [file 41598_2025_96918_MOESM1_ESM.zip › ALL_IDB Dataset/L3/Im003_13.jpg]

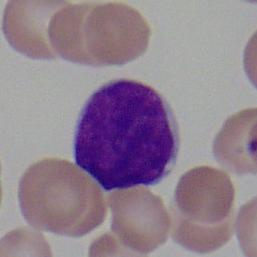

Supplement: Supplementary file 1 — Supplementary Information 1. [file 41598_2025_96918_MOESM1_ESM.zip › ALL_IDB Dataset/L3/Im003_14.jpg]

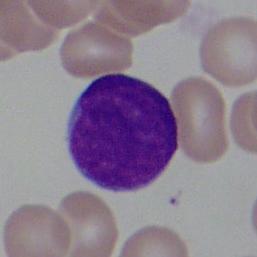

Supplement: Supplementary file 1 — Supplementary Information 1. [file 41598_2025_96918_MOESM1_ESM.zip › ALL_IDB Dataset/L3/Im003_15.jpg]

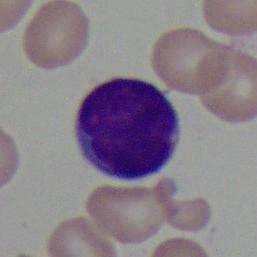

Supplement: Supplementary file 1 — Supplementary Information 1. [file 41598_2025_96918_MOESM1_ESM.zip › ALL_IDB Dataset/L3/Im003_16.jpg]

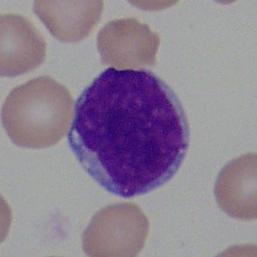

Supplement: Supplementary file 1 — Supplementary Information 1. [file 41598_2025_96918_MOESM1_ESM.zip › ALL_IDB Dataset/L3/Im003_17.jpg]

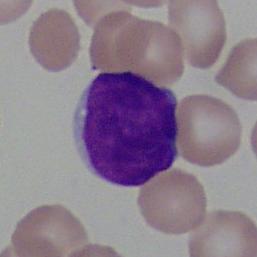

Supplement: Supplementary file 1 — Supplementary Information 1. [file 41598_2025_96918_MOESM1_ESM.zip › ALL_IDB Dataset/L3/Im003_18.jpg]

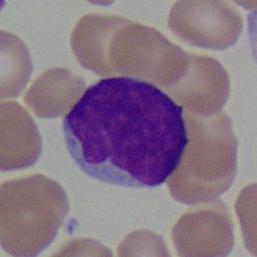

Supplement: Supplementary file 1 — Supplementary Information 1. [file 41598_2025_96918_MOESM1_ESM.zip › ALL_IDB Dataset/L3/Im003_21.jpg]

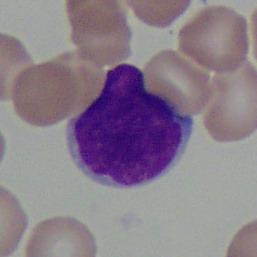

Supplement: Supplementary file 1 — Supplementary Information 1. [file 41598_2025_96918_MOESM1_ESM.zip › ALL_IDB Dataset/L3/Im003_22.jpg]

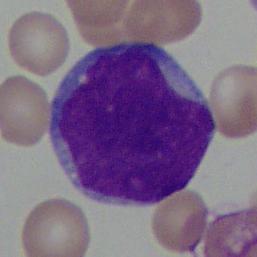

Supplement: Supplementary file 1 — Supplementary Information 1. [file 41598_2025_96918_MOESM1_ESM.zip › ALL_IDB Dataset/L3/Im003_23.jpg]

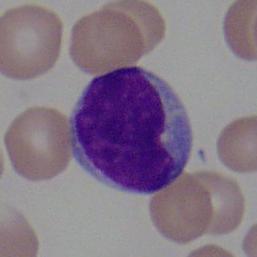

Supplement: Supplementary file 1 — Supplementary Information 1. [file 41598_2025_96918_MOESM1_ESM.zip › ALL_IDB Dataset/L3/Im003_24.jpg]

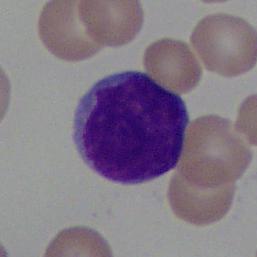

Supplement: Supplementary file 1 — Supplementary Information 1. [file 41598_2025_96918_MOESM1_ESM.zip › ALL_IDB Dataset/L3/Im003_26.jpg]

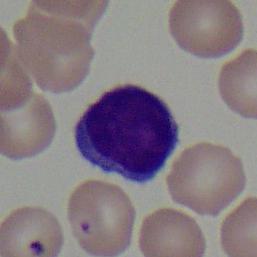

Supplement: Supplementary file 1 — Supplementary Information 1. [file 41598_2025_96918_MOESM1_ESM.zip › ALL_IDB Dataset/L3/Im003_28.jpg]

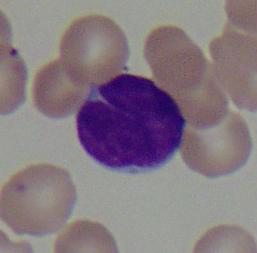

Supplement: Supplementary file 1 — Supplementary Information 1. [file 41598_2025_96918_MOESM1_ESM.zip › ALL_IDB Dataset/L3/Im003_29.jpg]
